# Time-series analysis of satellite imagery for detecting vegetation cover changes in Indonesia

**DOI:** 10.1038/s41598-023-35330-1

**Published:** 2023-05-25

**Authors:** Takuro Furusawa, Takuya Koera, Rikson Siburian, Agung Wicaksono, Kazunari Matsudaira, Yoshinori Ishioka

**Affiliations:** 1 Graduate School of Asian and African Area Studies, Kyoto University, Room #AA431, Research Bldg. No. 2, Yoshida-Honmachi, Sakyo-ku, Kyoto, 606-8501 Japan; 2 Graduate School of Science, University of Tokyo, Tokyo, Japan; 3 GEOINT Department, Satellite Business Division, PASCO CORPORATION, Japan Map Center Bldg., Aobadai 4-9-6, Meguro-ku, Tokyo, 153-0042 Japan; 4 Universitas Sumatera Utara, Medan, 20155 Indonesia; 5 Universitas Gadjah Mada, Yogyakarta, Indonesia; 6 Graduate School of Medicine, University of the Ryukyus, Okinawa, Japan

**Keywords:** Environmental impact, Sustainability

## Abstract

Indonesia has one of the world’s largest tropical forests; thus, its deforestation and environmental degradation are a global concern. This study is the first to perform comprehensive big data analyses with coherent vegetation criteria to measure the vegetation change at a high temporal resolution (every 16-day period) for 20 years and the high administrative resolution (regency or city) all over Indonesia. The normalized difference vegetation index (NDVI) of the Moderate Resolution Imaging Spectroradiometer is analyzed through state space modeling. The findings reveal that the NDVI increases in almost all regencies, except in urban areas. A high correlation between the NDVI change and the time is observed in Sumatra, Papua, and Kalimantan. The gain of the NDVI values is obvious in the Central and Eastern Java Island. Human activities, such as the expansion of agriculture and forestry and forest conservation policies, are the key factors for the observed pattern.

## Introduction

Indonesia has been home to one of the world’s largest tropical forests and holds the largest biodiversity within the greatest landmass in Southeast Asia ^1–3^. However, by the second half of the twentieth century, environmental degradation in Indonesia had become a serious problem in terms of Southeast Asia’s tropical rain forest ^1^. This has become a global concern related to climate change because of its direct connection to global carbon dioxide sinks and greenhouse gas emission ^2,4^.

Despite widespread belief that Indonesia’s tropical rainforests are in a state of continuous decline, recent studies show that vegetation has grown in some administrative units as a result of conservation policies and expansion of forestry and agriculture ^5–10^. The difficulty of assessing various types of vegetation has prevented the disclosure of an overall vegetation trade-off over Indonesia. The lack of knowledge on long-term vegetation change may have misled our understanding of the global climate change, and not just that of Indonesia. Satellite-based time-series analyses examining all available observation with dense periodicity are well-known accurate methods to monitor changes in vegetation for several decades ^4,11–13^. The normalized difference vegetation index (NDVI) of the Moderate Resolution Imaging Spectroradiometer (MODIS) observes every part of Earth on a near-daily basis and provides 16-day composite data of the vegetation index, allowing for the display of continuous vegetation changes ^14–16^. From February 2000 to the present, MOD13Q1 version 6.1 has provided the NDVI at 16-day intervals with a 250 m spatial resolution ^17,18^. These data are ideal for tracking the overall change of vegetation over a long time period and with great spatial precision.

This study measures the vegetation changes at a high temporal resolution (every 16-day period) for 20 years and the high administrative resolution (regency/city) throughout Indonesia. This study also examines how human activities, such as forest protection, agriculture, and those related to climate change, affect the way vegetation changes. Accordingly, special attention is placed on consistency in changes (i.e., patterns brought on by continuous human activities or climate changes) and values in changes (i.e., gains of the vegetation itself). We will describe the long-term vegetation change in Indonesia based on two measures: (1) consistency of the NDVI changes over 20 years, which is herein referred to as the NDVI consistent trend; and (2) comparison of the vegetation index values referred to as the NDVI value change over 20 years.

## Methods

### Regional context

Indonesia (officially the Republic of Indonesia) is the largest archipelago country (over 17,000 islands) and the 14th largest country by area (about 2 million km^2^) in the world ^19^. The archipelago covers tropical rainforest, tropical monsoon, and tropical savanna climates, where there are more than 300 ethnic groups ^19^. Indonesia consists of 38 provinces, and the governance system is decentralized since the end of the twentieth century ^19,20^. In Indonesia, regencies (*kabupaten*) and representative cities (*kota*) are positioned at the same administrative level (level 2); the average size of regencies/cities is 3622 km^2^ (ranging from 10 to 44,013 km^2^). Regencies/cities are administratively separated by geographic conditions and historical (social, cultural, and political) backgrounds, with each regency/city having homogeneous environmental conditions and a socioeconomic status. ^16^.

The main drivers of the rapid deforestation in Indonesia were population growth and economic expansion, including the illegal clearance of forests ^21,22^. Indonesia’s economy has grown by > 5% per year on average since 2000 until the Covid-19 pandemic; GDP PPP (international dollars) increased about three times from 1 Trillion USD in 2000 to 3.33 Trillion USD in 2019 ^23^. Additionally, its population has increased by 1.14% annually, leading to an approximately 30% growth during this period (i.e., from 211 Million in 2000 to 273 Million in 2020), making it the most populous and prosperous country in Southeast Asia ^19^. Indonesia is also one of the largest emitters of greenhouse gases (GHG) ^24^.

In contrast, the Indonesian government is committed to unconditionally reduce its GHG emission by 29% and up to 41% if international assistance for finance, technology transfer, and capacity building are provided^25^. The land-use and energy sectors are to minimize the national GHG emission. Therefore, Indonesia first introduced moratorium on new forest clearance in 2011 ^26^ and made it permanent in 2019 ^3^. This moratorium mainly targeted Kalimantan, Sumatra, and Papua (New Guinea), which had been center of deforestation in the beginning of 2000s ^3,27^.

Meanwhile, agriculture has expanded in this century, with the primary export goods (e.g., oil palm and rubber plantations) coming from Sumatra and Kalimantan ^5^. Conversely, since the turn of the century, sustainable forestry policies in Java have caused a surge in the forestation of local smallholders ^6^. There are indications that forests on Java Island are recovering ^7,8^. Due to improved forestry policies and reforestation activities, the deforestation rate has decreased since 2011 ^9^. Deforestation has been reported from all over Indonesia, but the causes of deforestation were different from one region to another because of geographic conditions and socioeconomic statuses ^28^.

### NDVI at the regency/city level

Many studies have demonstrated that the NDVI is related to the leaf area, green biomass, percent green cover, and a fraction of absorbed photosynthetically active radiation (fAPAR) ^18,29,30^; moreover, NDVI is a global-based vegetation index. We utilized the product of NASA's MODIS Terra Program version 6.1. This product provides data at 16-day intervals (i.e., composite data for a 16-day period derived from images that are acquired almost every day) at 250 m spatial resolution after correction for atmospheric effects (aerosols and gases) and sensor degradation, angular consideration, and minimization of the influence of daily cloud covers for consistent spatial and temporal comparisons of vegetation ^17,18^. The NDVI runs from − 1 to + 1 (acceptable range for the NDVI of the MODIS: from − 0.2 to + 1) and is determined from the visible and near-infrared light reflected by the quantity (biomass) and/or composition of vegetation.

The information used was from LP DAAC, a part of NASA’s Earth Observing System Data and Information System run in cooperation between the US Geological Survey and NASA. We utilized the shapefile of Administrative Level 2 in April 2020 created by the Indonesian Bureau of Statistics (BPS). This was made available through the Humanitarian Data Exchange program (HDX) of the United Nations Office for Coordination of Humanitarian Affairs (OCHA) (https://data.humdata.org/) ^31^. The Administrative Level 2 consisted of 93 cities (*kota*), five administrative cities (*kota administrasi*), 415 regencies (*kabupaten*), and one administrative regency (*kabupaten administrasi*) (Supplementary Table 1). Each MOD13Q1 picture was dissected for the Administrative Level 2 and pixel reliability (− 1: no data; 0: excellent data; 1: poor data; 2: snow/ice; and 3: cloud). The coastal regions, where the pixels had recorded water cover, experienced the pixel reliability of MOD13Q1 with − 1 (no data). The NDVI was unreliable when the pixels were classified as level 2 (snow/ice). No NDVI was available for the pixels covered in clouds (pixel reliability = 3). These cases were disregarded from further analysis. Hence, the average NDVI was determined for regions in each regency/city at each time point with a pixel reliability range of 0 or 1. Seribu Islands was the sole administrative regency, which was omitted from the studies because it was composed of a number of tiny islands, and all pixels covering it had some sea in them. Accordingly, further evaluations were conducted for 513 regencies and cities throughout a 20-year span (i.e., every 16 days; 460 time points). We downloaded a total of 5520 pictures since 12 MOD13Q1 images at each time point cover the whole Indonesia.

### Time-series analyses

We split the NDVI changes in the MOD13Q1 data into trends, seasonal changes, and residuals using a stochastic level and deterministic seasonal state space model (SSM). We performed time-series studies based on SSM ^32^ using two steps ^16^: (1) the NDVI data were averaged for each geographic unit (regency or city); and (2) stochastic level and deterministic seasonal models were used. The time-series change in this model was divided into trends, cycles, and residuals while excluding noises. The slopes and the levels were determined by a stochastic process, seasonal changes (annual cycle), and irregular changes with interporation of missing data smoothed by the Kalman filter. The maximum likelihood estimation was made for the following equations:

$${y}_{t}={\mu }_{t}+ {\gamma }_{t}+ {\varepsilon }_{t}, {\varepsilon }_{t}\sim \mathrm{NID} \left(0, {\sigma }_{\varepsilon }^{2}\right)$$

$${\mu }_{t+1}={\mu }_{t}+{\xi }_{t}, { \xi }_{t}\sim \mathrm{NID}(0,{\sigma }_{\xi }^{2})$$

$${\gamma }_{1, t+1}= {-\gamma }_{1, t} {-\gamma }_{2, t}\dots {-\gamma }_{22, t}$$

$${\gamma }_{2, t+1}= {\gamma }_{1, t}$$

$$\dots$$

$${\gamma }_{22, t+1}= {\gamma }_{21, t}$$

for *t* = 1, *n*, where ${y}_{t}$ is the observation (NDVI) at time *t*; ${\mu }_{t}$ is the unobserved level at time *t*; ${\gamma }_{t}= {\gamma }_{1, t}$ denotes the seasonal component; ${\varepsilon }_{t}$ is the observation disturbance term at time *t*; and ${\xi }_{t}$ is called the level disturbance term at time *t*. The level ${\mu }_{t}$ was allowed to vary over time in the stochastic level and deterministic seasonal model. The seasonal changes, trends, and residuals are represented by *γ*, *μ*, and *ε*, respectively. R Software version 4.1.2 with “dlm” package was used for the analysis ^33^.

### Precipitation data

The USGS Earth Resources Observation and Science Center and the Climate Hazard Center of the University of California in Santa Barbara developed climate hazards group infrared precipitation with station (CHIRPS) v2p0, which provide data on rainfall estimates from rain gauge and satellite observations and is available for the entire world, including areas with sparse surface data ^34^. CHIRPS provide moderate resolution (0.05°) gridded precipitation information. We obtained the CHIRPS monthly data for Indonesia from 2001 to 2020 and masked them at the administrative 1 (province) level (data compiled by Indonesian Statistical Office and available at OCHA HDX) because the resolution of CHIRPS is coarser than that of MOD13Q1. Using the same SSM model with the NDVI data, we decomposed the precipitation data into trends and seasonal cycles while excluding noises. Furthermore, the monthly average precipitation for each province over the course of 20 years was calculated from this dataset (“rainfall level”).

### Socioeconomic data

We used the population density data and the GDP at the regency/city level issued by the Ministry of Internal Affairs of the Republic of Indonesia ^35^. However, we were unable to study the time-series changes in socioeconomic development over a 20-year period because the administrative units increased from 397 in 2001 to 514 in 2020 due to the administrative reforms brought about by population and economic growth. The data used in this study were (1) the population densities in 2020, (2) GDP proportion from agriculture, forestry, and fisheries (as an indicator of the land-use intensities for agricultural and forestry development), and (3) GDP proportion from financial and insurance activities (as urban development). These indicators reflected the socioeconomic conditions of Indonesia, where the inequality in development among regions is very high. The total GDP was not used because it was closely correlated with the three variables used in this work.

### Field observation

After obtaining results of time-series analyses, we also conducted field observations in 2022 in North Sumatra, the Special Capital Region of Jakarta, Central Java, the Special Region of Yogyakarta, and South Sulawesi. We visited regencies and cities showing very consistent increases or decreases in NDVI, rapid loss or growth in NDVI, or dramatically irregular changes (e.g., disasters); furthermore, we observed the reasons behind such changes. In addition, we observed vegetation changes between 2014 and 2017 in parts of East Nusa Tenggara ^16^.

### Statistical analyses

We defined the Pearson’s correlation coefficients of the NDVI trend (after noise and cycle elimination) with time (every 16 day) as the “NDVI consistent trend.” Furthermore, we defined the NDVI variation between 2001 and 2020 (in other words, differences in the average NDVI between 2020 and 2001) as the “NDVI value change.” Pettitt’s Test was used to identify the trend change-points ^36^. For precipitation, the correlation of the trend with time and the difference in the NDVI between 2020 and 2001 were also computed (CHIPRS data).

We used classification and regression trees (CART), a decision tree model data mining method that explains how a target variable is predicted by other variables based on categorizing samples into binary classes ^37^; exponential was used for NDVI consistent trend. The decision tree regression analysis was conducted to explore factors contributing to the consistent trend of the NDVI and the NDVI value changes. To minimize overfitting, the complexity parameter (cp) value for the biggest cross-validated prediction error of less than the minimal relative error plus the cross-validated prediction standard deviation was used as the cut-off (pruning tree model). To reflect the differences in agricultural intensities and main tree crops, all regencies and cities were classified into Sumatra, Western Kalimantan, Eastern Kalimantan, Western Java, Eastern Java, Nusa Tenggara, Northern Sulawesi, Southern Sulawesi, Maluku, and Papua (Supplementary Fig. 1). QGIS 3.22.4 Białowieża (QGIS Development Team) (https://qgis.org/) was utilized for the map creation.

## Results

### Overall patterns

**Figure 1:**
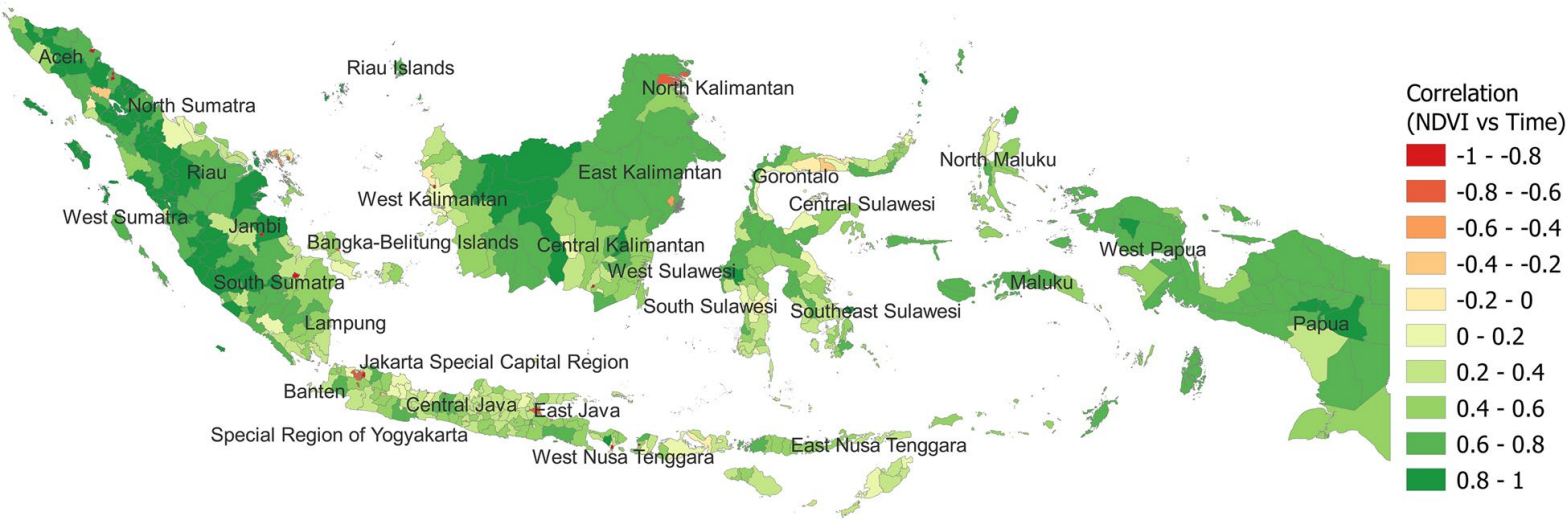
Correlation (“NDVI consistent trend”) between the NDVI trend and time in every 16-day period from January 1, 2001 to December 19, 2020. This map was created by TF using QGIS 3.22.4 Białowieża software and open administrative boundary data published by OCHA HDX.

The predicted NDVI values (i.e., the trend including level disturbance term plus seasonal fluctuations) and the NDVI trend by the SSM, respectively, for all regencies/cities are available in Supplementary Information (Figs. S2–S3). Out of the 513 regencies/cities (i.e., 415 regencies, 93 cities, and five administrative cities), 442 districts (86.2%; equivalent to 97.1% of the country’s land area) showed consistent positive NDVI trends (i.e., significantly positive correlation of NDVI with time; Pearson’s correlation coefficient > 0 and *P* < 0.05); 43 had consistent negative NDVI trends (8.4%; only 0.9% of the land area); and 28 (5.5%) had no significant correlation. Figure 1 shows the NDVI consistent trend (green: positive trend; red: negative trend). The Sumatra, Kalimantan, and Papua islands were expected to have positive correlations (green dense), while urban regions such as the Special Capital Region of Jakarta showed strong negative correlations (red). The trend was mildly favorable (light green) in areas with high population density, monsoon climate (e.g., Central and Eastern Java Island and Southern Sulawesi Island), and savanna areas (Nusa Tenggara).

**Figure 2:**
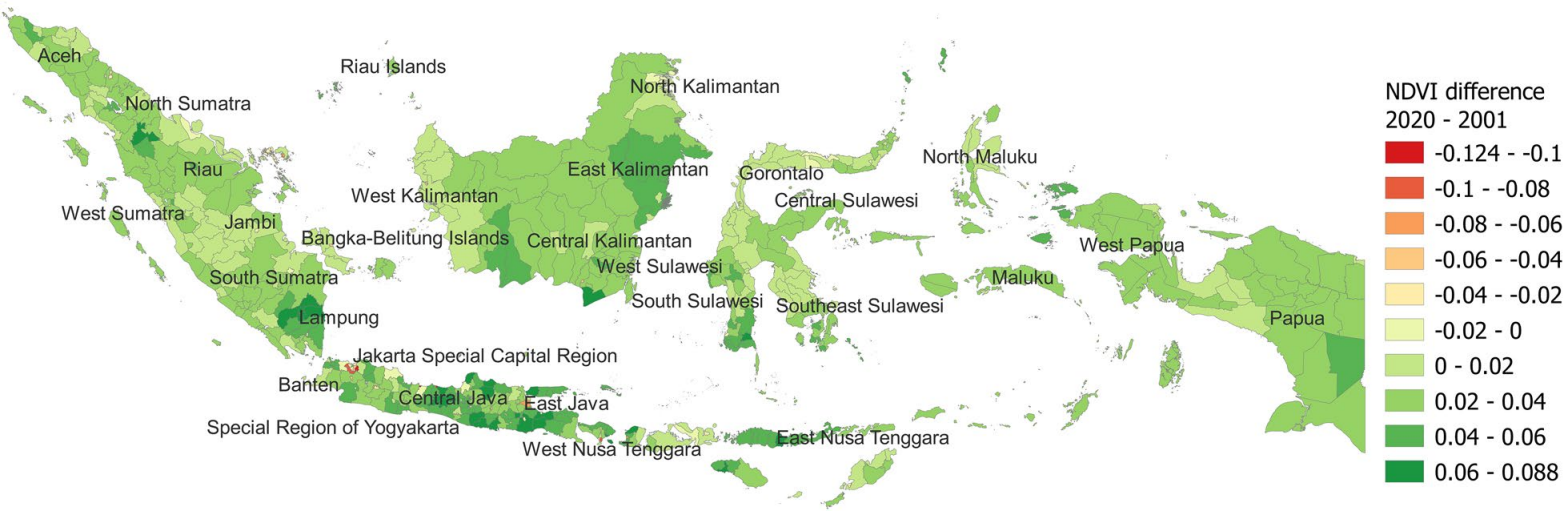
Increase or decrease (NDVI value change) in the NDVI values from 2001 to 2020. This map was created by TF using QGIS 3.22.4 Białowieża software and open administrative boundary data published by OCHA HDX.

The average NDVI for each year from 2001 to 2020 is displayed after seasonal cycles, and residuals have been considered (Supplementary Fig. 4). A total of 463 (90.3%) regencies/cities displayed an increase in the NDVI. This increase was visible not only in East Kalimantan, Lampung, South Sulawesi, and Papua, which had tropical rain forest climates, but also in Central and Eastern Java and Nusa Tenggara, which had tropical monsoon or savanna climates (Fig. 2). Notably, the pattern in the NDVI value change was partially different from that in the NDVI consistent trend.

**Figure 3:**
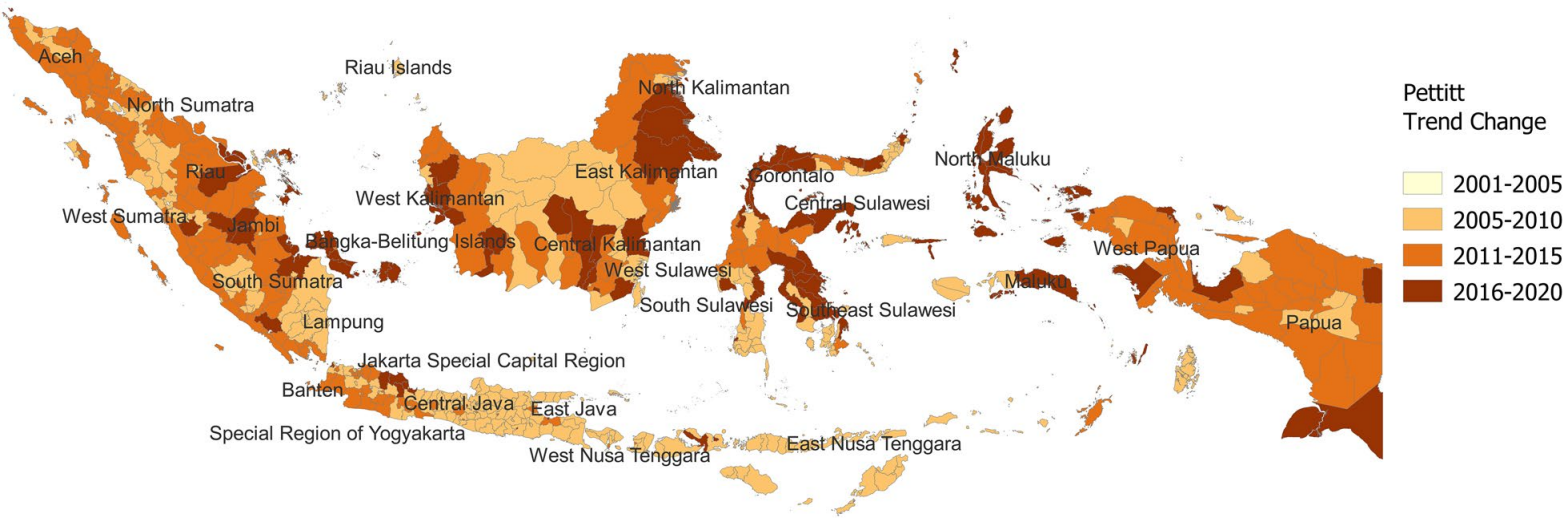
Pettitt's Test for Change-point Detection. The darker orange color represents the recent trend change. The gray color indicates no significance in Pettitt’s Test. This map was created by TF using QGIS 3.22.4 Białowieża software and open administrative boundary data published by OCHA HDX.

Figure 3 shows the results of Pettitt’s Test for Change-point Detection. The NDVI change started in an earlier period (before 2010) in Central and Eastern Java and Nusa Tenggara and in a later period (after 2010) in many parts of Sumatra, Papua, Western Java, and Kalimantan.

### Impact of disasters

**Figure 4:**
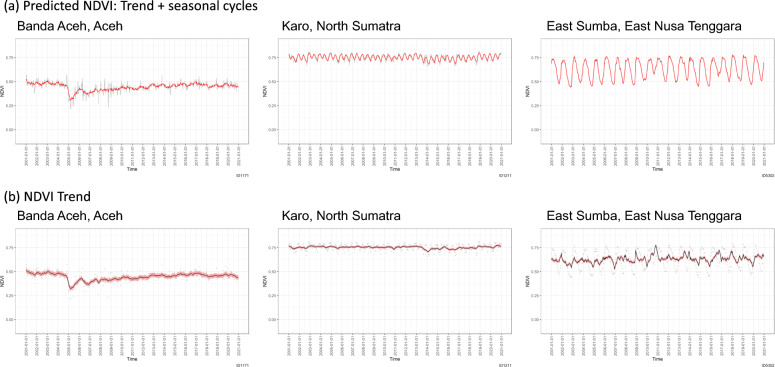
Predicted NDVI (**a**: red line = predicted NDVI; gray line = observed NDVI) and NDVI trends (**b**: black line = NDVI trend; red zone = 95% confidence limits; gray dots = observed NDVI) in association with disasters. Banda Aceh City experienced the 2004 Indian Ocean earthquake and tsunami. Karo experienced a series of Volcano Sinabung eruptions. East Sumba eruptions abnormal rainfall and drought.

**Figure 5:**
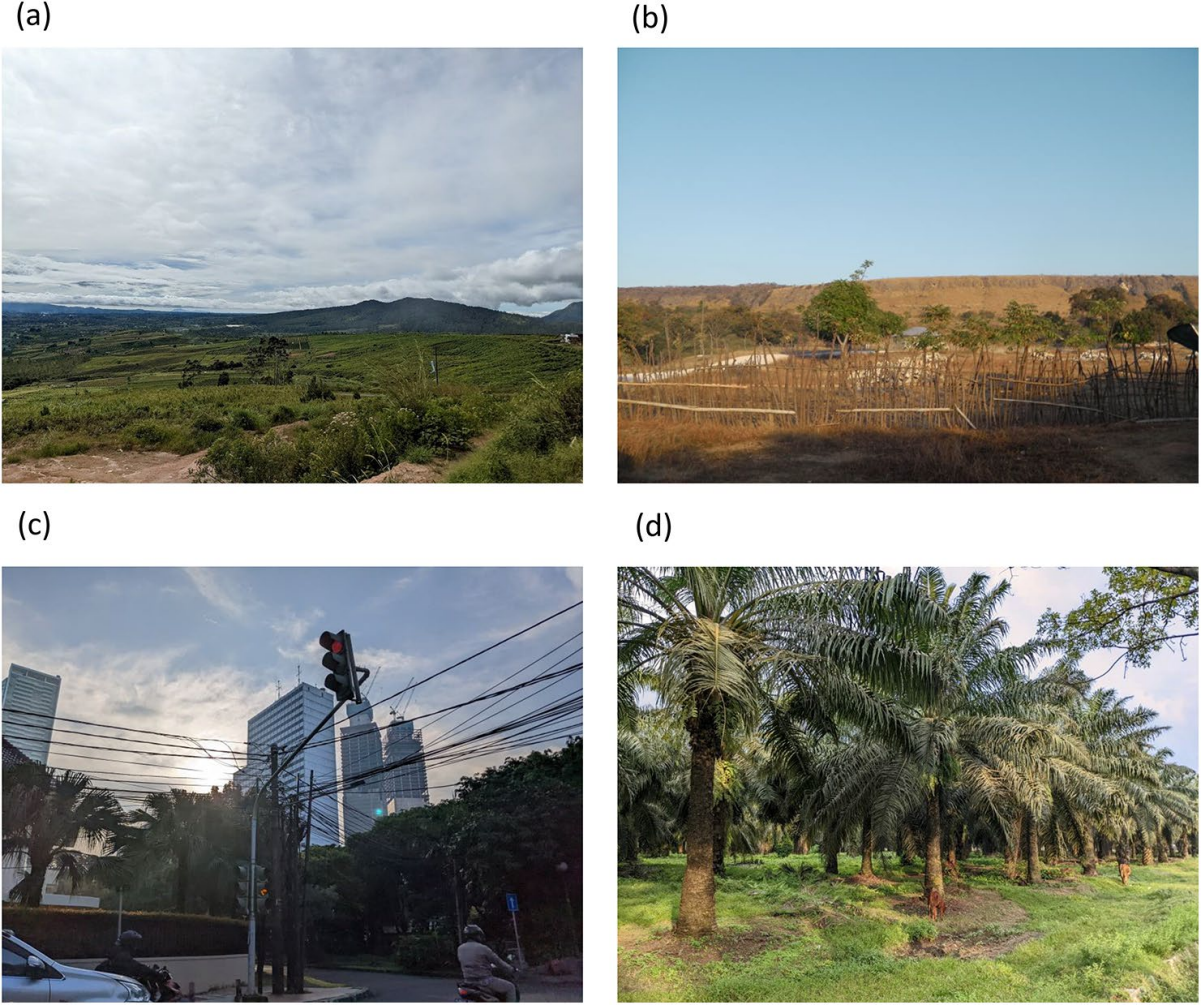
Photos of (**a**) agricultural expansion in lands devastated by a volcano eruption 7 years ago in Karo Regency (North Sumatra, June 2022), (**b**) land during a dry season in Eastern Sumba Regency (East Nusa Tenggara, August 2012), (**c**) greening of the Central Jakarta City (Special Capital Region of Jakarta, May 2022), and (**d**) oil palm plantations growing in a former paddy field in Serbang Bedagai Regency (North Sumatra, June 2022). All photos were taken by TF.

Disasters affect vegetation. Figure 4 shows the example cases of three regencies/cities. The NDVI in Banda Aceh City, which is the capital city of Aceh Province, dramatically decreased when the 2004 Indian Ocean earthquake and tsunami flushed most of the city’s greenery. When Volcano Sinabung erupted and its lava and pyroclastic flow destroyed vegetation in the mountain ranges, the NDVI also decreased in Karo Regency in North Sumatra Province (e.g., 29th August 2010, series of eruptions in 2014, and a massive eruption on 21st May 2015). These cases showed that the lost vegetation rapidly recovered, depicting a regrowth of more than 60% of the loss in the following year (Fig. 5a). However, the NDVI in Banda Aceh City took 12 years to return to its pre-disaster state. East Sumba, which is one of the driest environments in Indonesia, represents steep seasonal NDVI changes. However, irregularities were observed in 2010 and 2016 when rainfall continued, even in the dry seasons, and in 2015 and 2019 when a severe drought caused social problems (Fig. 5b). These extreme weather events spiked the trend, but did not affect the NDVI in the years that followed. The NDVI anomalies were also more apparent in drier environments with substantial seasonal cyclic changes (tropical monsoon and savanna) than in tropical rainforest climates with moderate seasonal changes.

### Effects of the population and industries

All of the top 10 regencies/cities with a highly positive NDVI consistent trend (Supplementary Table 2) were located in Sumatra Island. North Padang Lawas (*r* = 0.990) and Batu Bara (*r* = 0.982) in North Sumatra Province are known for their large-scale agriculture. Toba Samosir (*r* = 0.952) in North Sumatra and Lebong (*r* = 0.964) in Bengkulu Province are popular in forestry operations in addition to a disturbed or protected tropical rain forest. All extremely negative trends, however, were found in large cities like Jambi City (*r* =  − 0.996) in Jambi Province, Pekalongan City (*r* =  − 0.994) in Central Java Province, Langsa City (*r* =  − 0.981) in Aceh Province, and Palembang City (*r* =  − 0.956) in South Sumatra. On the contrary, the Central Jakarta Administrative City showed a positive trend (*r* = 0.85) because it was overburdened with urban infrastructure in the twentieth century (Fig. 5c). Consequently, urban growth has shifted to nearby areas (e.g., South Jakarta Administrative City: *r* =  − 0.61). The population density and the GDP proportions from financial and insurance activities, agriculture, forestry, and fisheries are shown in the maps in Supplementary Information (Fig. S5).

In terms of the NDVI gain (i.e., an increase in the NDVI value changes; Supplementary Table 3), the land-use change in agricultural lands from rice paddy fields to forests and/or oil palm plantations logically increased the NDVI because the vegetation loss during the off-season (i.e., scarce vegetation in the dry season) disappeared (Fig. 5d). These effects were visible in the NDVI gain in Central Java and Sumatra.

### Correlation with climatic, demographic, and economic variables

Finally, the factors for the NDVI consistent trend and the NDVI value change were analyzed. The univariate correlation matrix output showed that the NDVI consistent trend was positively correlated with the proportions of agriculture, forestry, and fisheries in the GDP and the rainfall level (monthly average) and negatively correlated with the rainfall correlation with time and population density (Supplementary Fig. 9). The NDVI value change was positively correlated with the proportions of agriculture, forestry, and fishing in the GDP and the rainfall change from 2001 to 2020 and negatively correlated with the population density and the proportion of financial and insurance activities in the GDP. However, these variables were interrelated.

**Figure 6:**
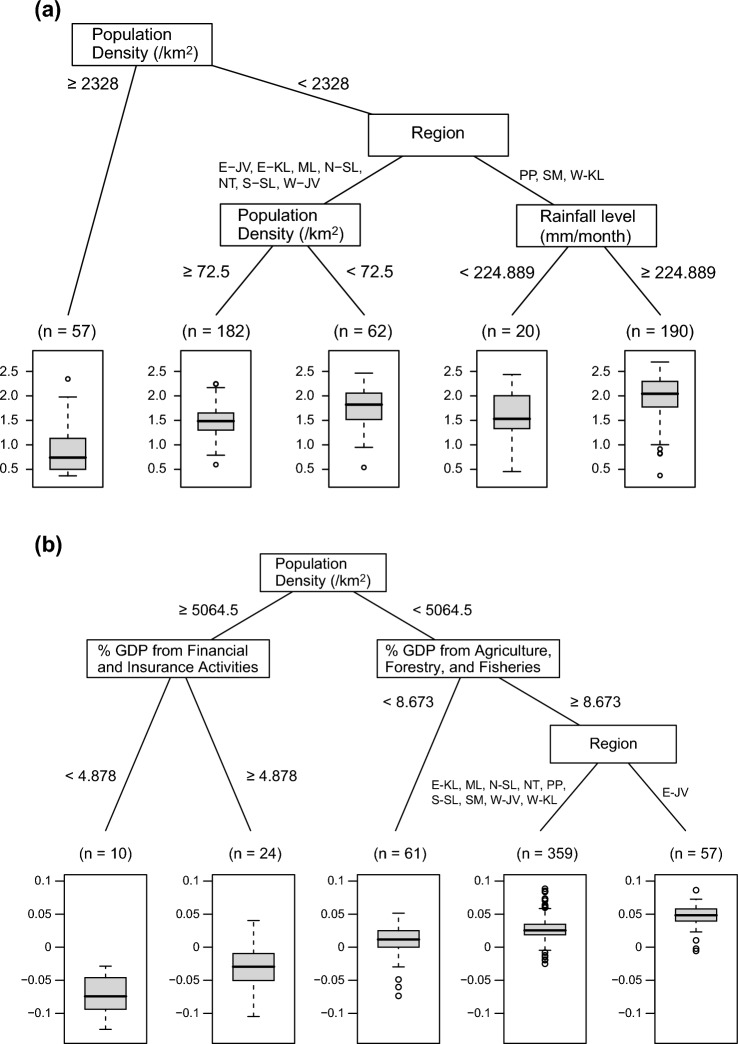
Classification and regression tree model for the (**a**) exponential of NDVI consistent trend (i.e., correlation coefficient between the NDVI and time) and (**b**) NDVI value change (i.e., difference of the NDVI between 2001 and 2020).

We also performed decision tree regression analyses (CART). The population density showed the greatest influence on exponential of the NDVI consistent trend (Fig. 6a). A population density ≥ 2328/km^2^ was classified as the lowest group. The regencies in Papua, Sumatra, and Western Kalimantan were segregated from the other regions in the group with lower population densities. Provinces with high rainfall levels (239.772 mm per month) exhibited the highest NDVI consistent trend in the former group (i.e., Papua, Sumatra, and Western Kalimantan), whereas the lowest population density was a beneficial feature in the latter group.

The population density was also the key determinant of the NDVI value change (Fig. 6b). The regencies with population densities greater than 5064.5/km^2^ showed negative NDVI value changes. Interestingly, however, cities with higher GDP income from financial and insurance activities (4.878%) exhibited better NDVI value changes. The GDP from agriculture, forestry, and fisheries was categorized with a higher NDVI gain in sparsely populated regencies at 8.673%. The biggest NDVI gain was found in the Central and Eastern Java regencies.

## Discussion

The high temporal and geographical resolutions study over Indonesia revealed that the NDVI increased in most regencies in the last 20 years. The NDVI increase was remarkable in Eastern Java Island and parts of Kalimantan; furthermore, this increasing trend was consistent in Sumatra, Kalimantan, and Papua. The NDVI decreased mainly in cities. The population densities, industries, rainfall, and regional differences were the major contributors to these changes.

Although the NDVI reflected a horizontal change of vegetation and vegetation activity, this study did not measure the vertical vegetation structure or biomass. Therefore, this study’s interpretation cannot be directly applied to GHG emissions or sink. In addition, we averaged the NDVI at administrative level 2 so as to match the vegetation change with socioeconomic and climate variables and to omit effects of cloud cover (no data pixel), but this method may have overlooked heterogeneity in each regency/city. Due to limitations in the availability of rainfall data, we used different spatial resolutions for the NDVI and the rainfall. We adopted this method because (i) our study prioritized the high temporal resolution (16-day period over 20 years) of the MODIS and (ii) the regency/city level represents good spatial resolution to match the vegetation change with socioeconomic and climate variables.

Despite these limitations, this work clarified the high-temporal-resolution pattern in the changes in the vegetation density and the trade-off between the demographic and industrial developments country-wide in Indonesia and its environmental policies and climatic changes. Even though natural forest covers decreased because of logging, fiber, oil palm, mining, and mix concession between 2001 and 2010 ^1,2^, the human-made vegetation improved because of the expansion of forestry (reforestation) and agriculture, including plantations, during this period ^9^. The deforestation on Java Island slowed until the 1960s and the forest cover began recovering in the 1970s as a result of the reforestation campaign policy, elevated prices of agricultural products, and rural depopulation ^38^. A study of time-series analyses of MODIS on one watershed in East Java showed that the forest increased because of transformation from rice paddies, upland, mixed gardens, and plantations from 2002 to 2011 ^8^. Reforestation and plantations were expanded from governmental to private lands, as well ^6,39,40^.

Although previous studies ^16,41^ have suggested that vegetation anomalies are correlated with the rainfall pattern as a result of the extreme weather events in Indonesia, such a correlation occurred only for short periods (occasional extreme weather events) and this vegetation change discontinues as long as the rainfall anomalies discontinue. The tropical monsoon climate with a lower baseline NDVI than the tropical rain forest climate contributed to the apparent increase in the NDVI. Extreme weather events like the El Nino (ENSO) and La Nina phenomena have increased in frequency over the last two decades ^42^, although their impacts vary by province considering various agricultural systems ^43^. Dryland farming, forestry, and natural vegetation are especially vulnerable on limestone terrains, such as in East Nusa Tenggara and Central Java ^44,45^. A previous satellite-based time-series analysis on a part of Java showed that a rapid forest increase was observed only in 2010—the year of abnormally high rainfall ^8^. If people had dams (e.g., Bilibili Dam in South Sulawesi) or traditional water management systems (e.g., *subak* in Bali and *selokan mataram* in Yogyakarta), they could control the water resources based on extreme weather events or agricultural necessities (e.g., multiple harvests per year for rice or dryland farming). The anomalies caused by rainfall and other natural disasters have affected the periodical patterns, but rarely impact the general pattern of increase.

In contrast, the NDVI increase was very consistent in Sumatra, Papua, and Western Kalimantan. These regions are characterized by tropical rain forests with high precipitation and the expansion of large-scale tropical agriculture involving oil palm ^5^. Oil palm plantations in Sumatra have spread from corporations to smallholders ^46^. In addition, these regions comprise several forest conservation and forestry zones targeted in a moratorium on logging concessions ^1,47,48^. In Indonesia, socioeconomic, biophysical, and ecological factors influence the expansion and the intensity of smallholder agriculture and forestry ^49,50^.

Previous studies suggested that that Java Island and parts of Indonesia are hotspots of increased built-up areas ^4^. Although the land sizes of these cities are small, cities with large population densities contributed to the vegetation loss. The demographic trend in Indonesia has been urbanization or rural–urban migration, which has improved the rural life by easing resource pressure and thus improvement in the vegetation ^51^. Therefore, our finding that a decrease in the vegetation density was observed only in very limited areas is consistent with these previous findings. Furthermore, the extension of green zones is part of the urban development master plans, particularly in Jakarta ^52^; therefore, the results of urban greening were visible on the NDVI in places like Central Jakarta, which has a thriving financial industry. However, this might not be easy to implement in other cities where the population is now growing quickly.

Several tropical countries have experienced vegetation loss, and they need to find policies to reverse the trend. The evaluation of agriculture has been paradoxical. The expansion of monoculture farmlands could be detrimental to the biodiversity. Agriculture also contributes to carbon dioxide emission ^24^, but biomass is being grown particularly in oil palm plantations with high vertical structures ^53^. In reality, the regency with the highest NDVI consistent trend was known for having one of the largest oil palm plantations because the growth of seedlings and young palms “trade-off” the harvest of old palms. In addition, the *alang-alang* (*Imperata cylindrica*) grassland that covers the land soon after deforestation or agricultural land abandonment has now been identified as a barrier to natural ecological succession ^54^. Efforts are currently being made to transform this grassland into productive land (e.g., Karo Regency) ^55^.

However, the growth of Indonesia’s vegetation density as a result of policies and people's actions, despite the country’s growing population and economy, is some positive news for global warming mitigation.

## Conclusions

Based on analysis of time-series satellite data at high temporal resolution (every 16-day period), this study found that vegetation density represented by the NDVI has consistently increased in 86.1% of regencies/cities during the past 20 years, and the NDVIs in 2020 were higher than those in 2001 in 90.6% of regencies/cities. The growth of vegetation density in Indonesia was multifactorial: forest moratoriums, reforestation, plantation growth, and urban greening. We observed that the loss of the NDVI was centered in newly developed urban areas. Disasters did not affect the long-term trend. Although further studies are necessary to confirm factors influencing the NDVI changes, this study suggests that vegetation density is increasing in Indonesia as a result of policies and people’s behavior.

## Supplementary Information

Supplementary Table S1.

Supplementary Figure S1.

Supplementary Figure S2.

Supplementary Figure S3.

Supplementary Figure S4.

Supplementary Table S2.

Supplementary Figure S5.

Supplementary Table S3.

Supplementary Figure S6.

## Data Availability

Raw data of the MODIS, CHIRPS, shapefiles, and socioeconomic data are available at respective sources described above. All figure and map outputs from the SSM are included in Supplementary Information. The R scripts and all other data will be provided upon reasonable requests to the corresponding author.
