# Supplementary material for: Time-series analysis of satellite imagery for detecting vegetation cover changes in Indonesia
Source: Sci Rep. 2023 May 25;13:8437. doi: 10.1038/s41598-023-35330-1 (PMC10212945; doi:10.1038/s41598-023-35330-1)
Supplement: Supplementary file 2 — Supplementary Figure S1. [file 41598_2023_35330_MOESM2_ESM.pdf]

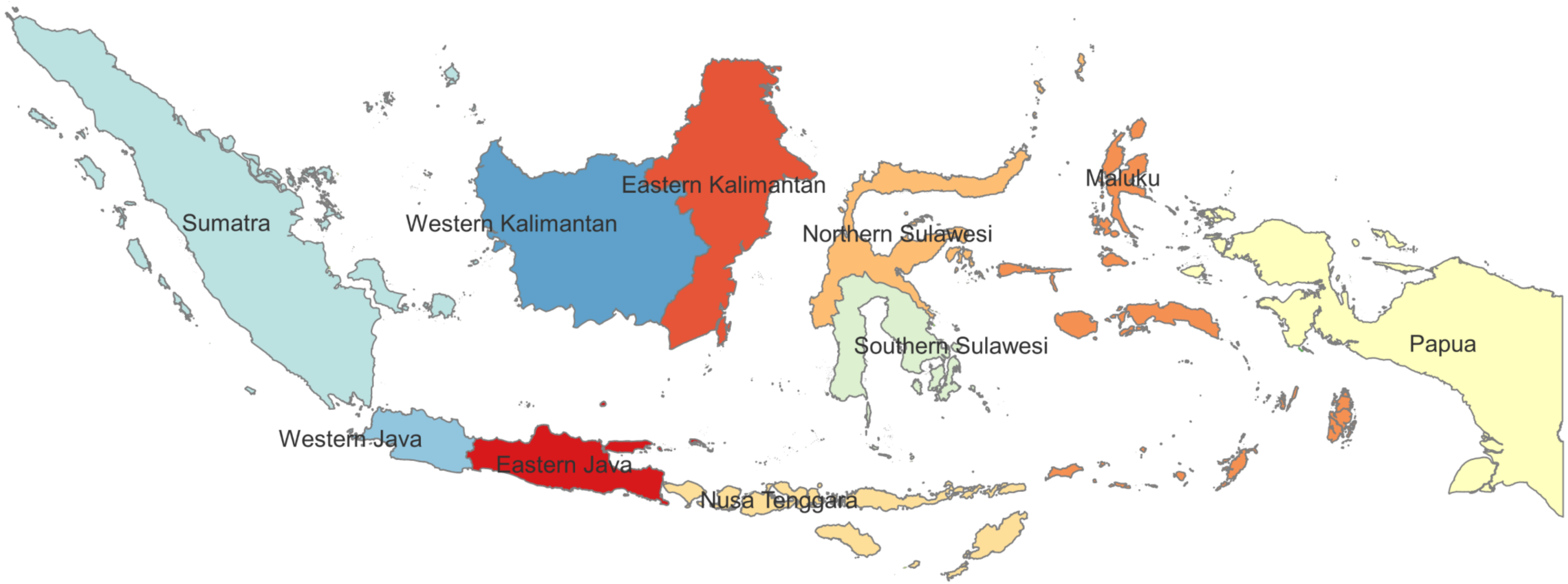

Figure S1. Zoning of Indonesian provinces for the classification and regression tree analysis. This map was created by TF using QGIS 3.22.4 Białowieża software and open administrative boundary data published by OCHA HDX
