# Supplementary material for: Time-series analysis of satellite imagery for detecting vegetation cover changes in Indonesia
Source: Sci Rep. 2023 May 25;13:8437. doi: 10.1038/s41598-023-35330-1 (PMC10212945; doi:10.1038/s41598-023-35330-1)
Supplement: Supplementary file 3 — Supplementary Figure S2. [file 41598_2023_35330_MOESM3_ESM.docx]

Figure S2. NDVI trend observed (gray line) and predicted (red line) in each regency/city of Indonesia.

## Aceh Province


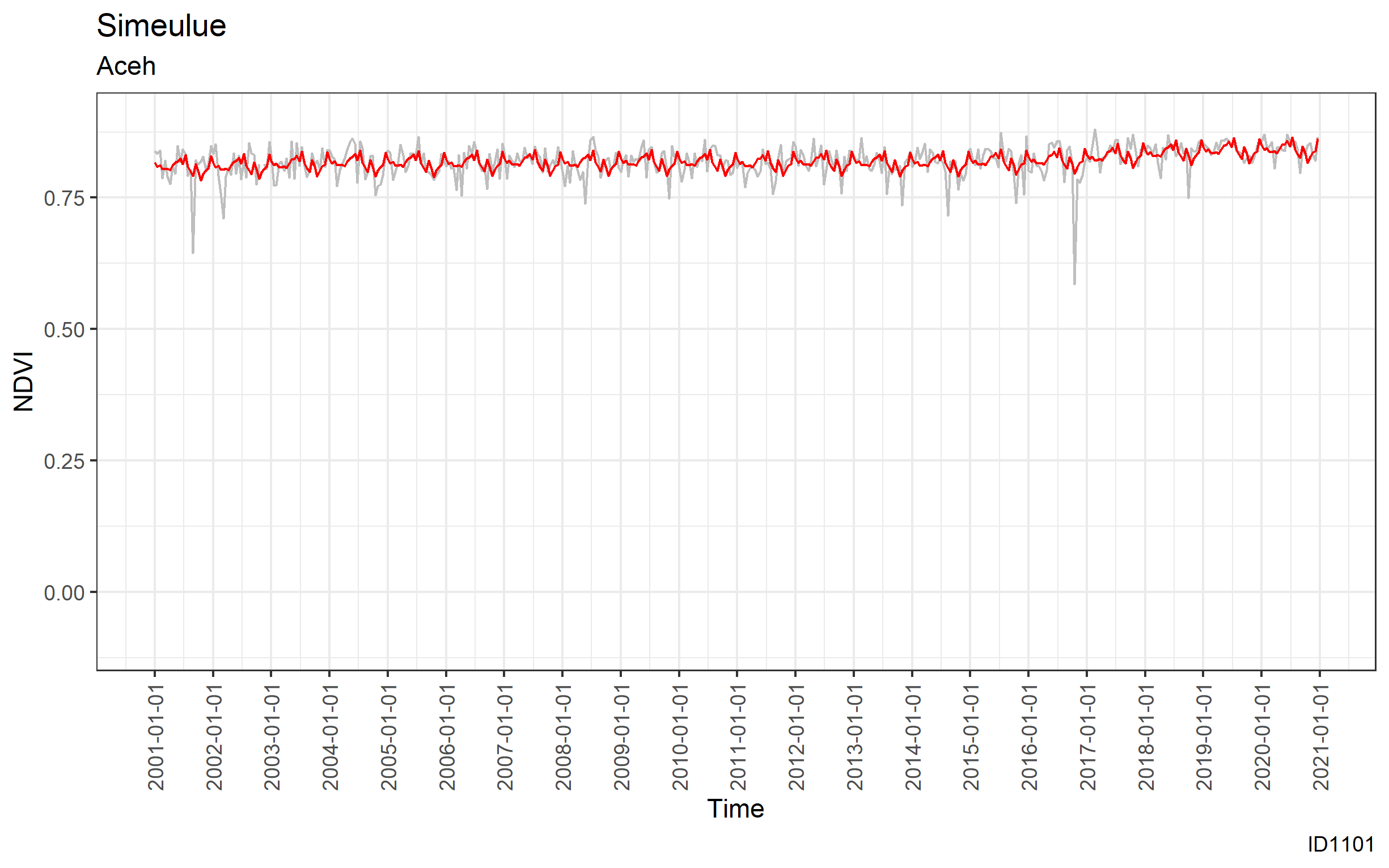

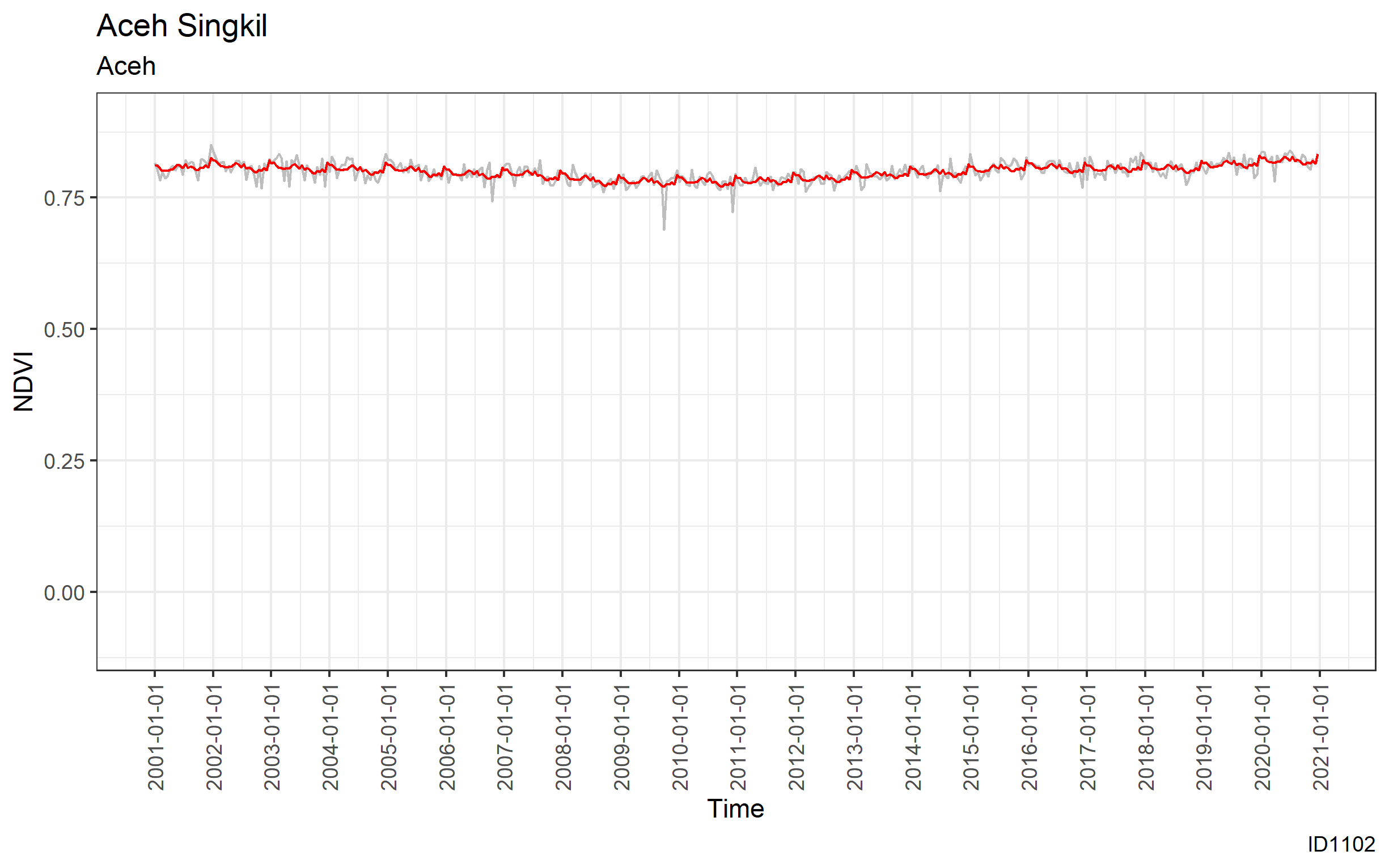

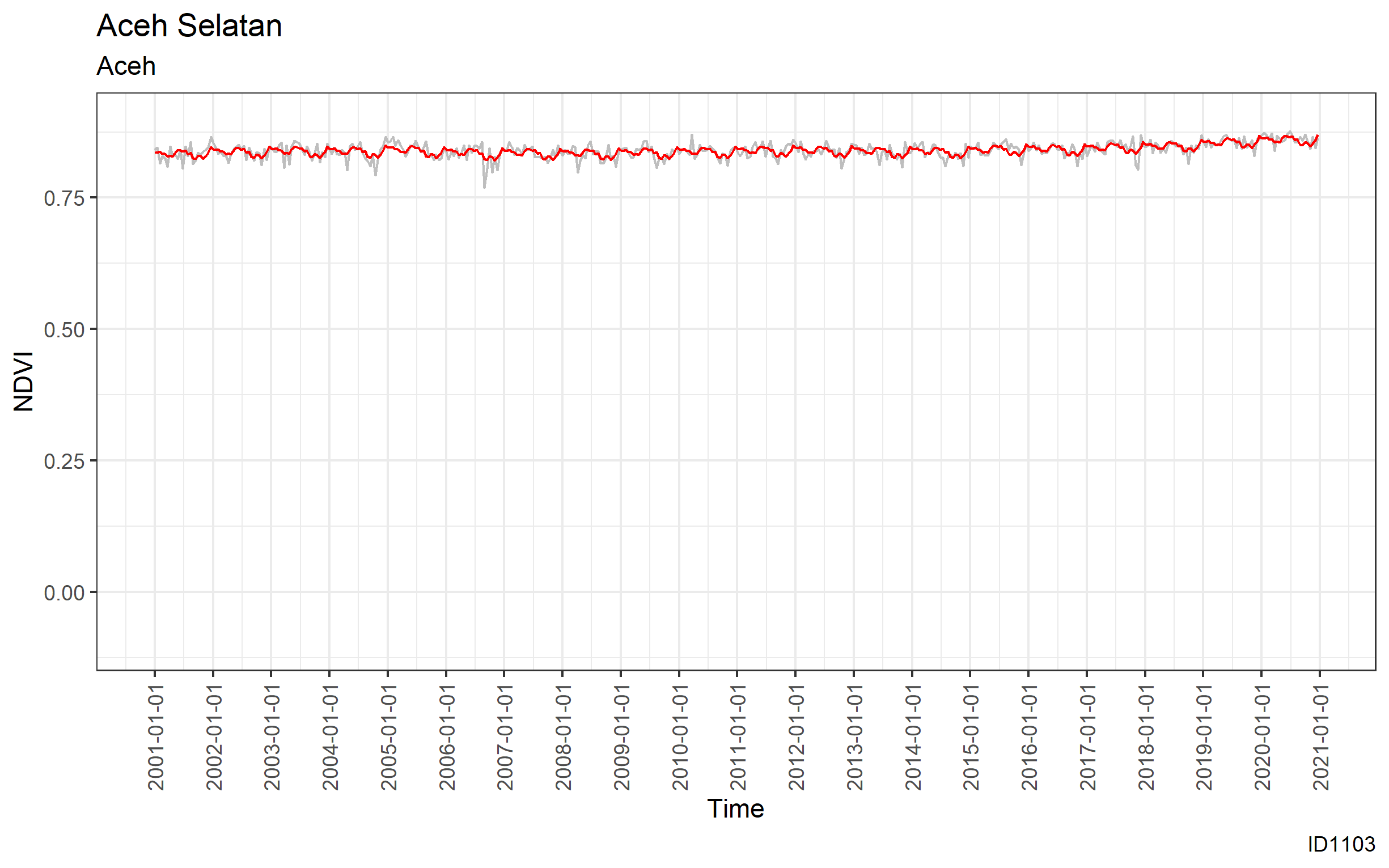

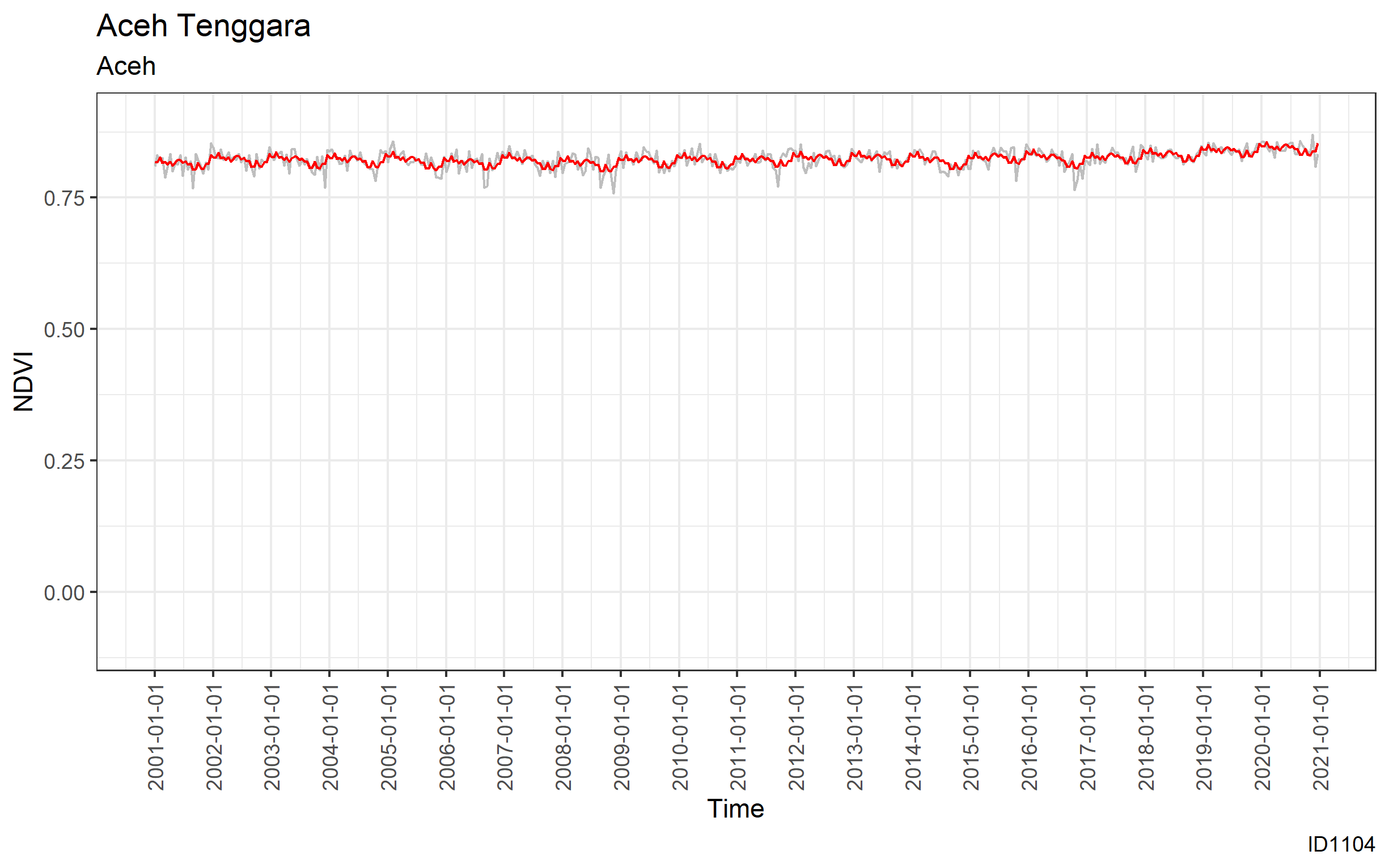


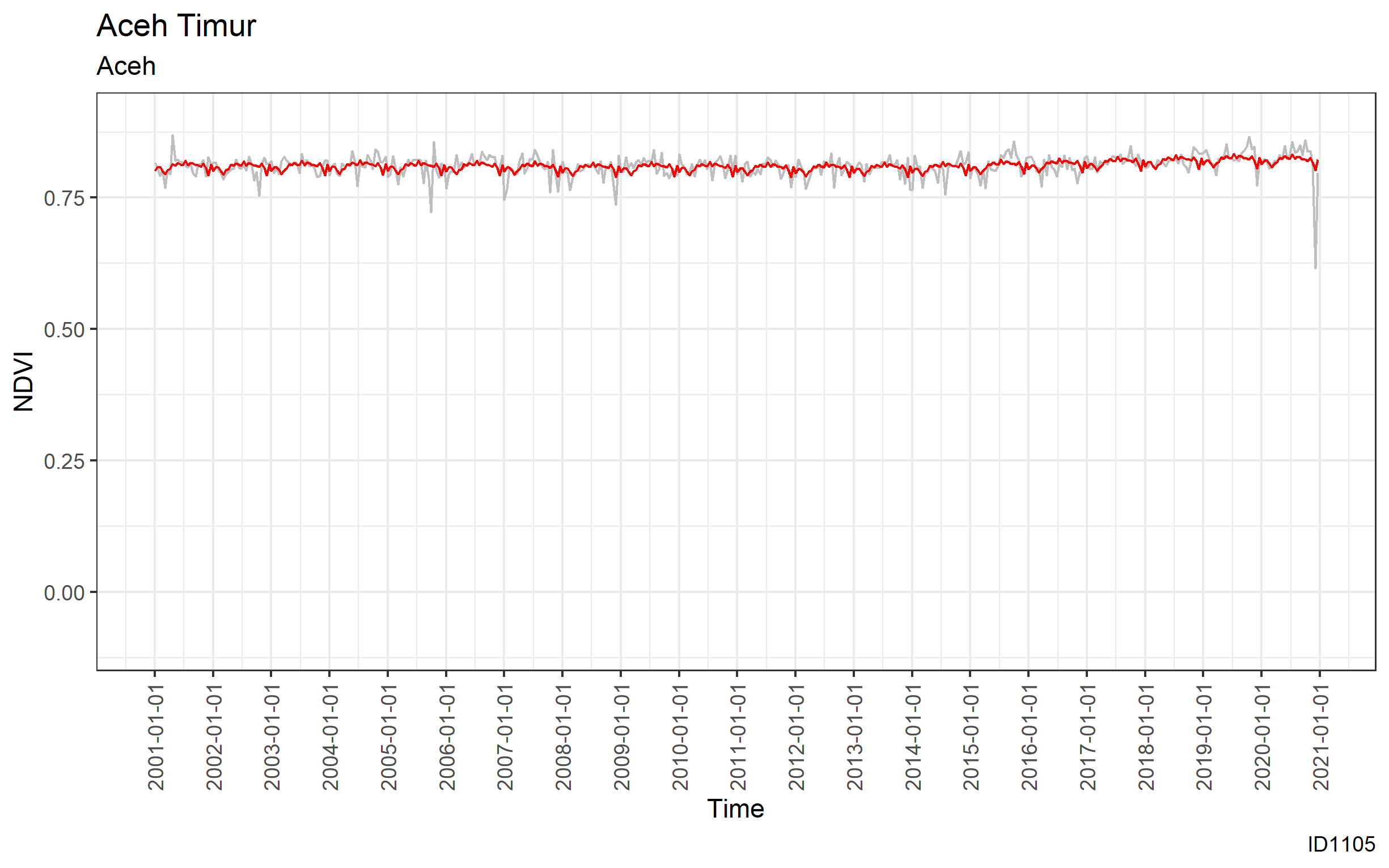

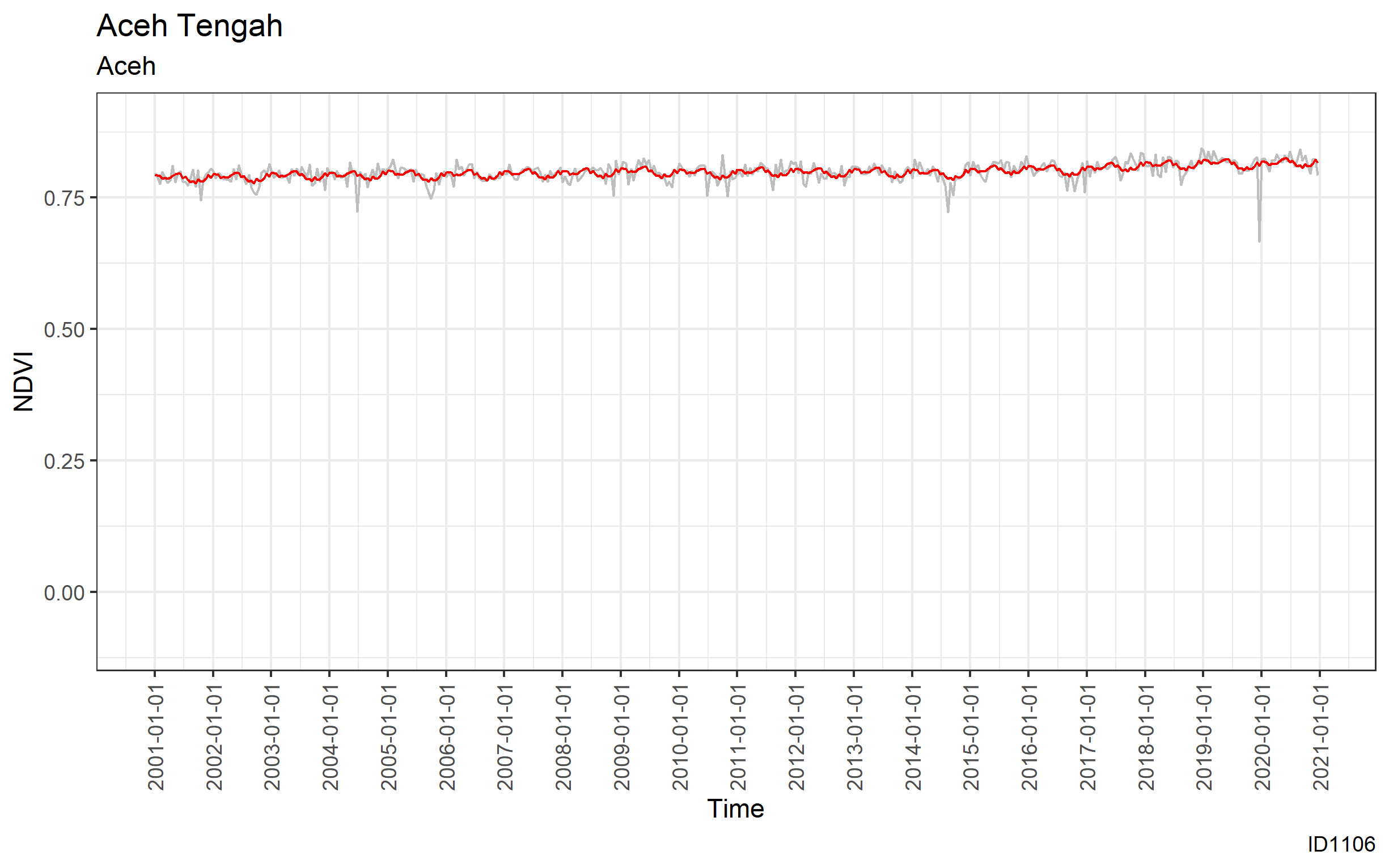

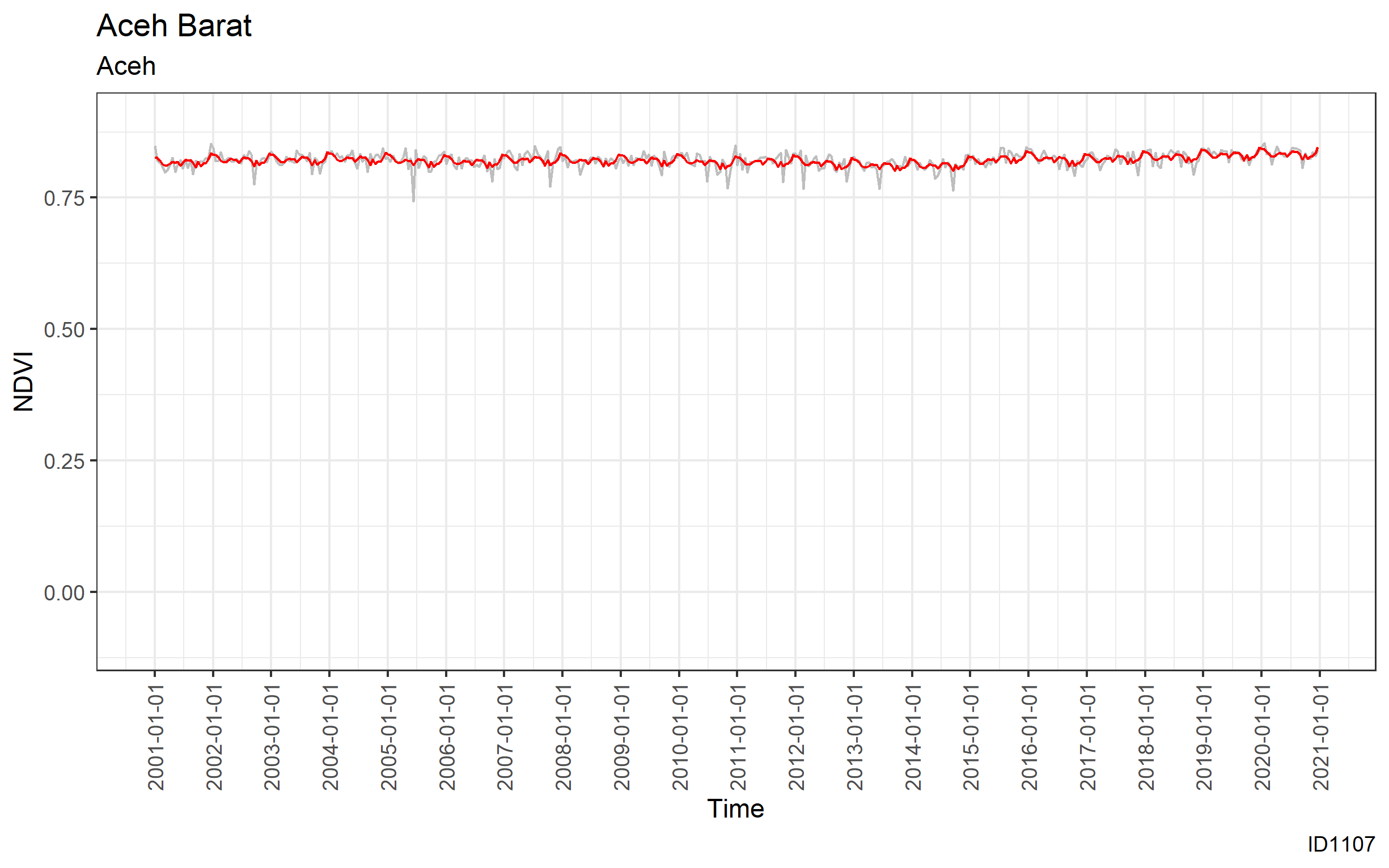

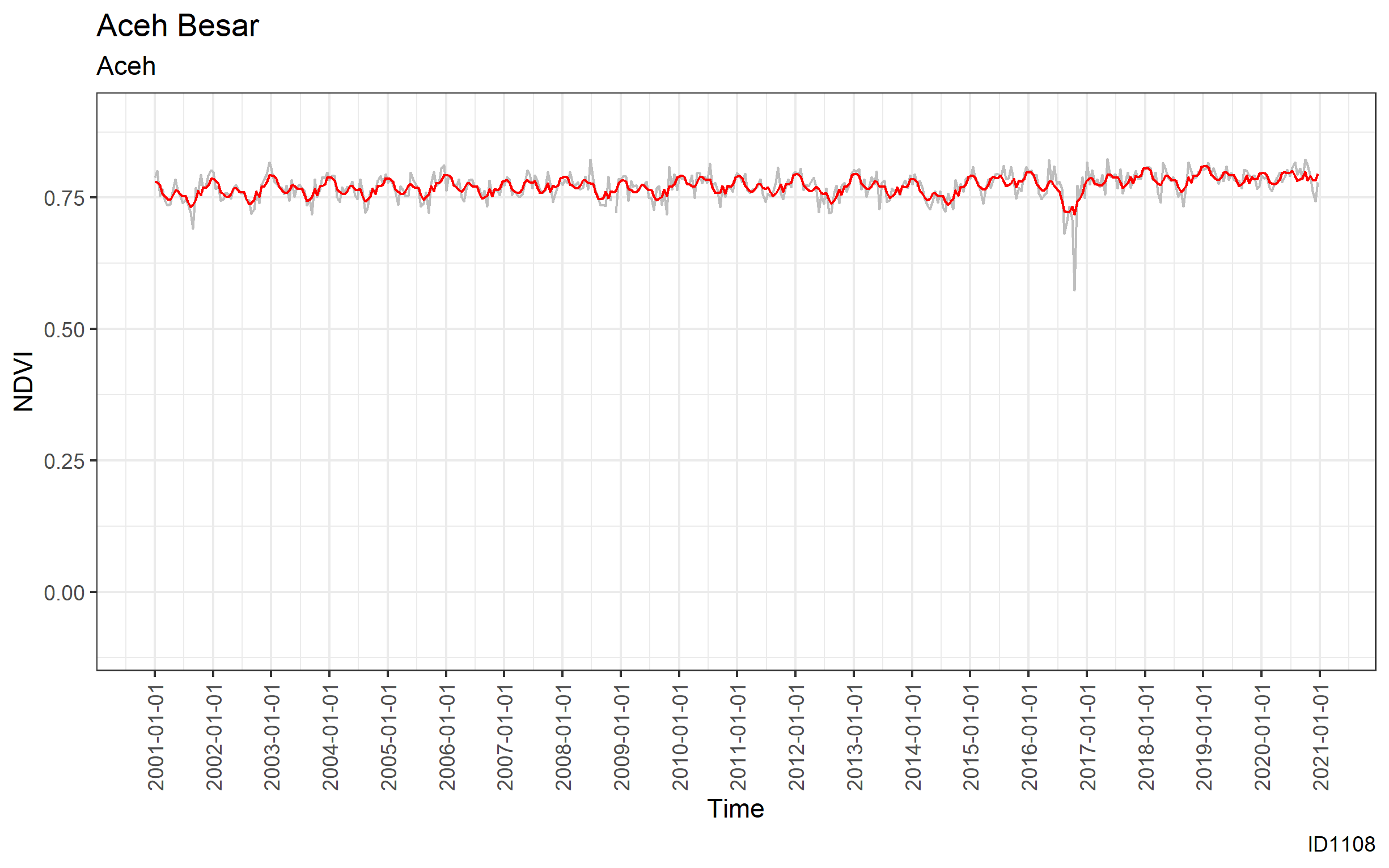

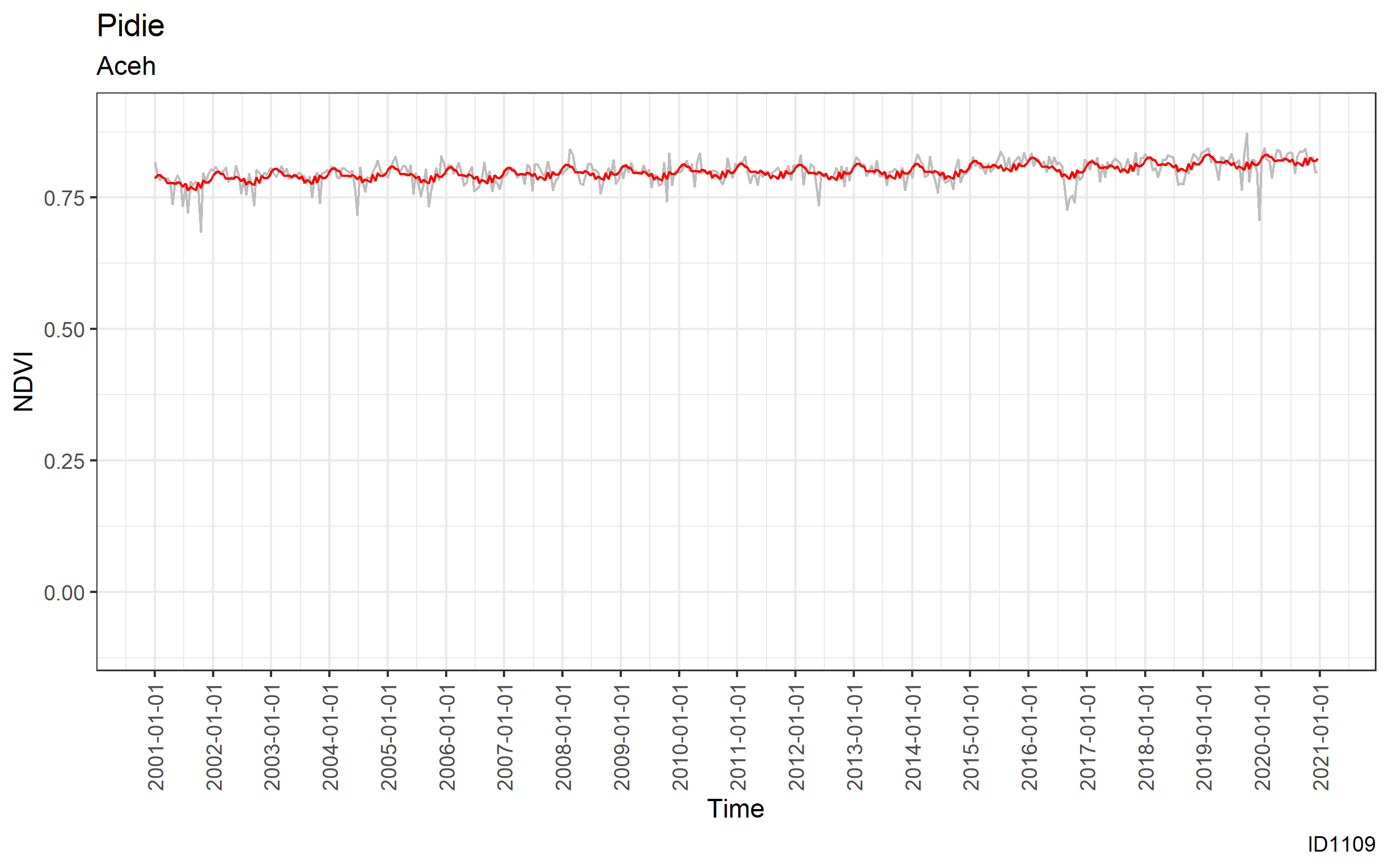

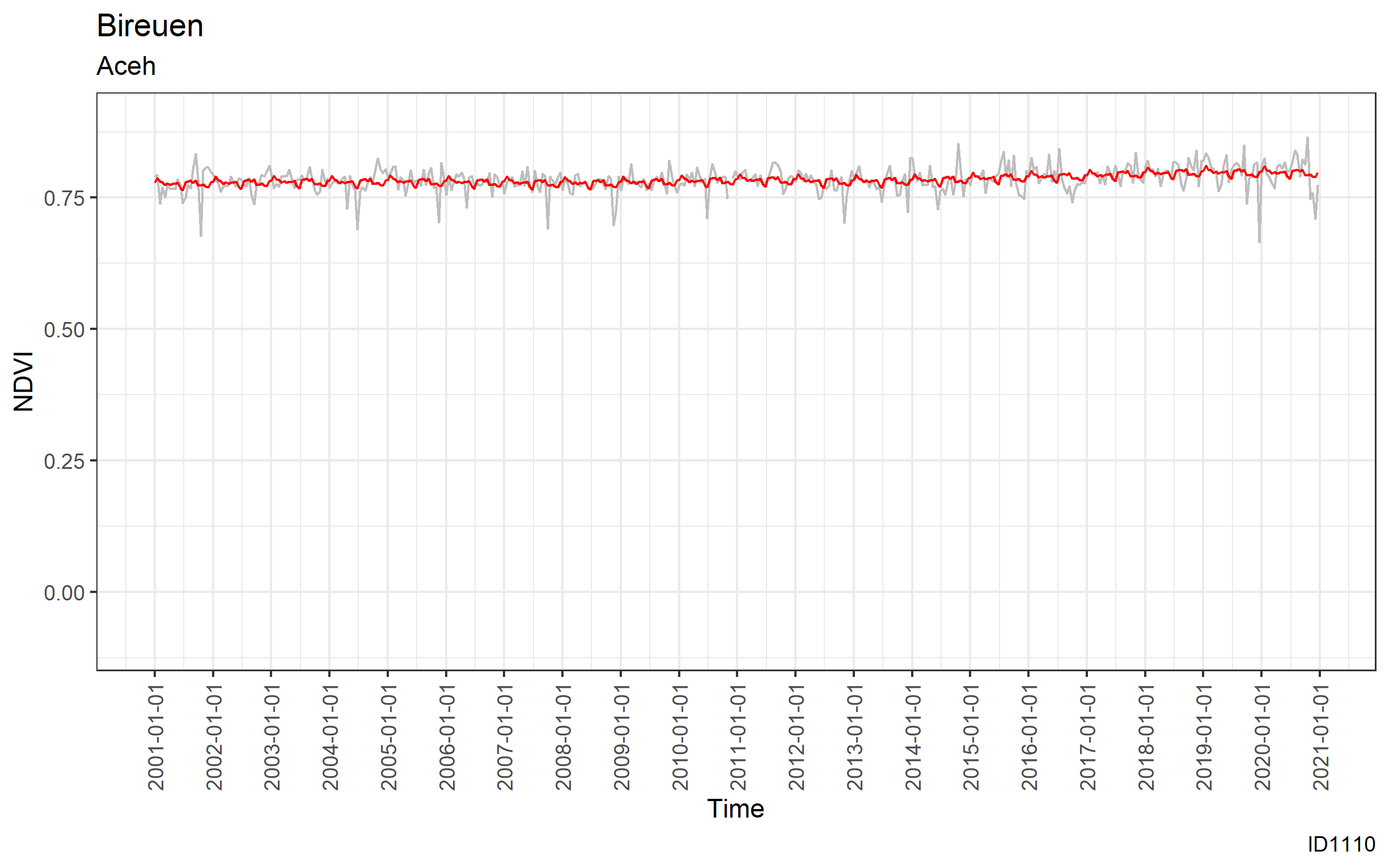

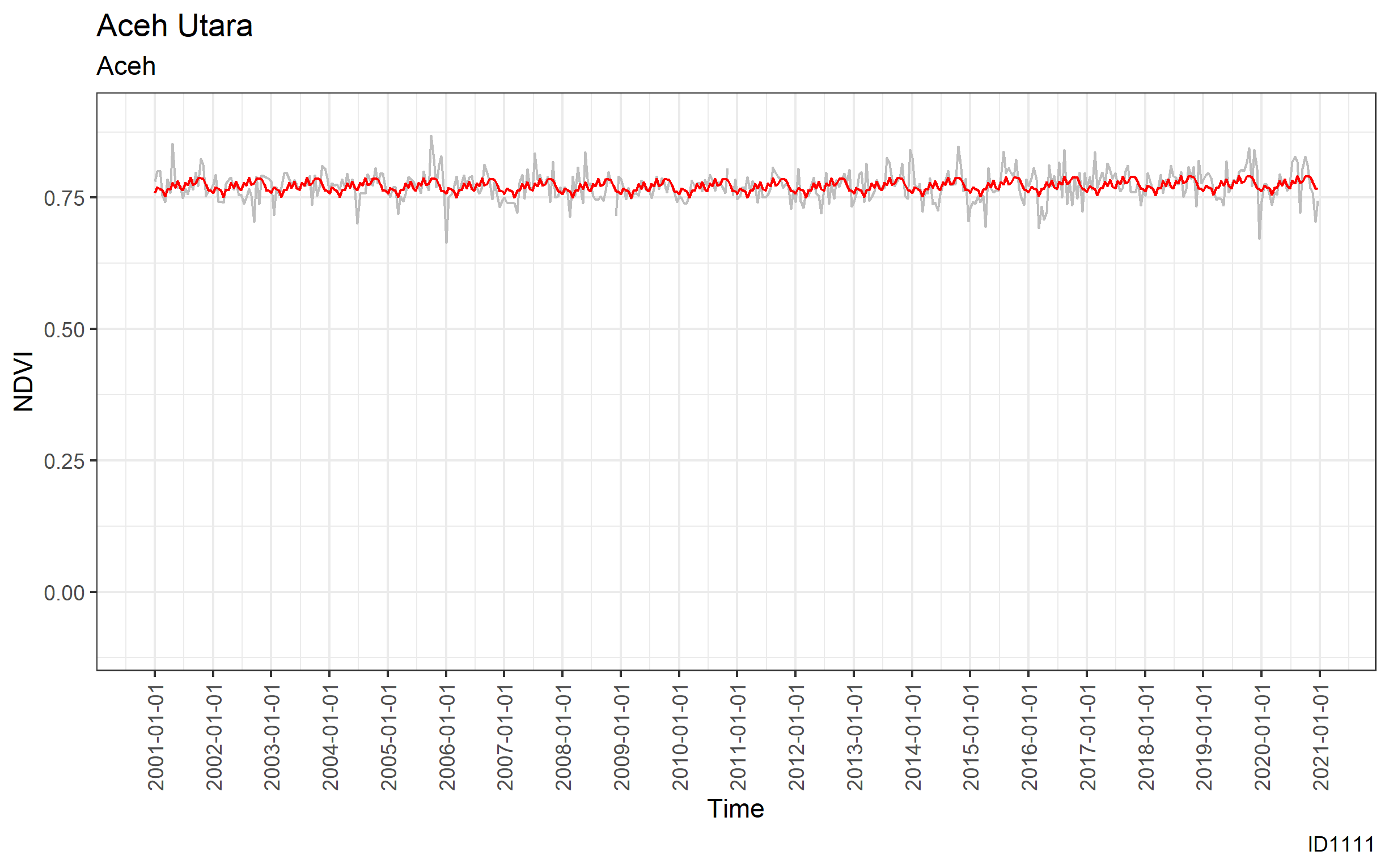

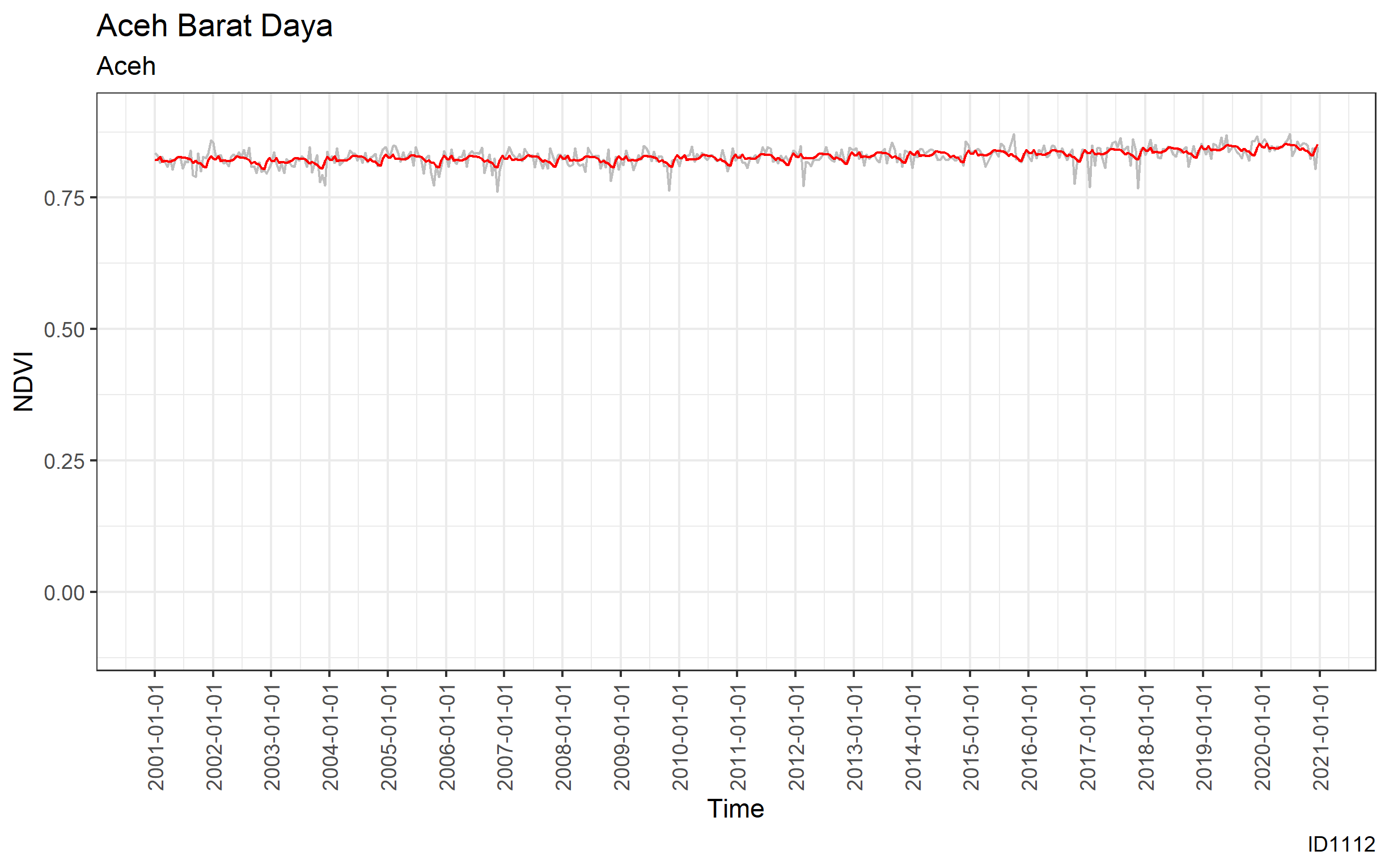

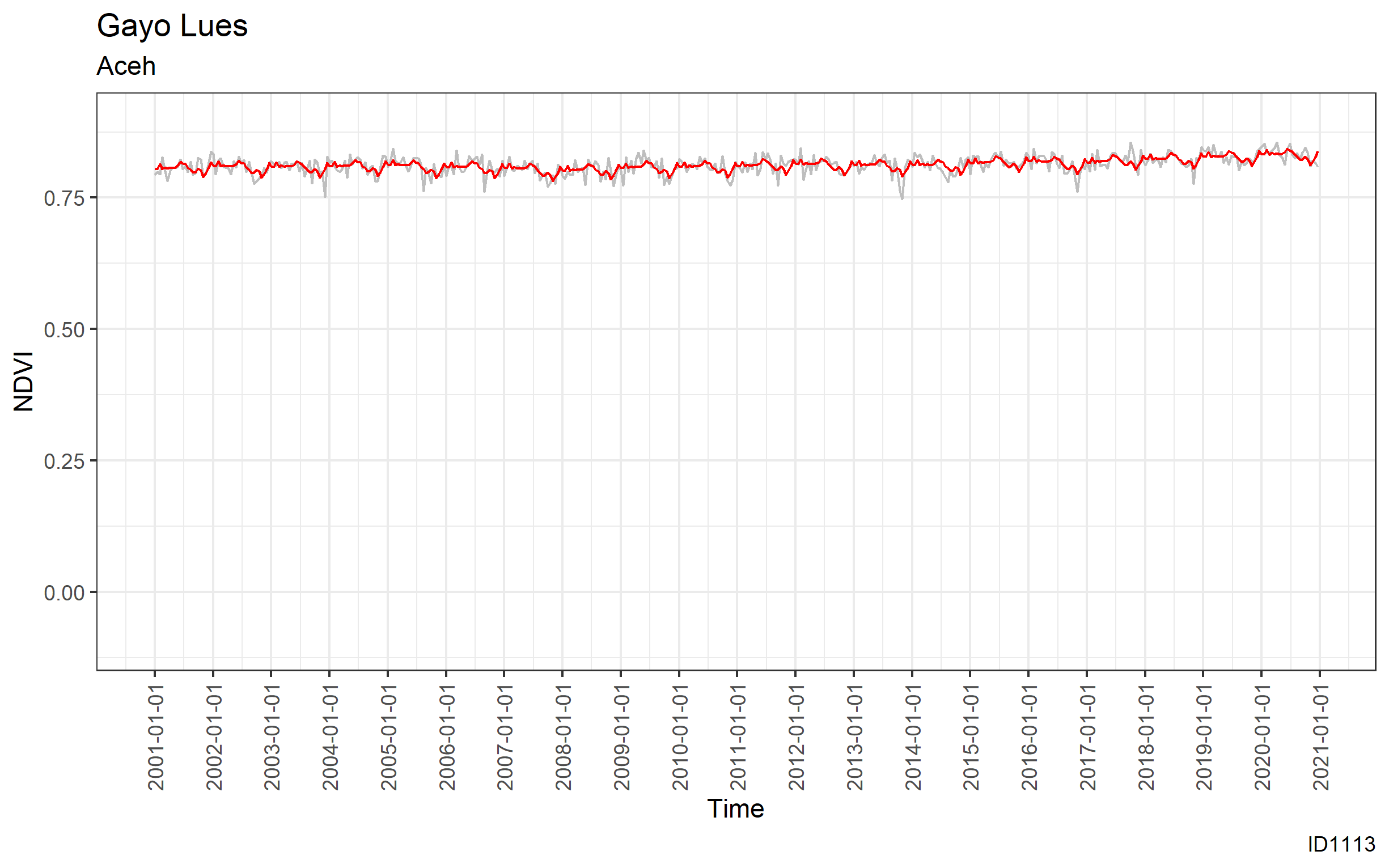

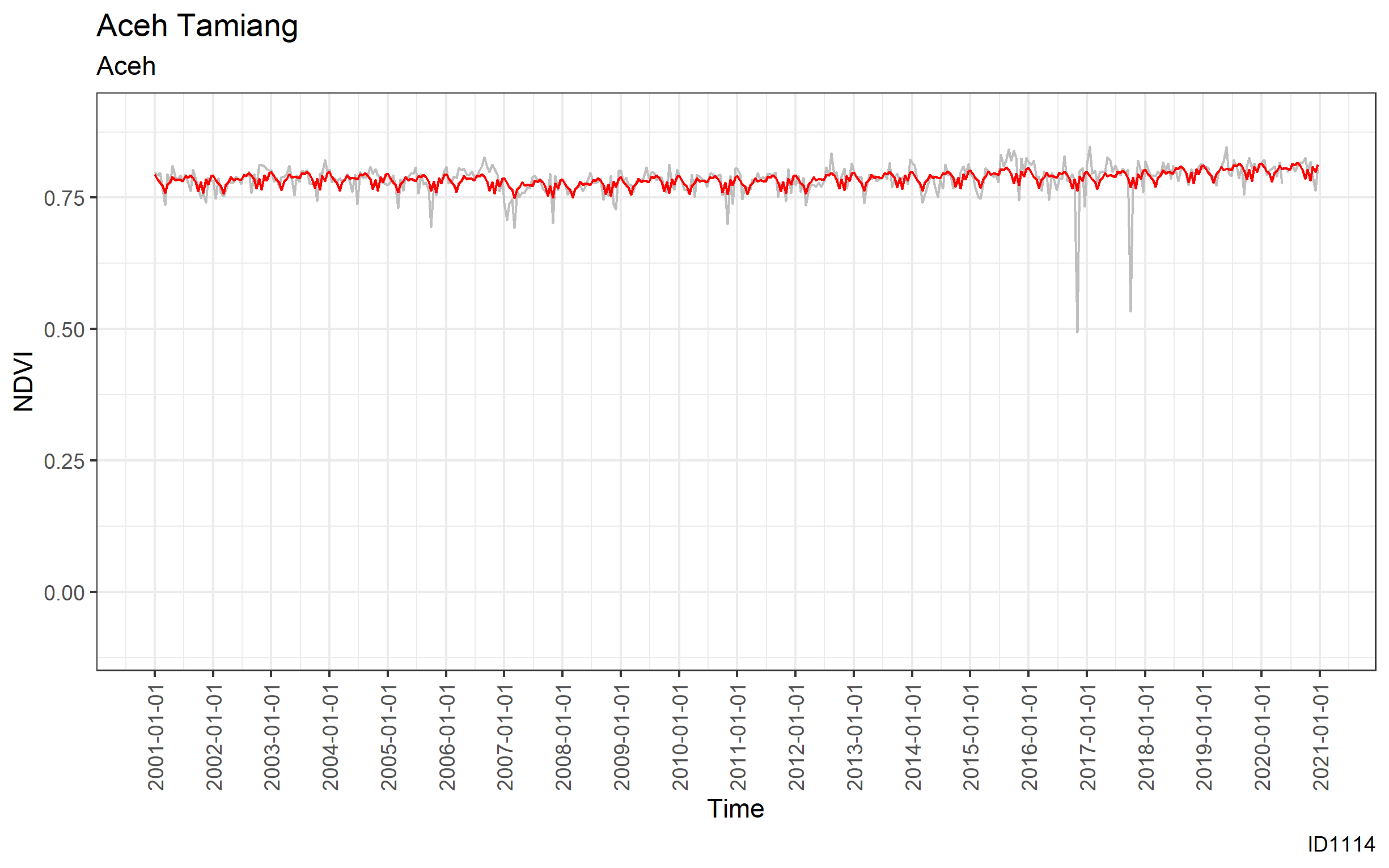

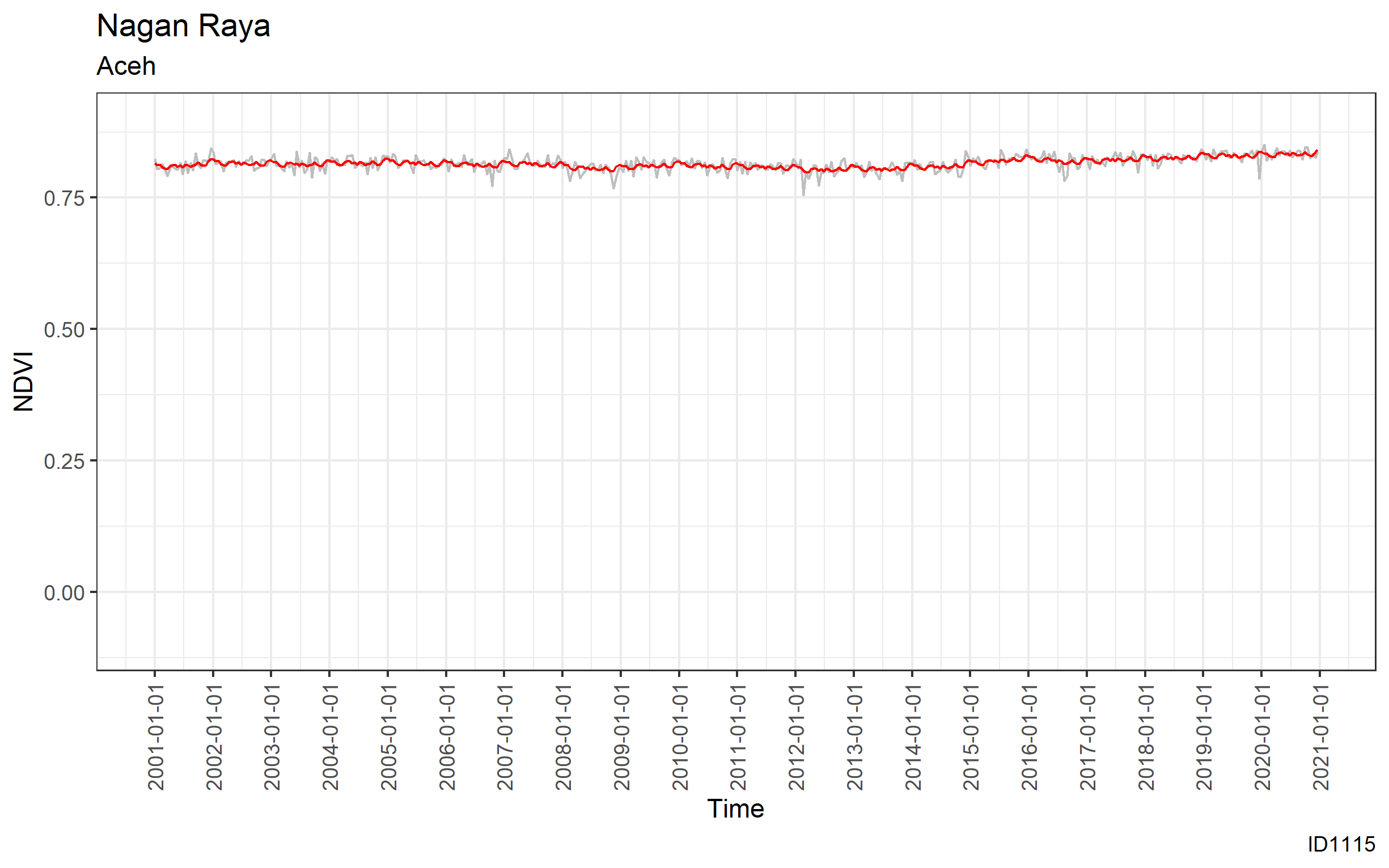

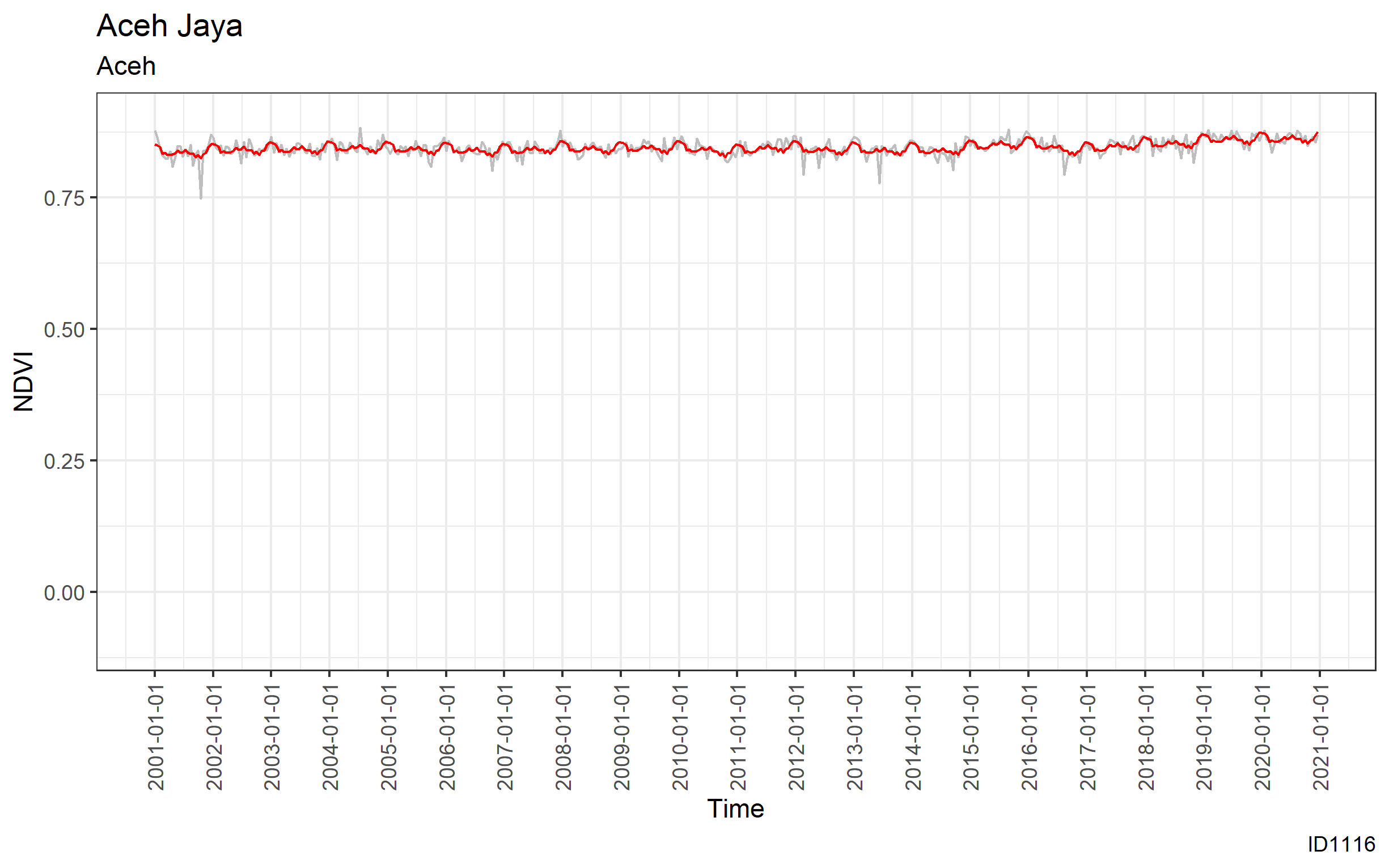

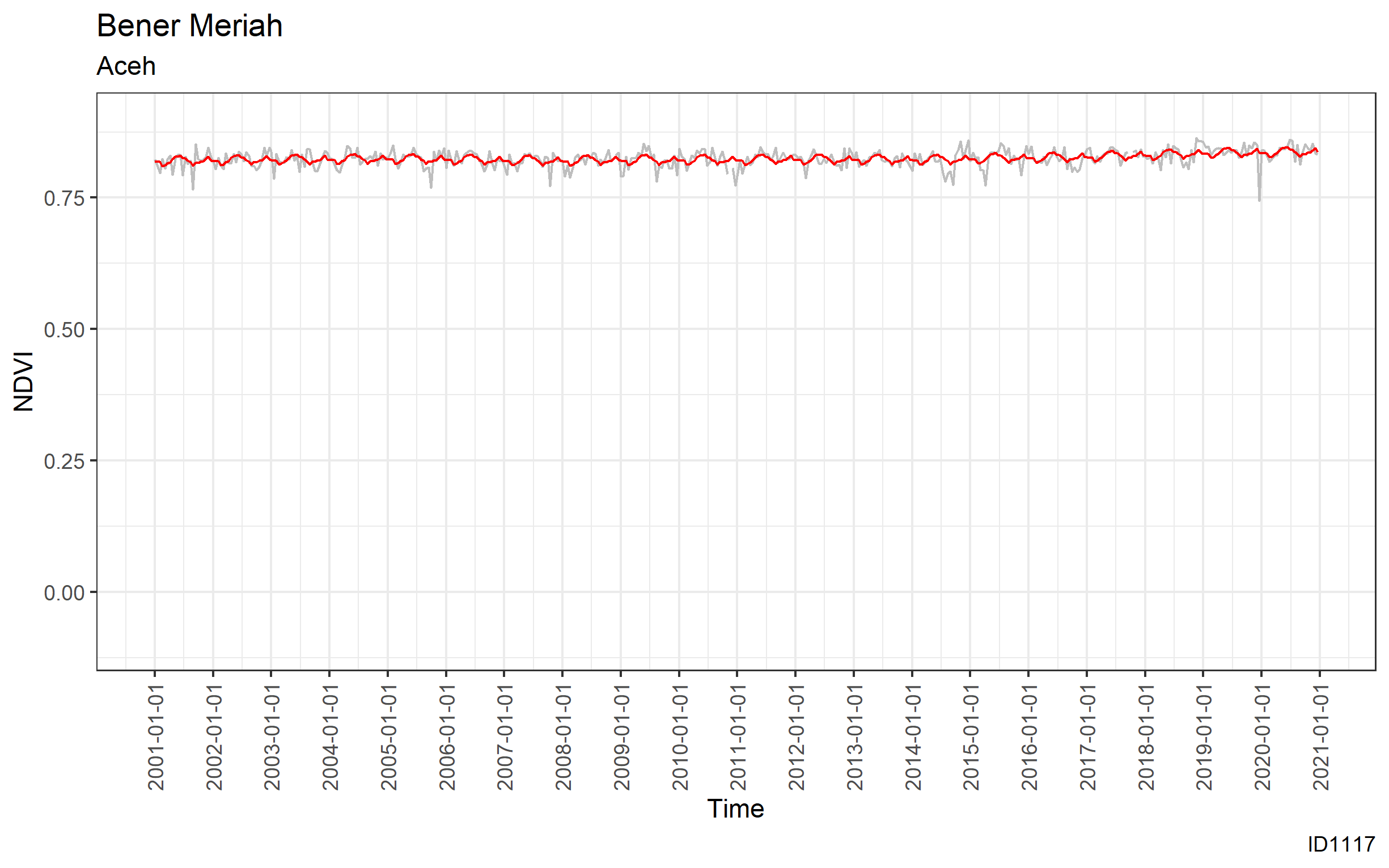

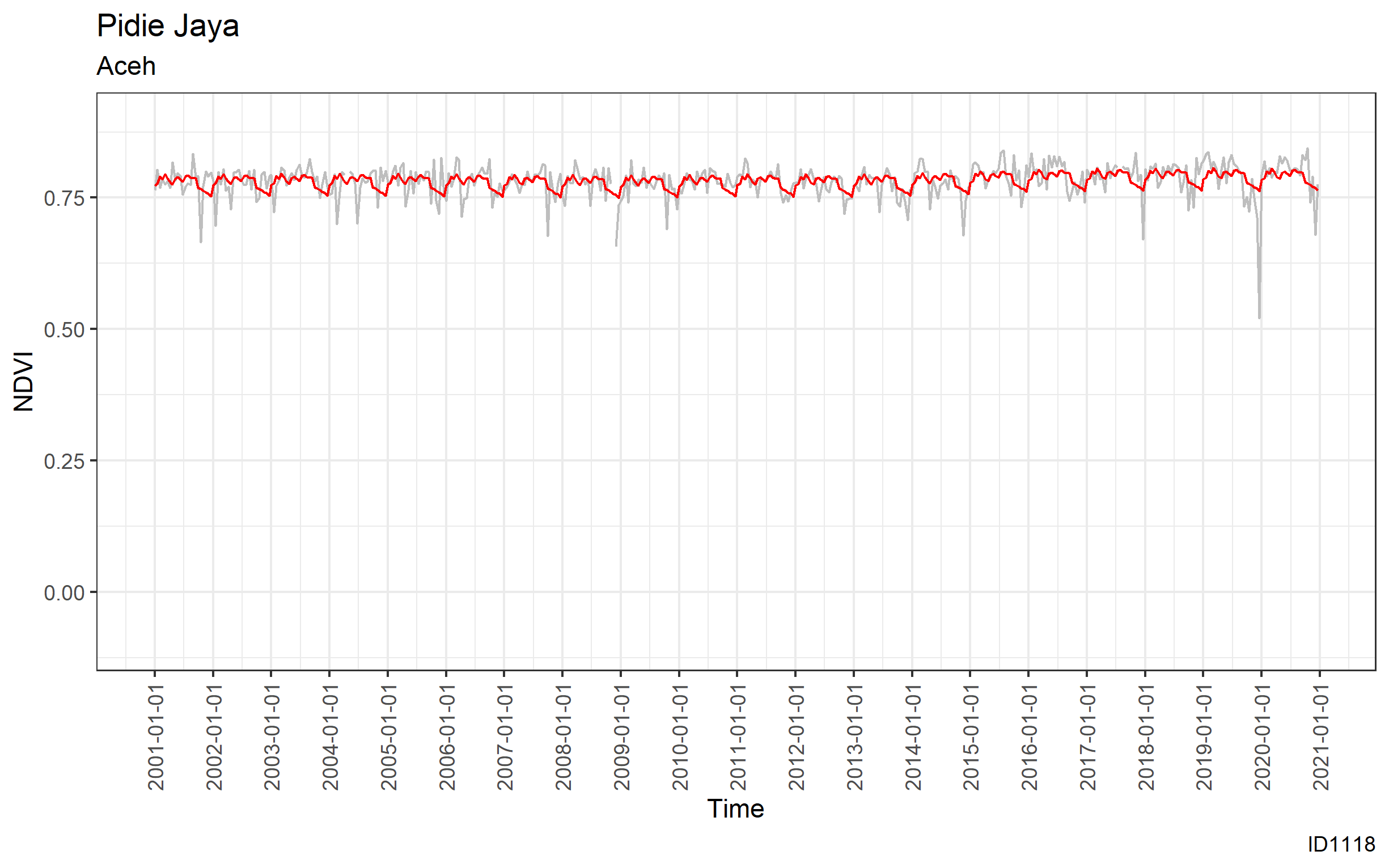

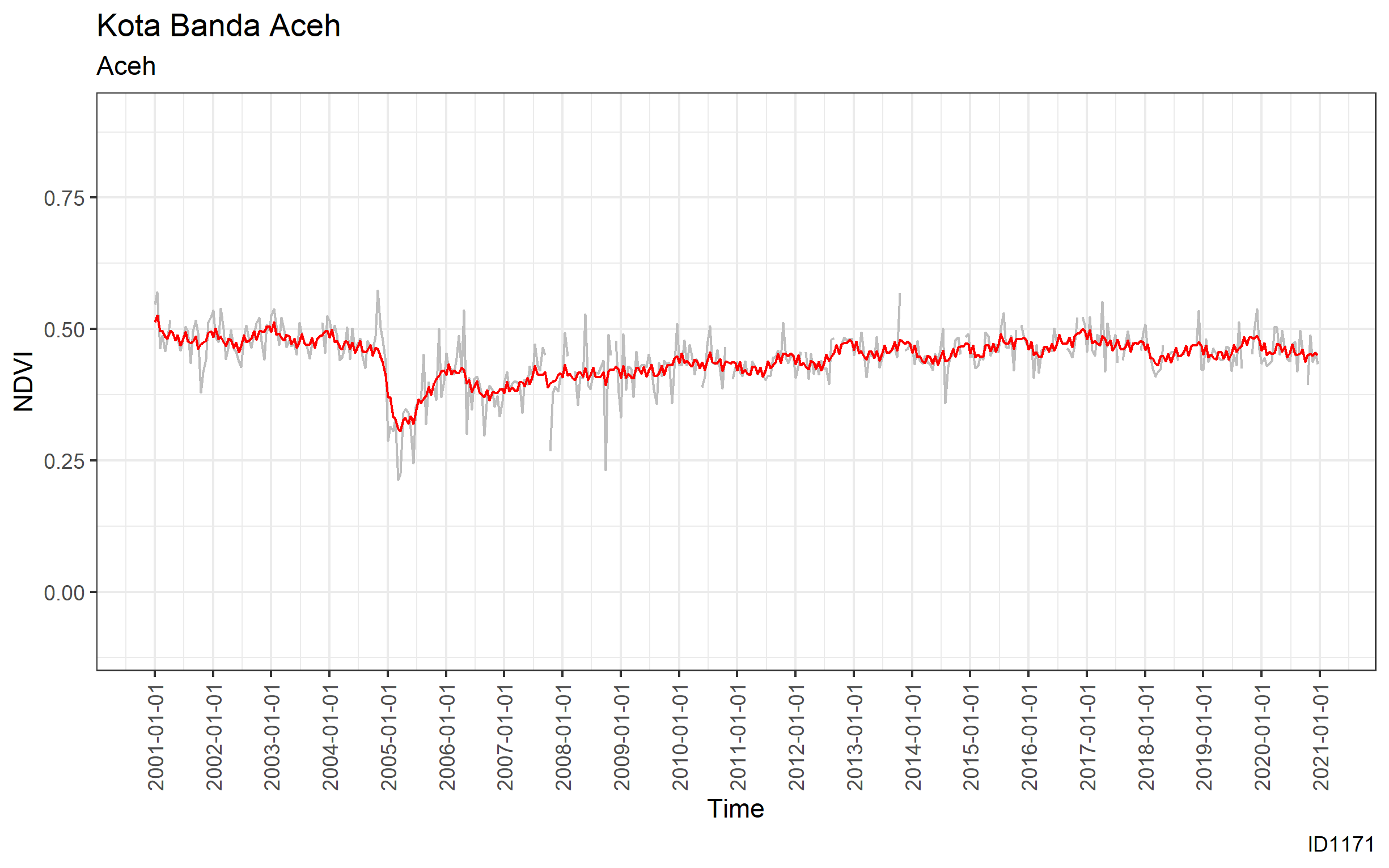

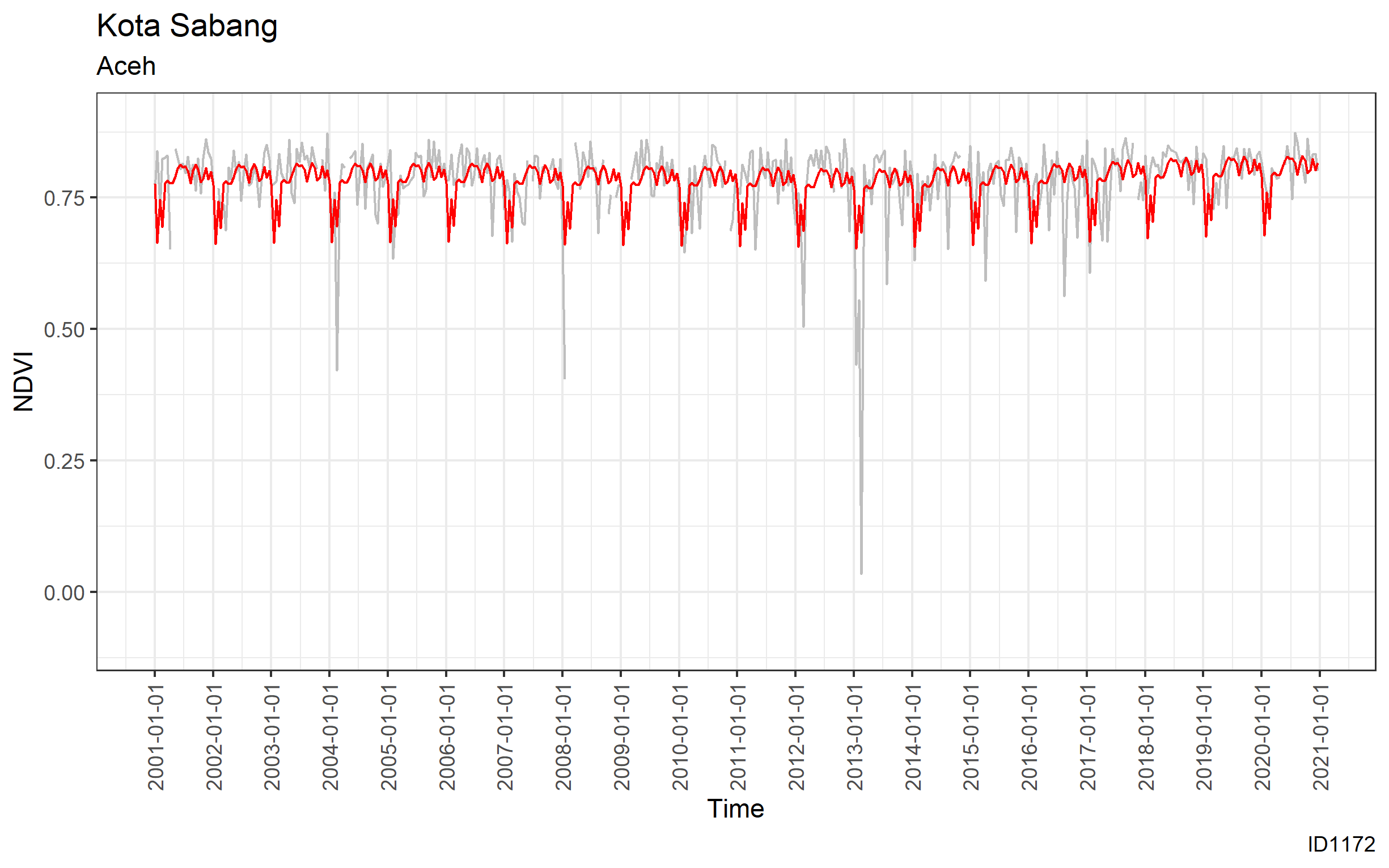

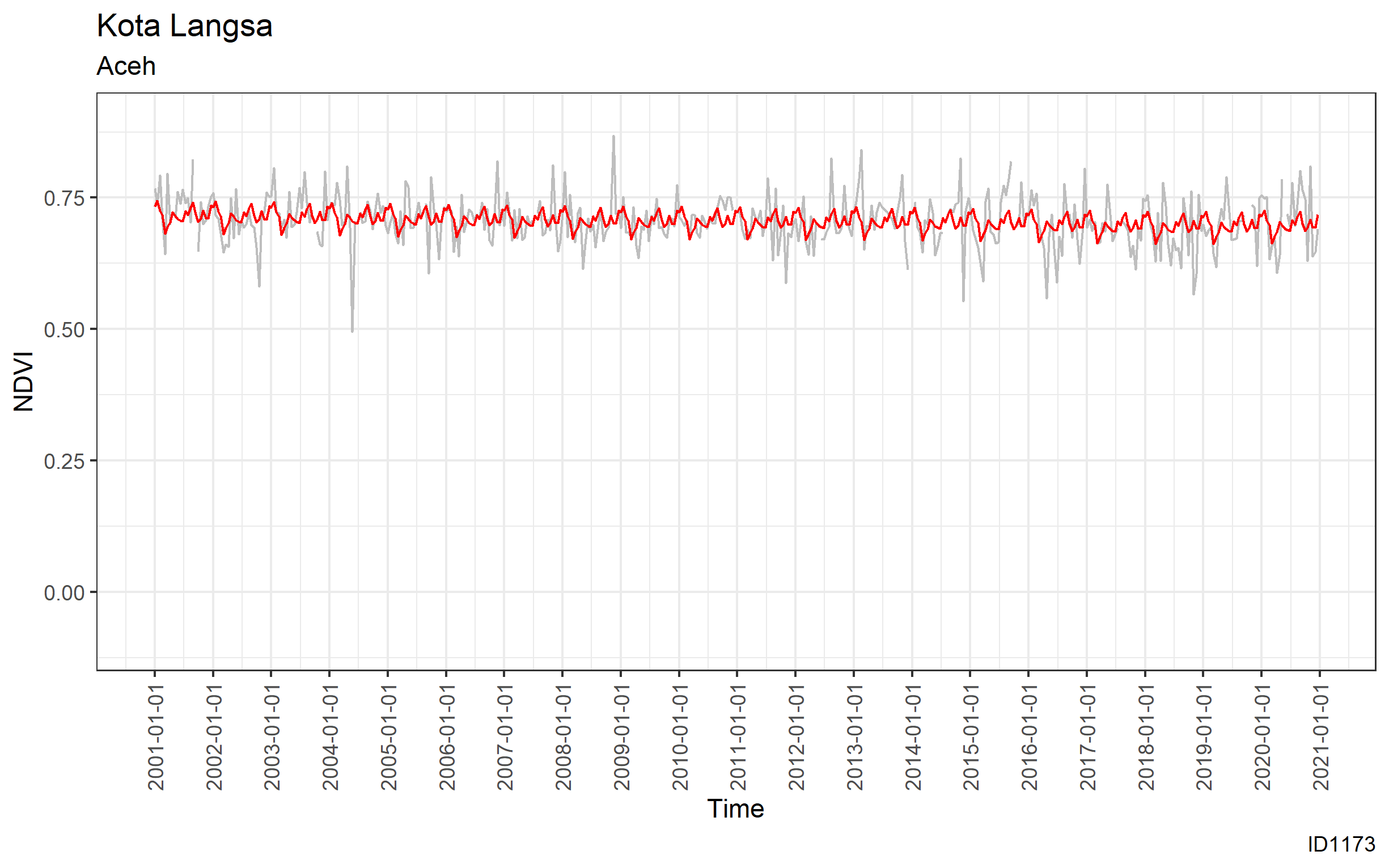

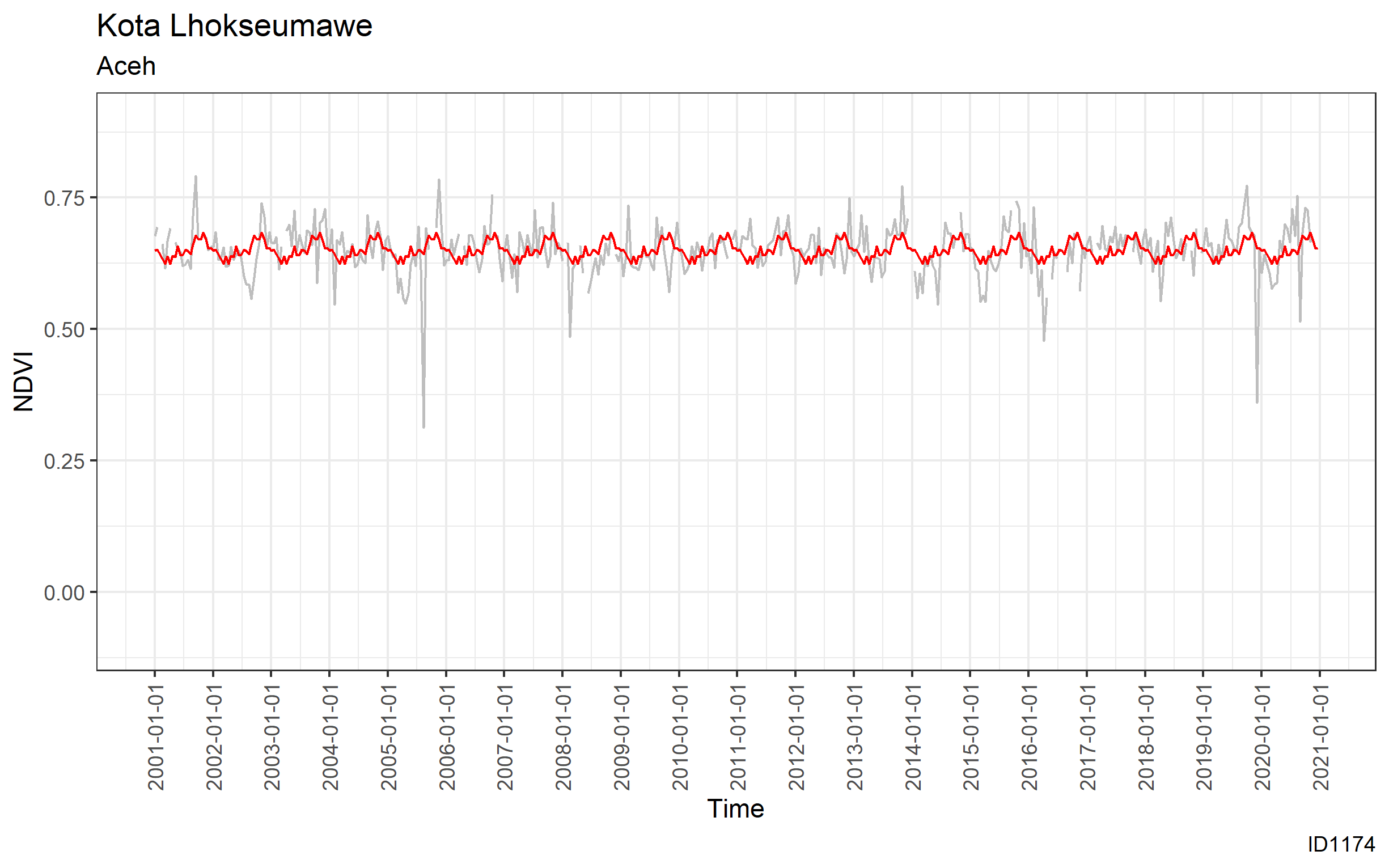

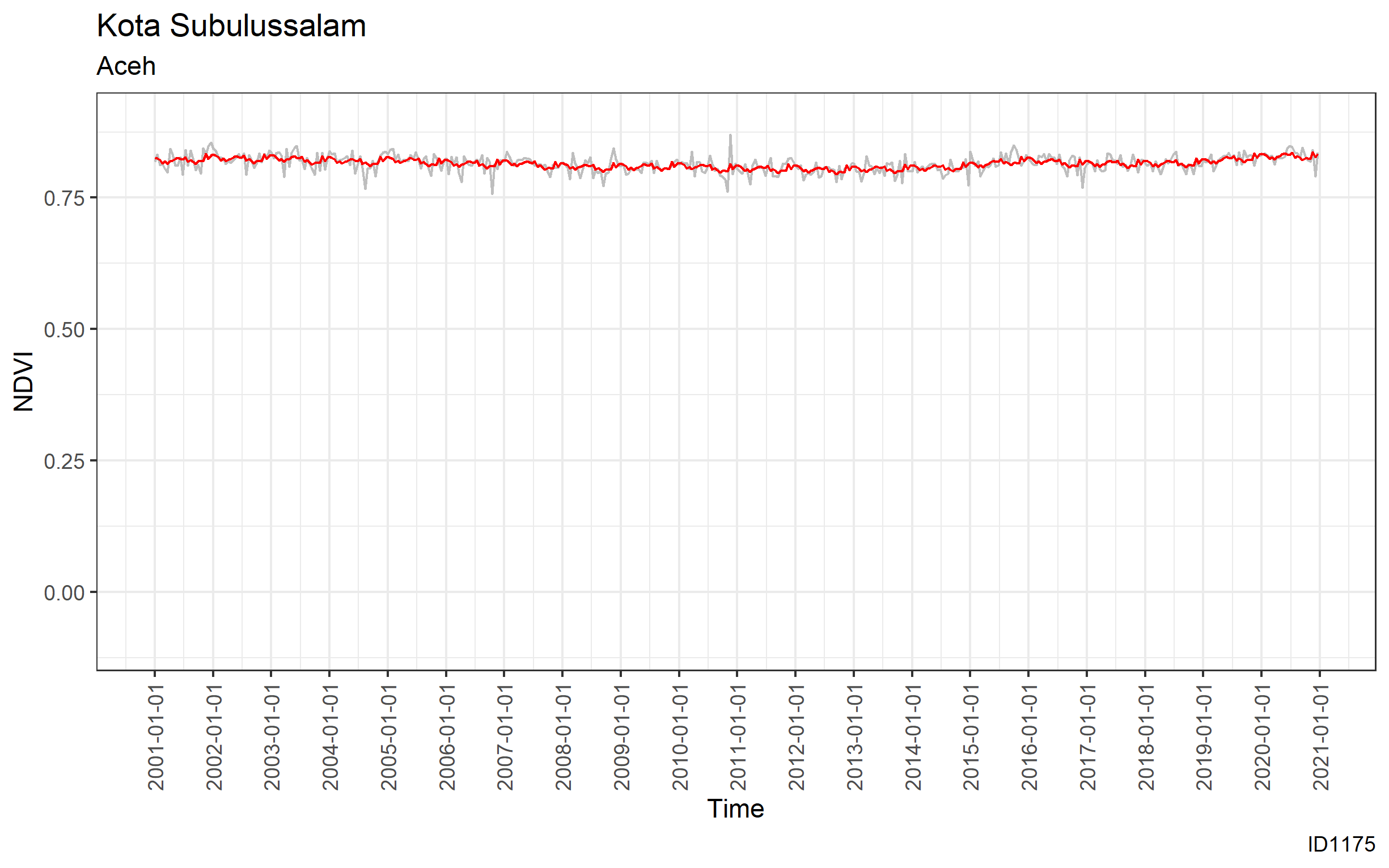


## North Sumatra Province


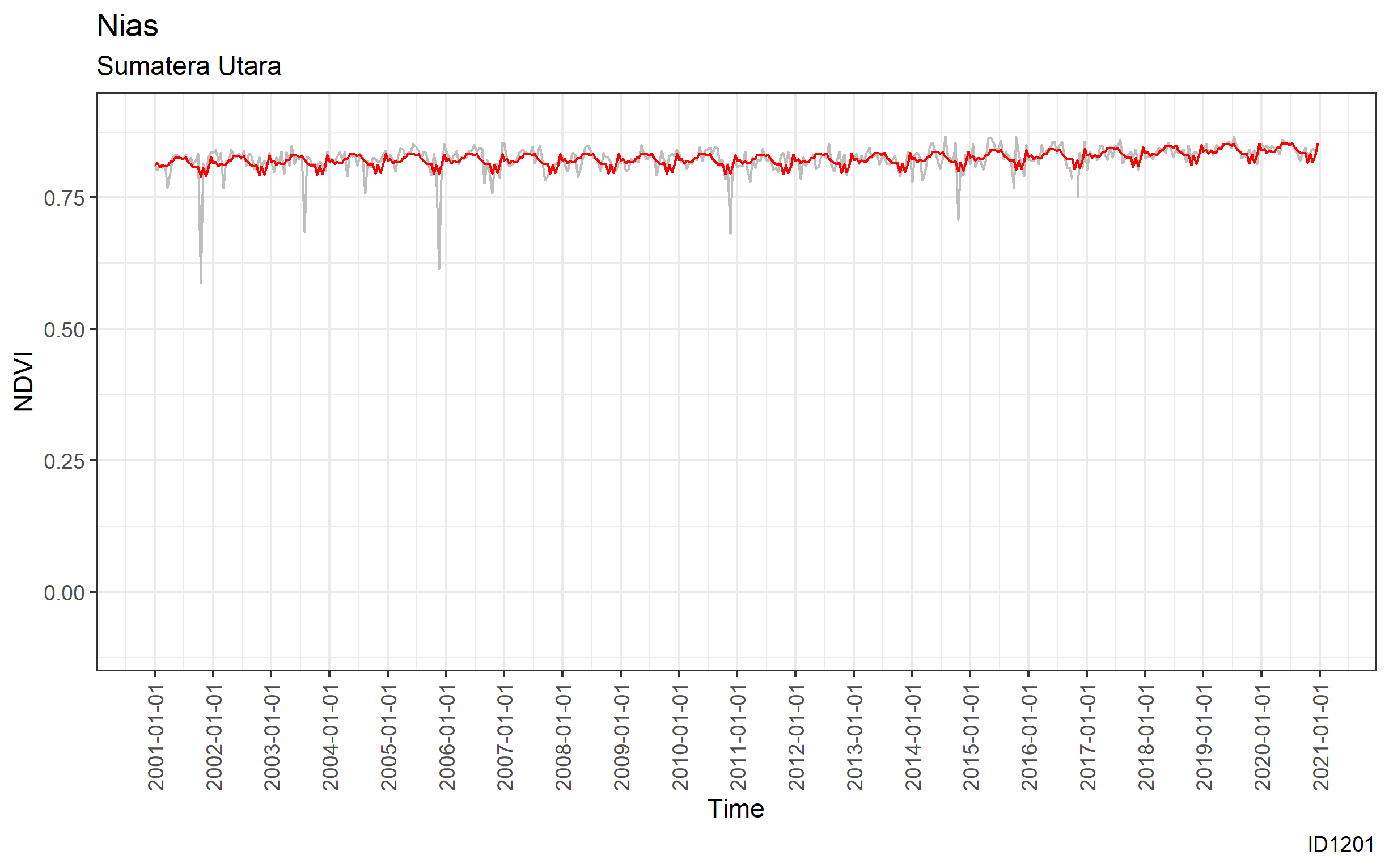

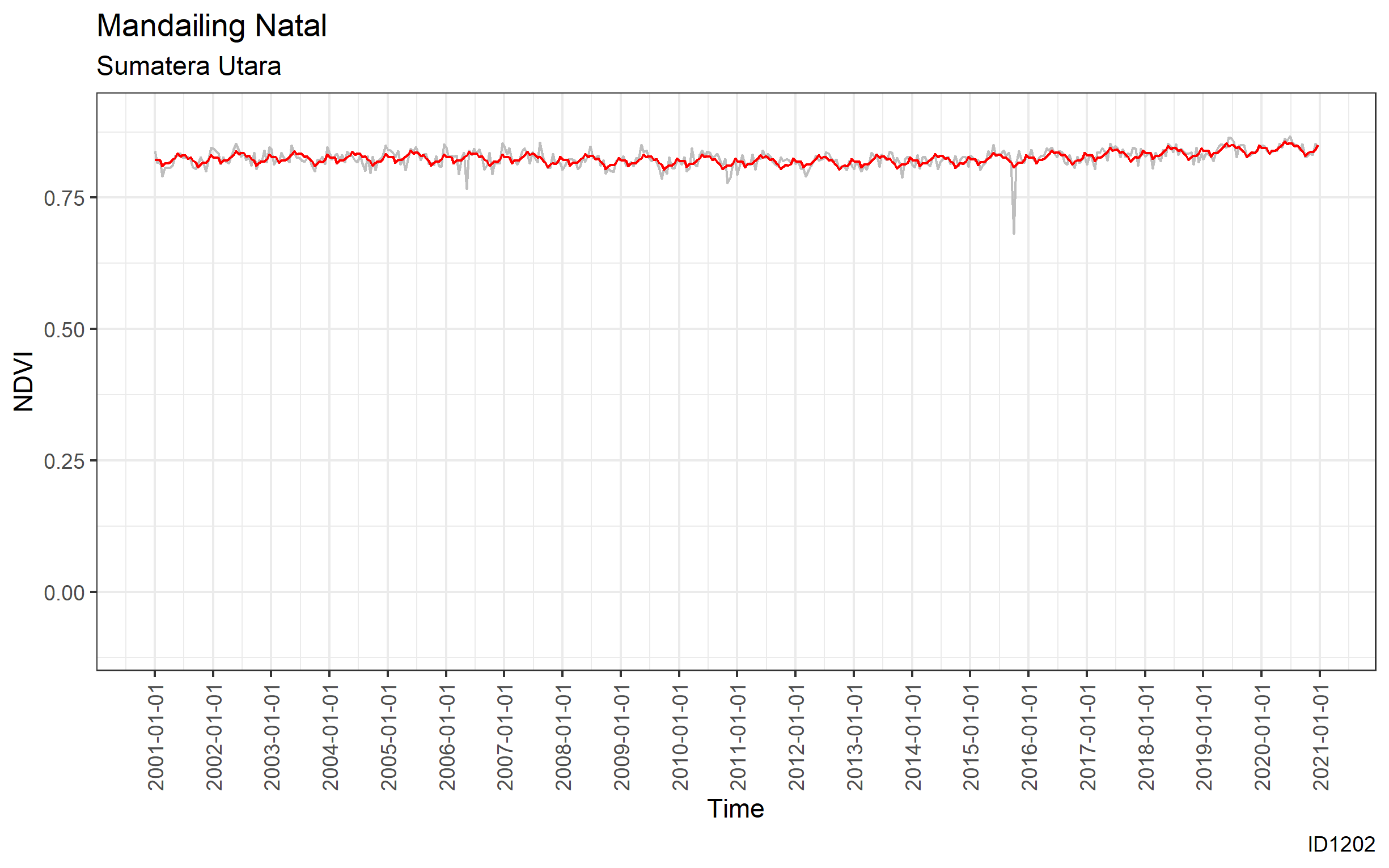

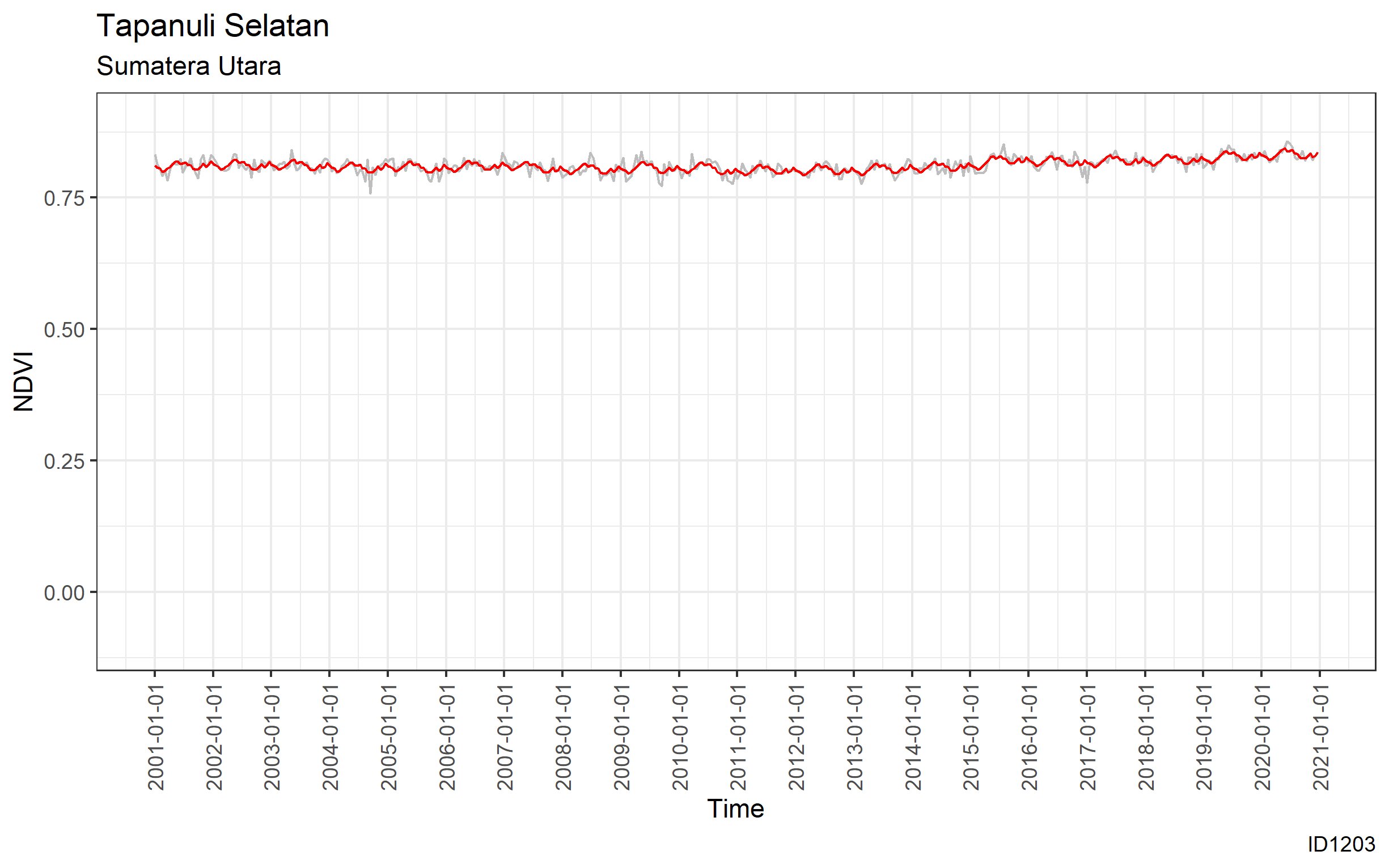

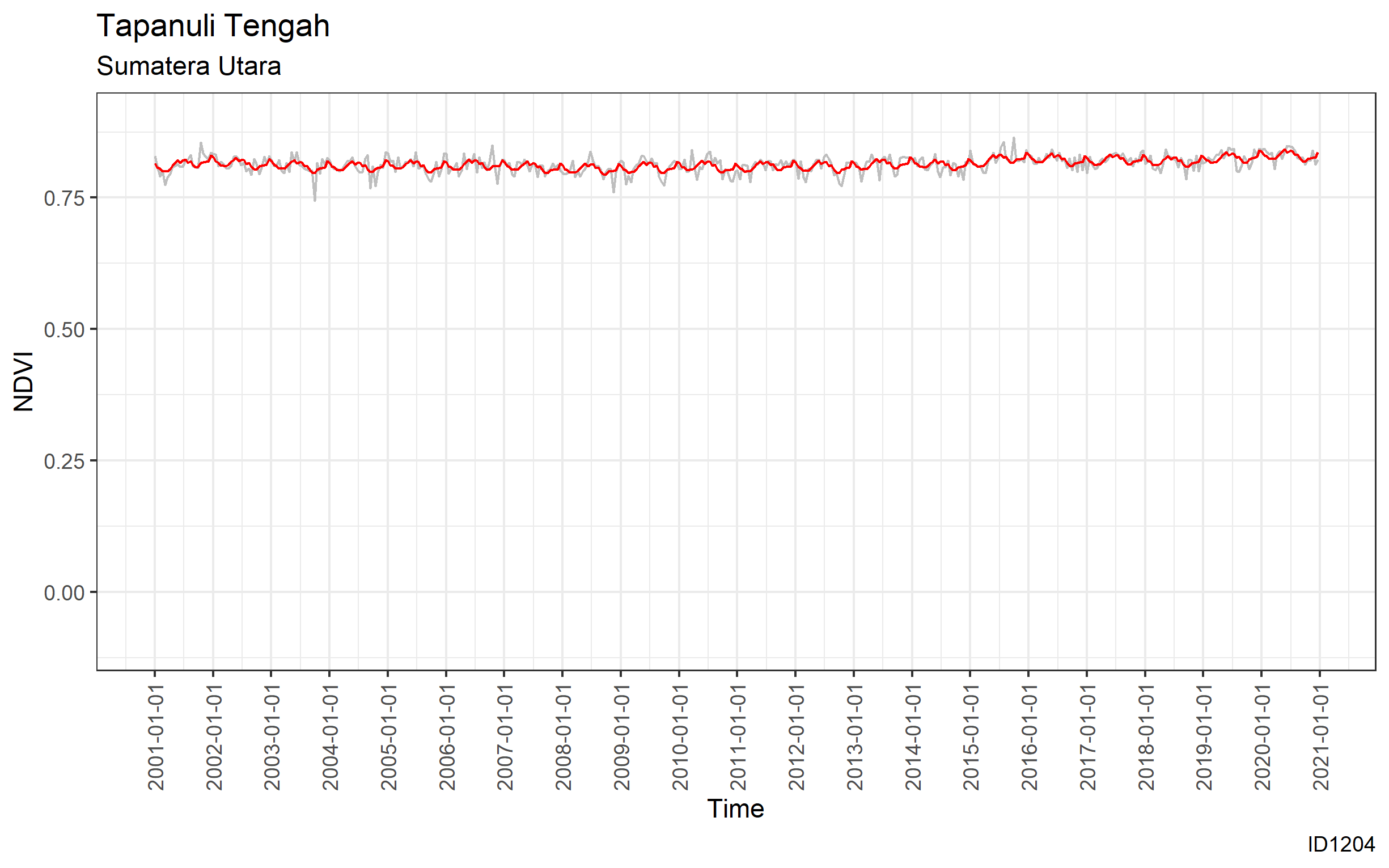


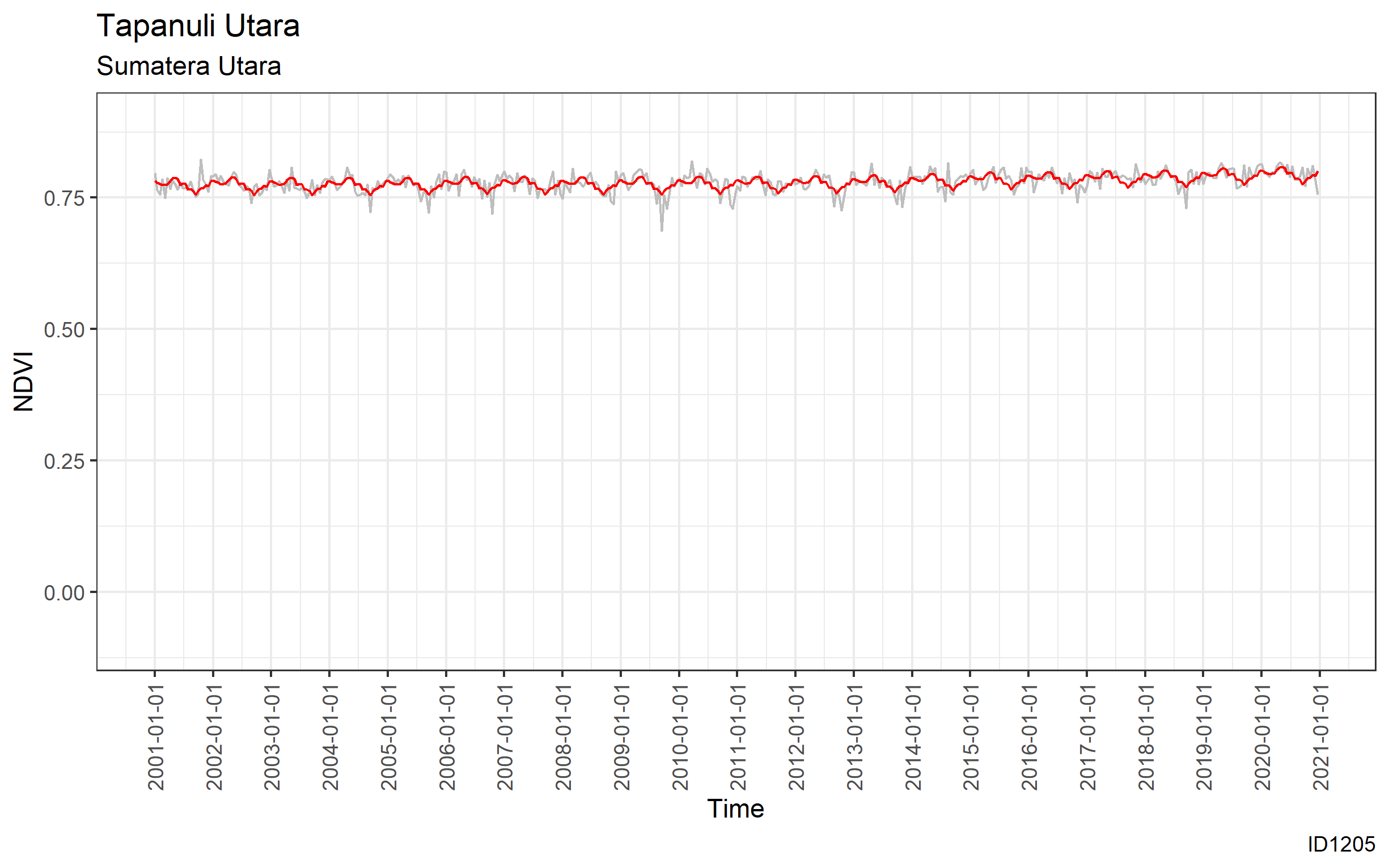

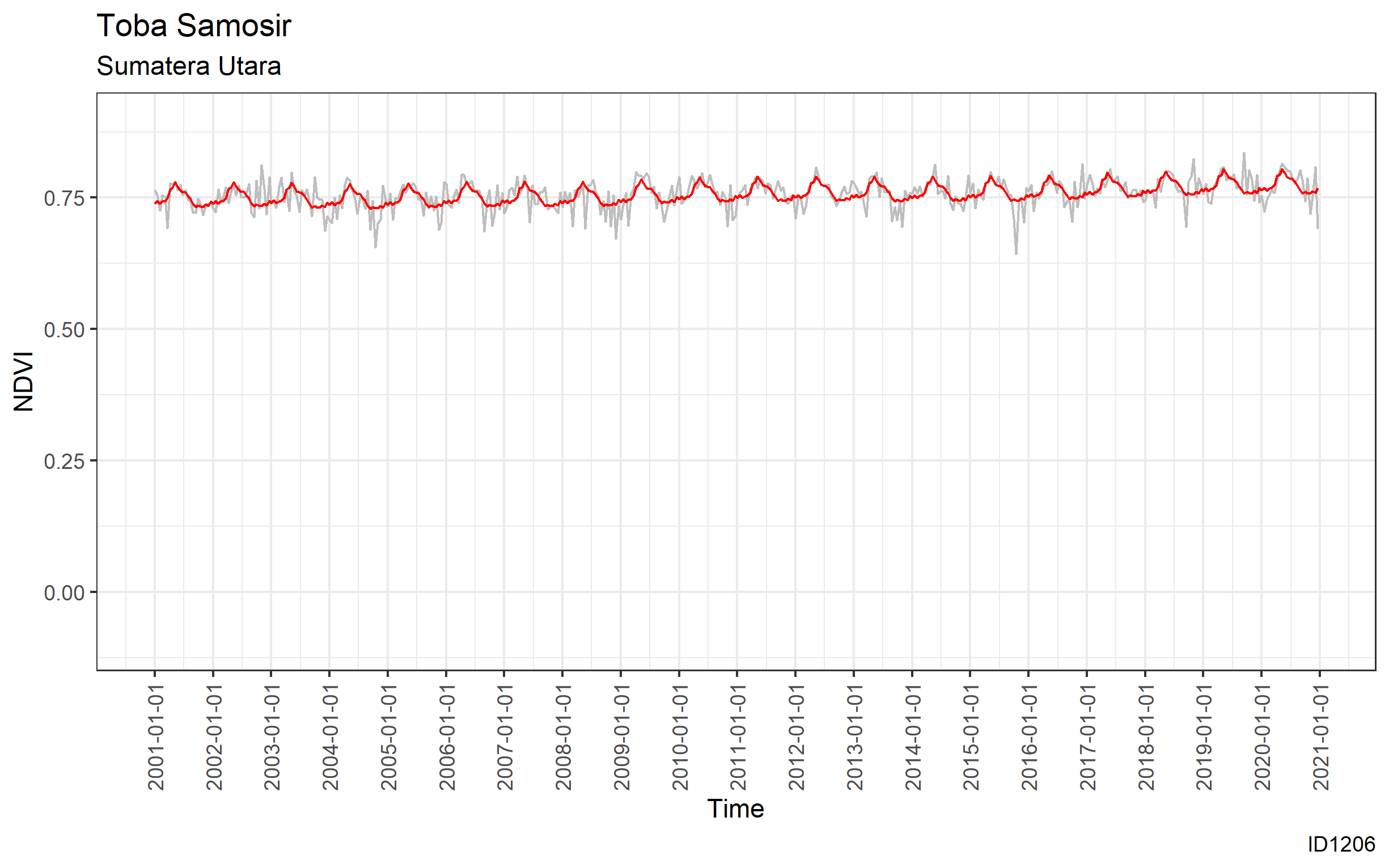

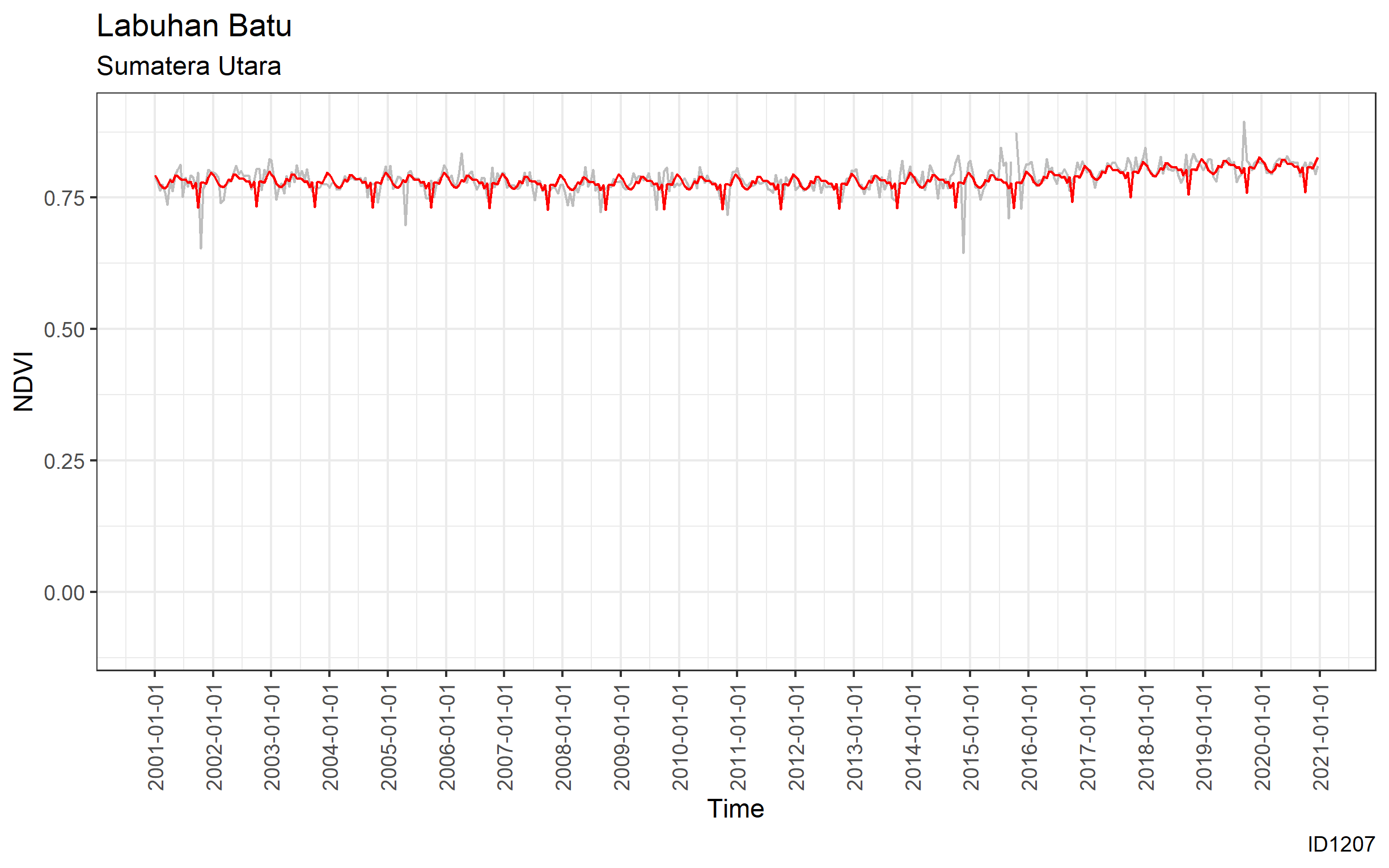

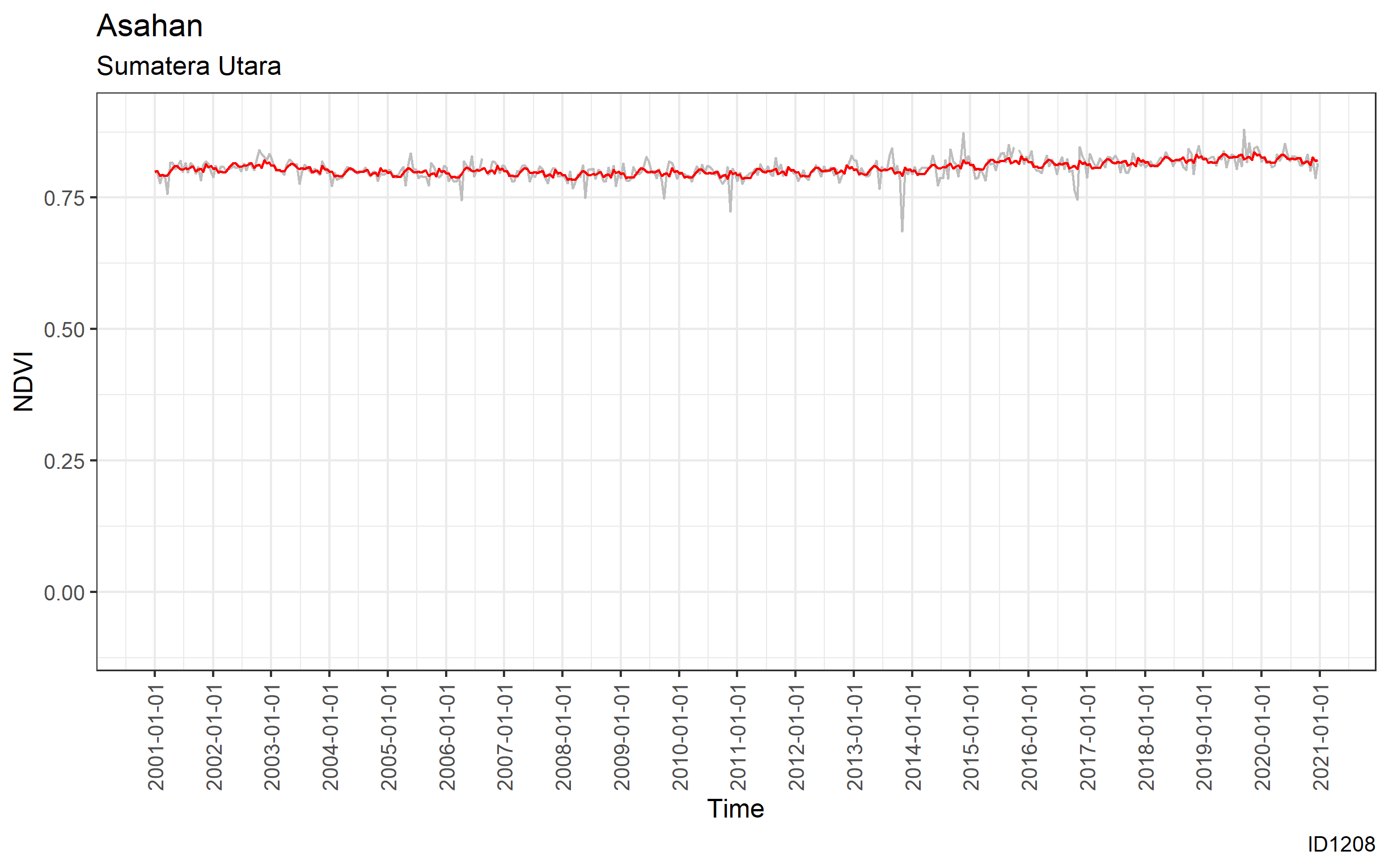

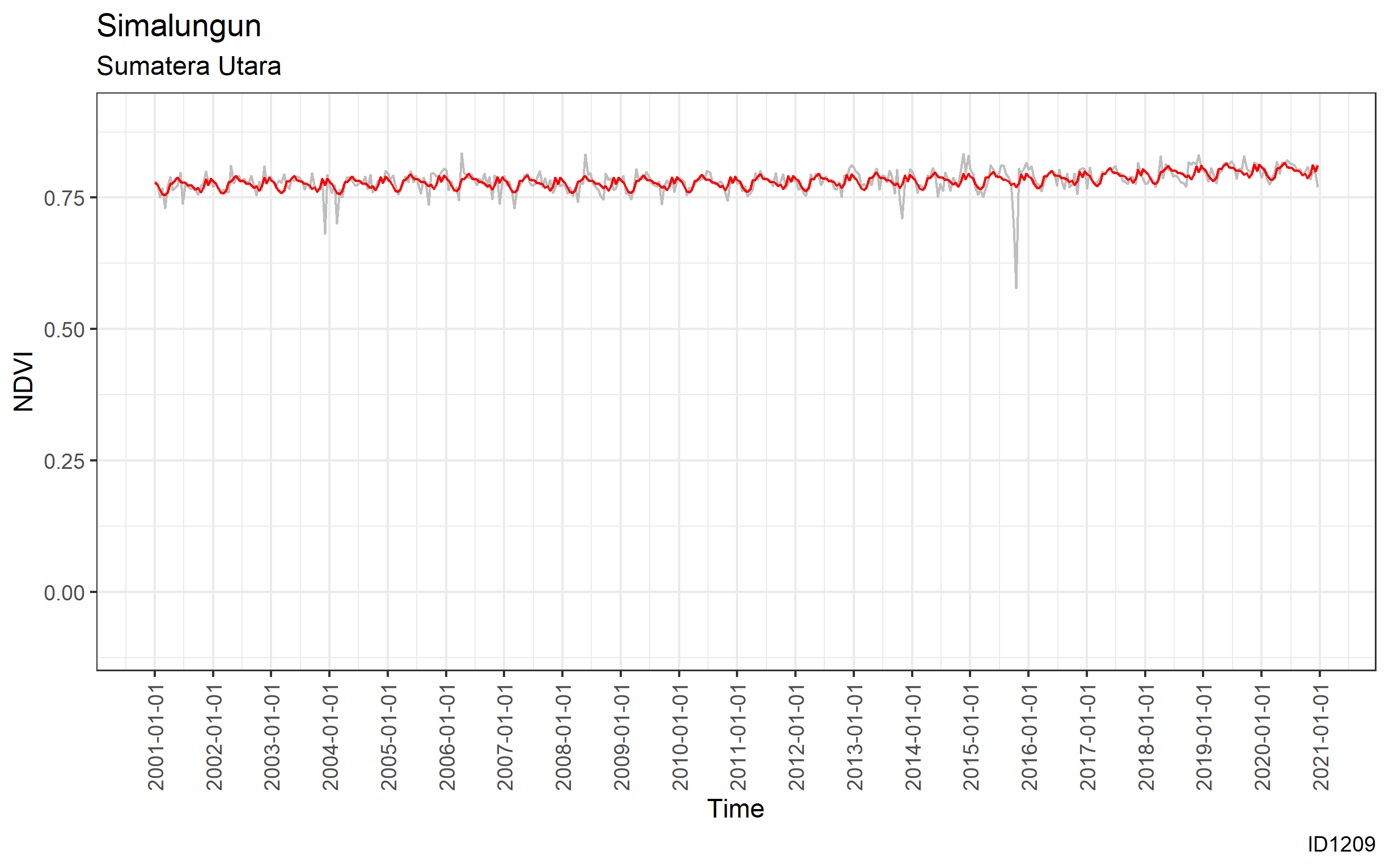

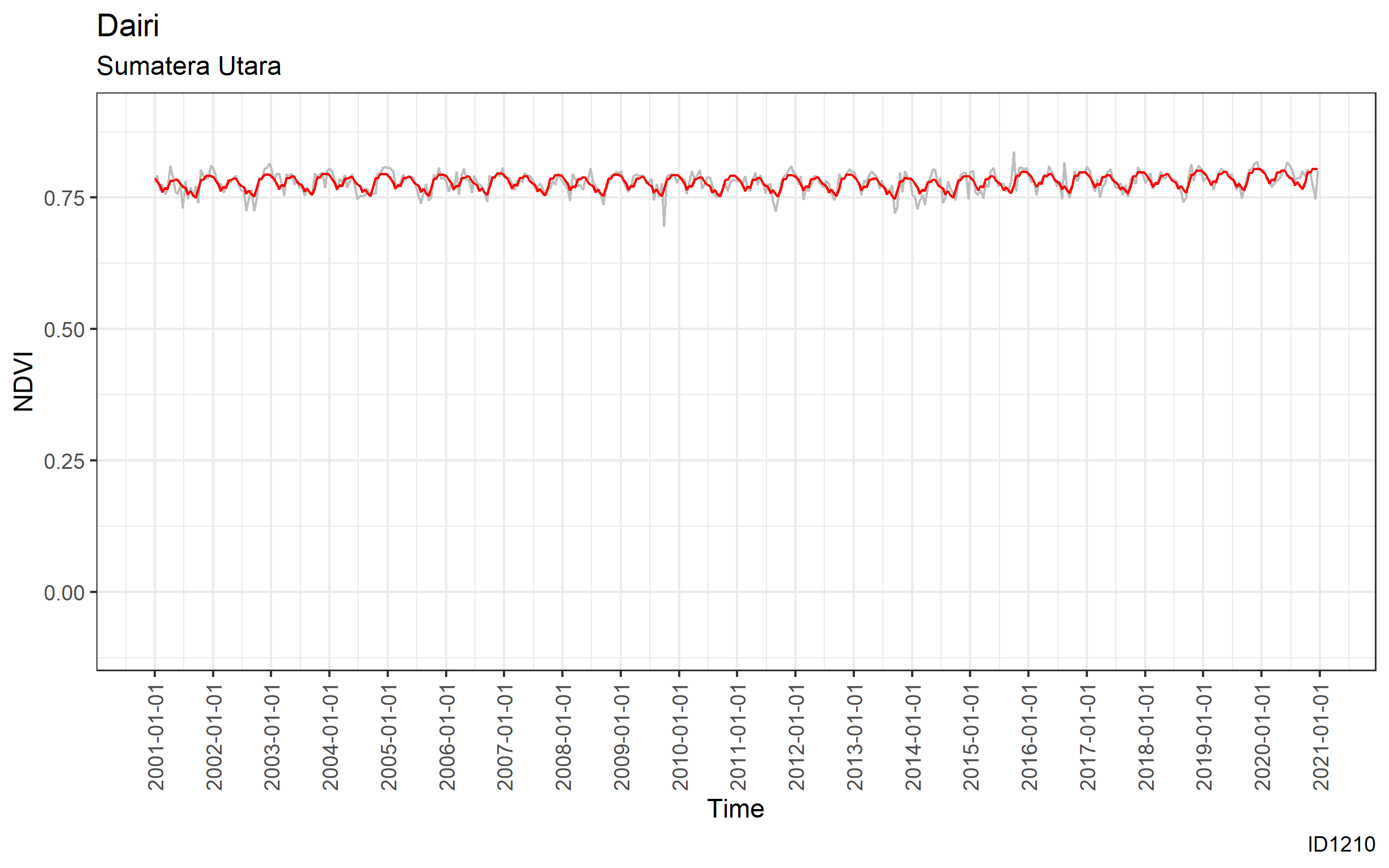

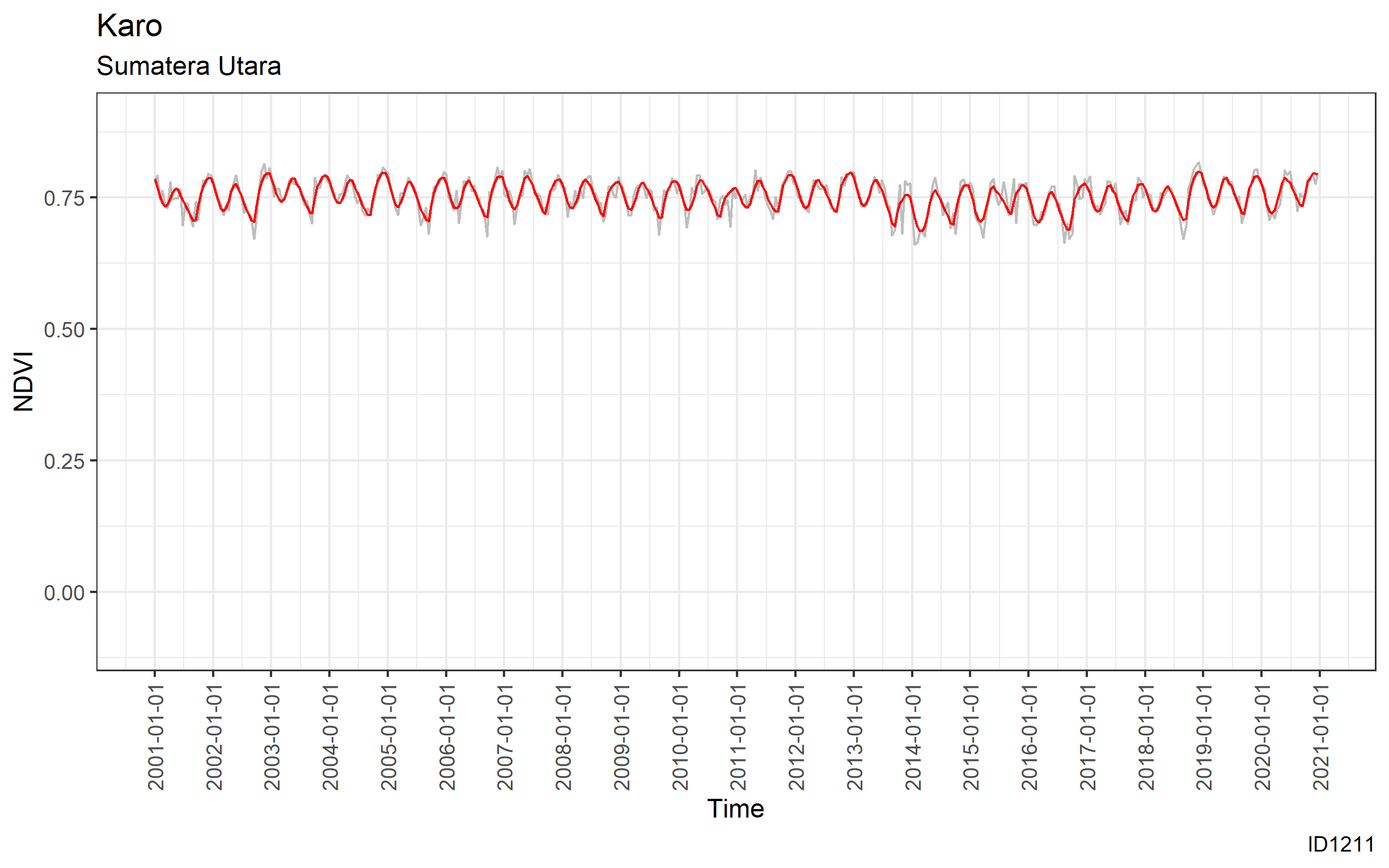

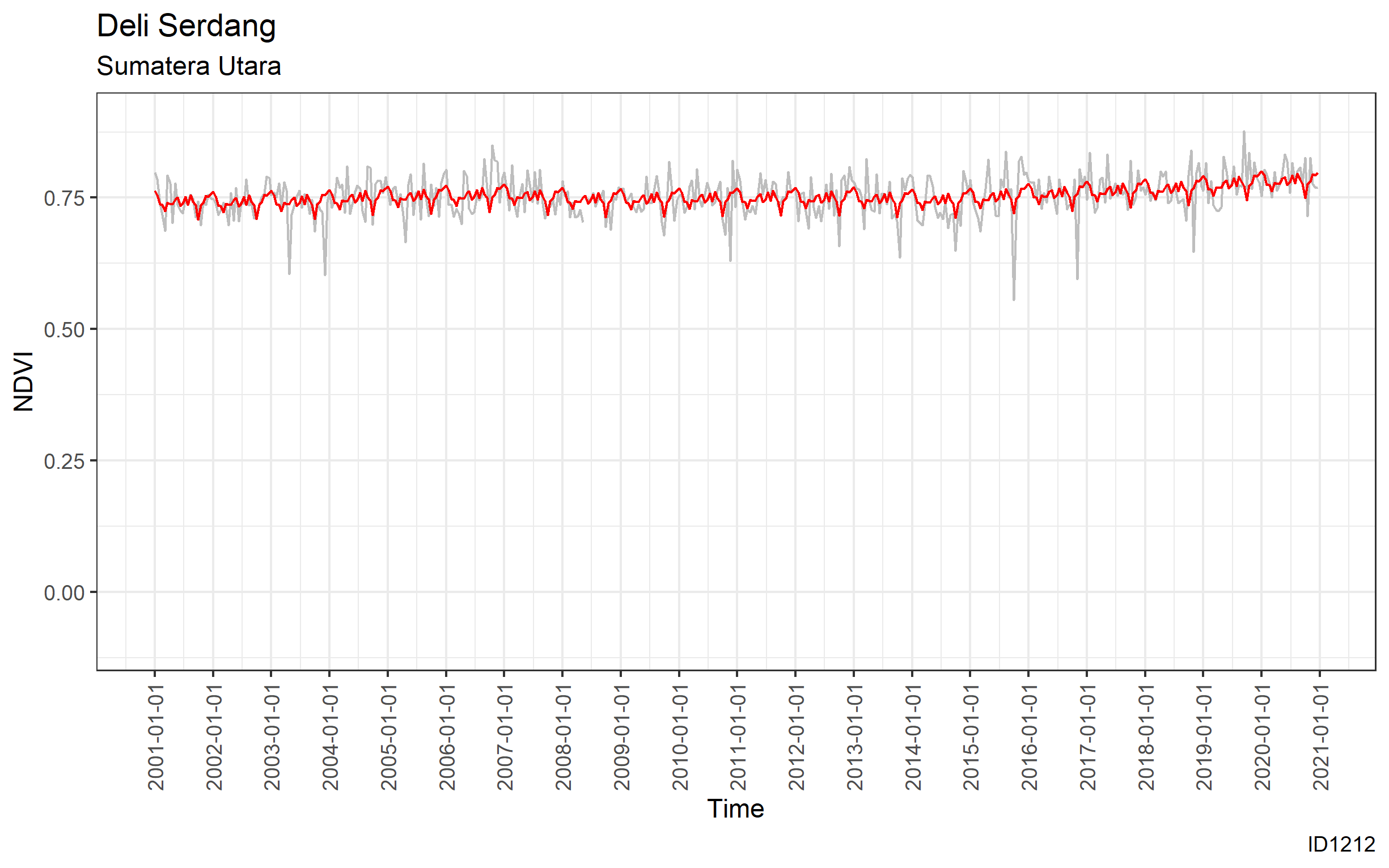

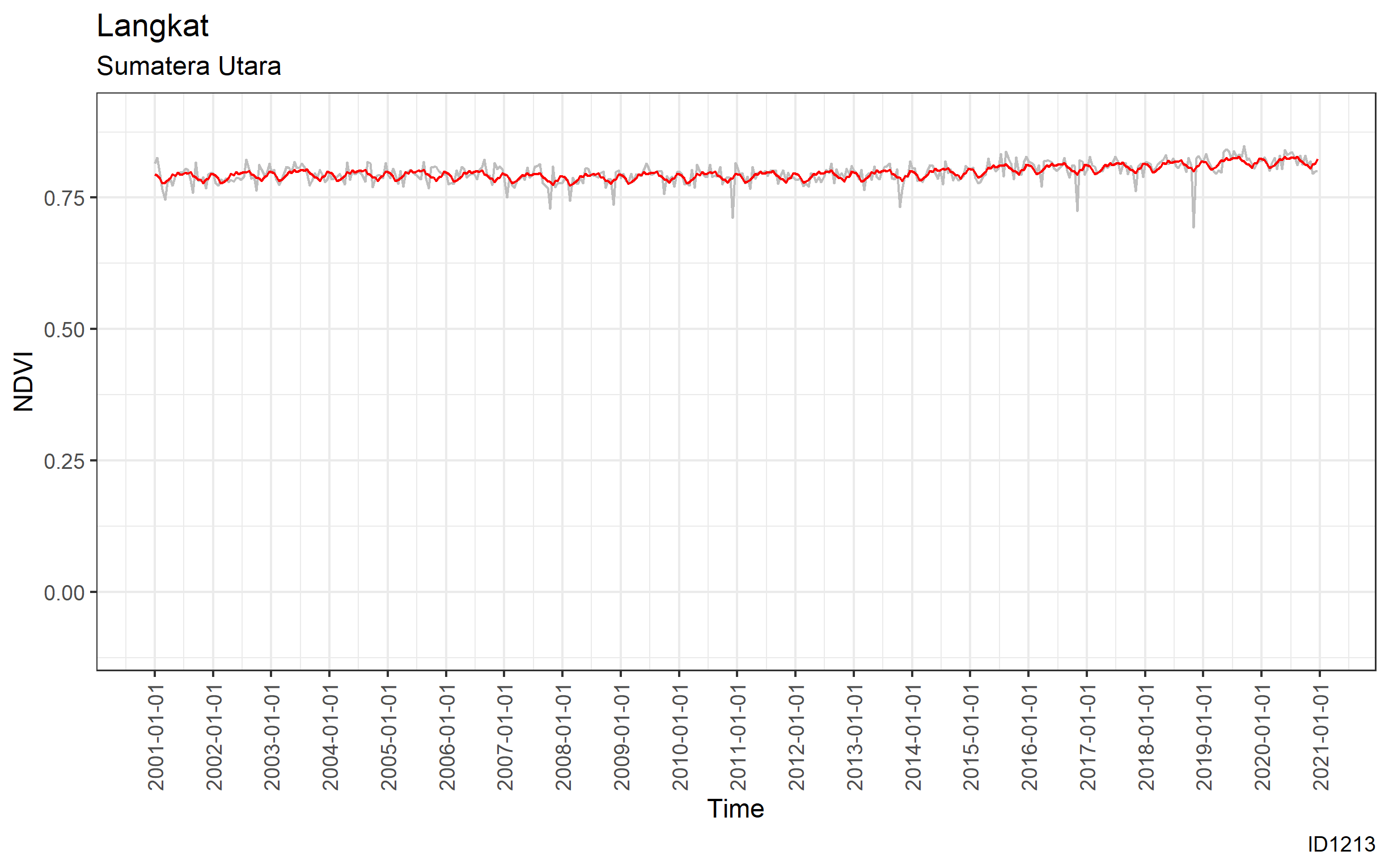

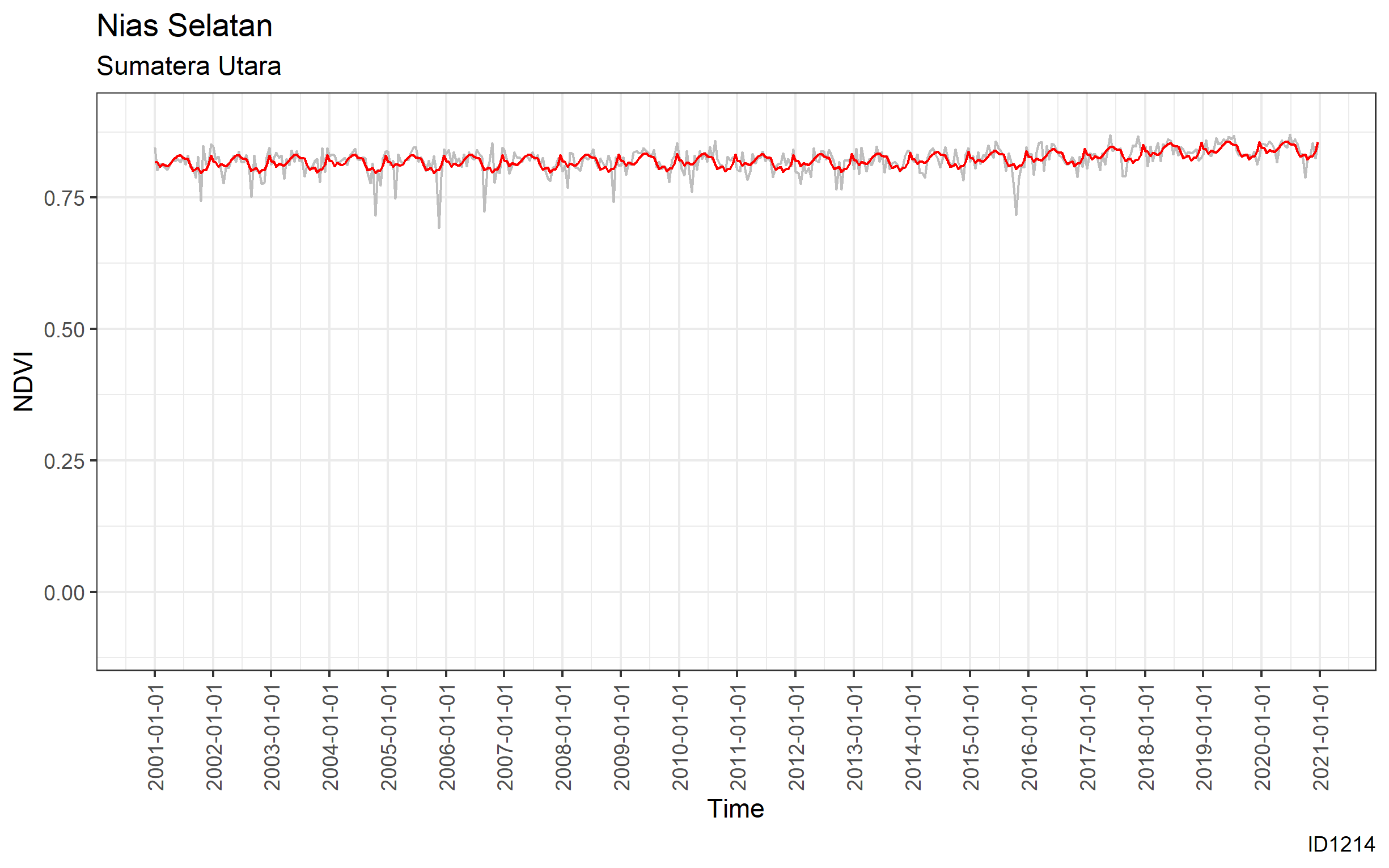

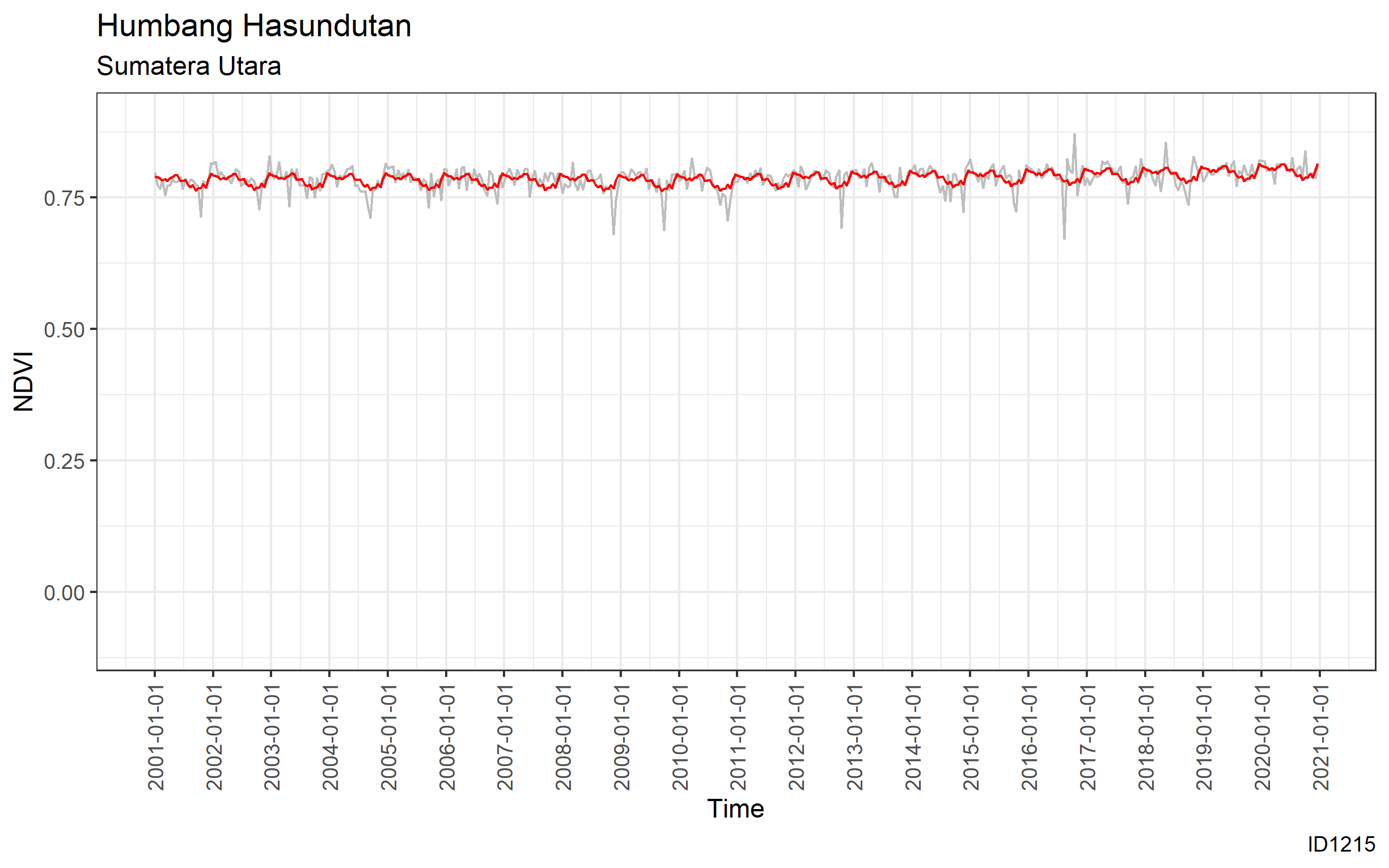

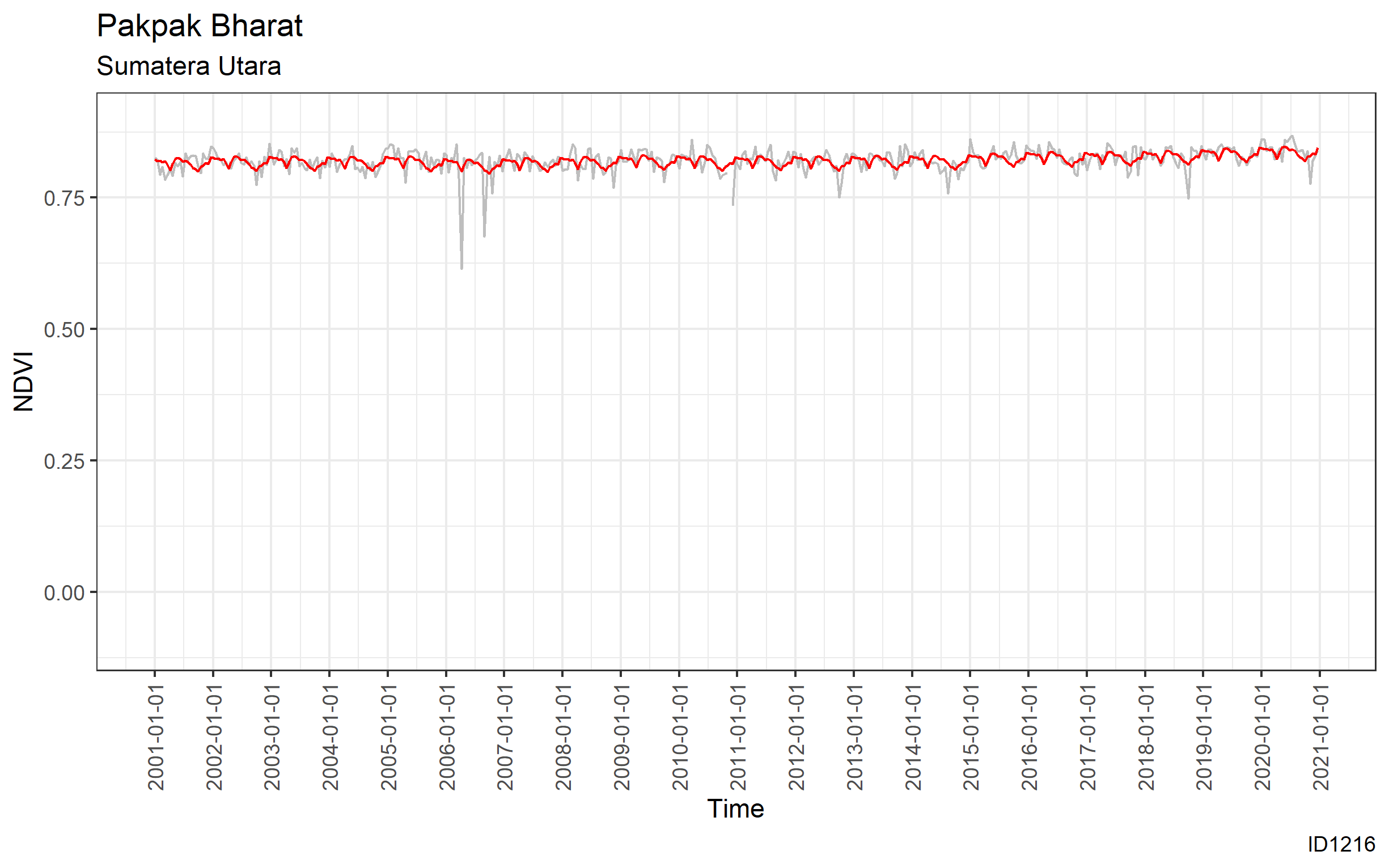

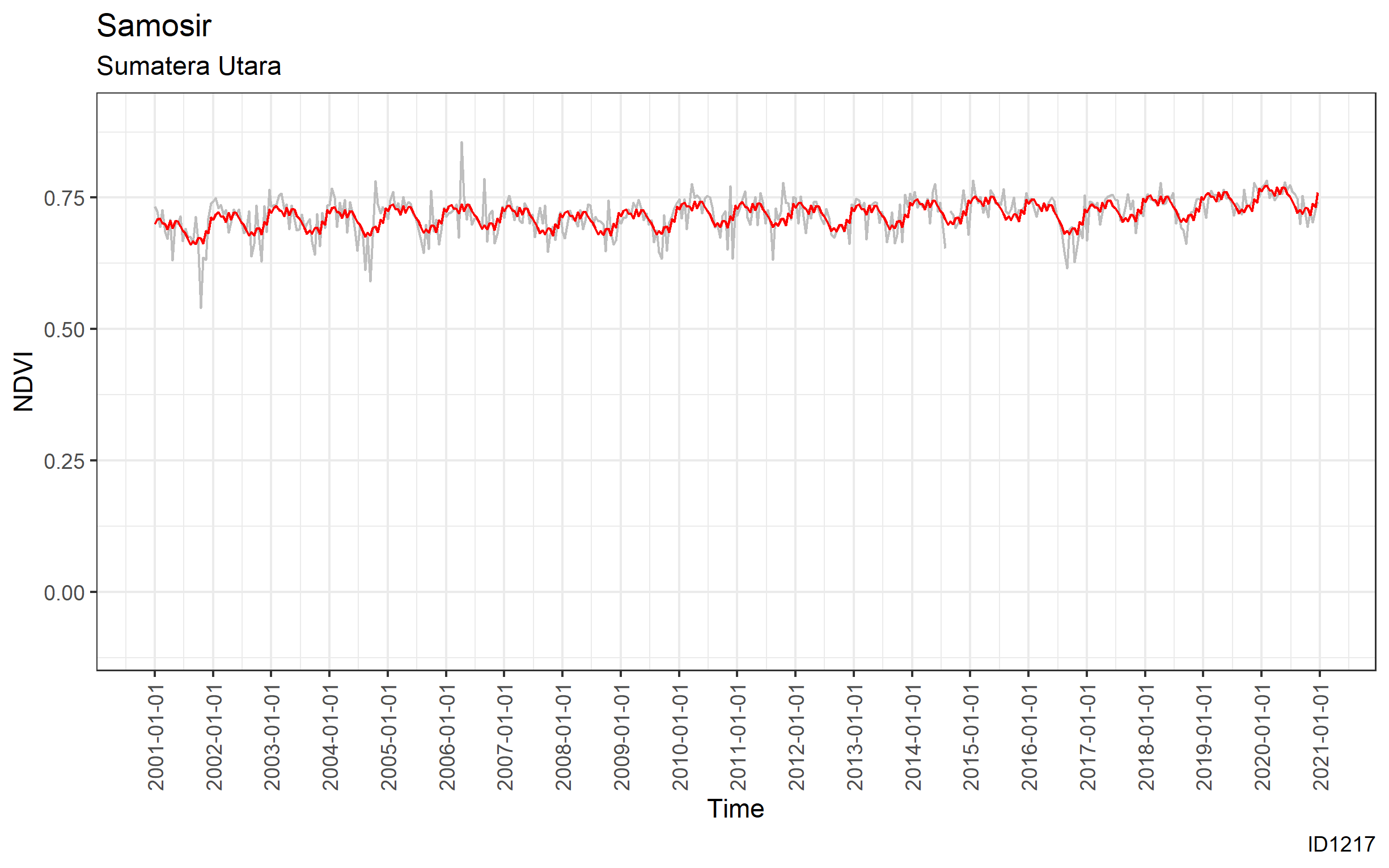

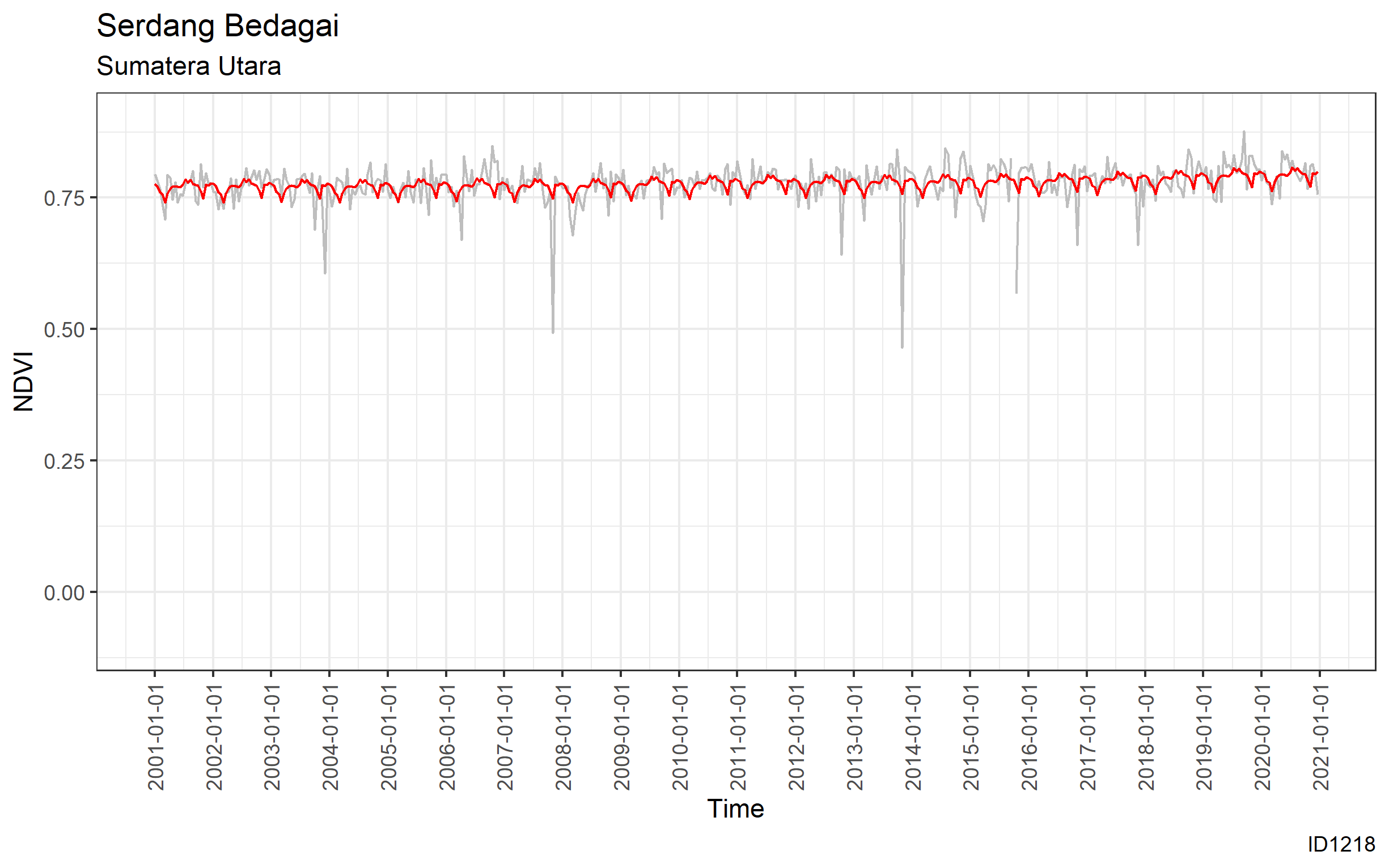

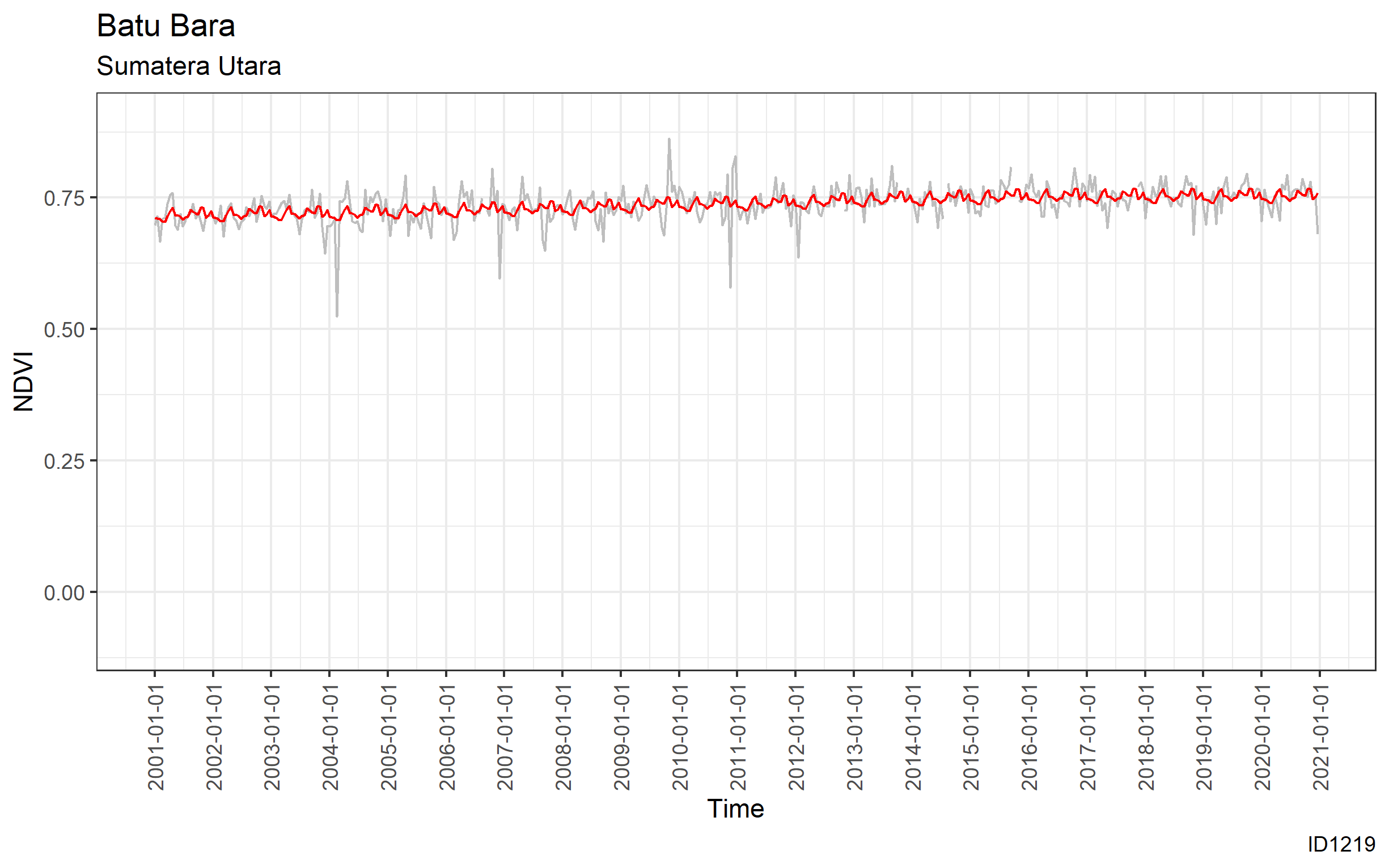

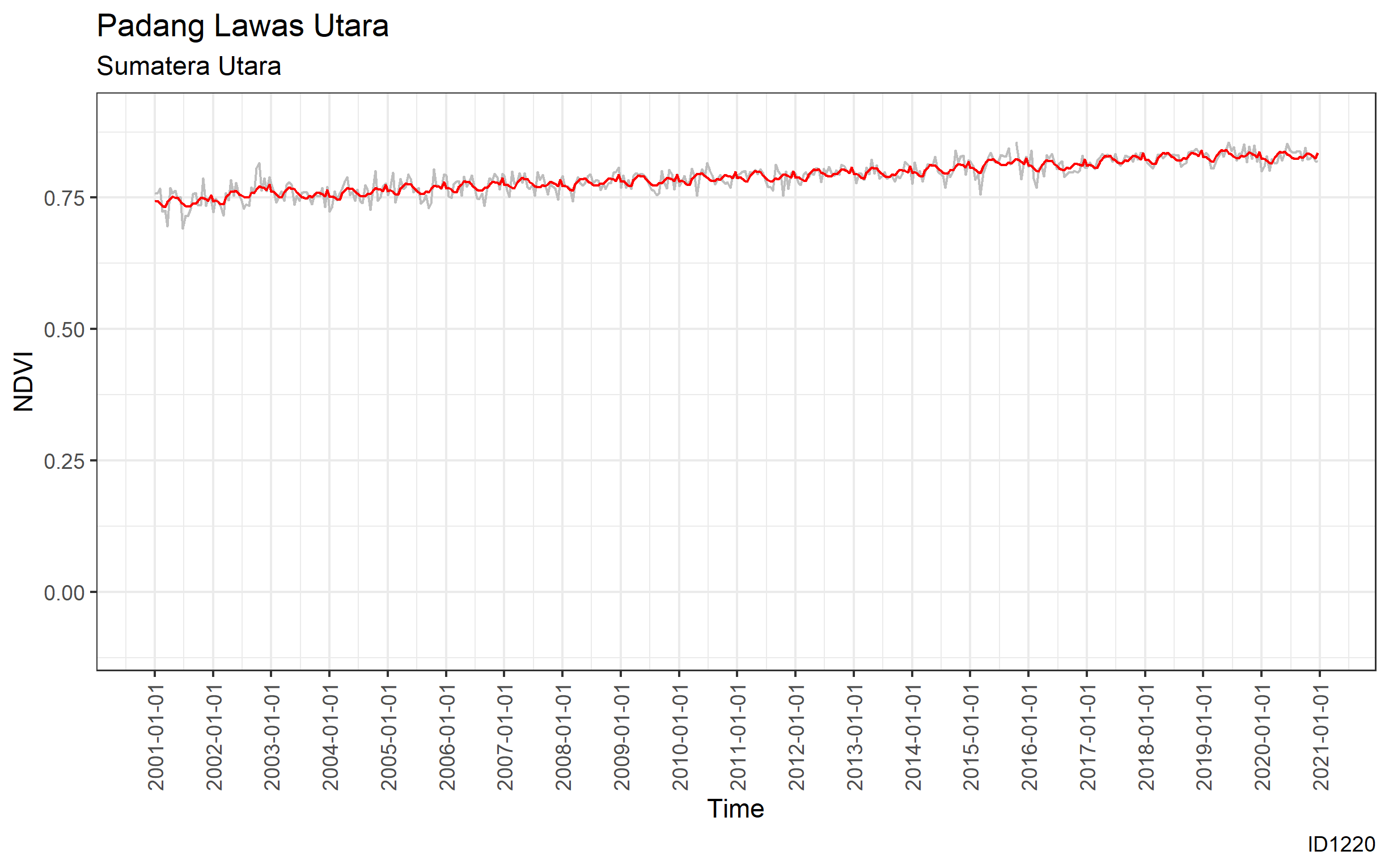

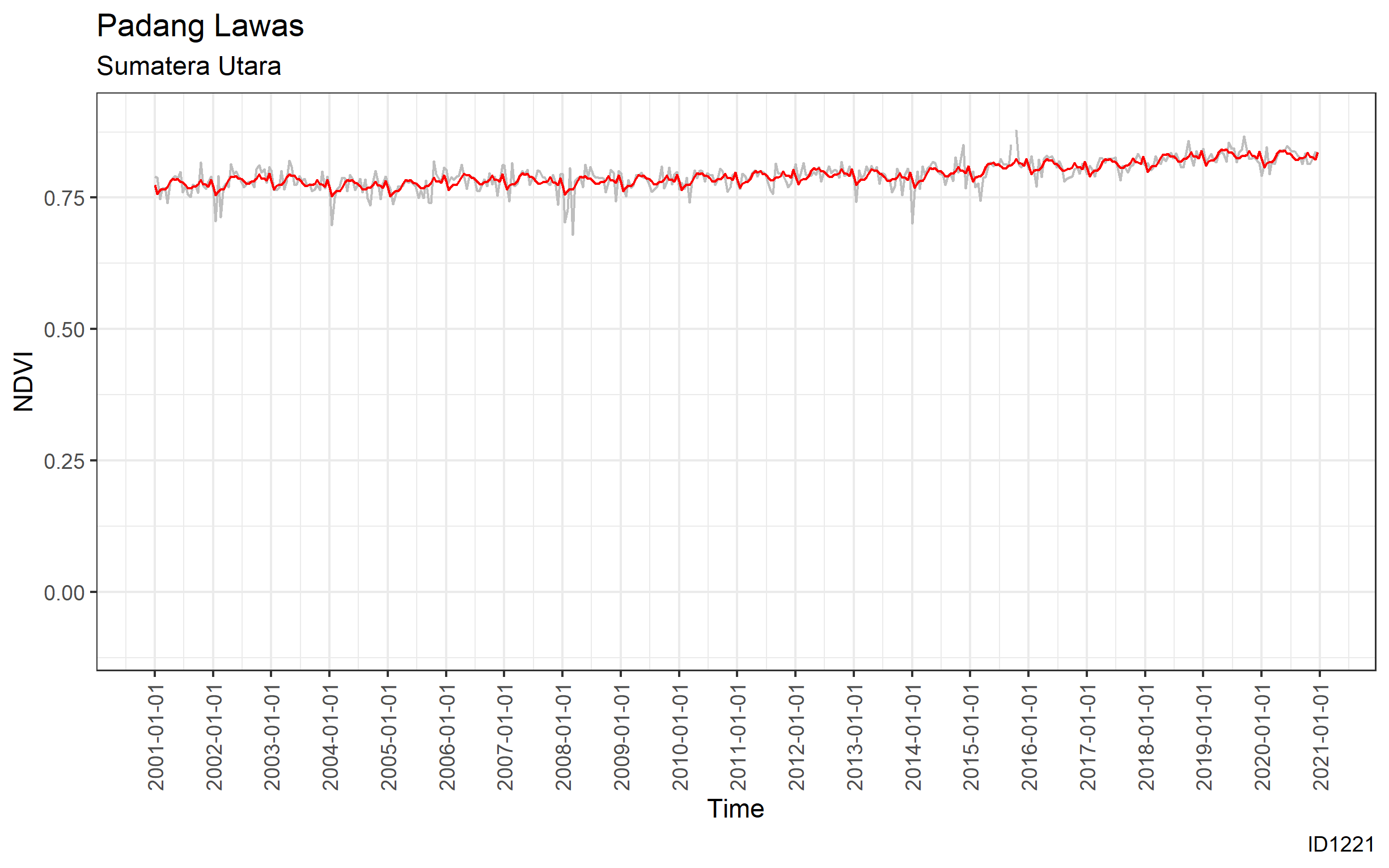

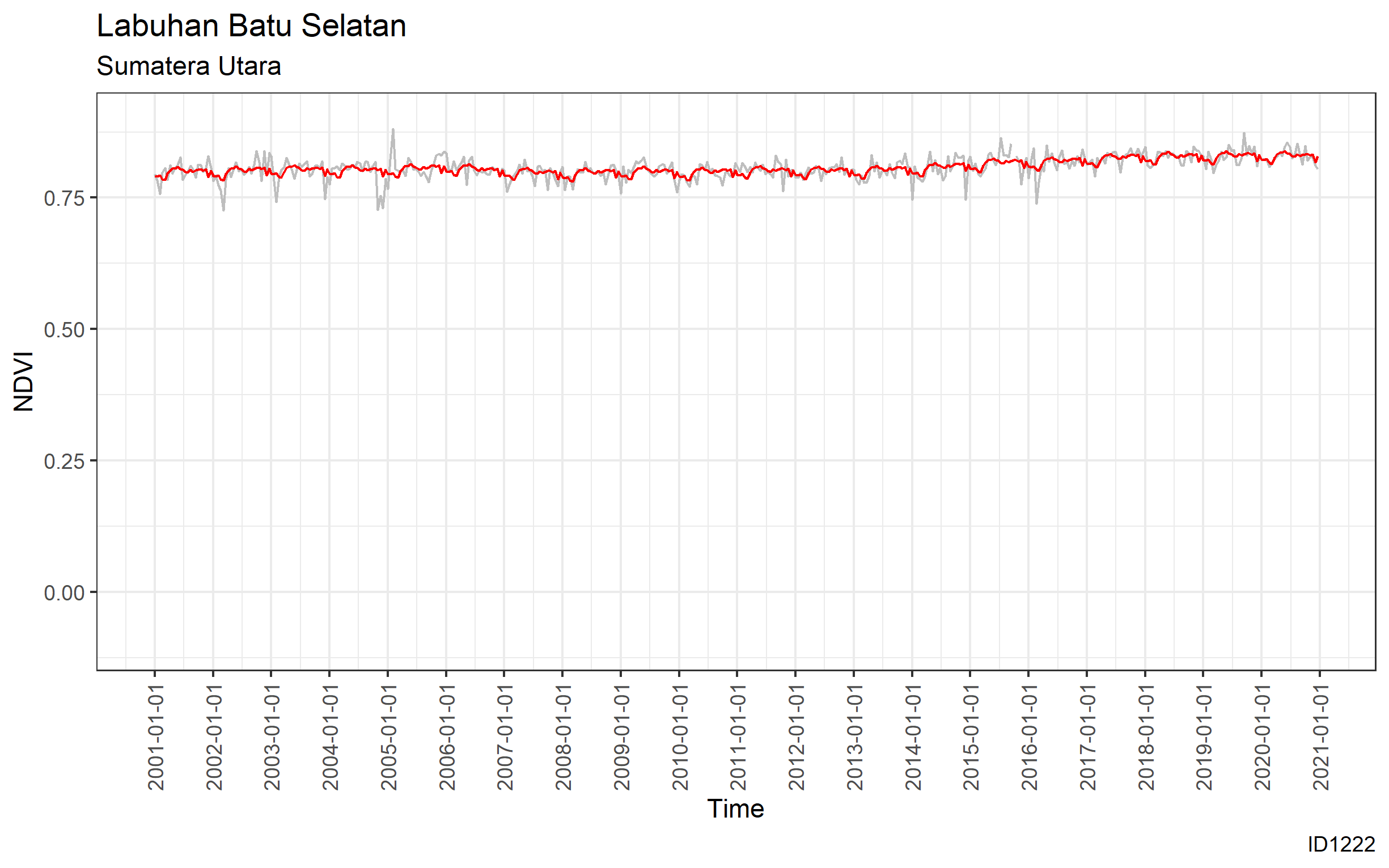

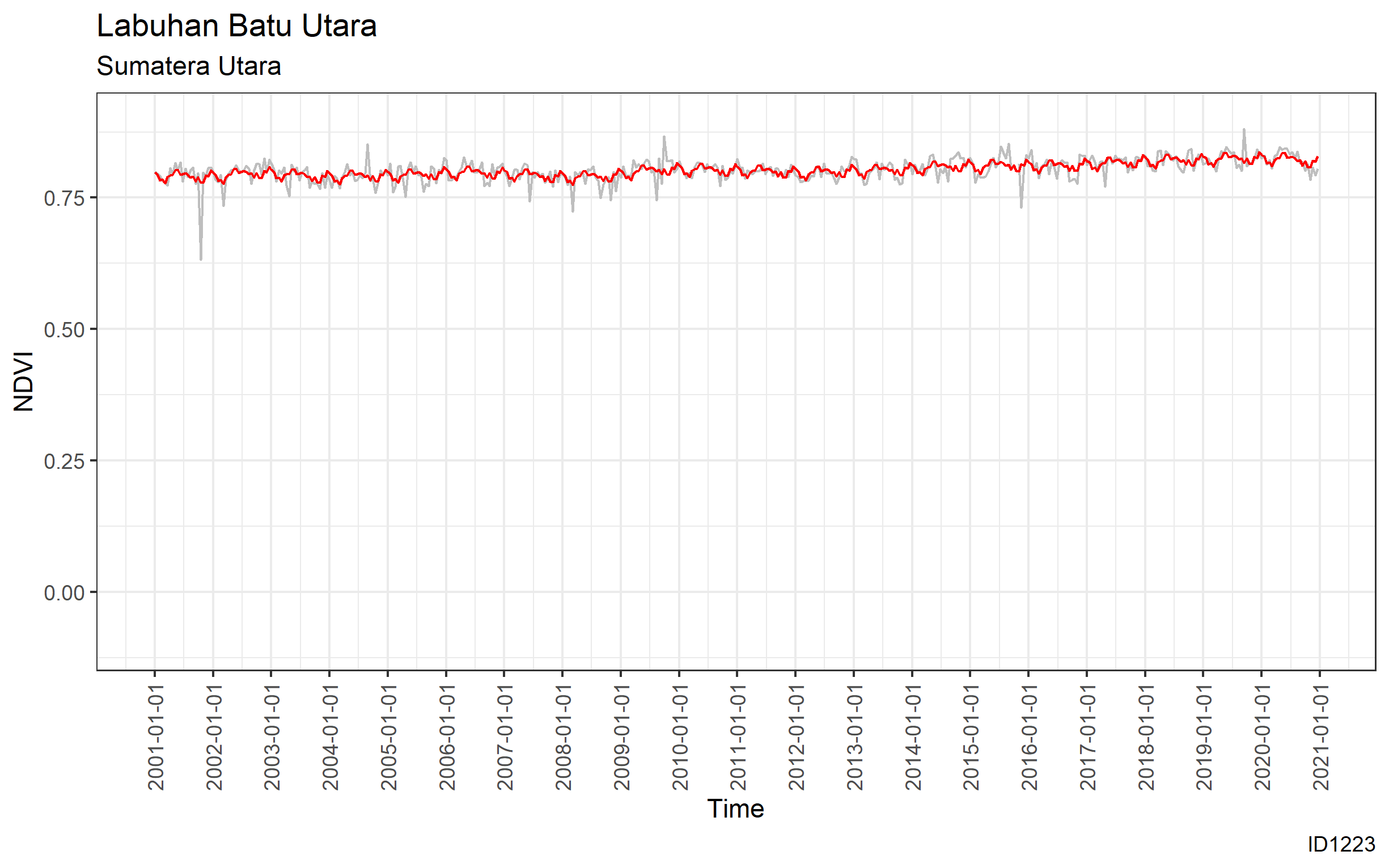

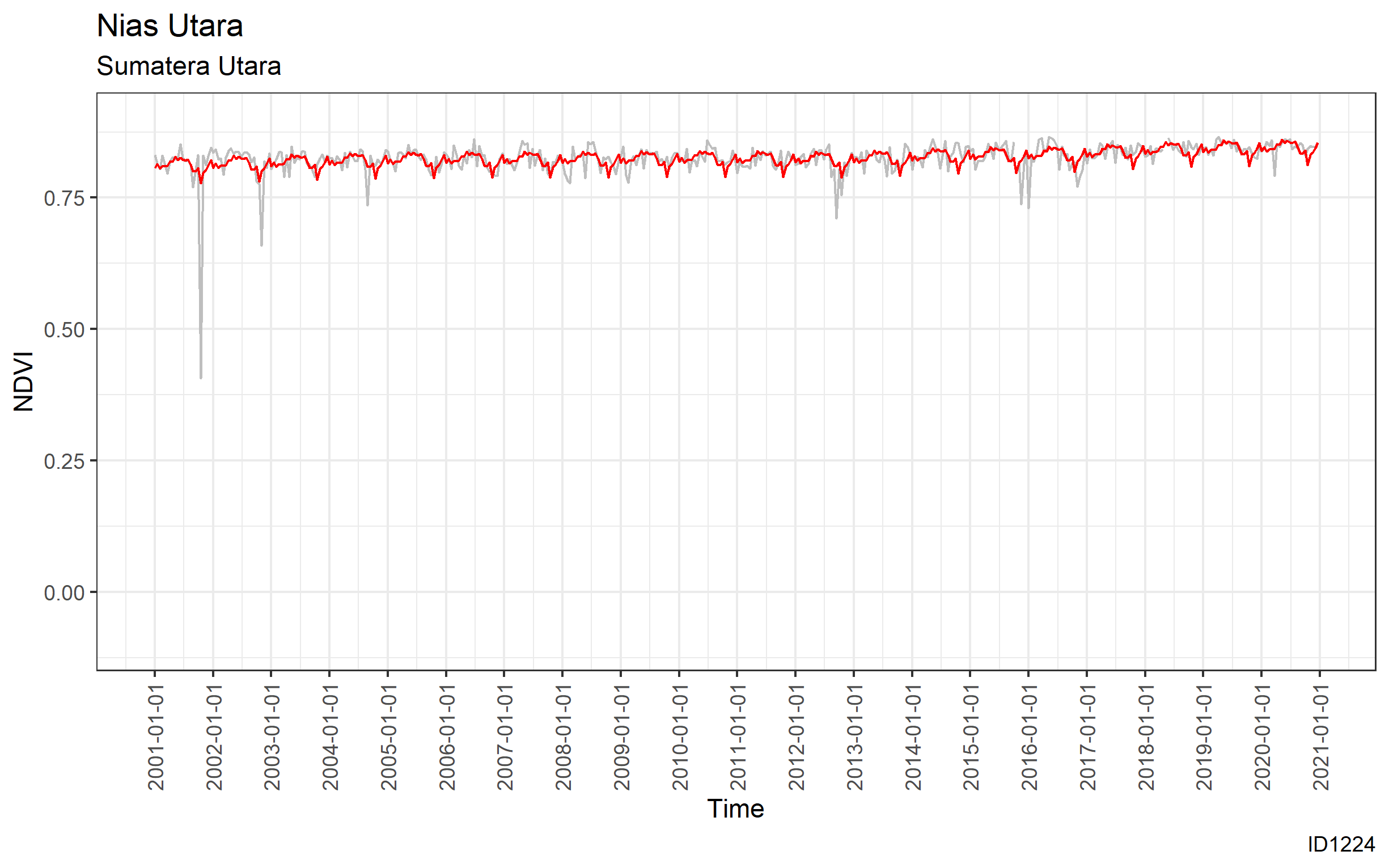

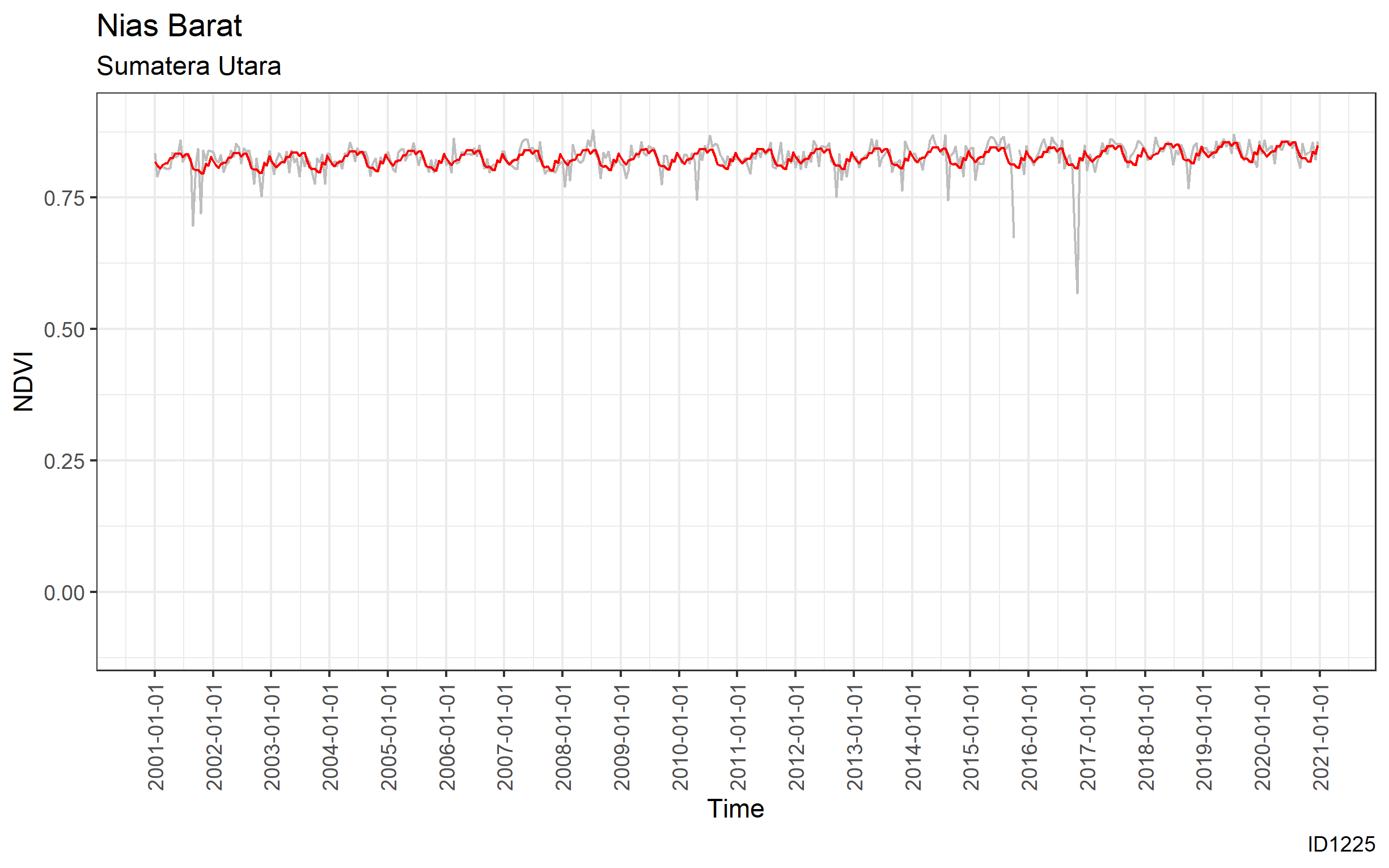

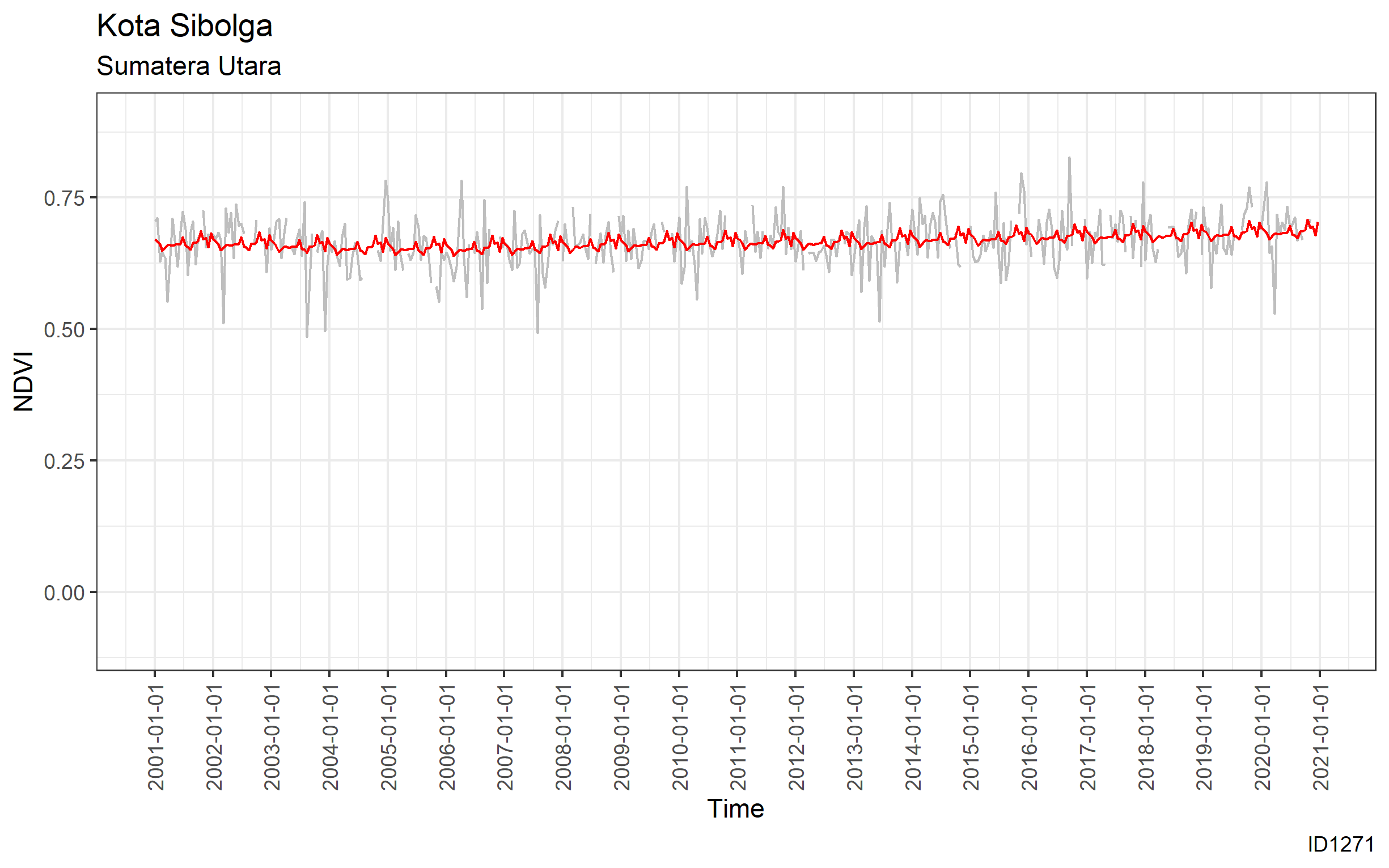

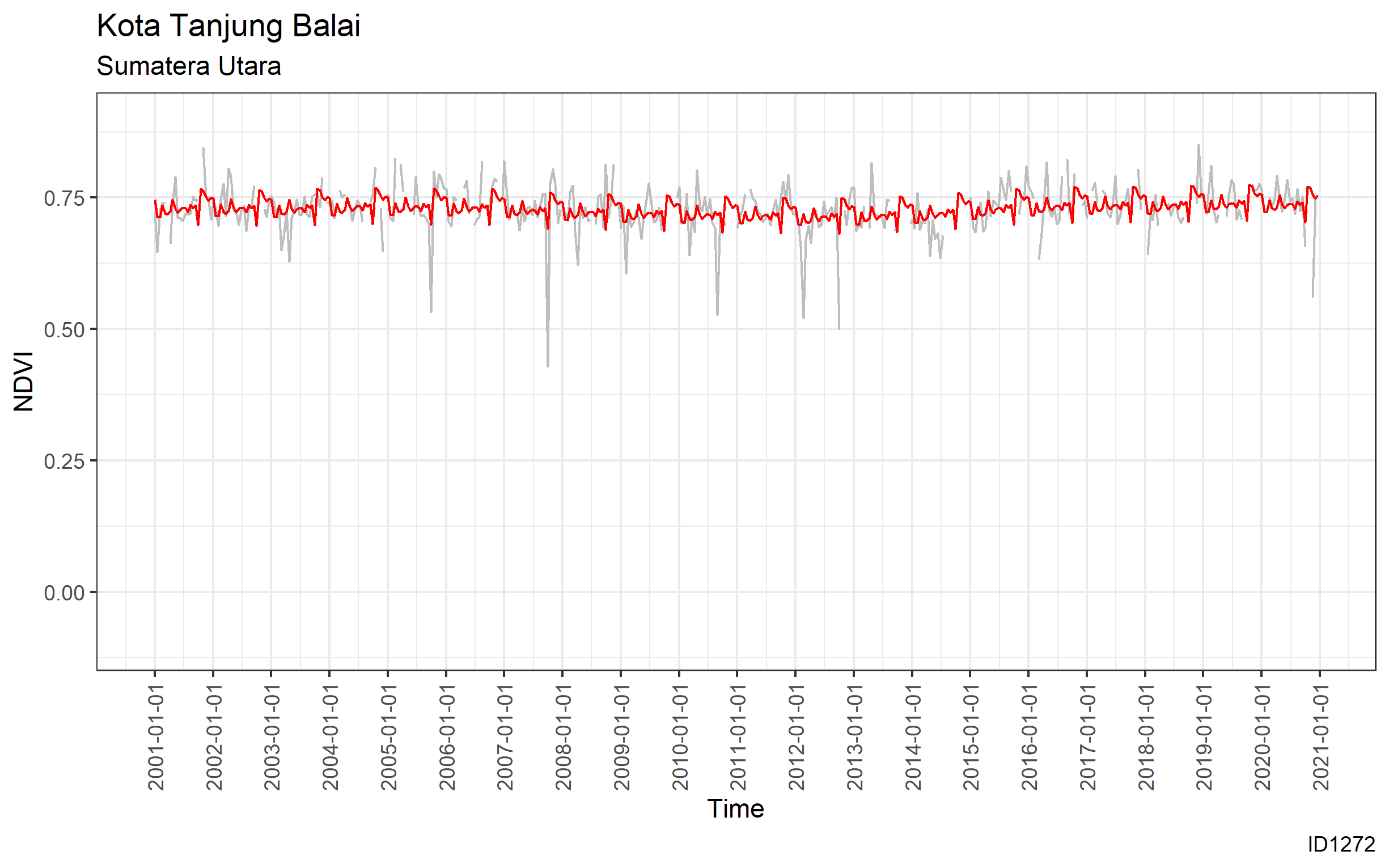

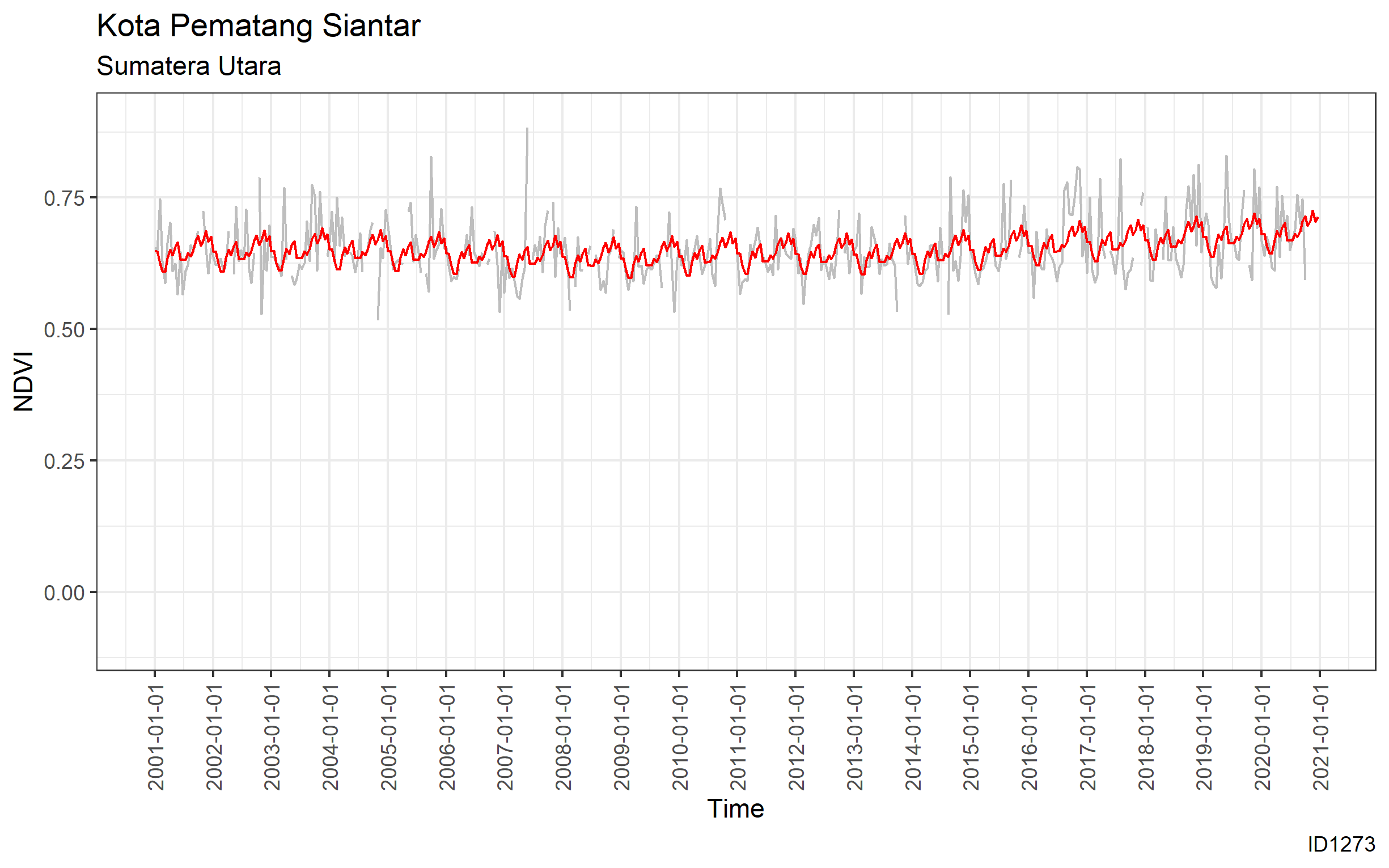

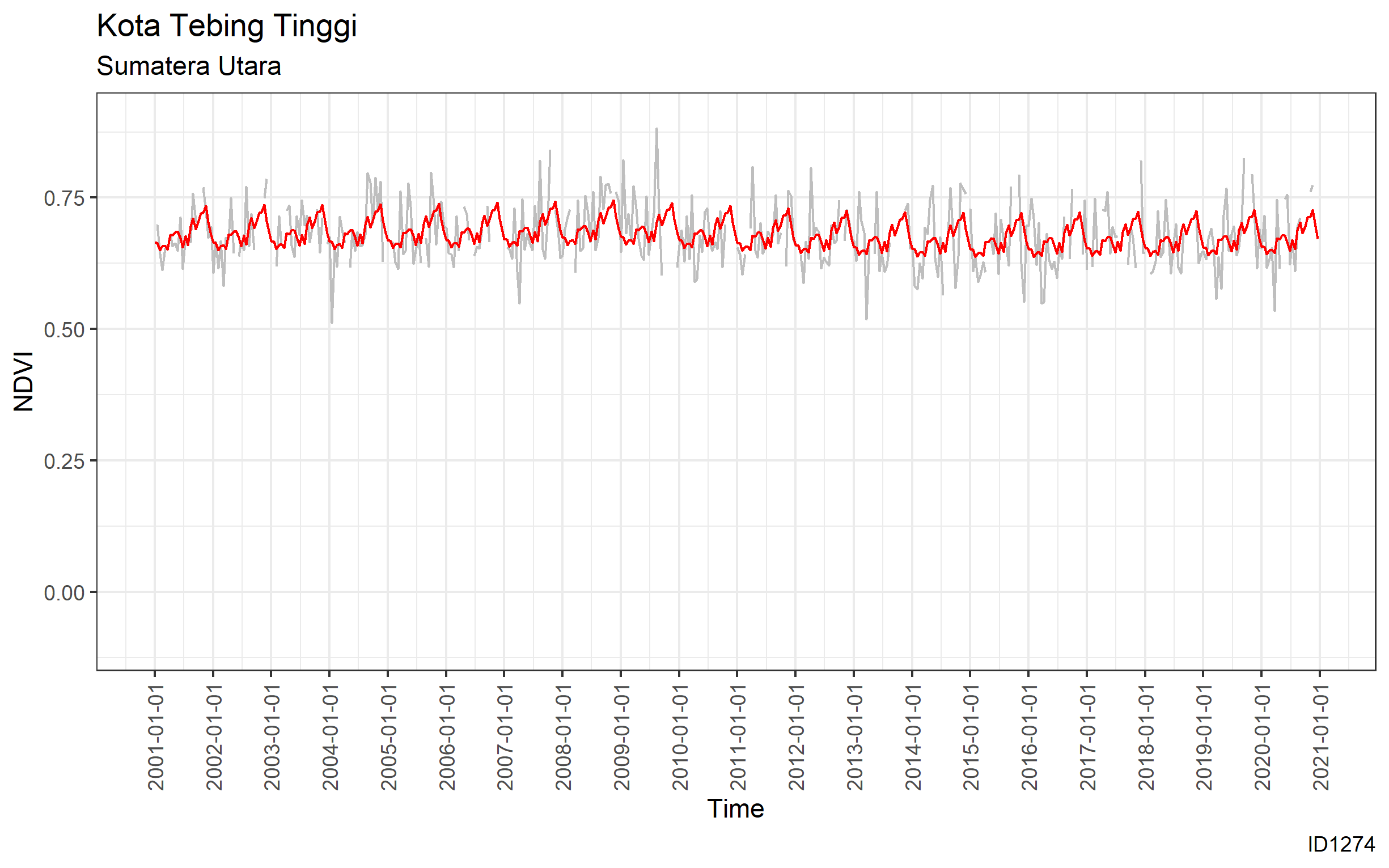

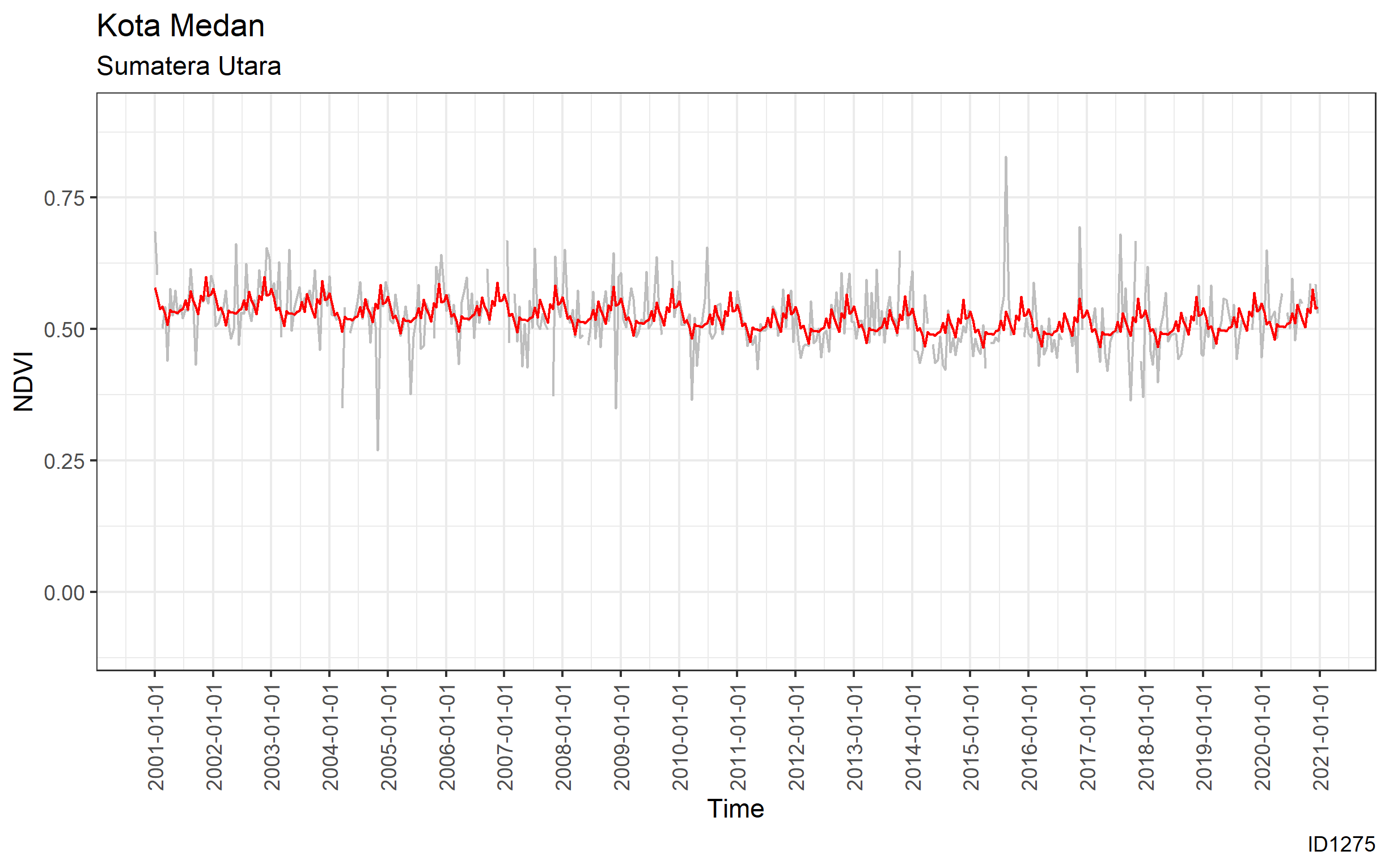

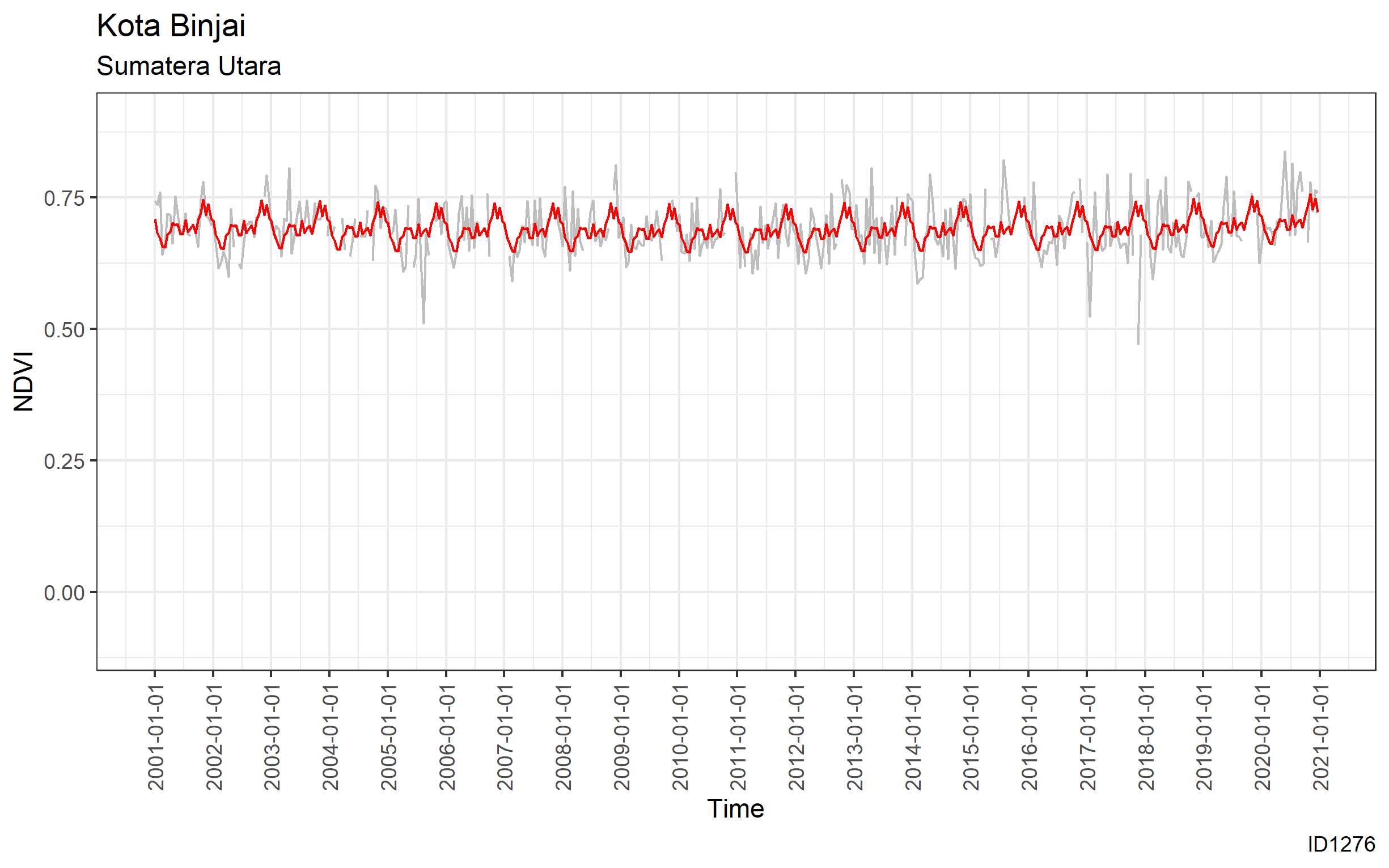

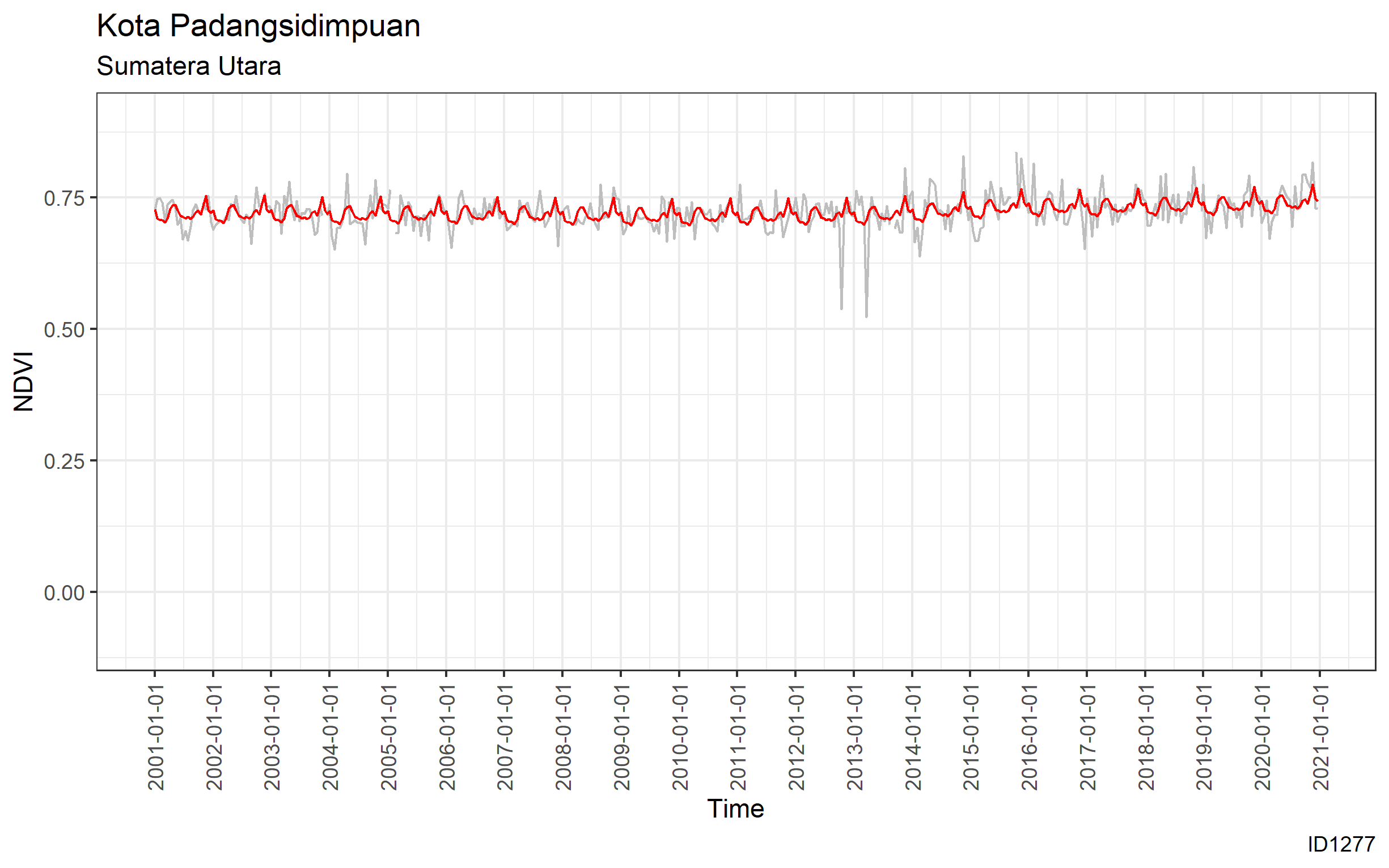

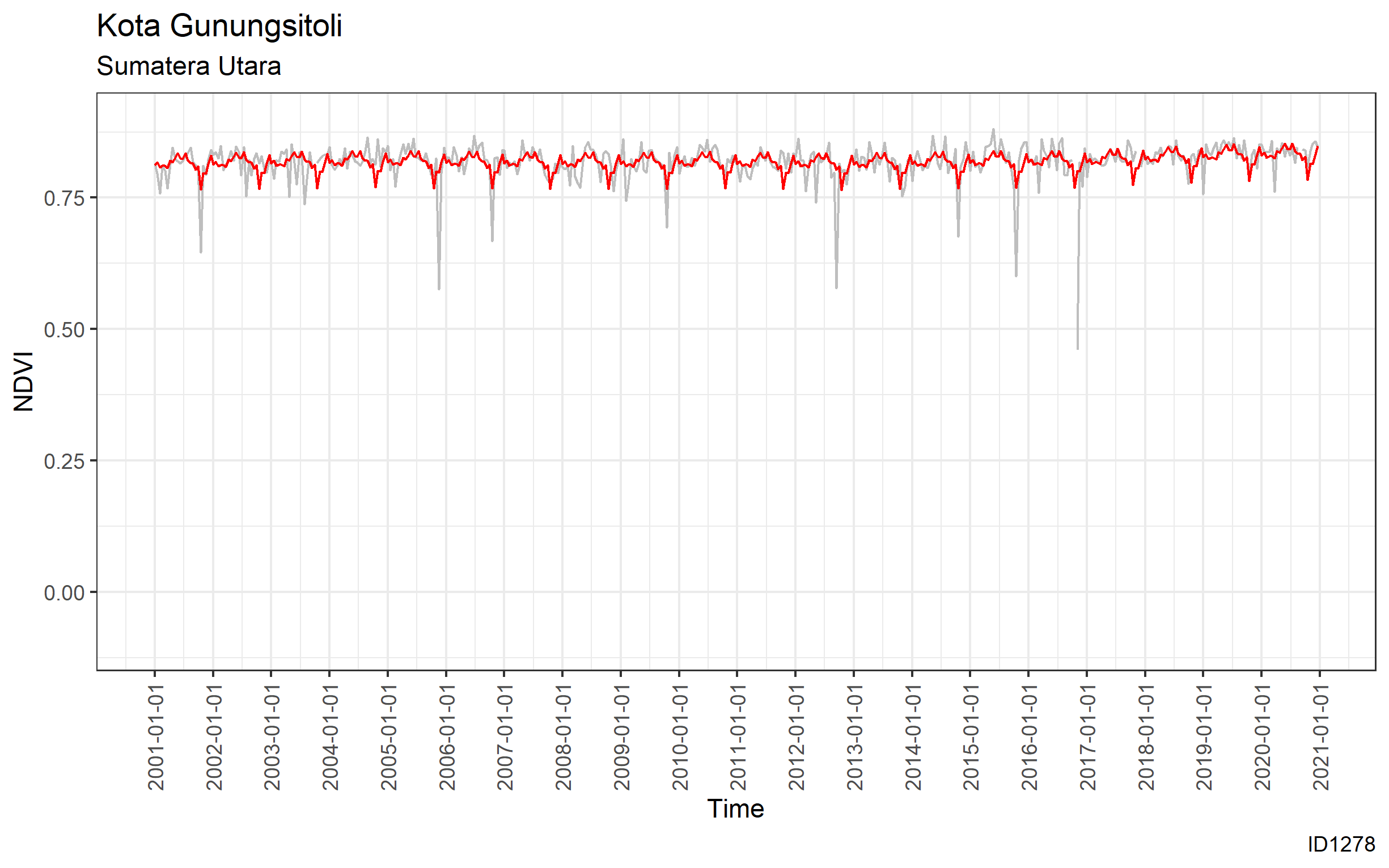


## West Sumatra Province


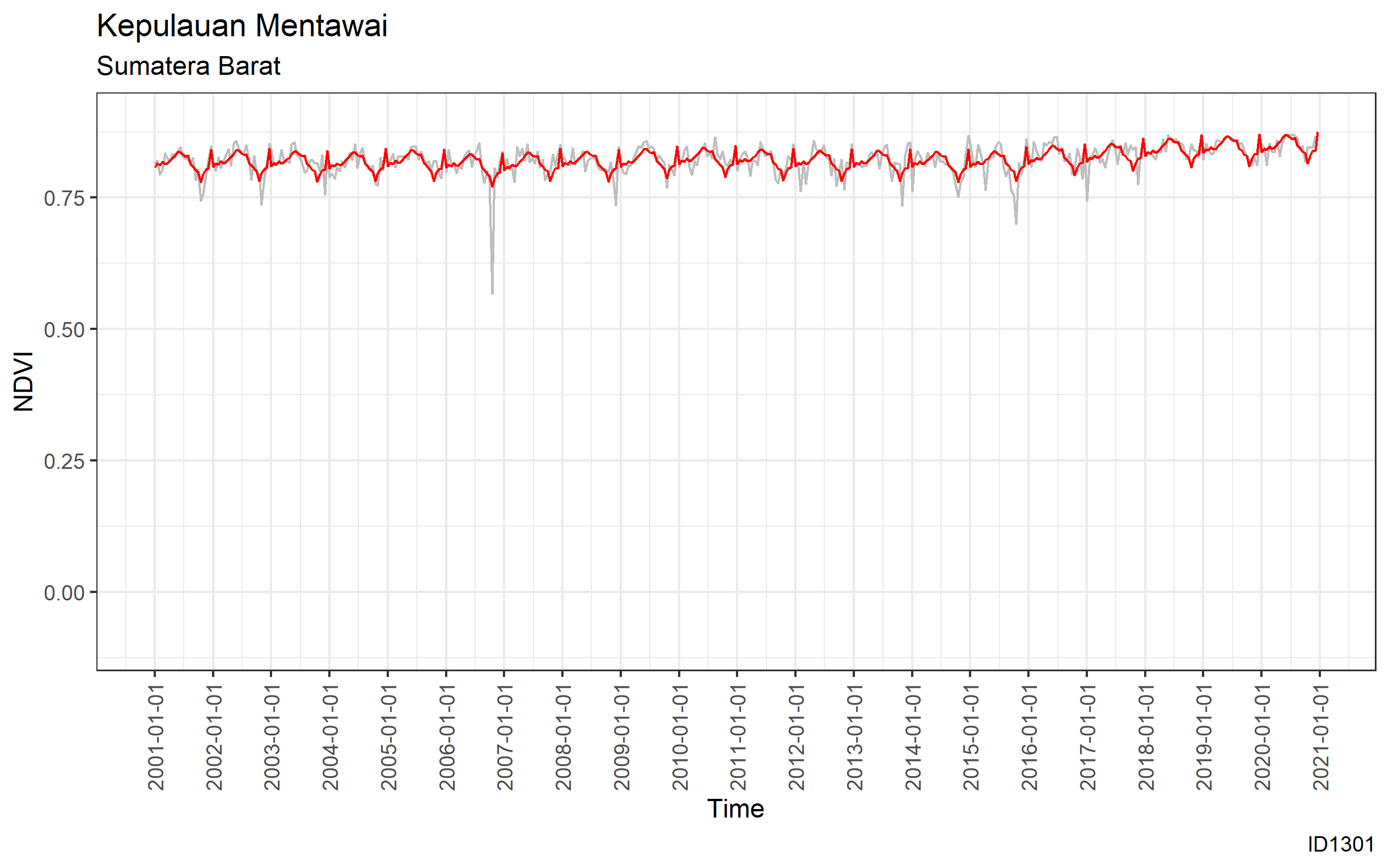

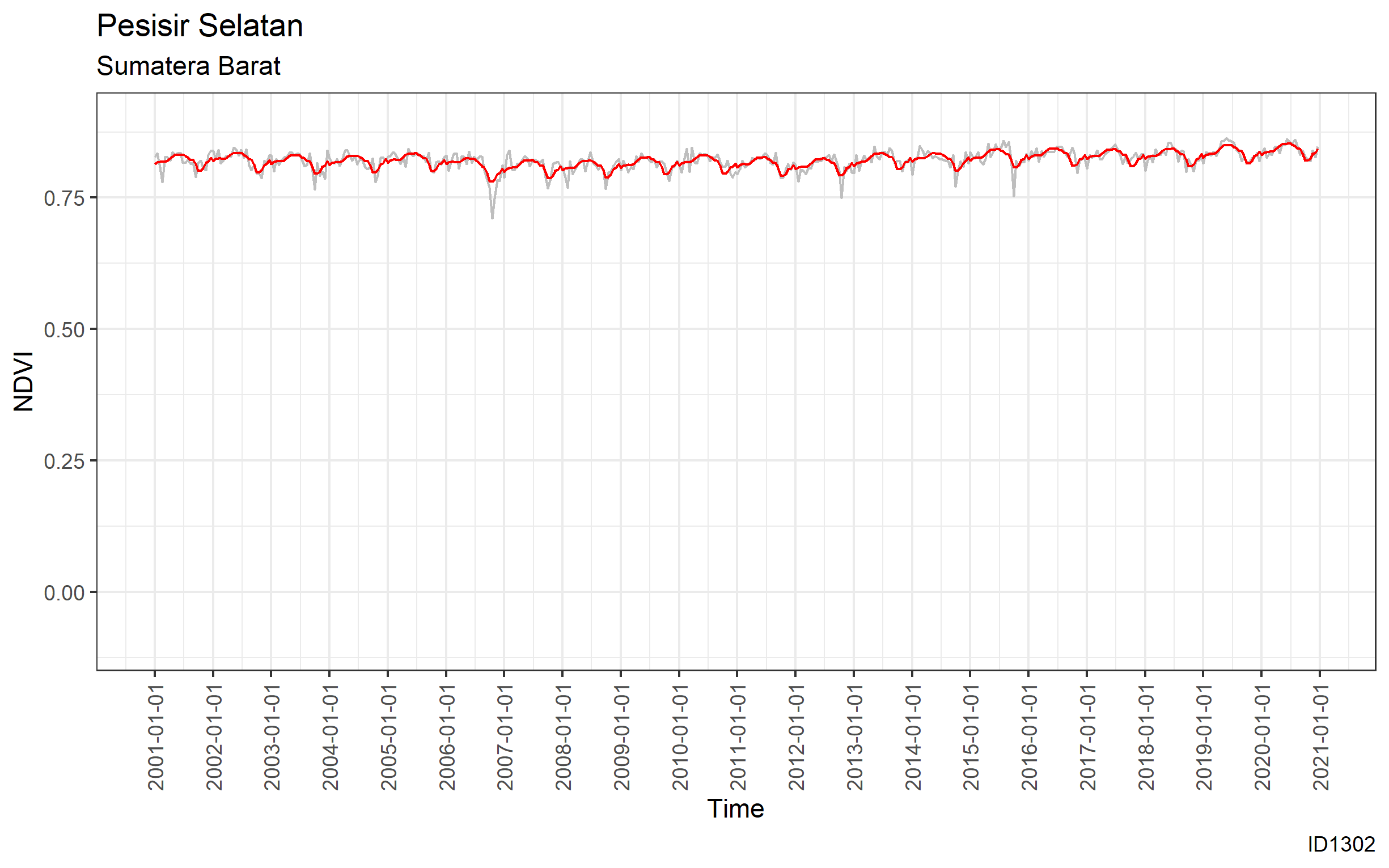

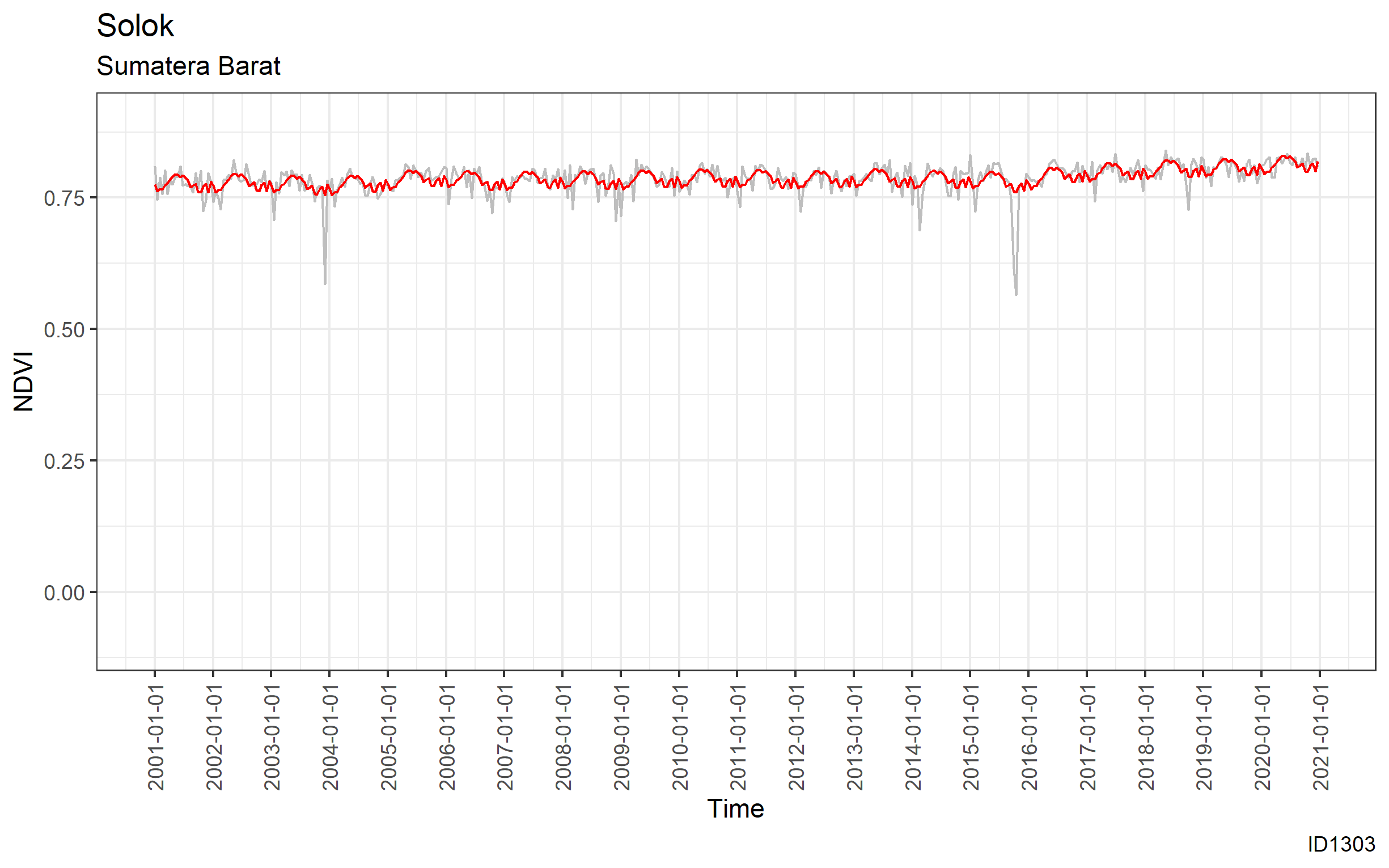

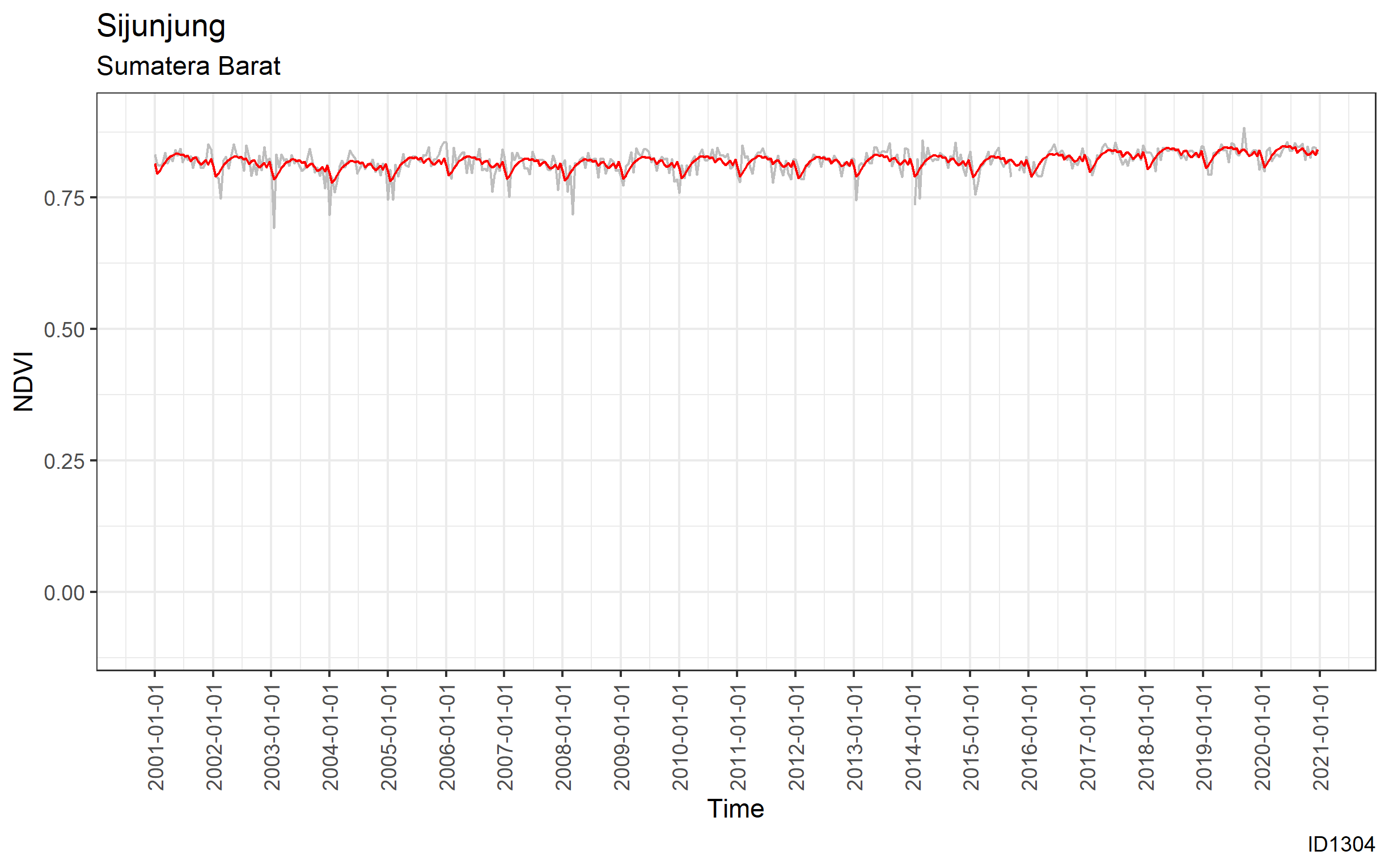


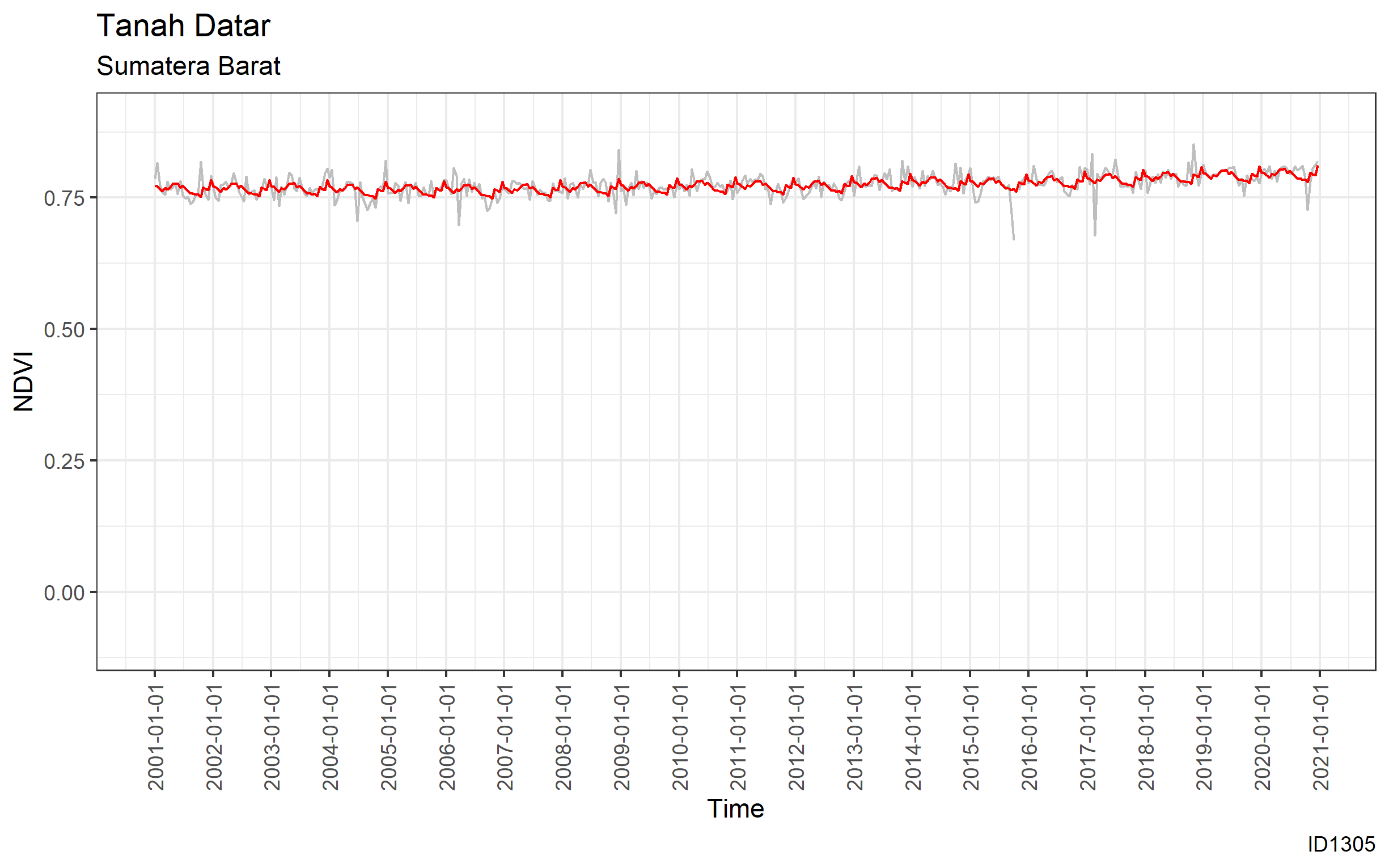

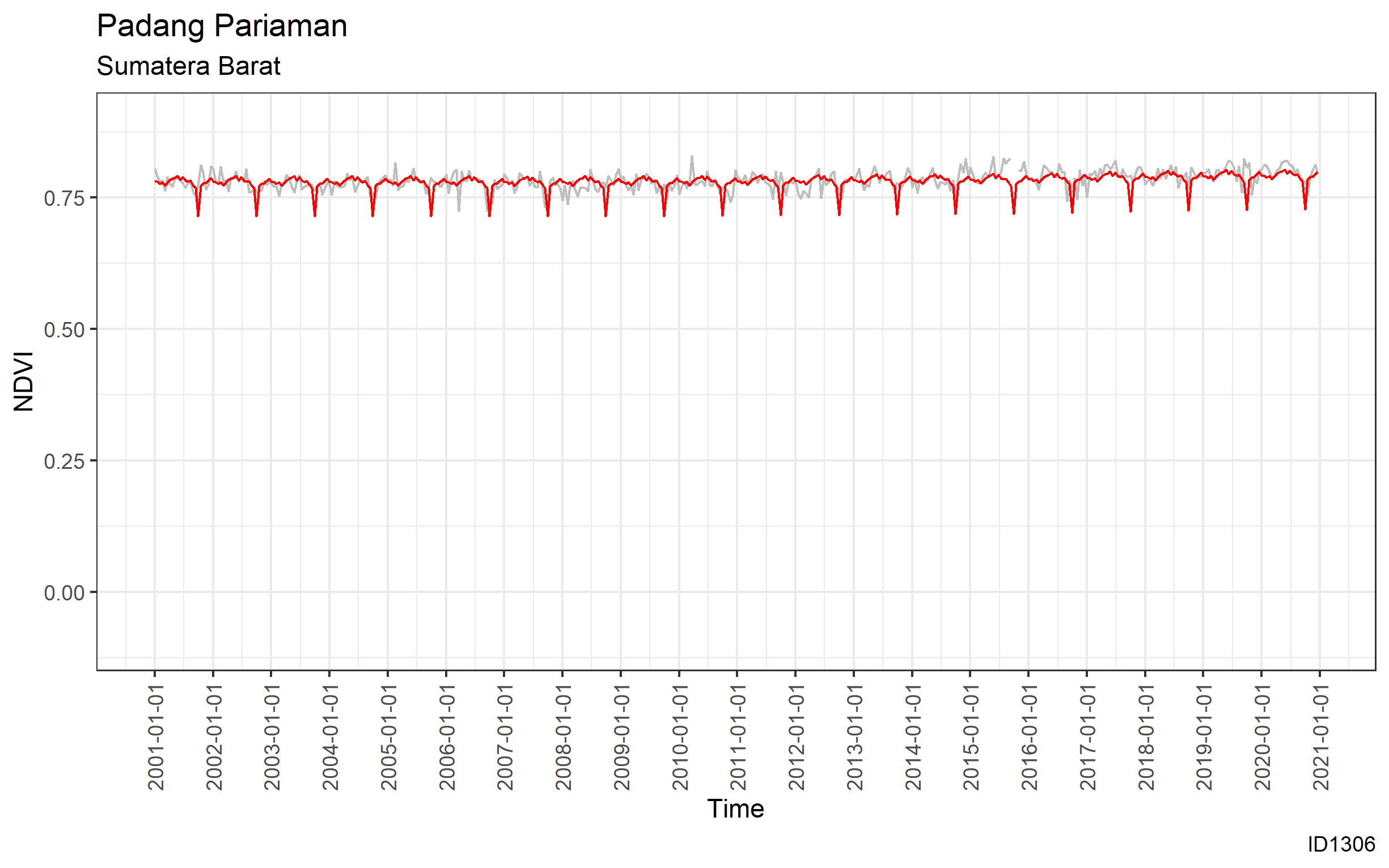

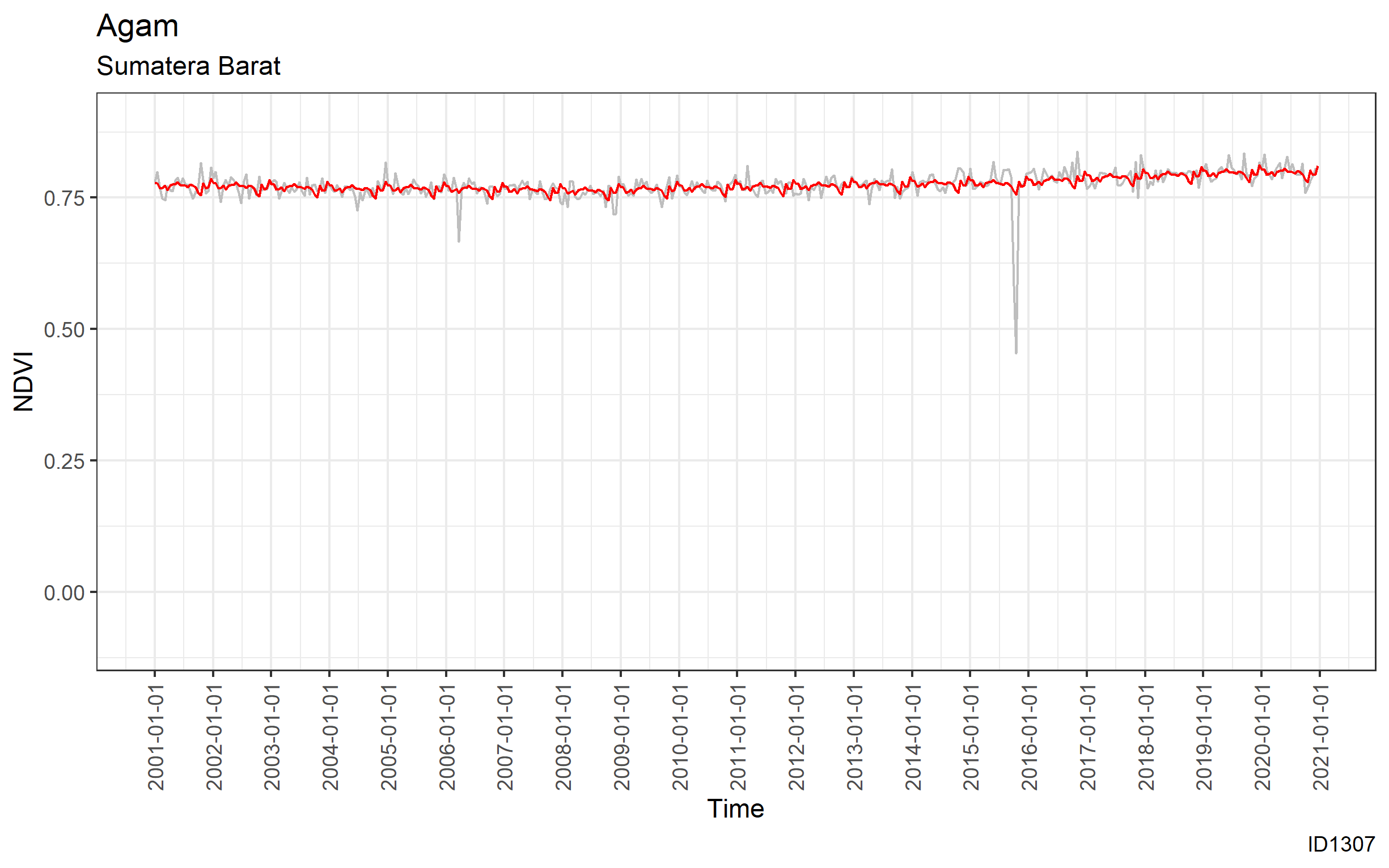

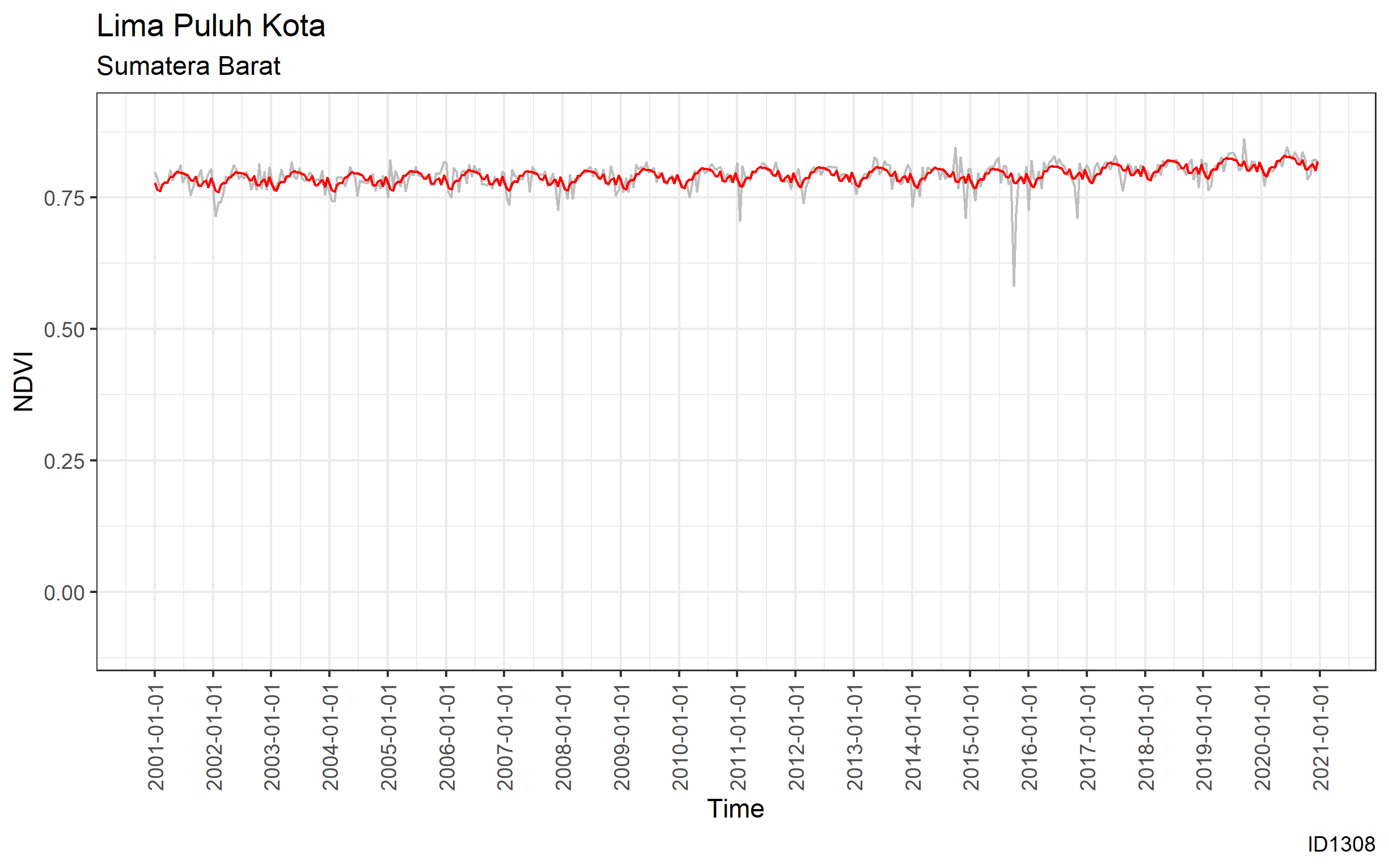

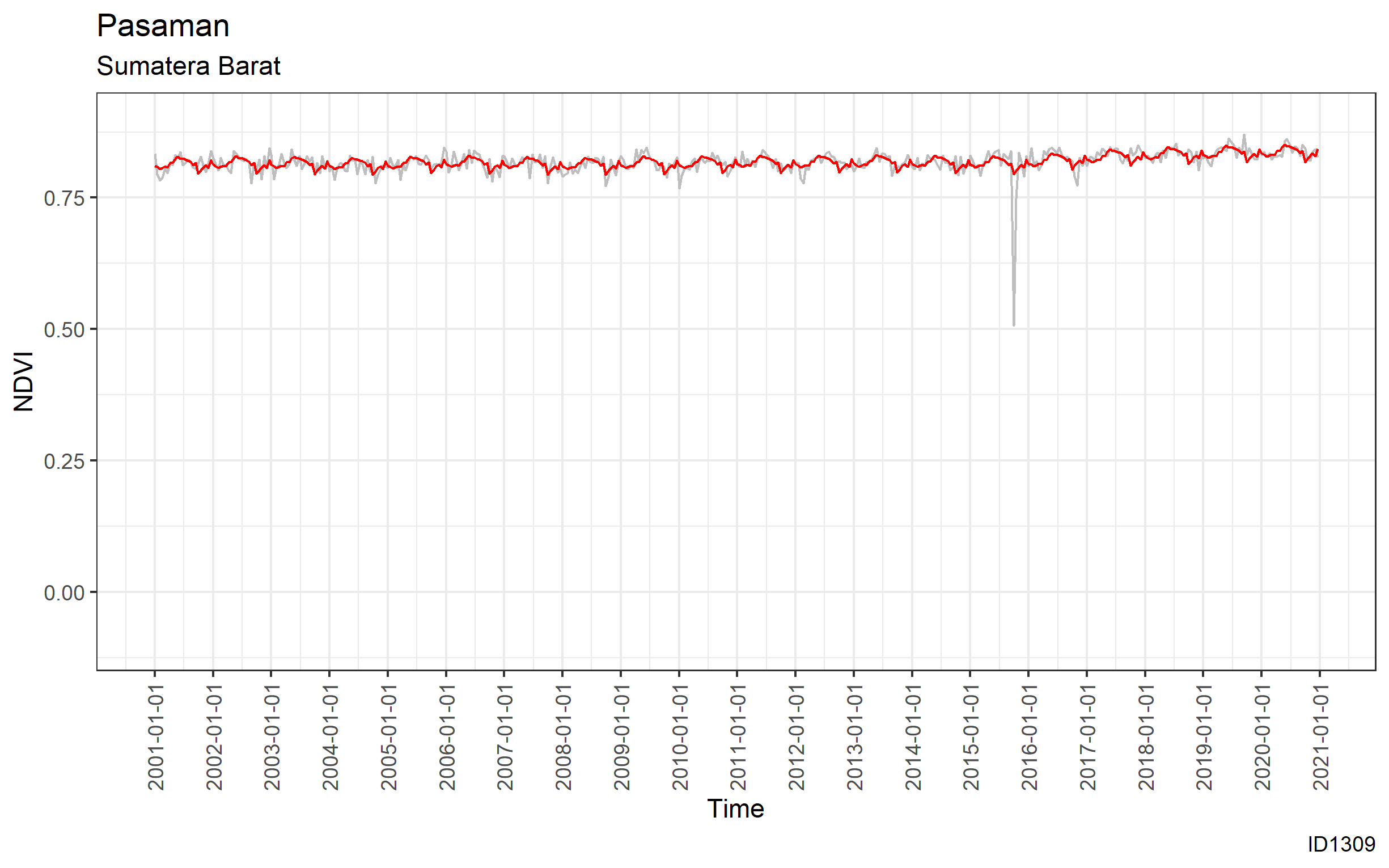

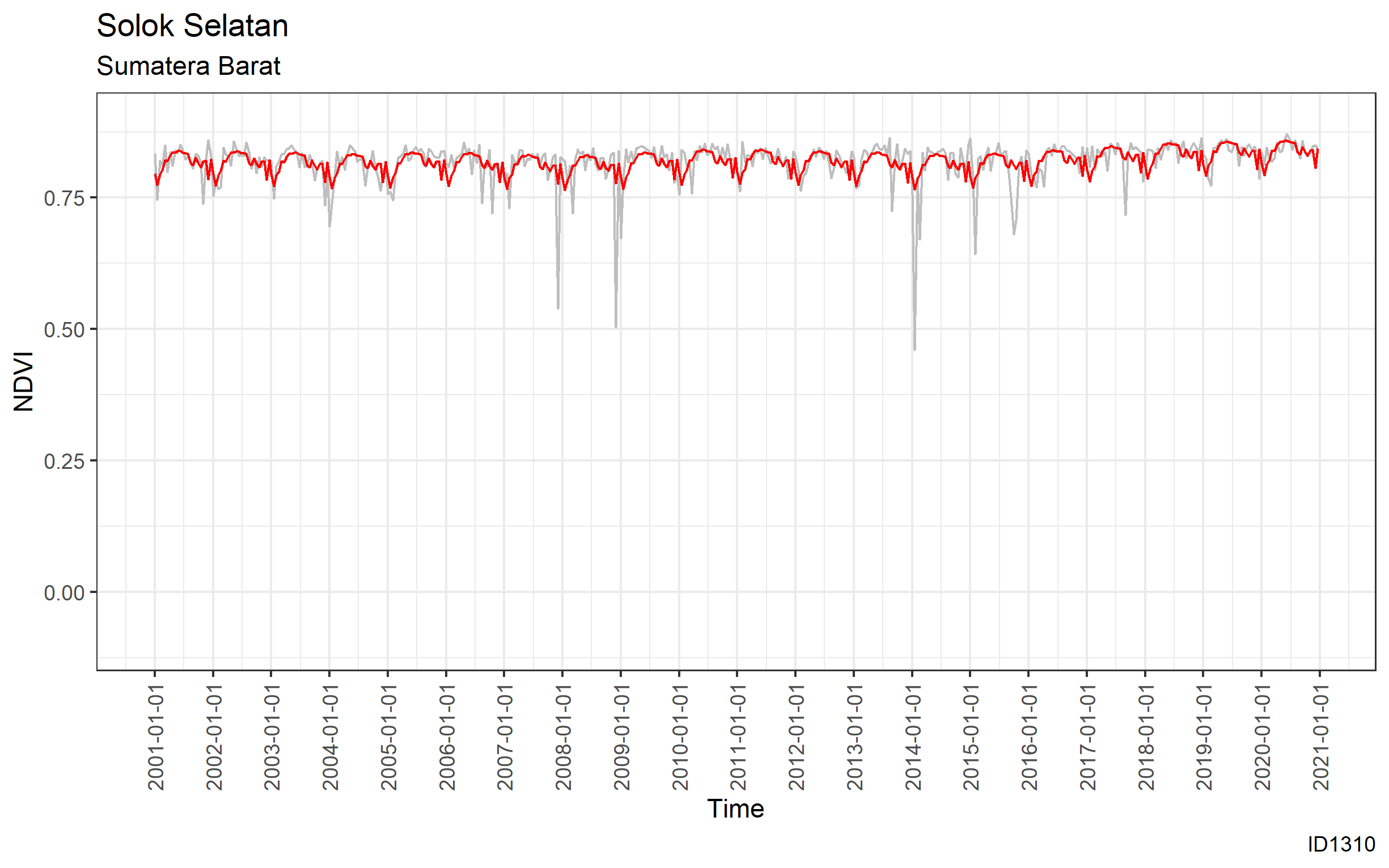

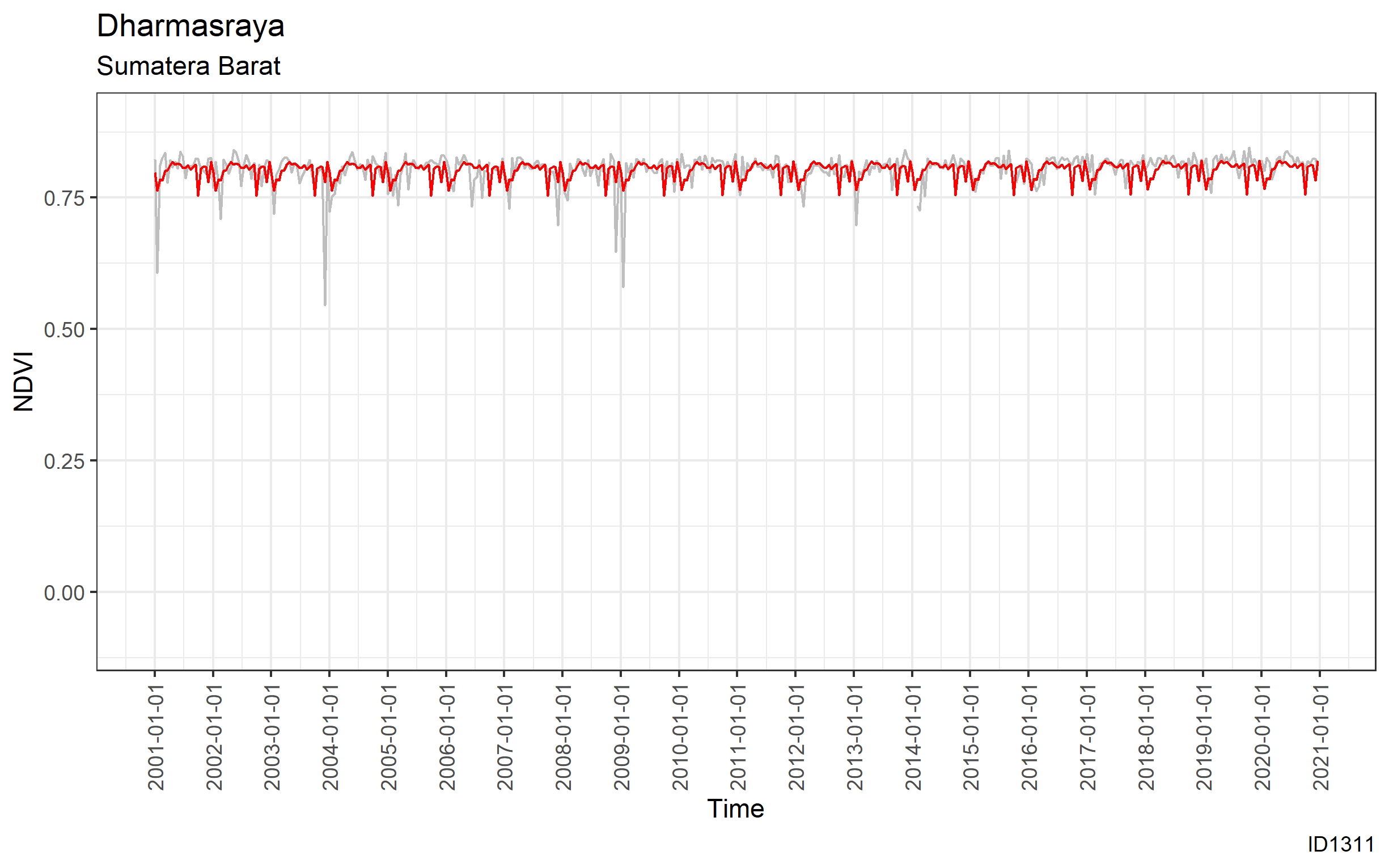

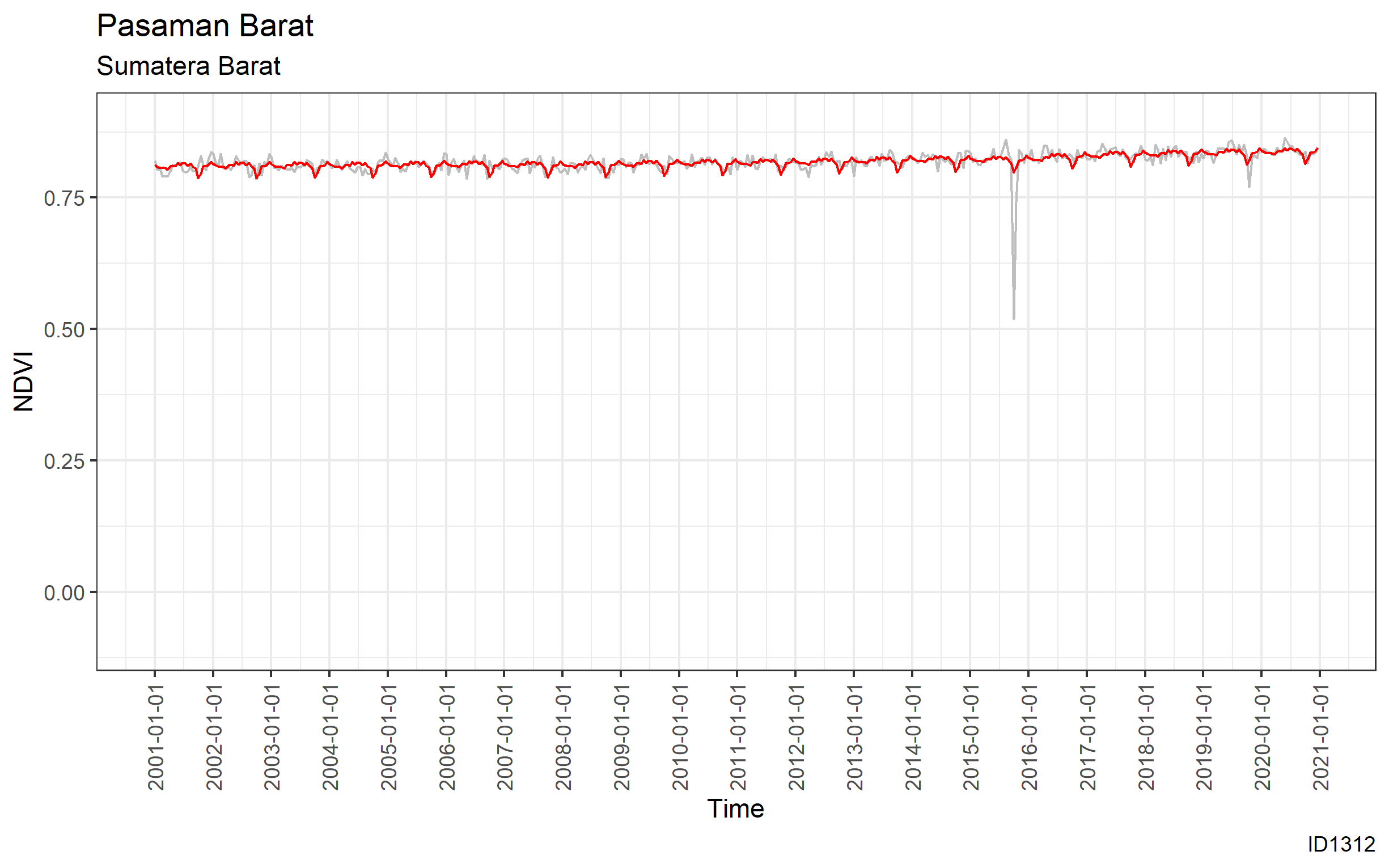

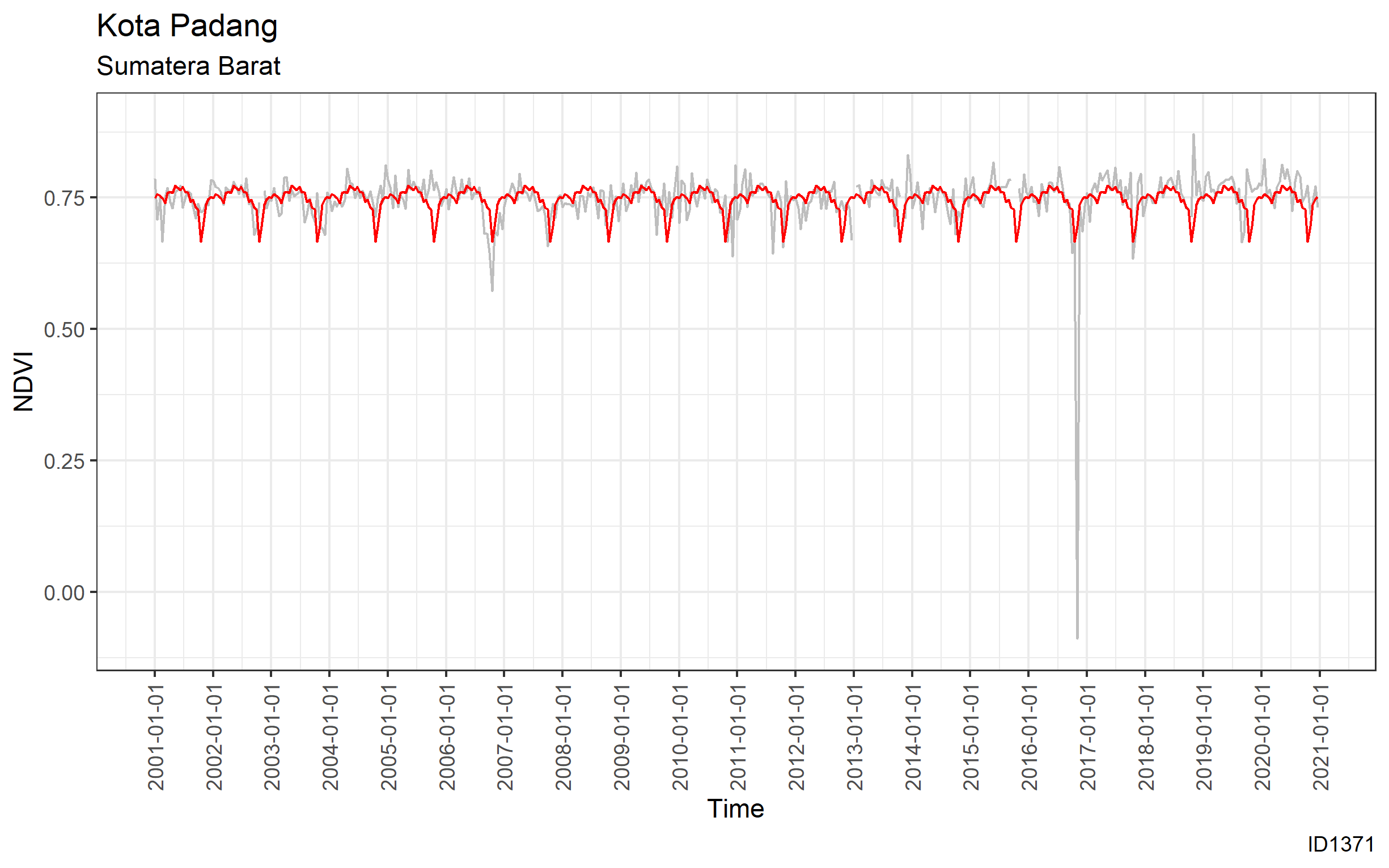

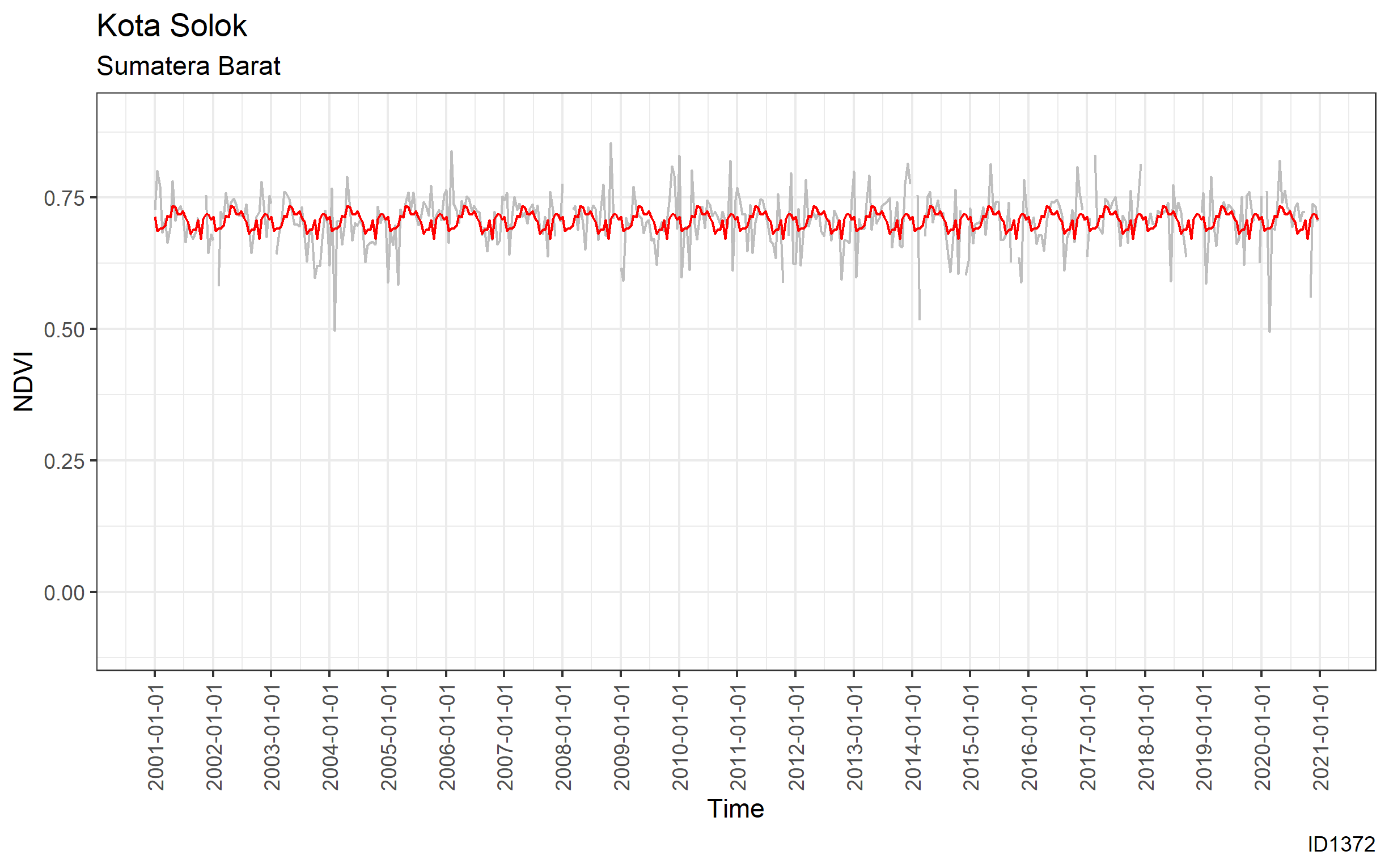

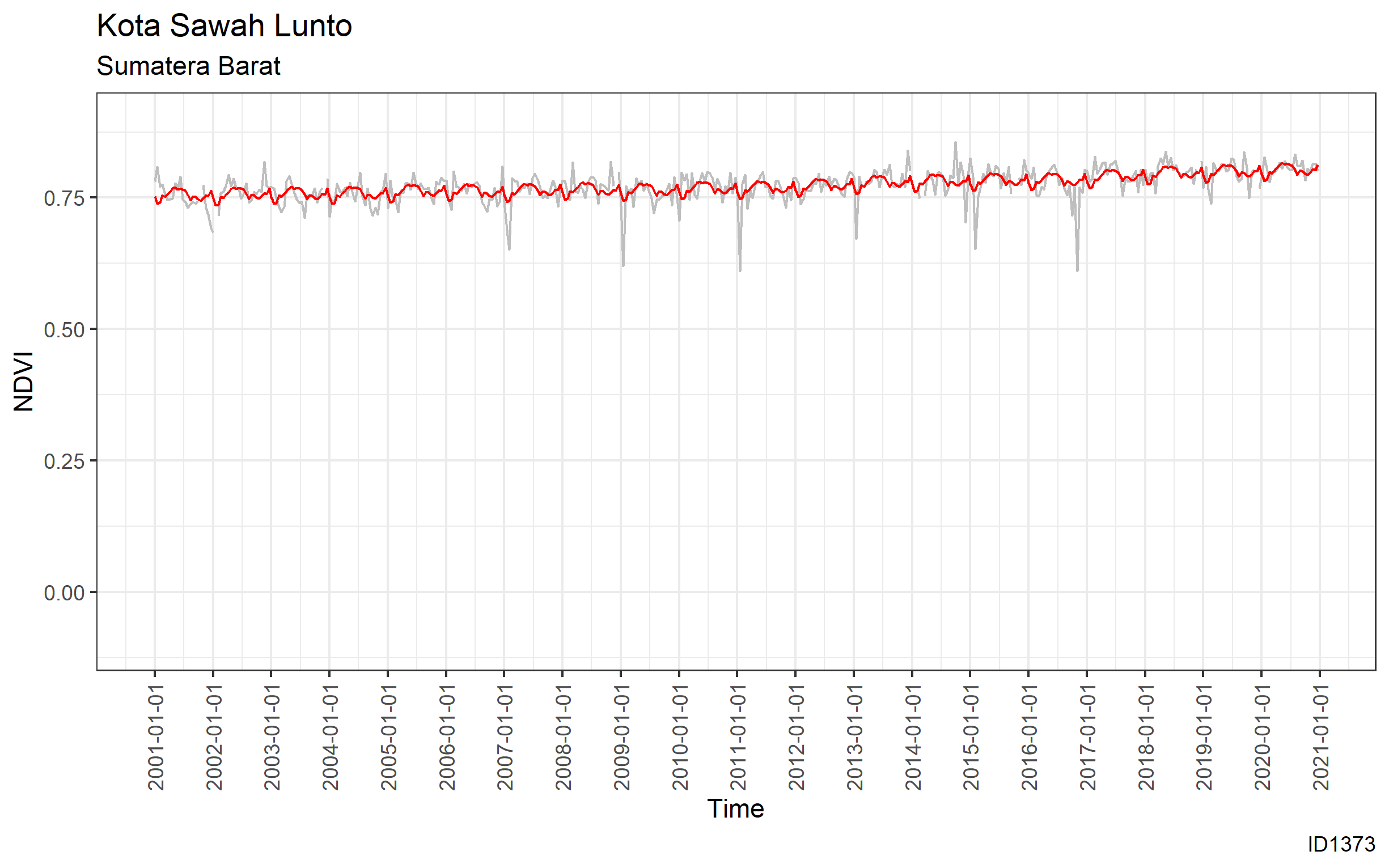

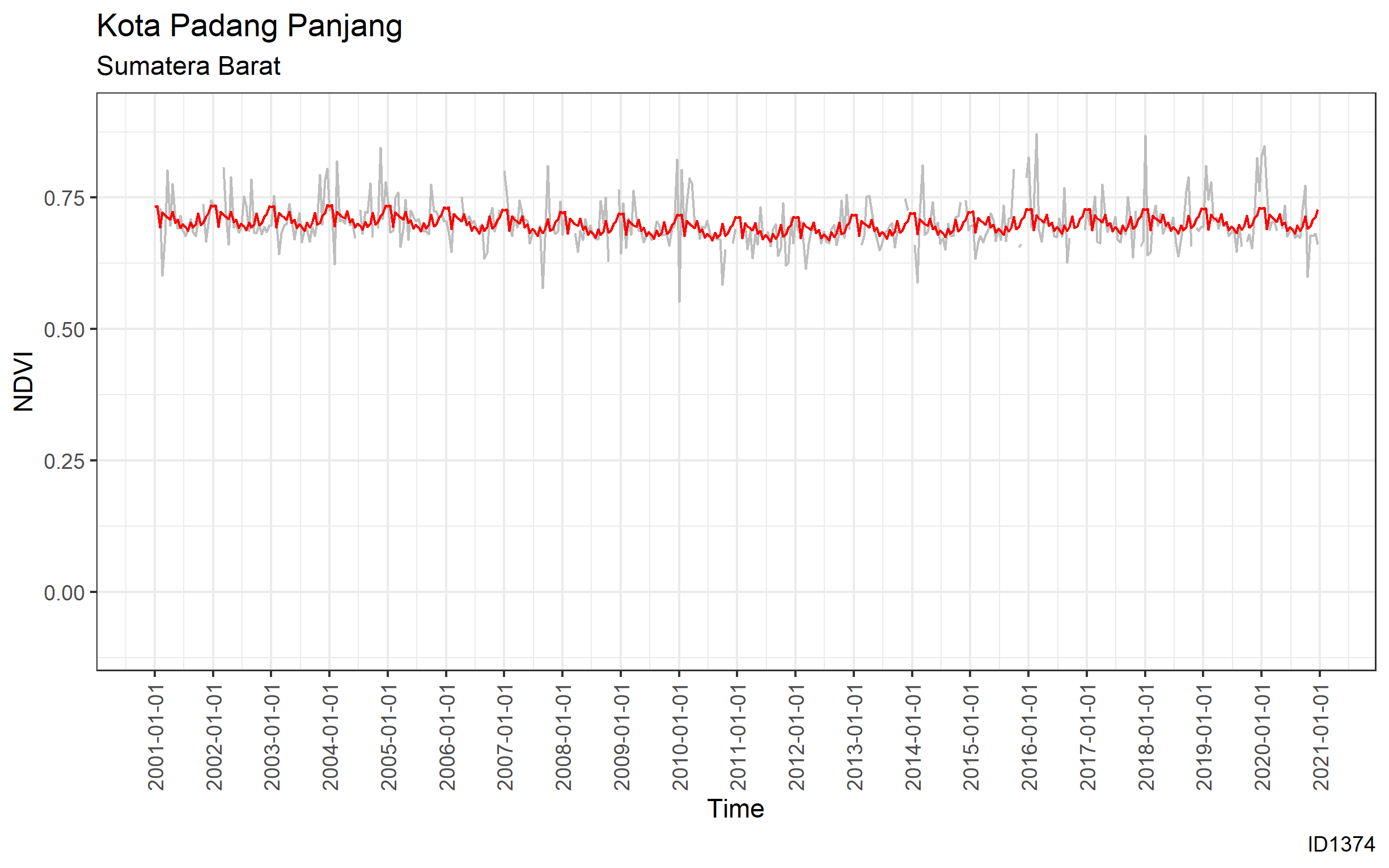

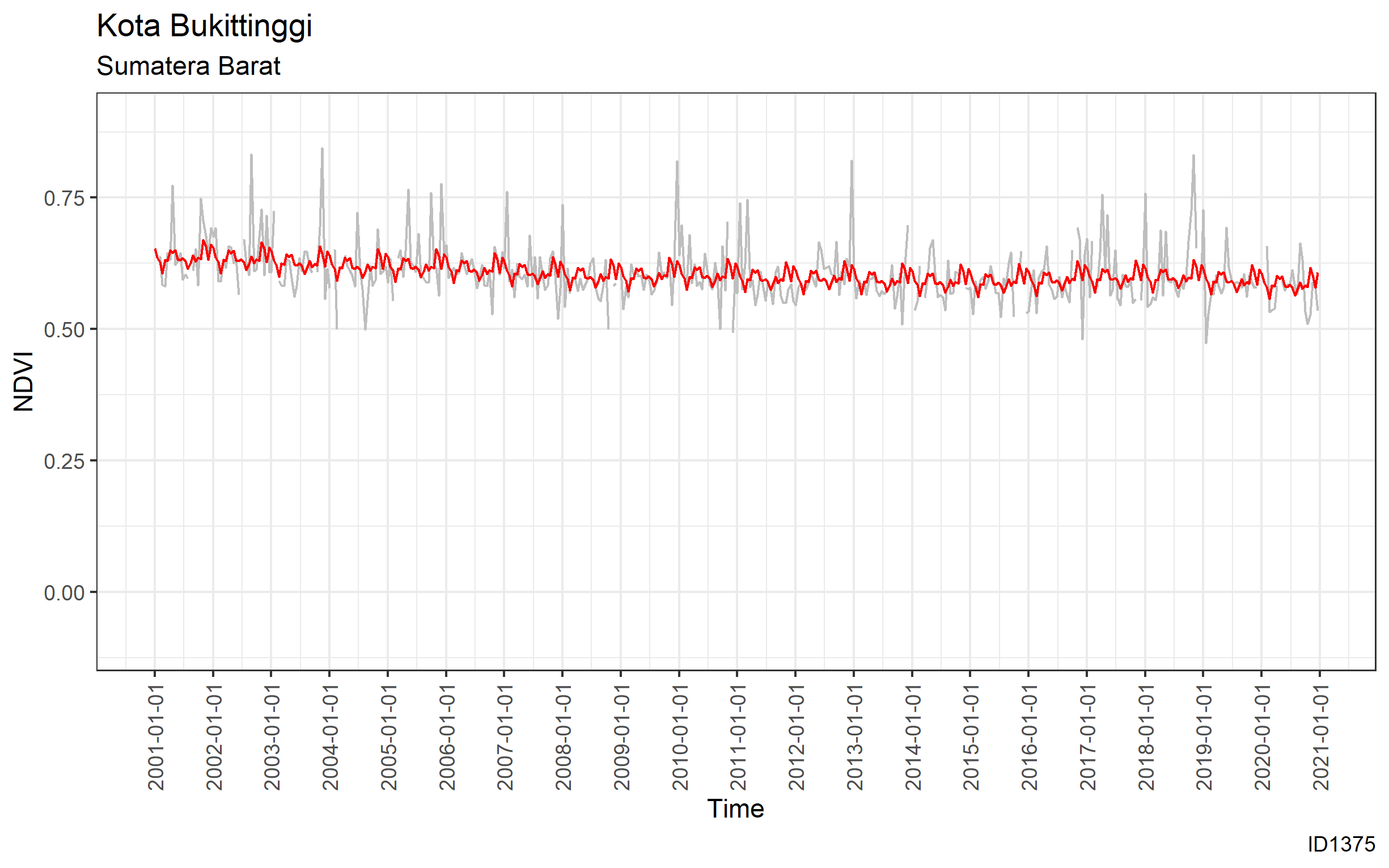

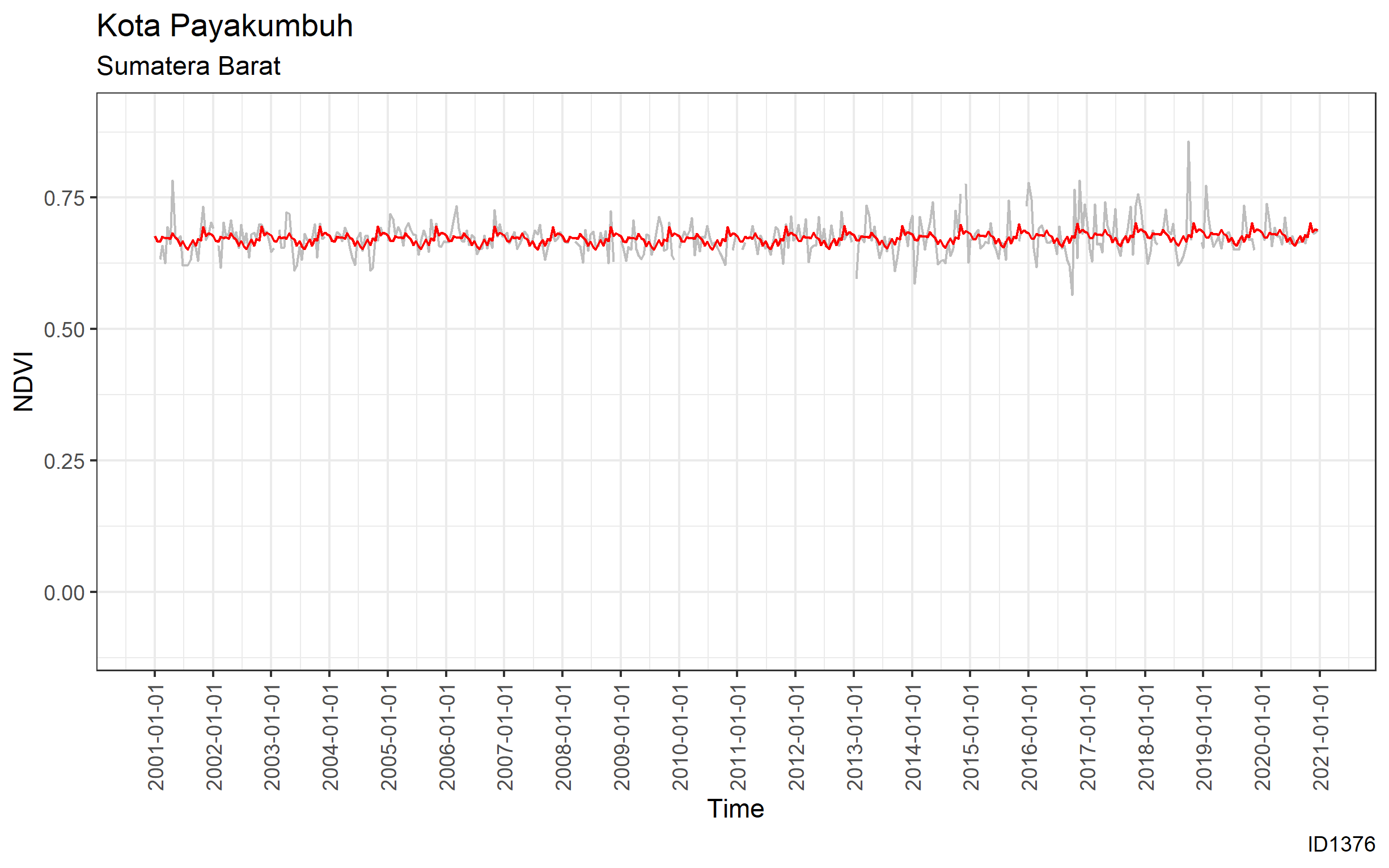

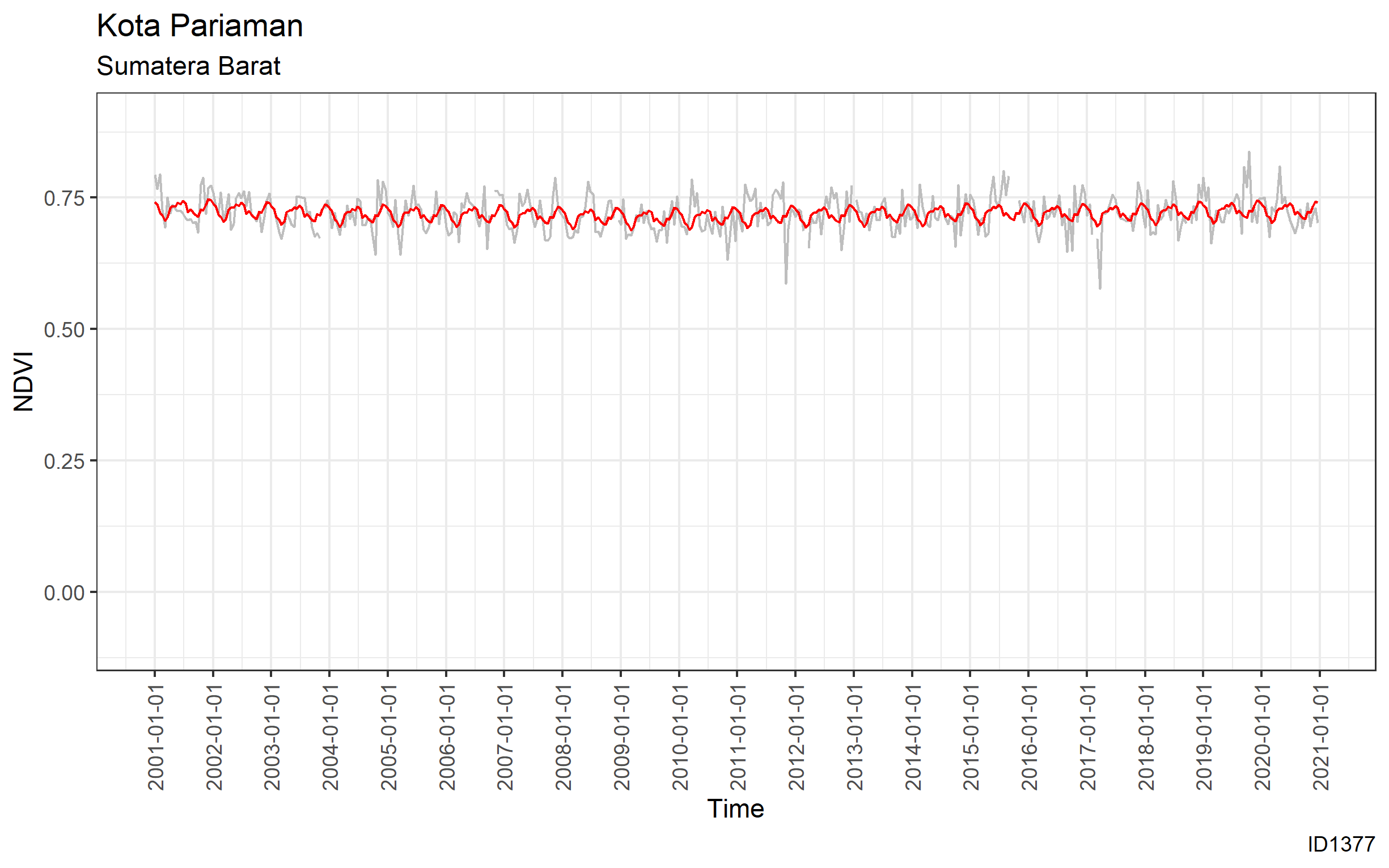


## Riau Province


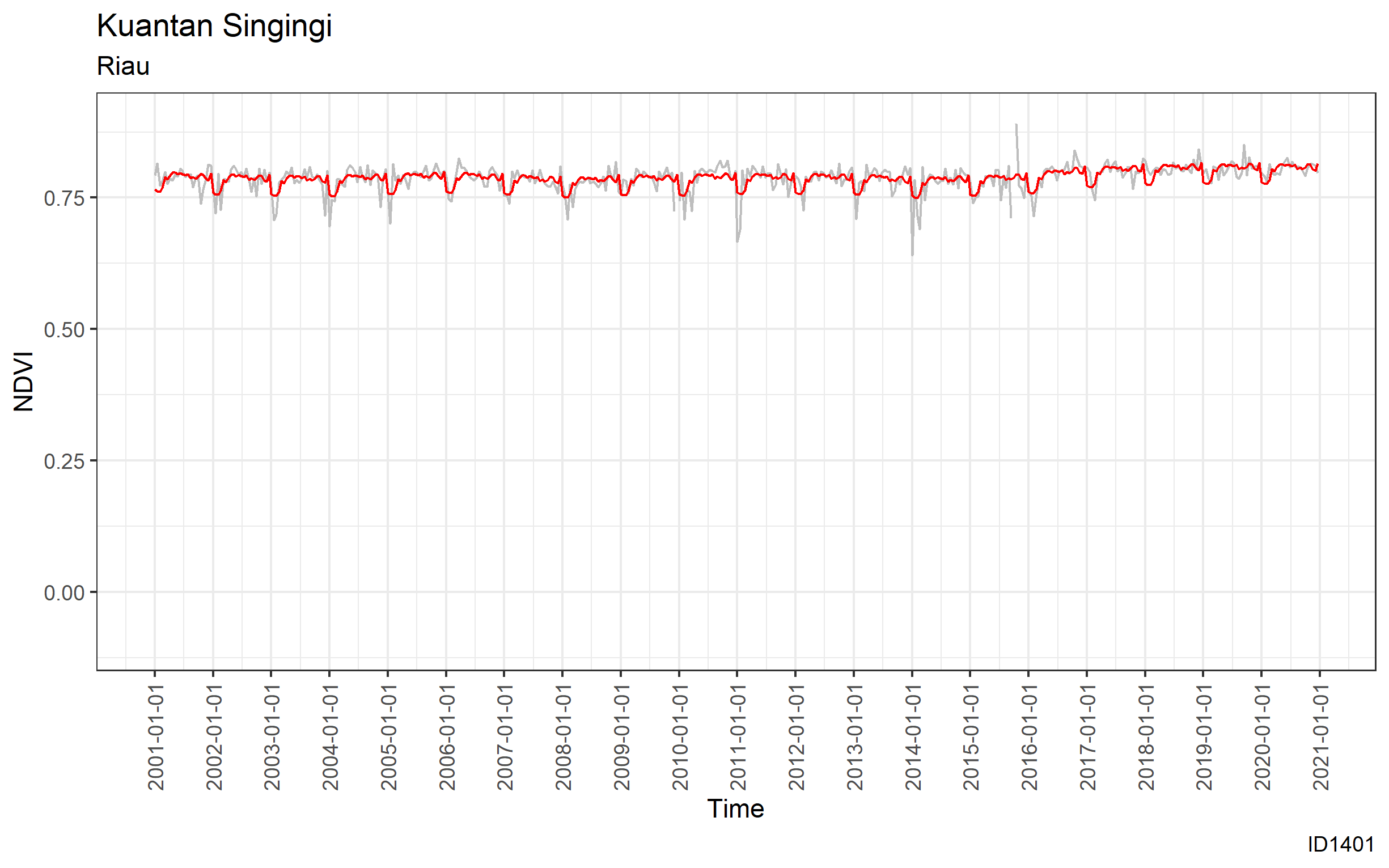

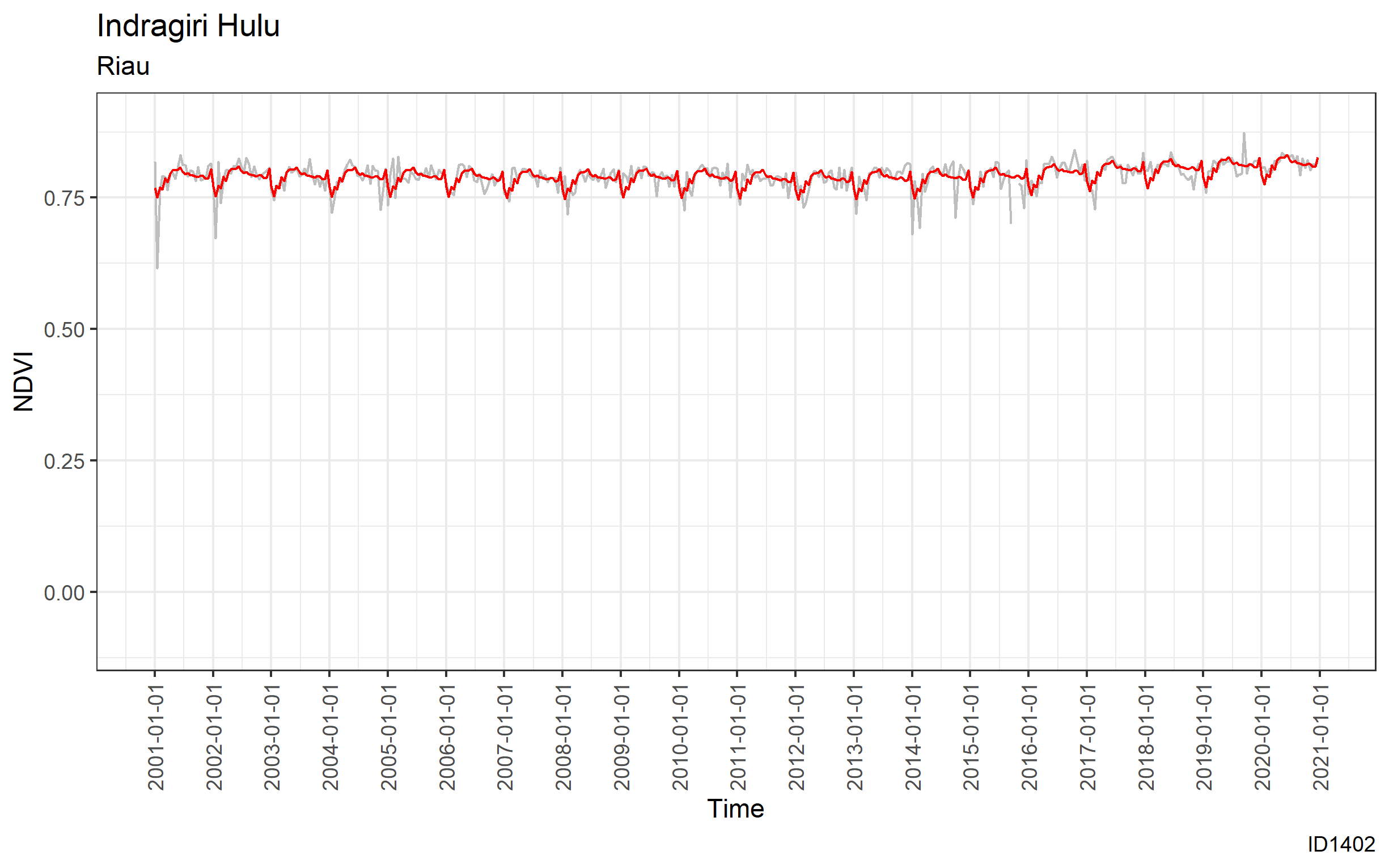

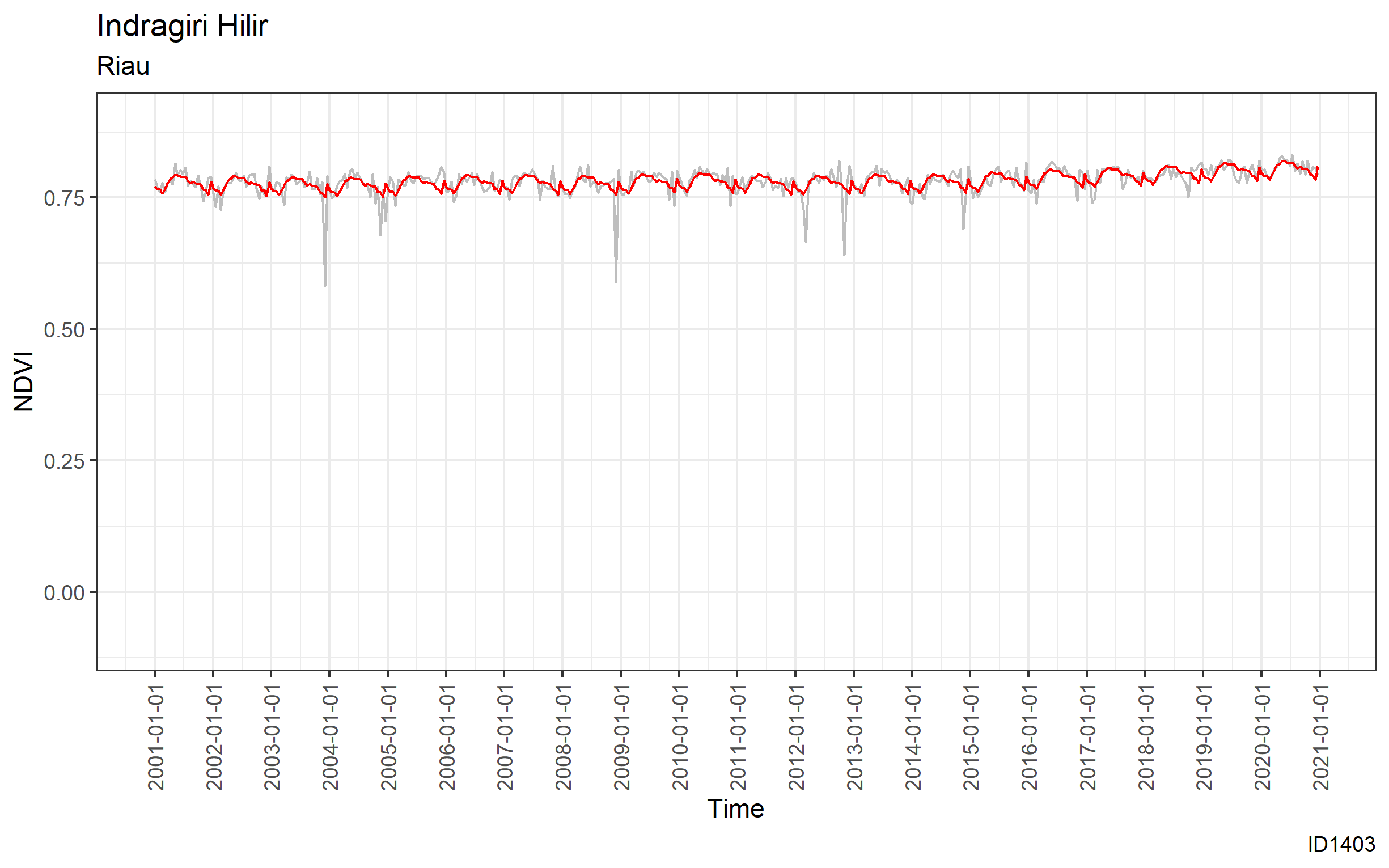

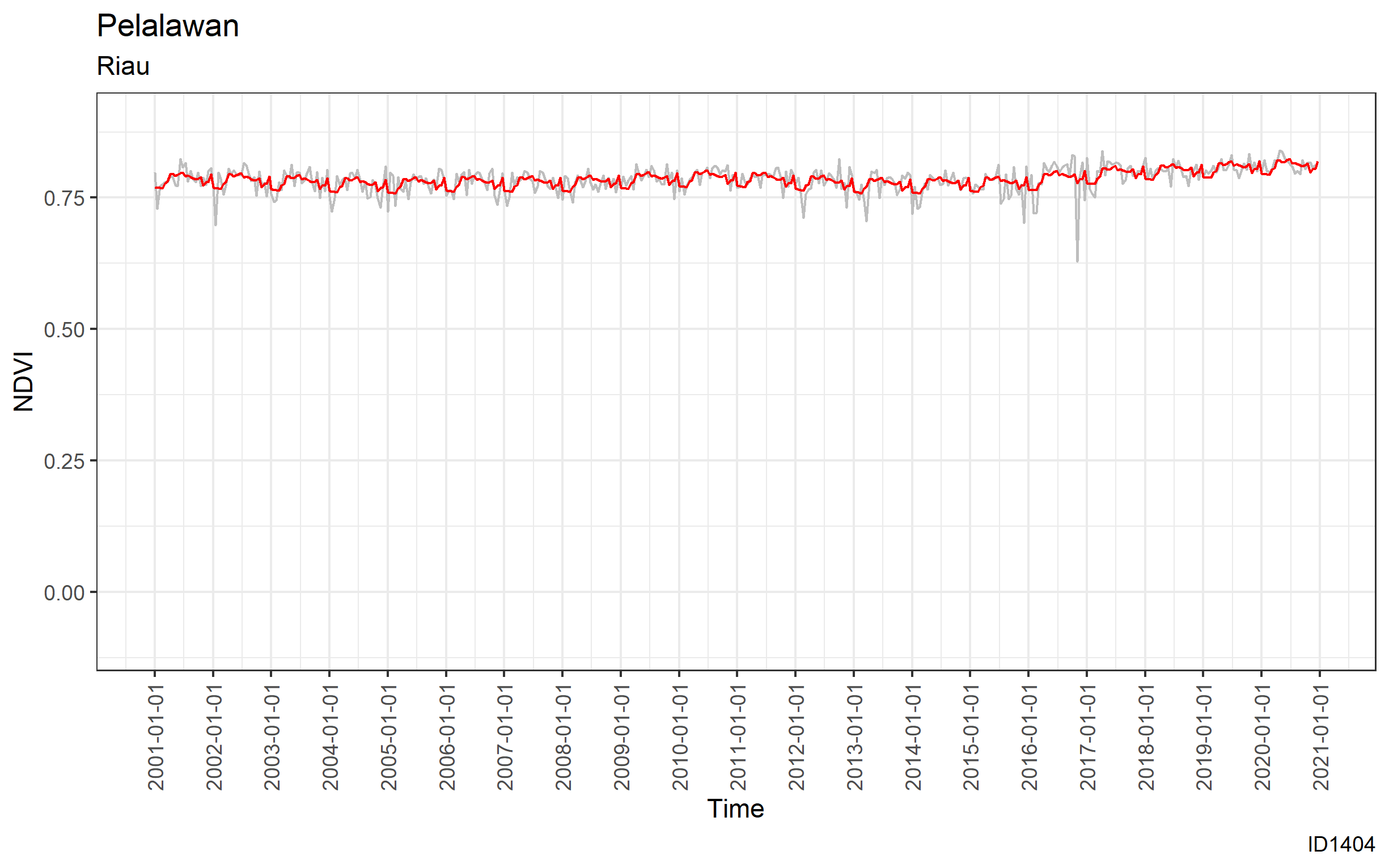


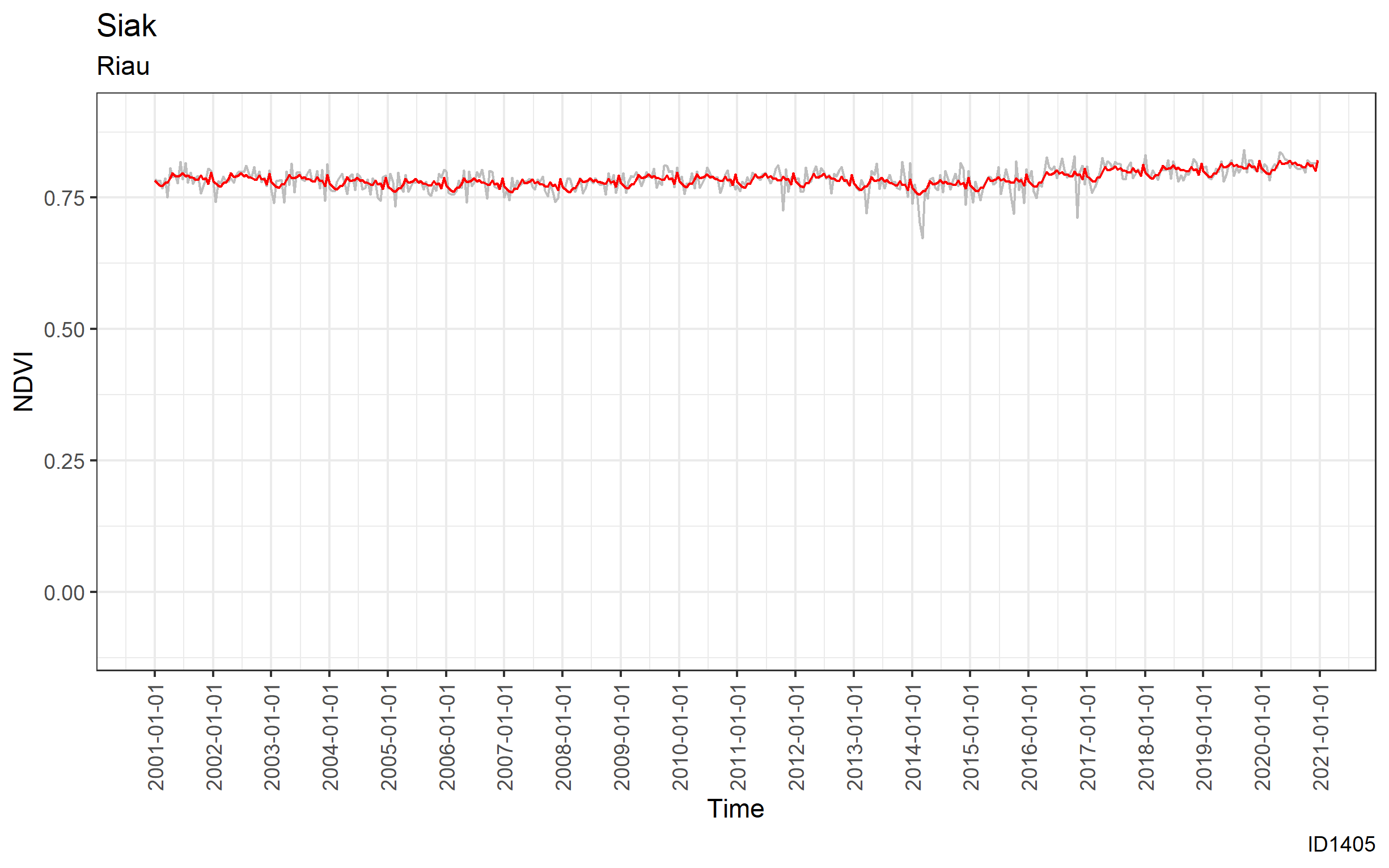

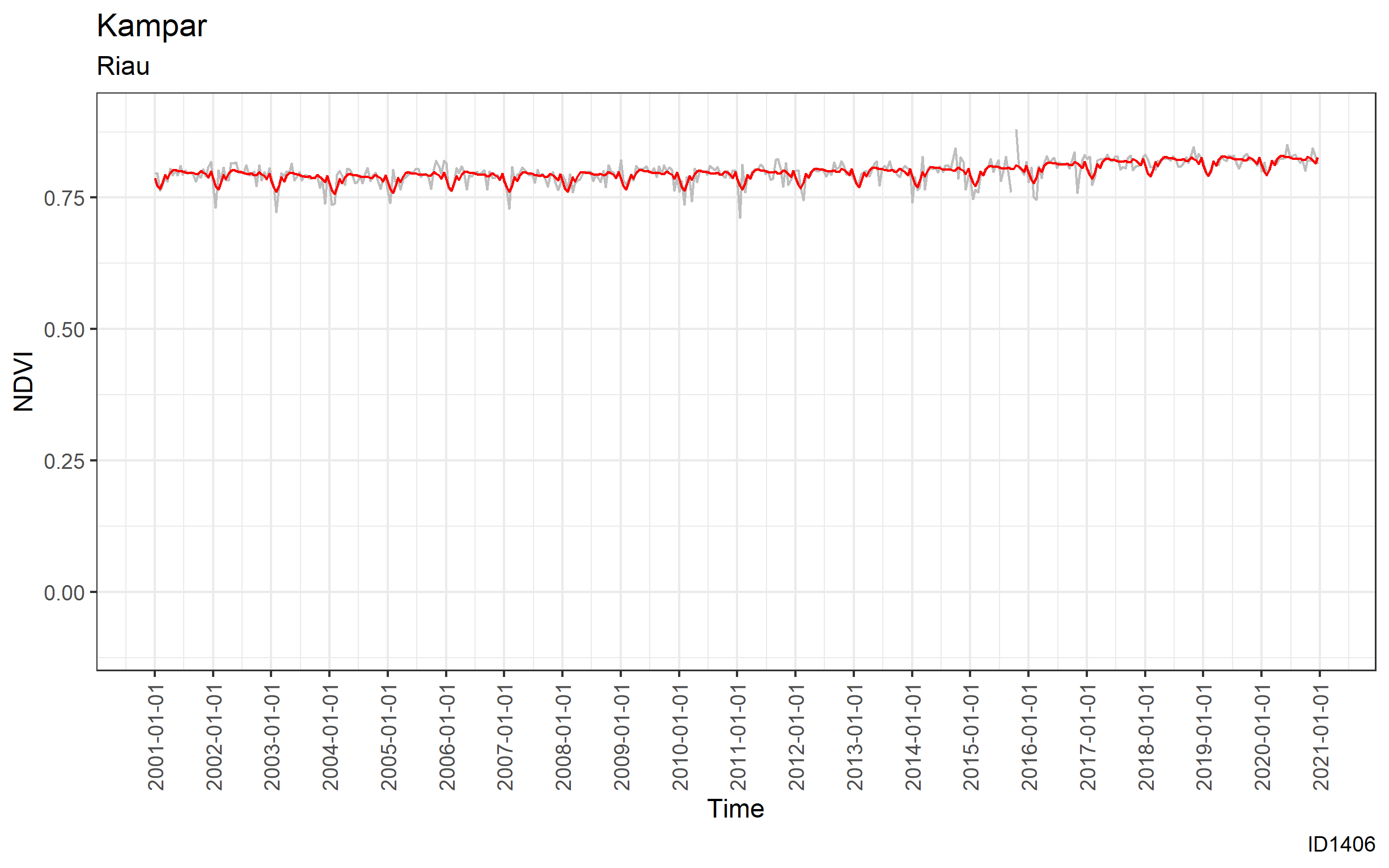

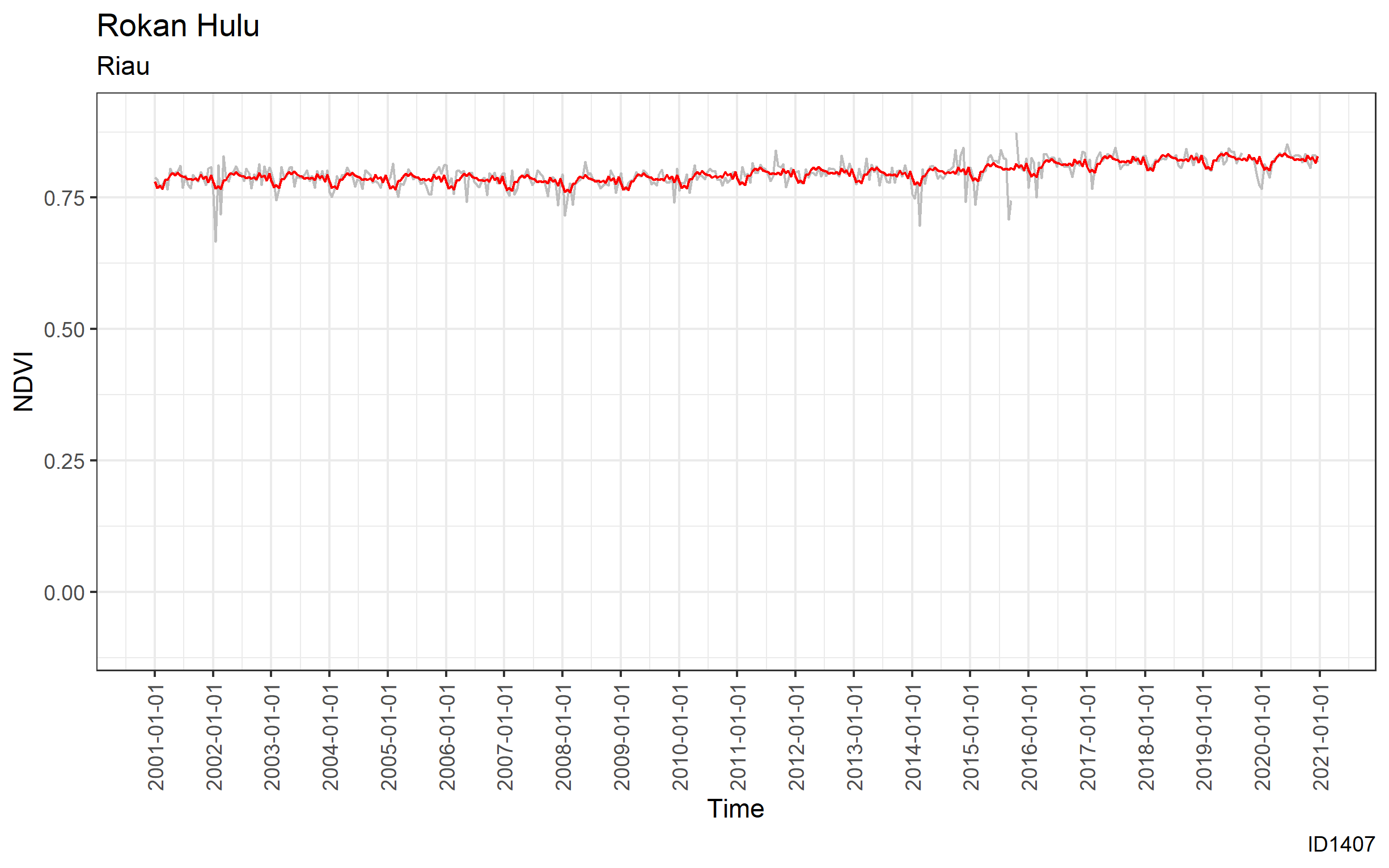

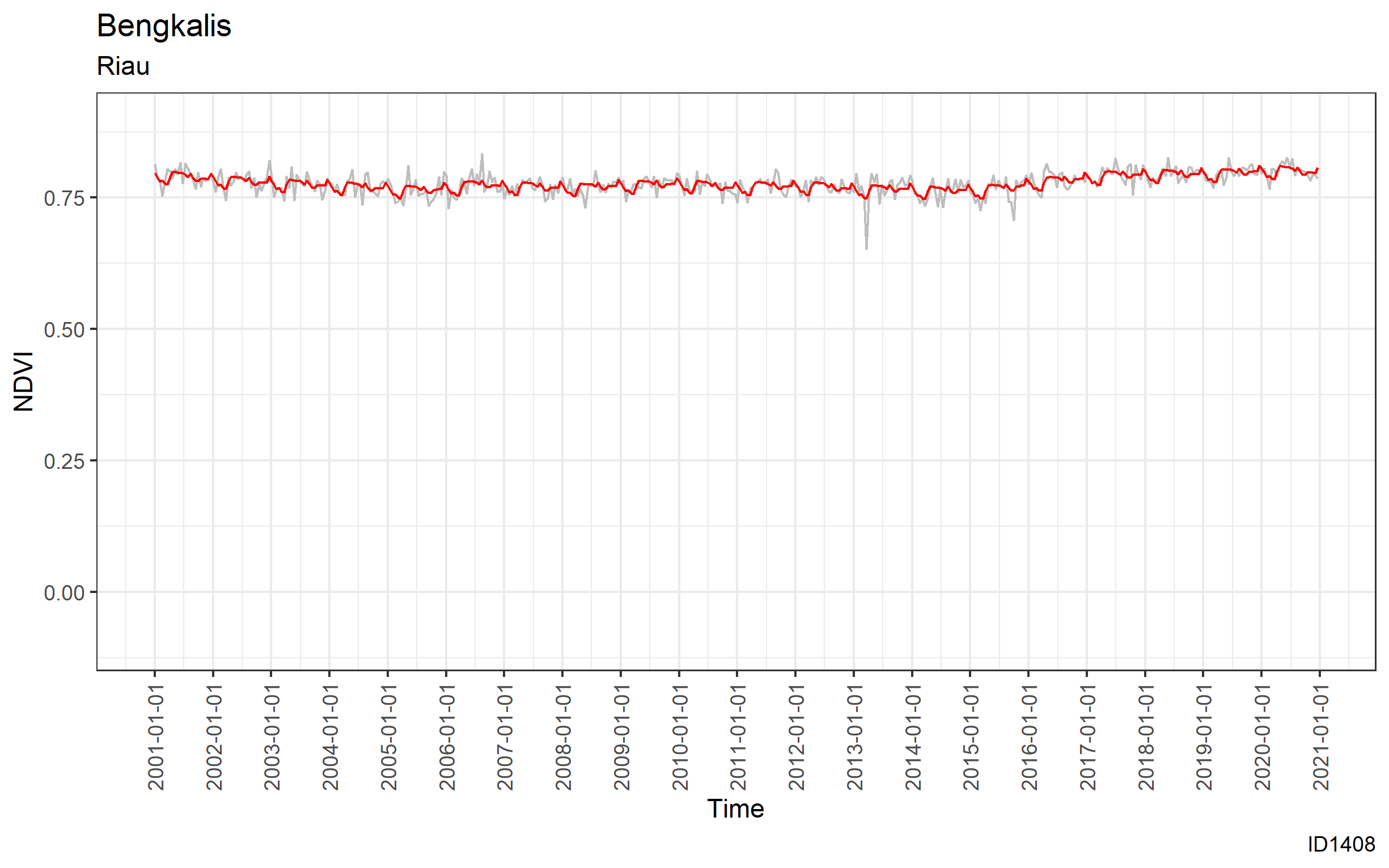

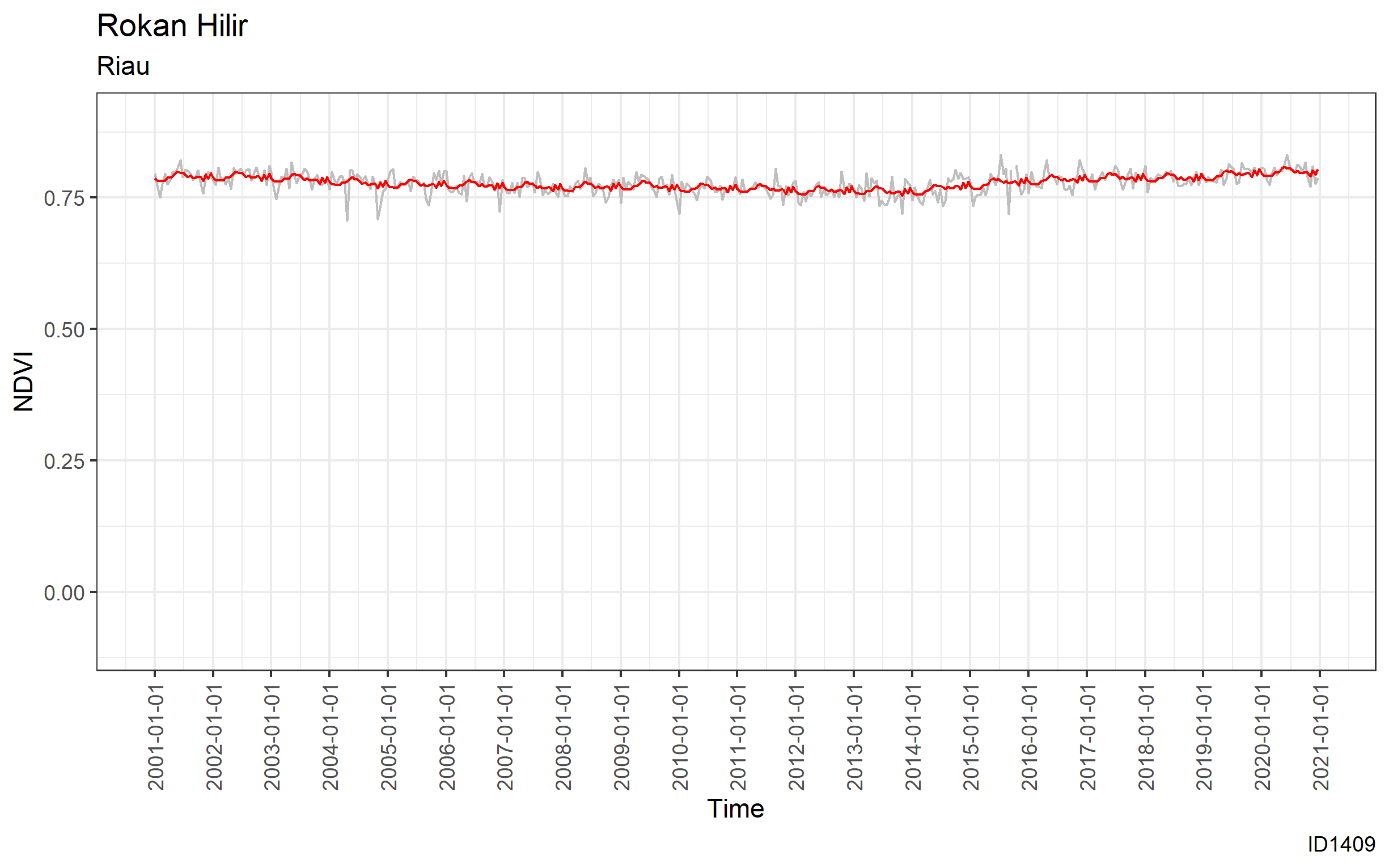

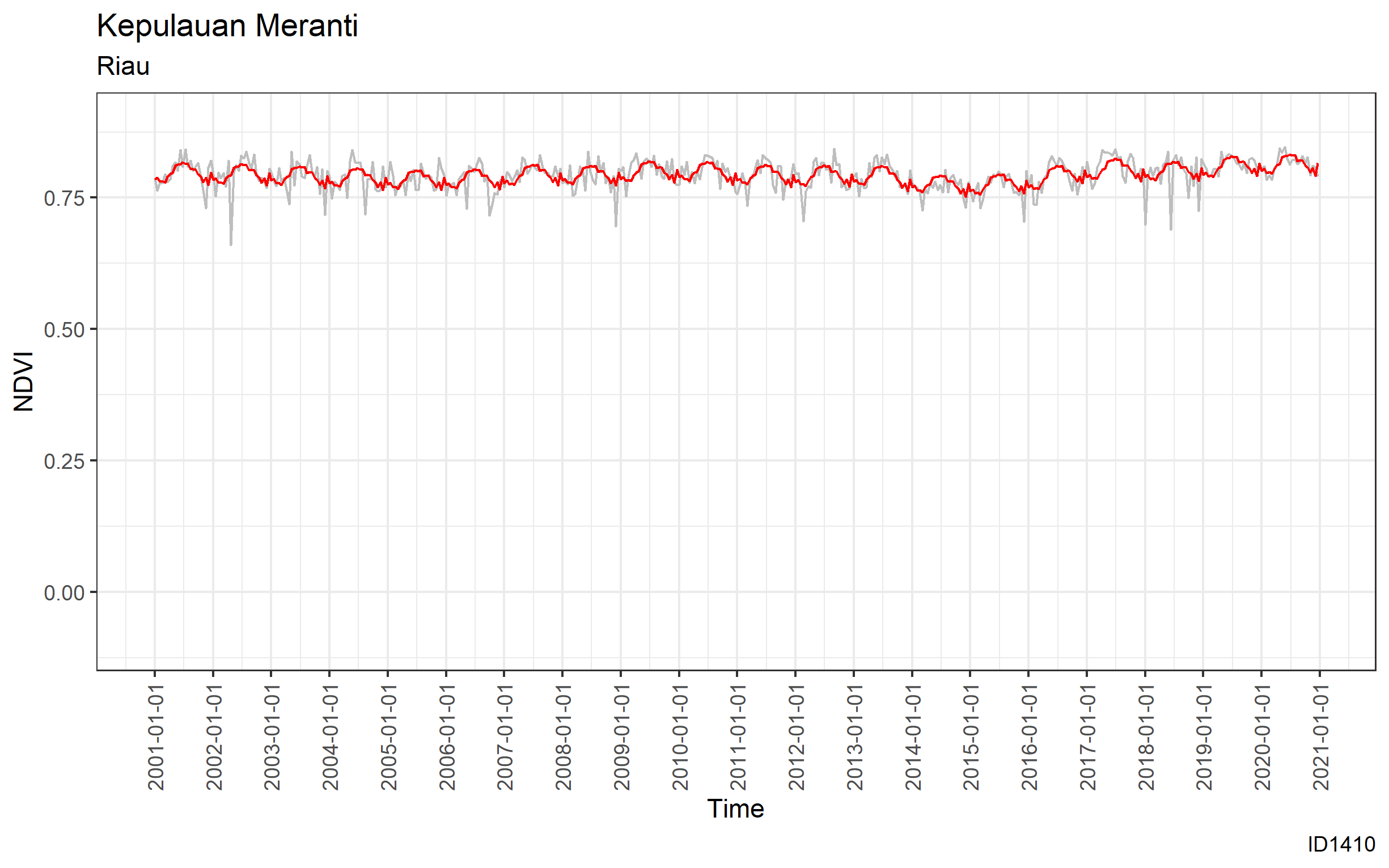

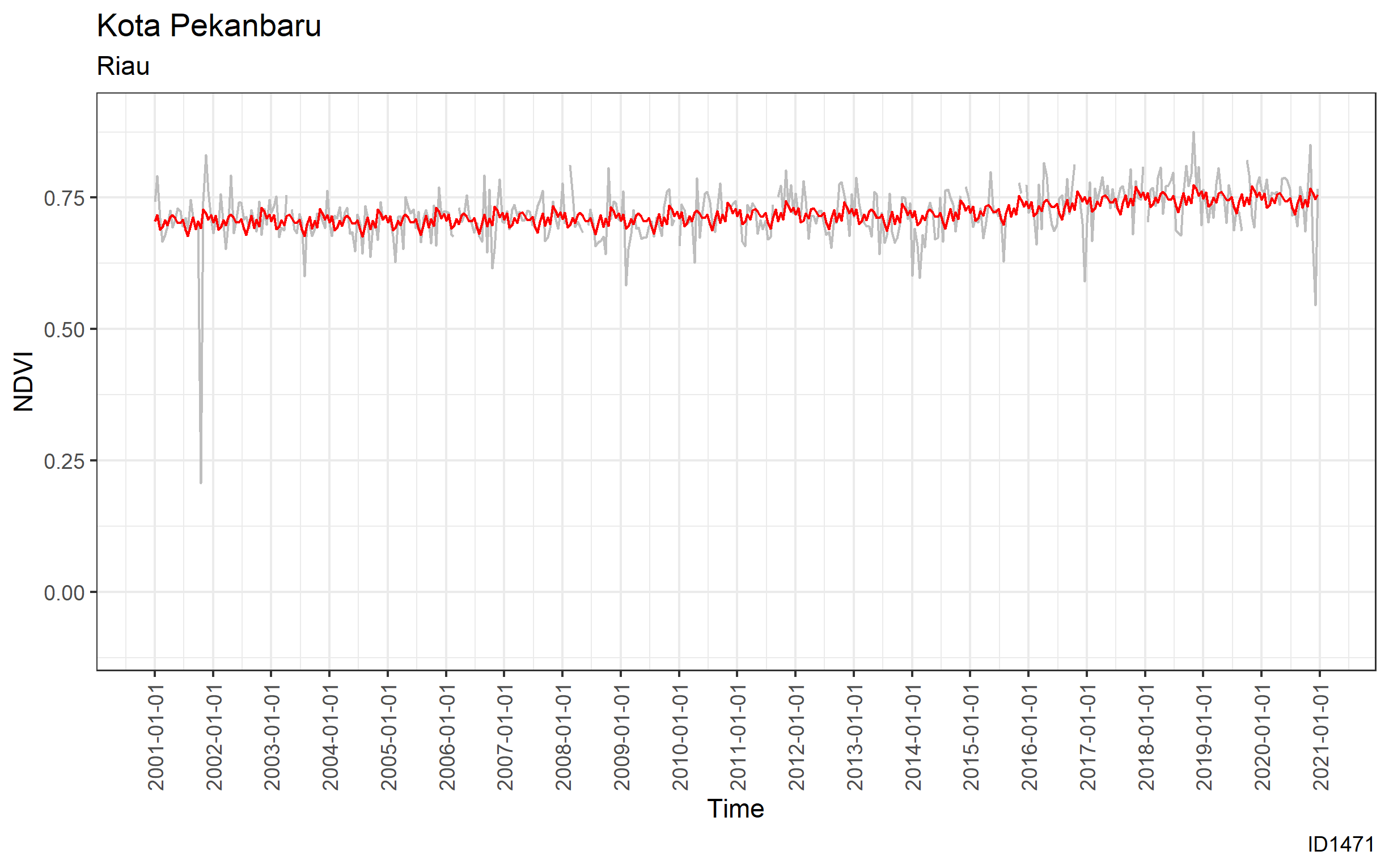

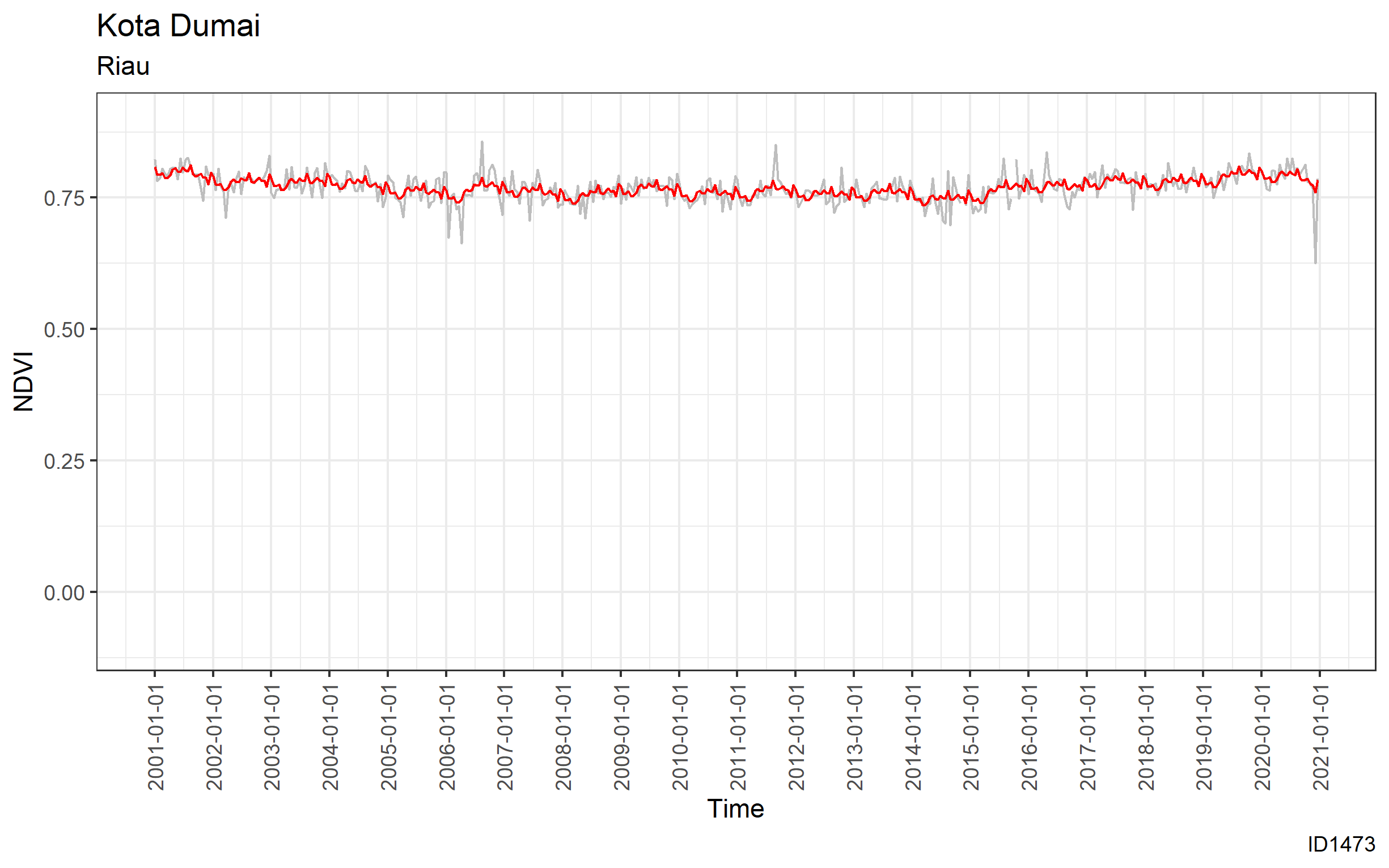


## Jambi Province


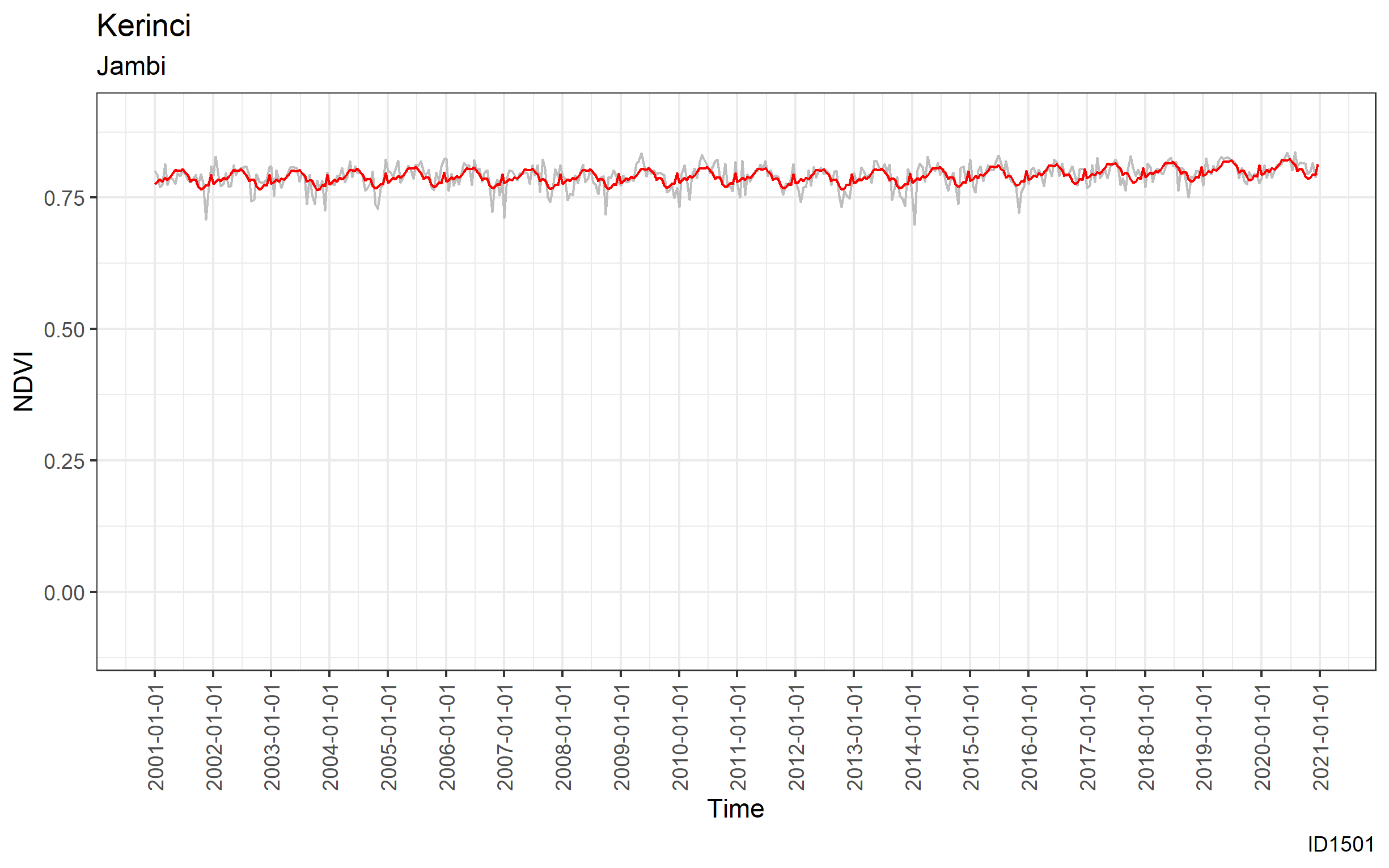

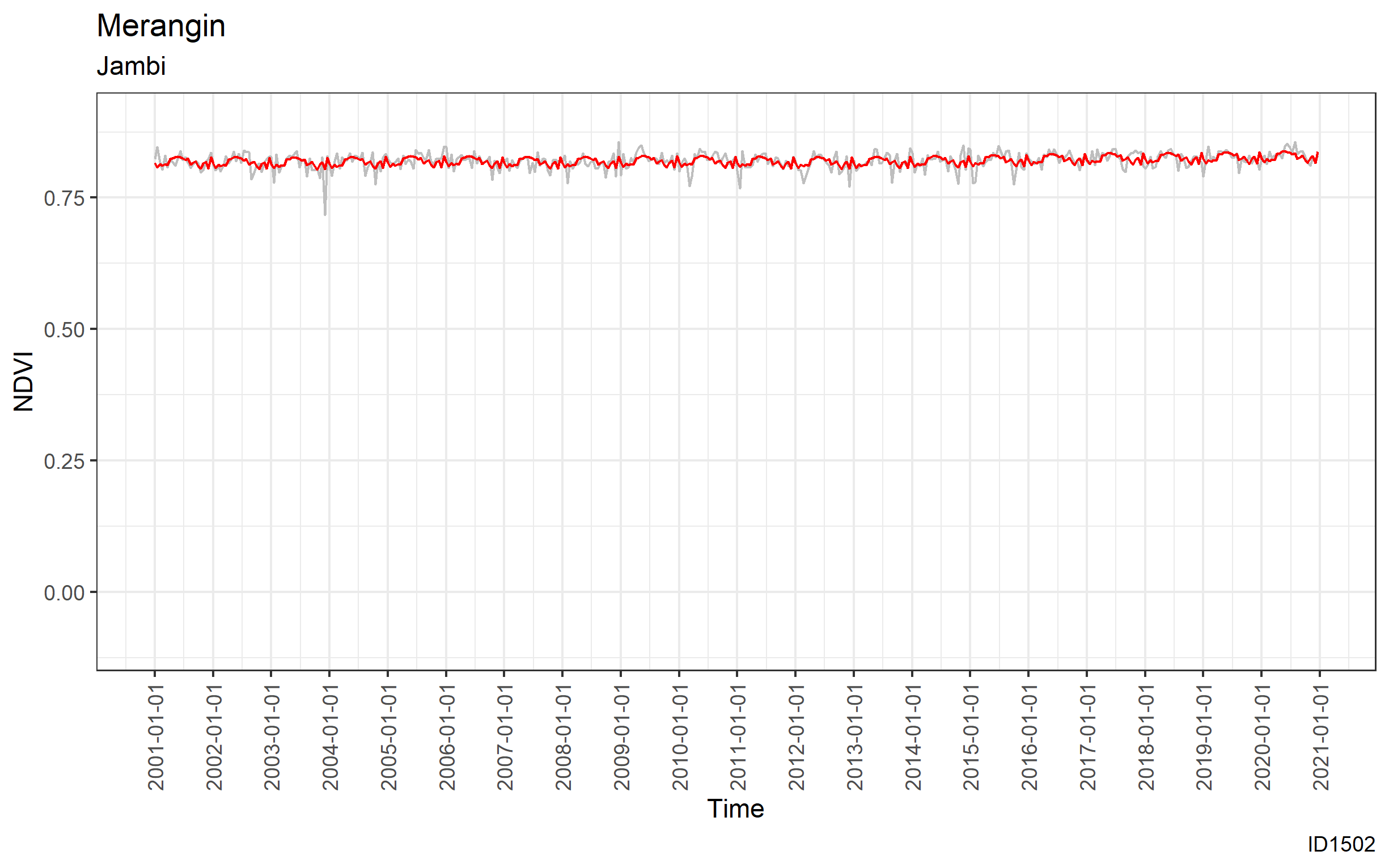

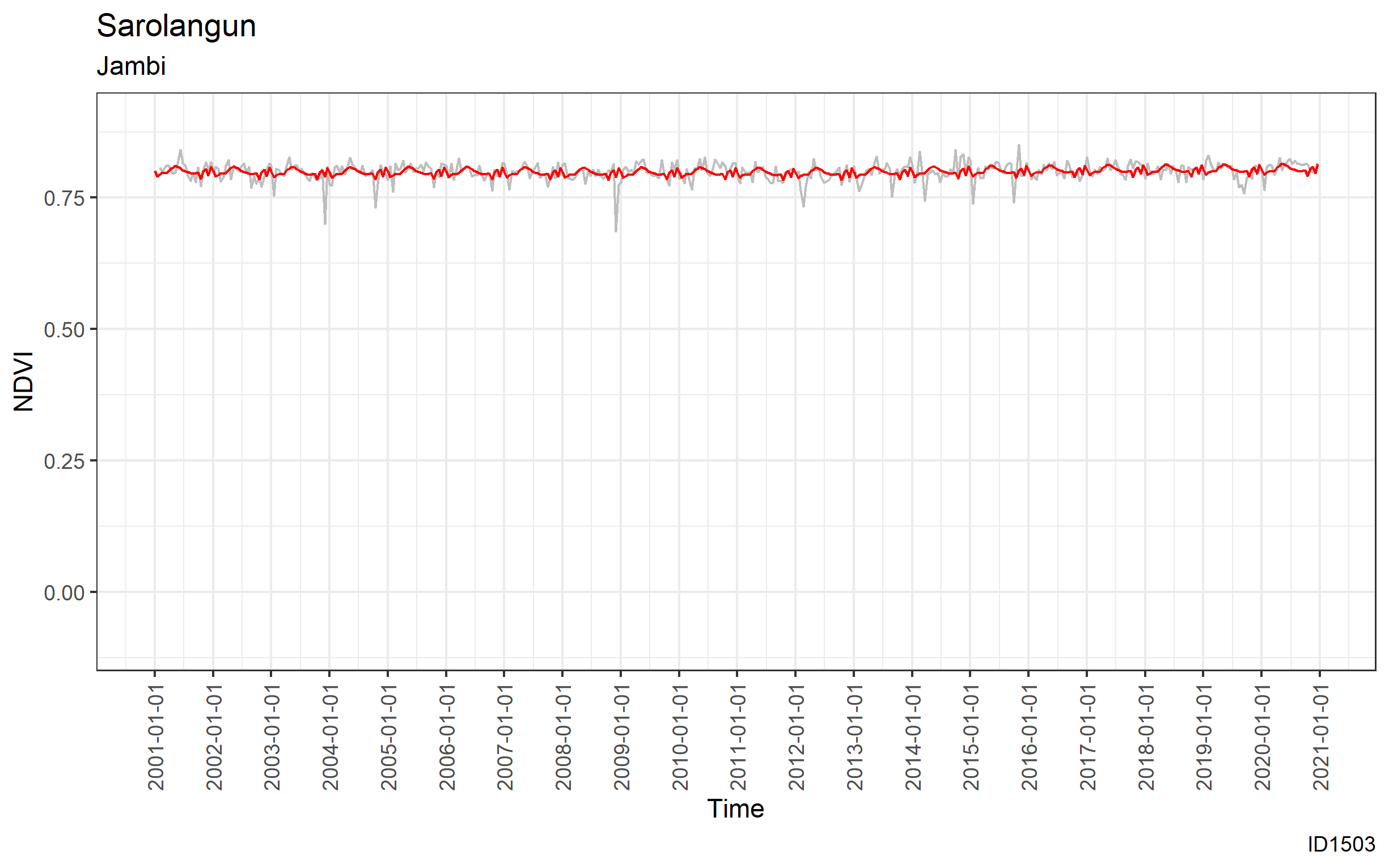

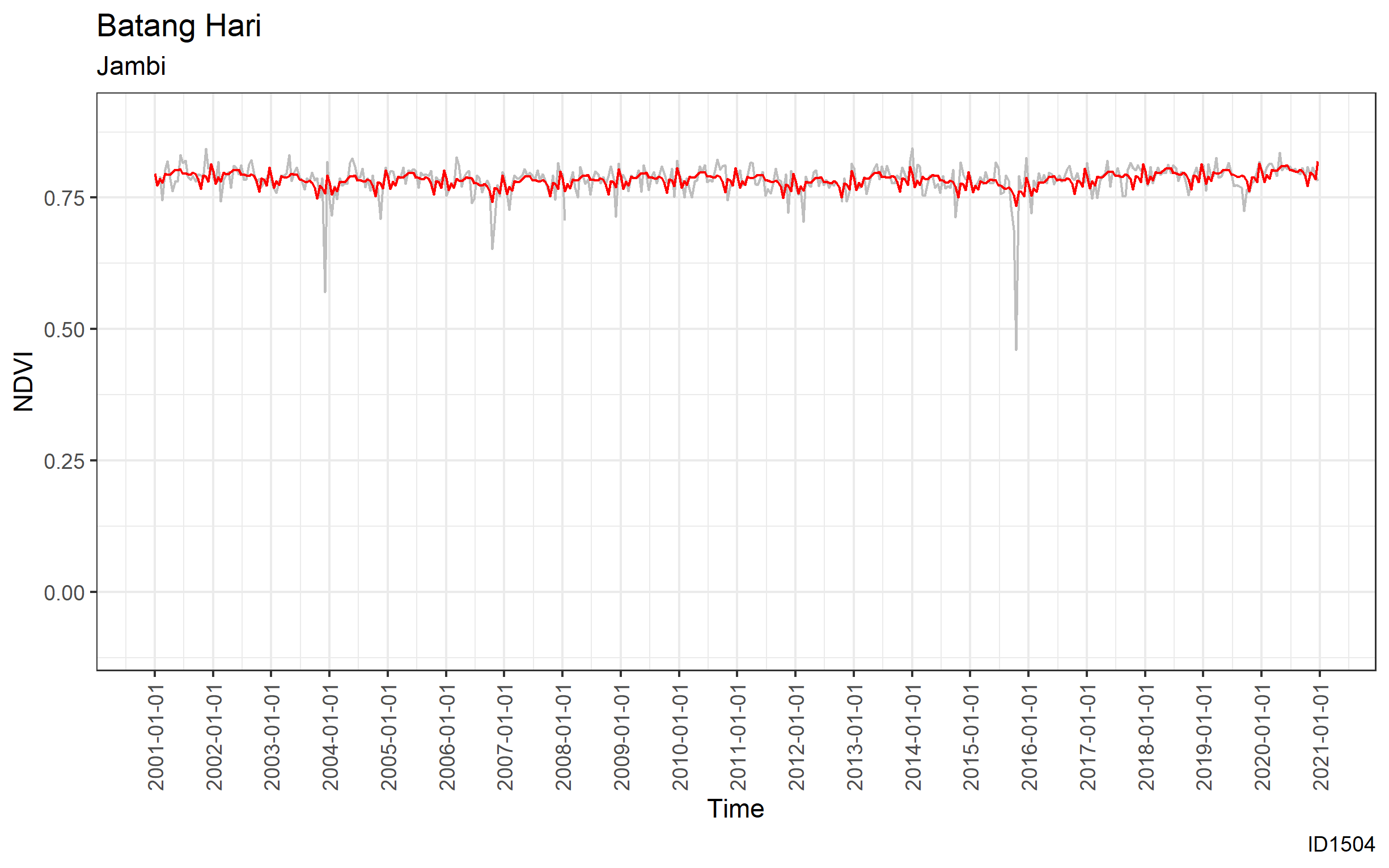


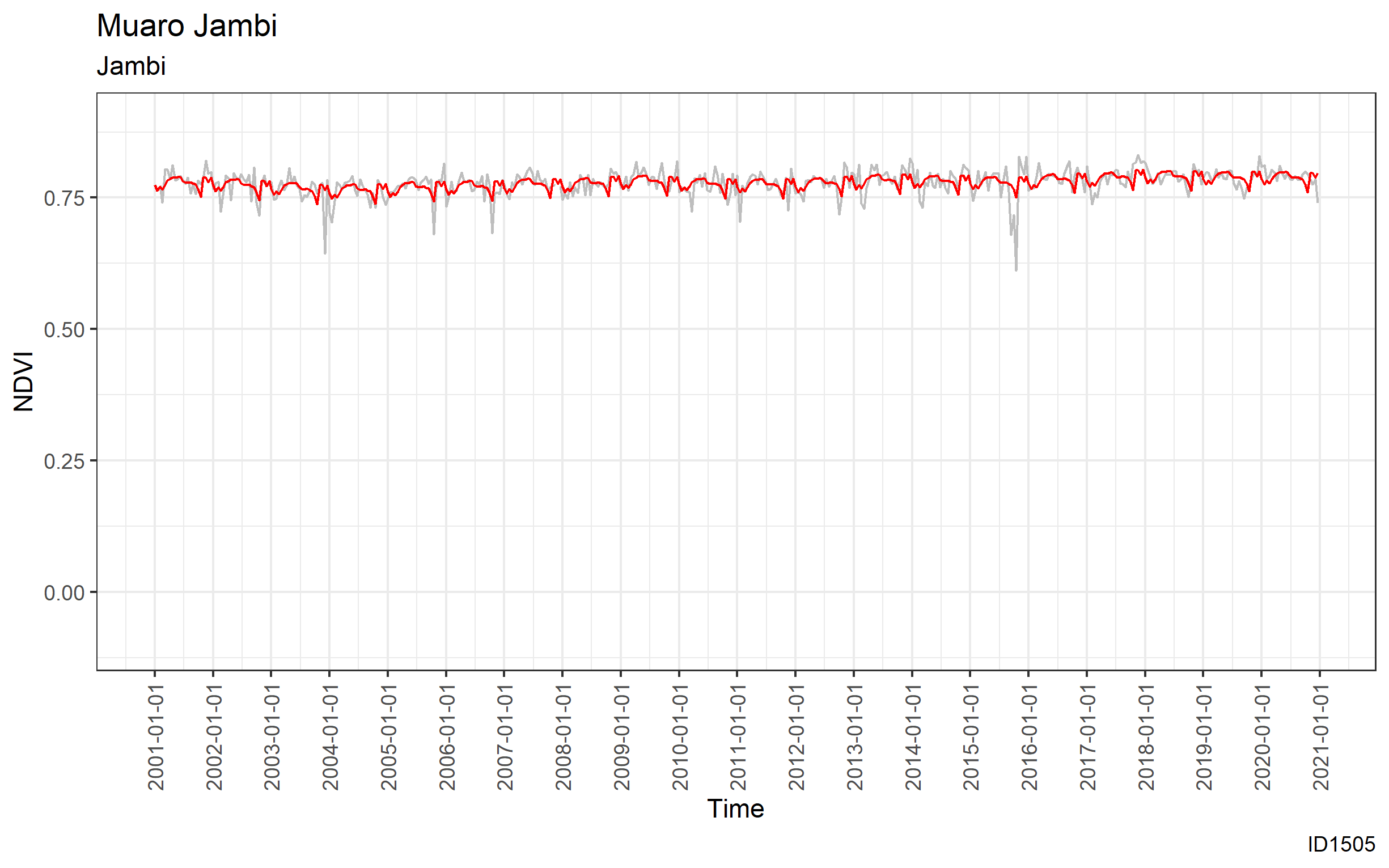

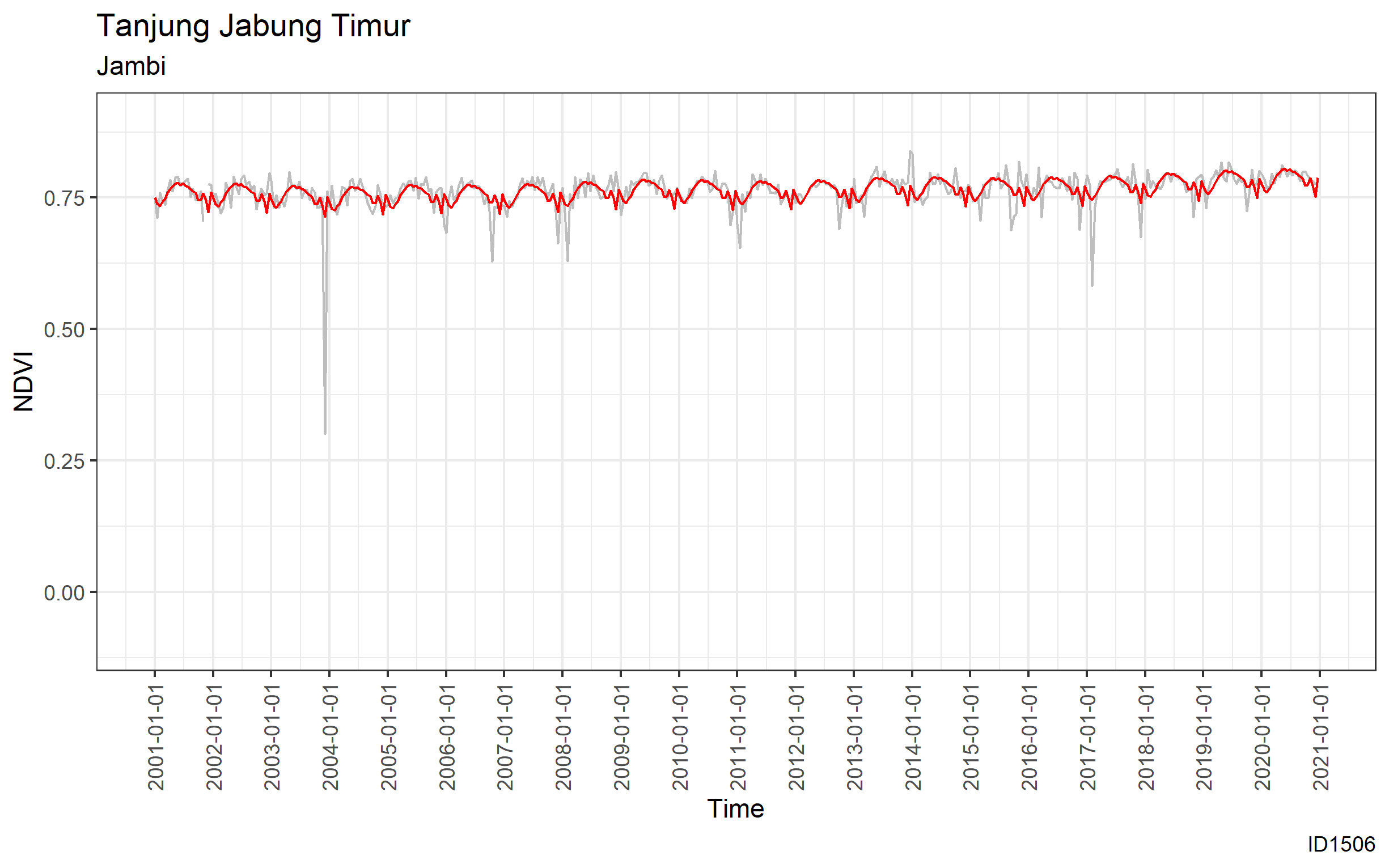

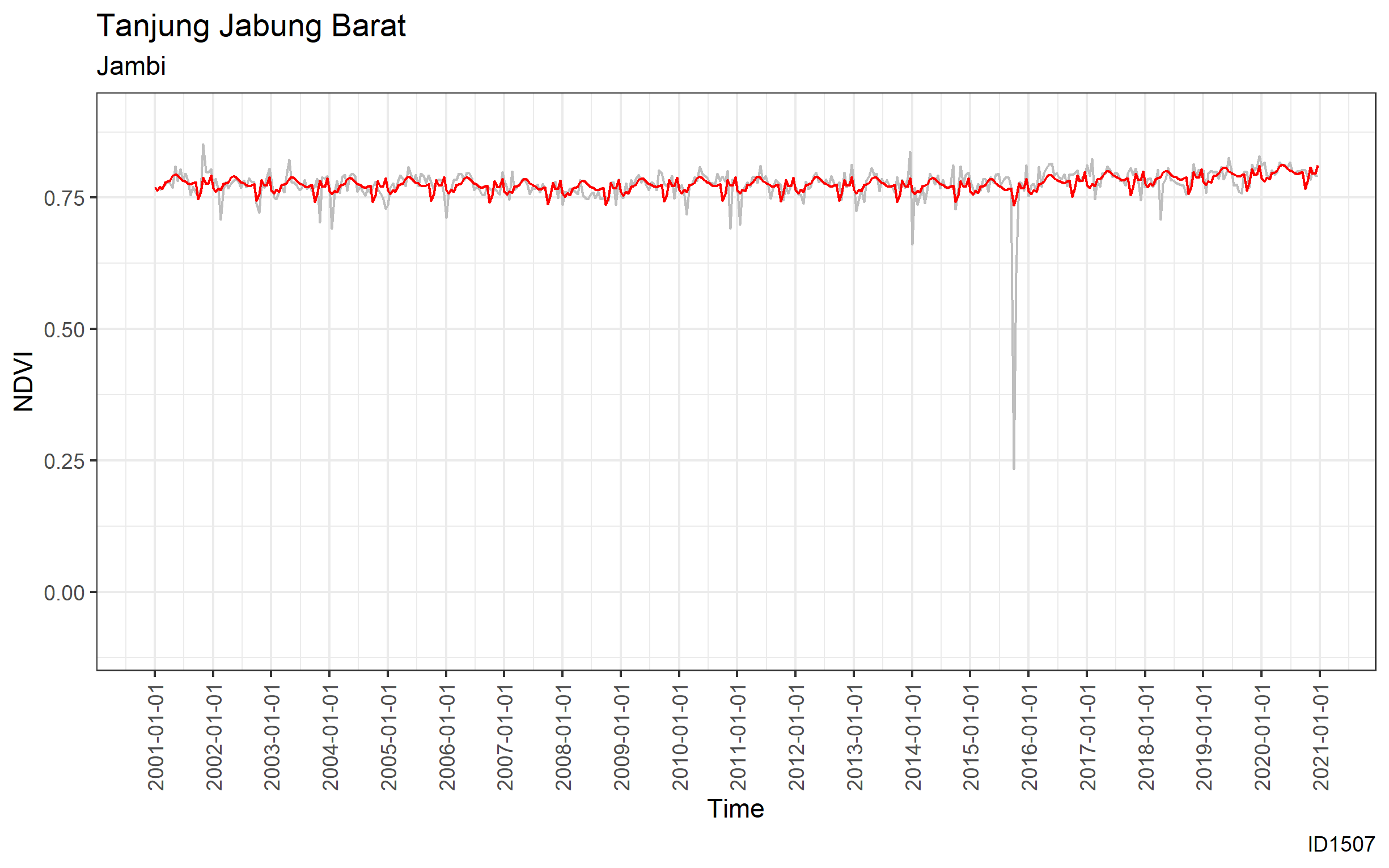

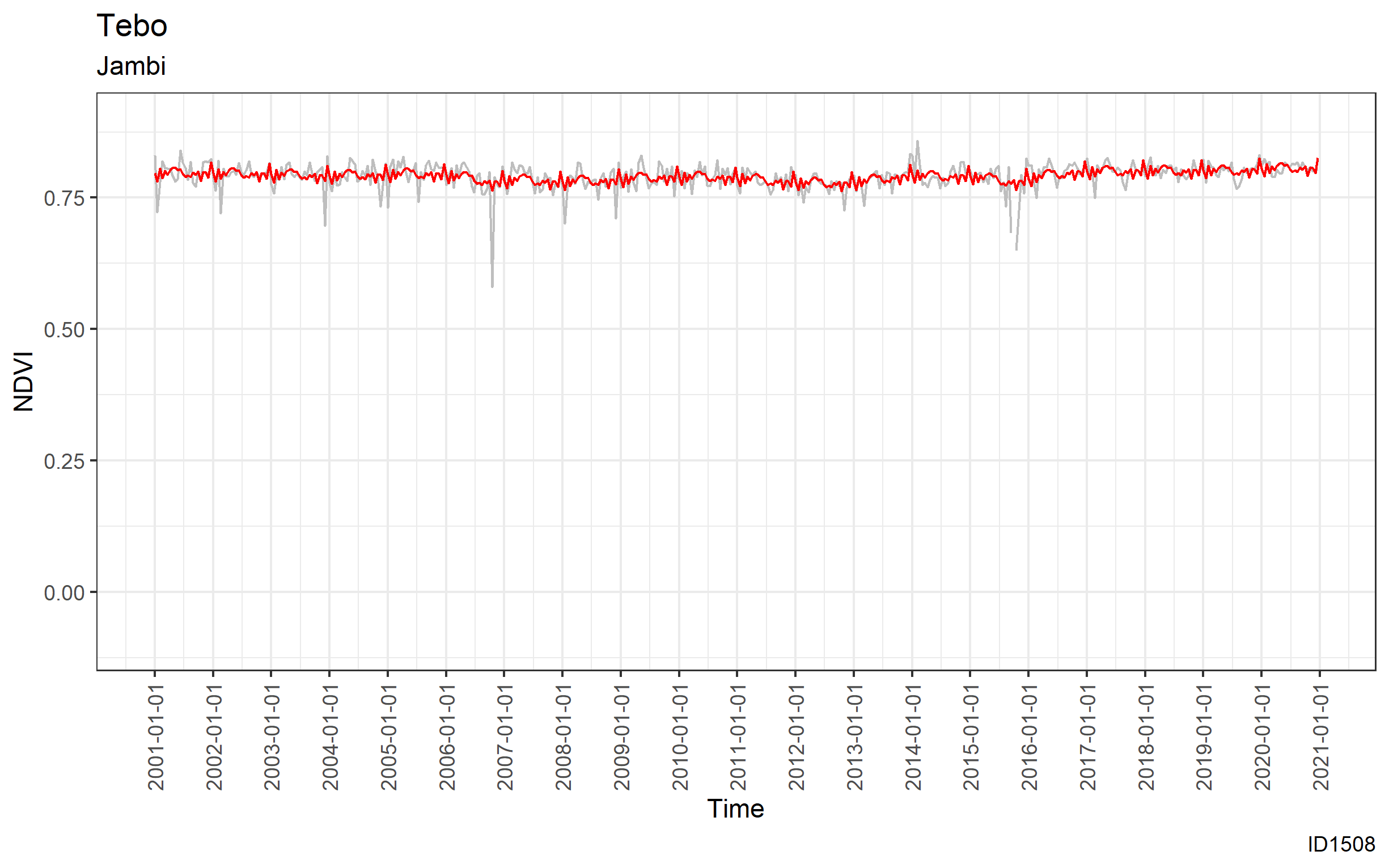

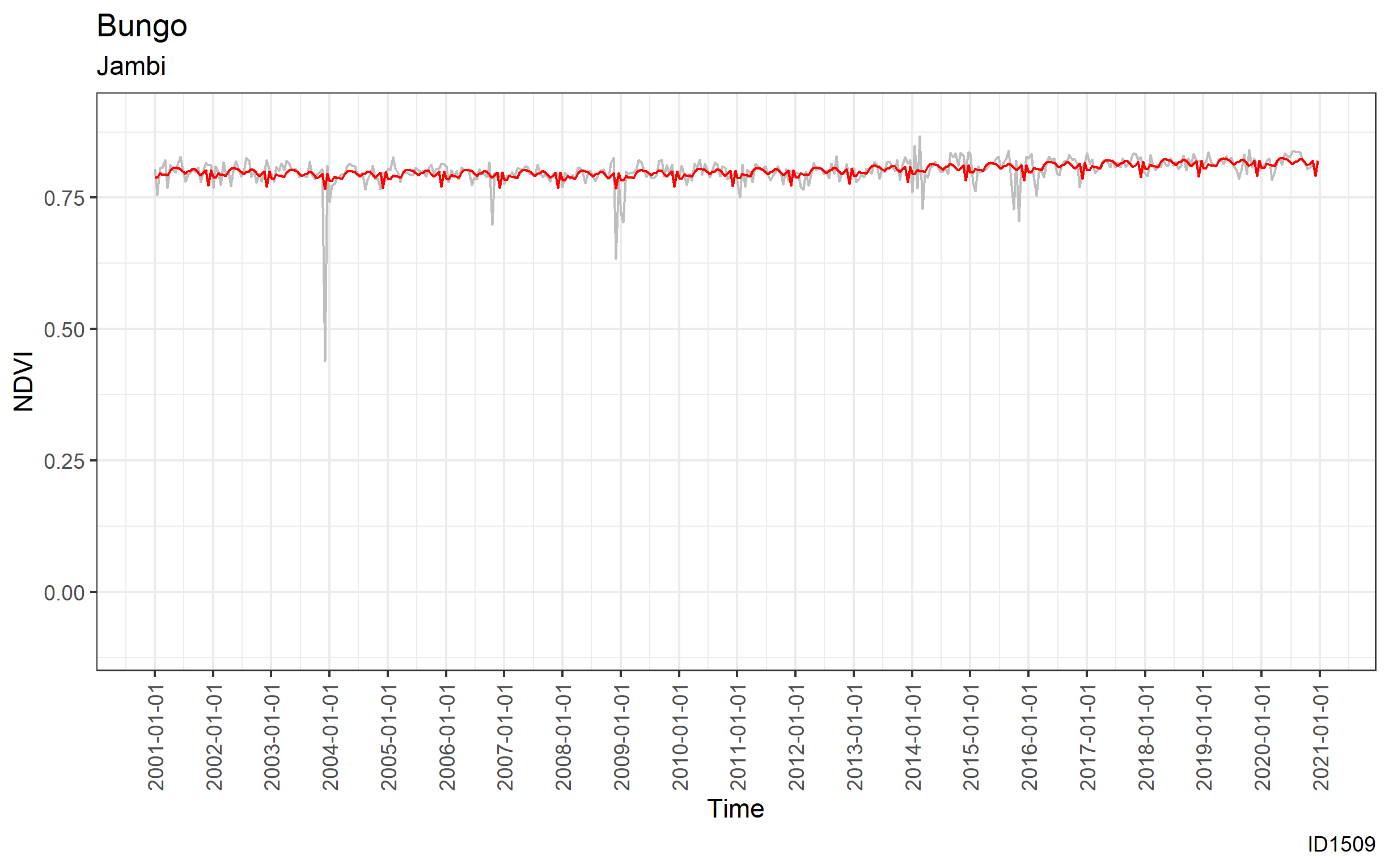

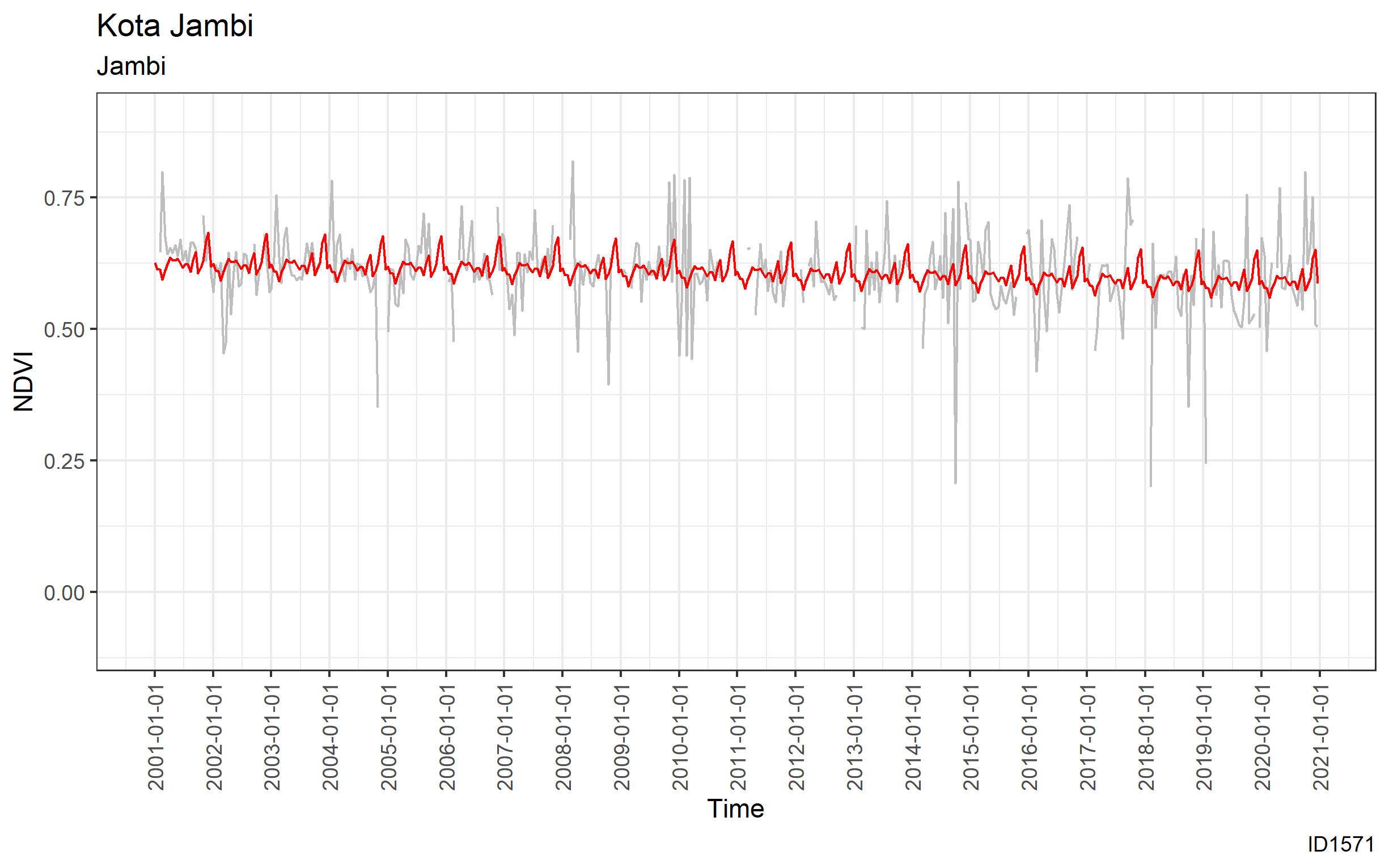

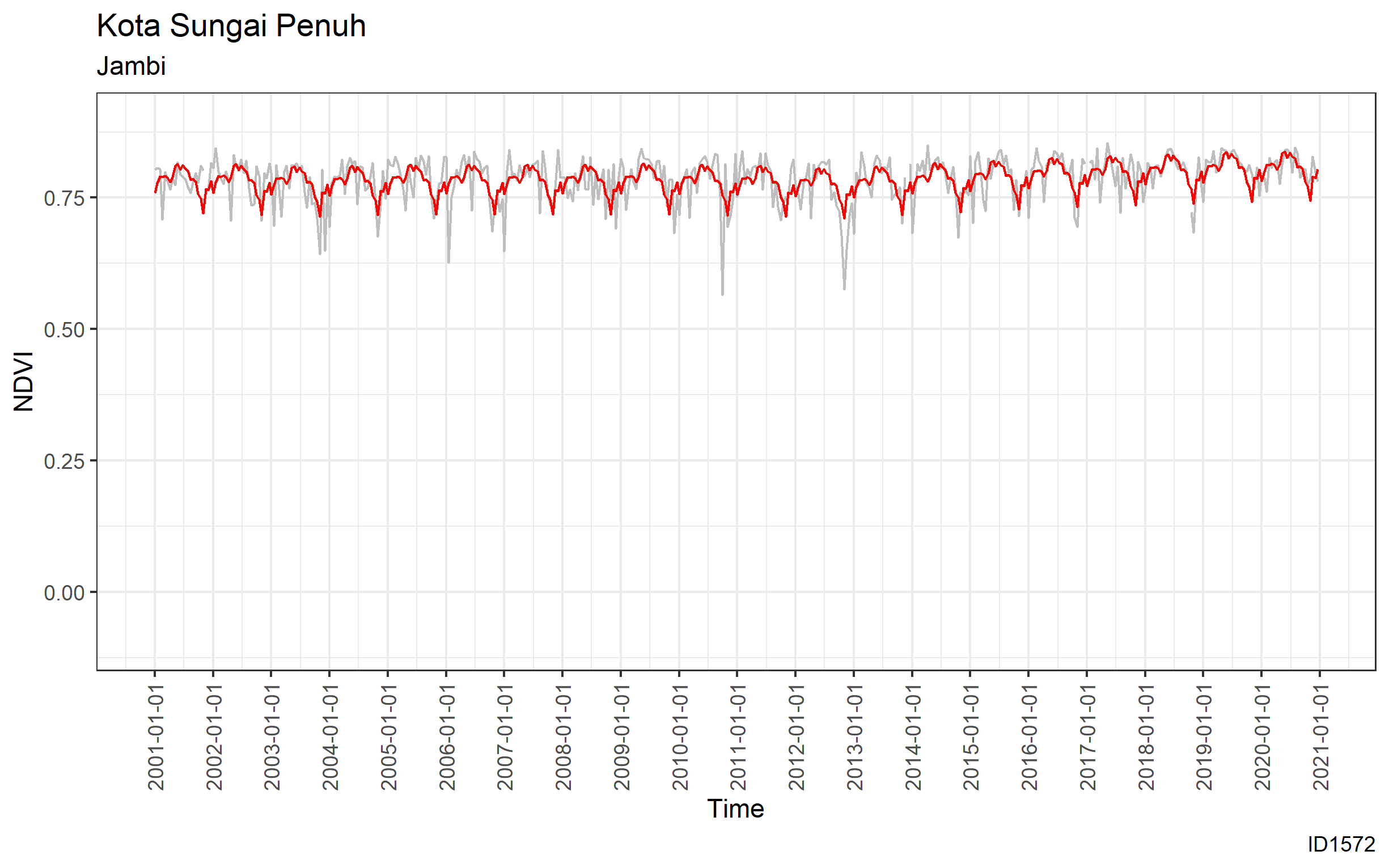


## South Sumatra Province


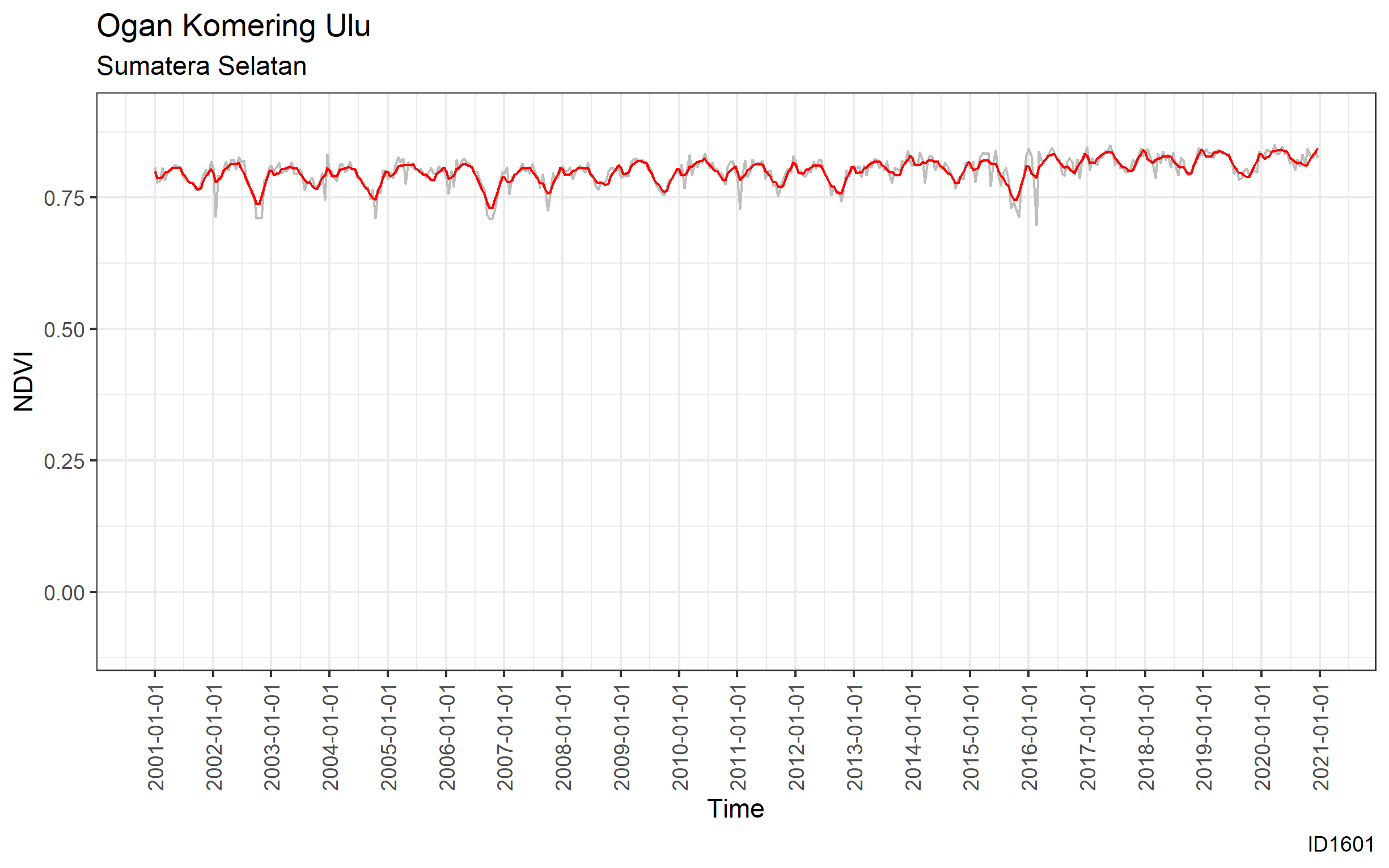


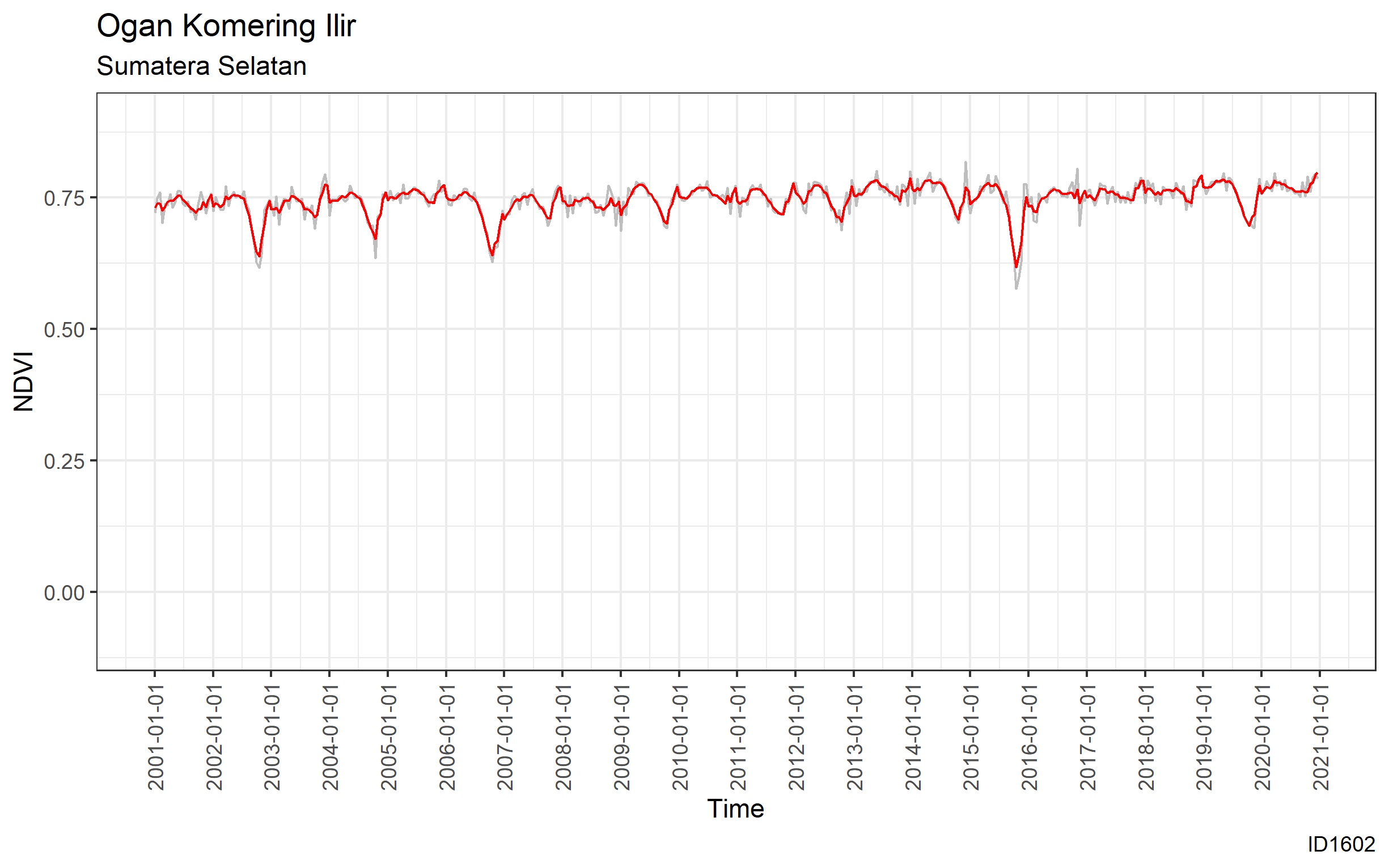


## Bengkulu Province

## Lampung Province

## Bangka Belitung Islands Province

## Riau Islands Province

## Special Capital Region of Jakarta

## West Java Province

## Central Java Province

## Special Region of Yogyakarta

## East Java Province

## Banten Province

## Bali Province

## West Nusa Tenggara Province

## East Nusa Tenggara Province

## West Kalimantan Province

## Central Kalimantan Province

## South Kalimantan Province

## East Kalimantan Province

## North Kalimantan Province

## North Sulawesi Province

## Central Sulawesi Province

## South Sulawesi Province

## Southeast Sulawesi Province

## Gorontalo Province

## West Sulawesi Province

## Maluku Province

## North Maluku Province

## West Papua Province

## Papua Province
