# Supplementary material for: Time-series analysis of satellite imagery for detecting vegetation cover changes in Indonesia
Source: Sci Rep. 2023 May 25;13:8437. doi: 10.1038/s41598-023-35330-1 (PMC10212945; doi:10.1038/s41598-023-35330-1)

Figure S4. Map of the yearly average NDVI at the regency/city level all over Indonesia. This map was created by TF using QGIS 3.22.4 Białowieża software and open administrative boundary data published by OCHA HDX.

2001
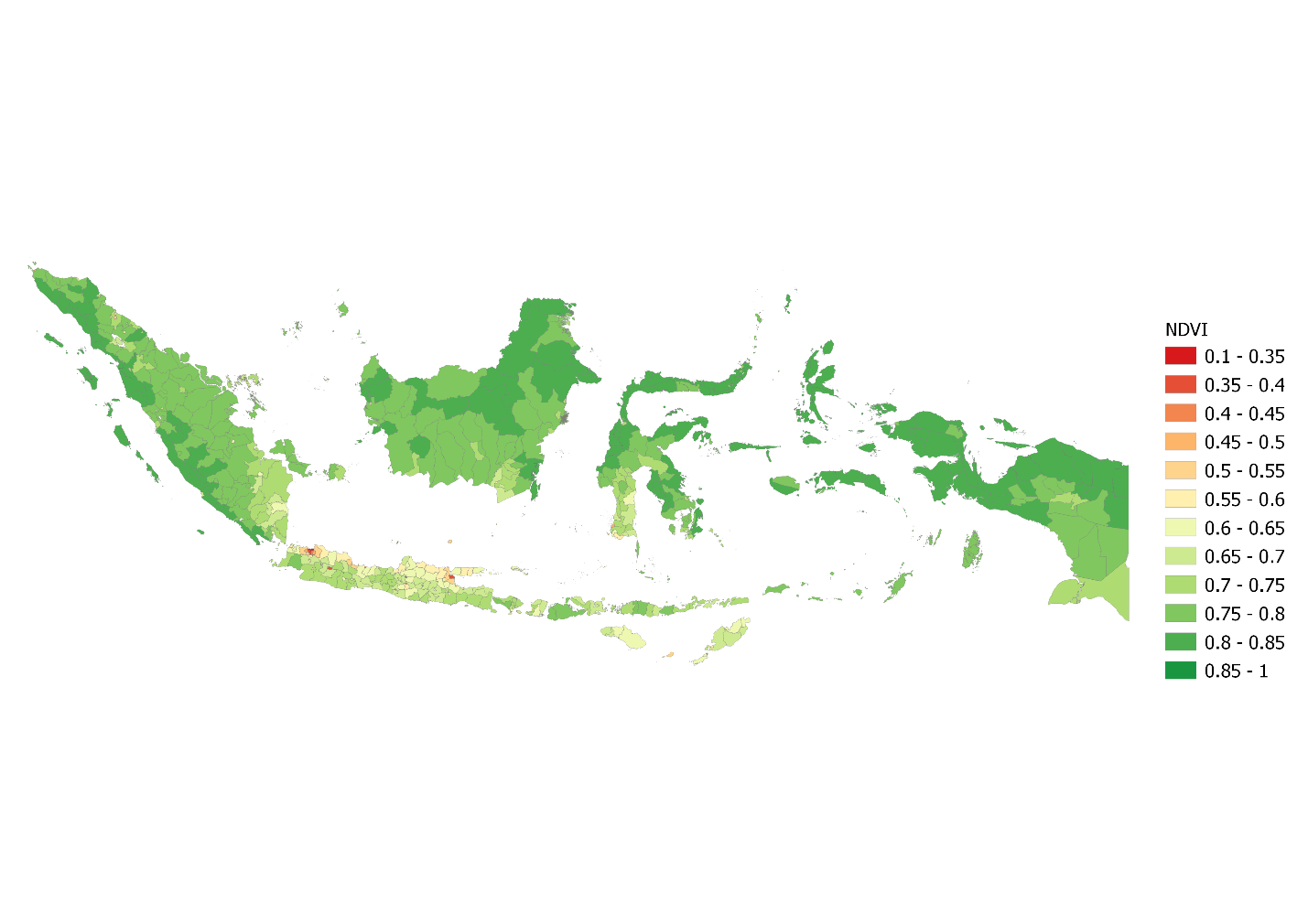


2002
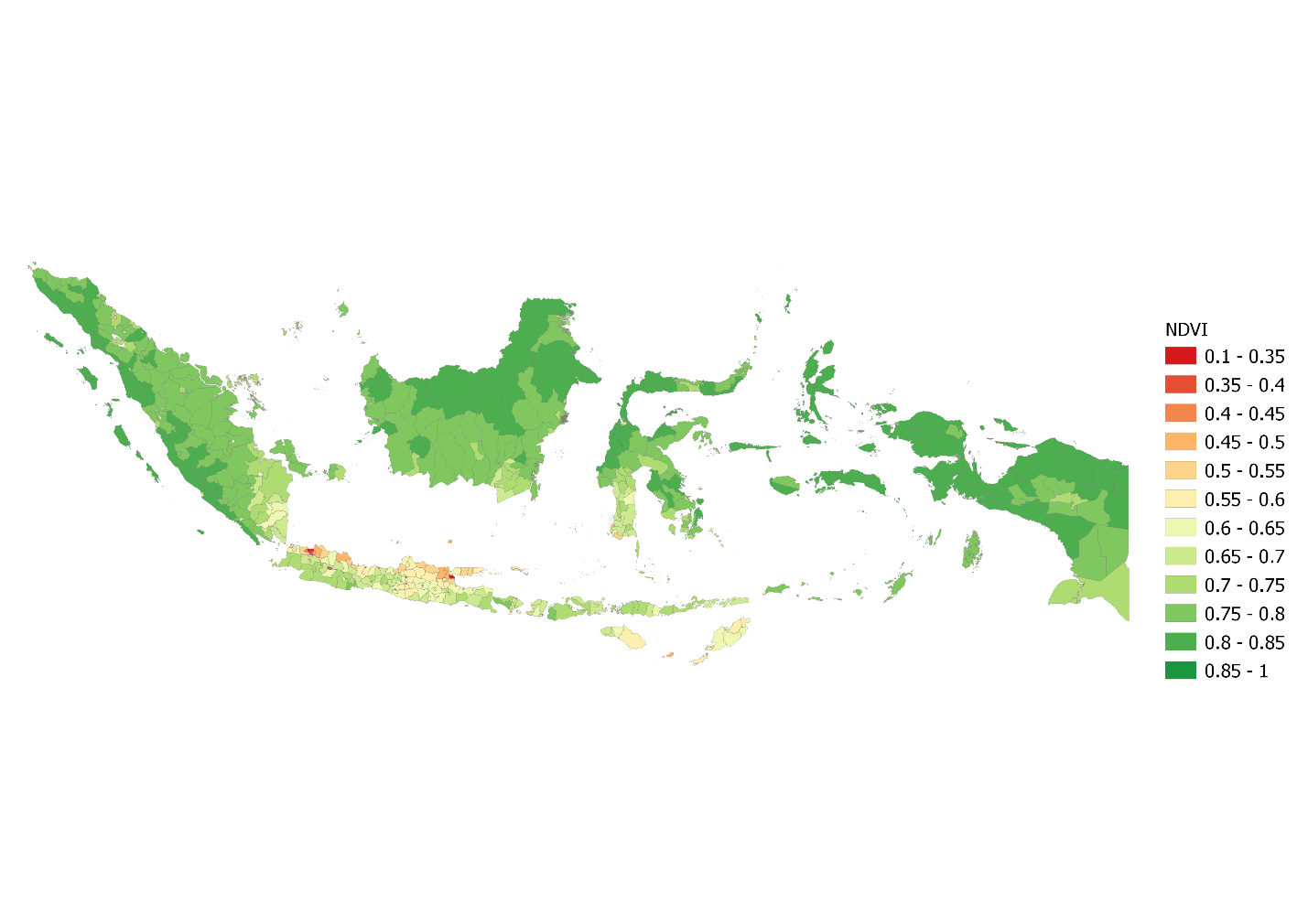


2003
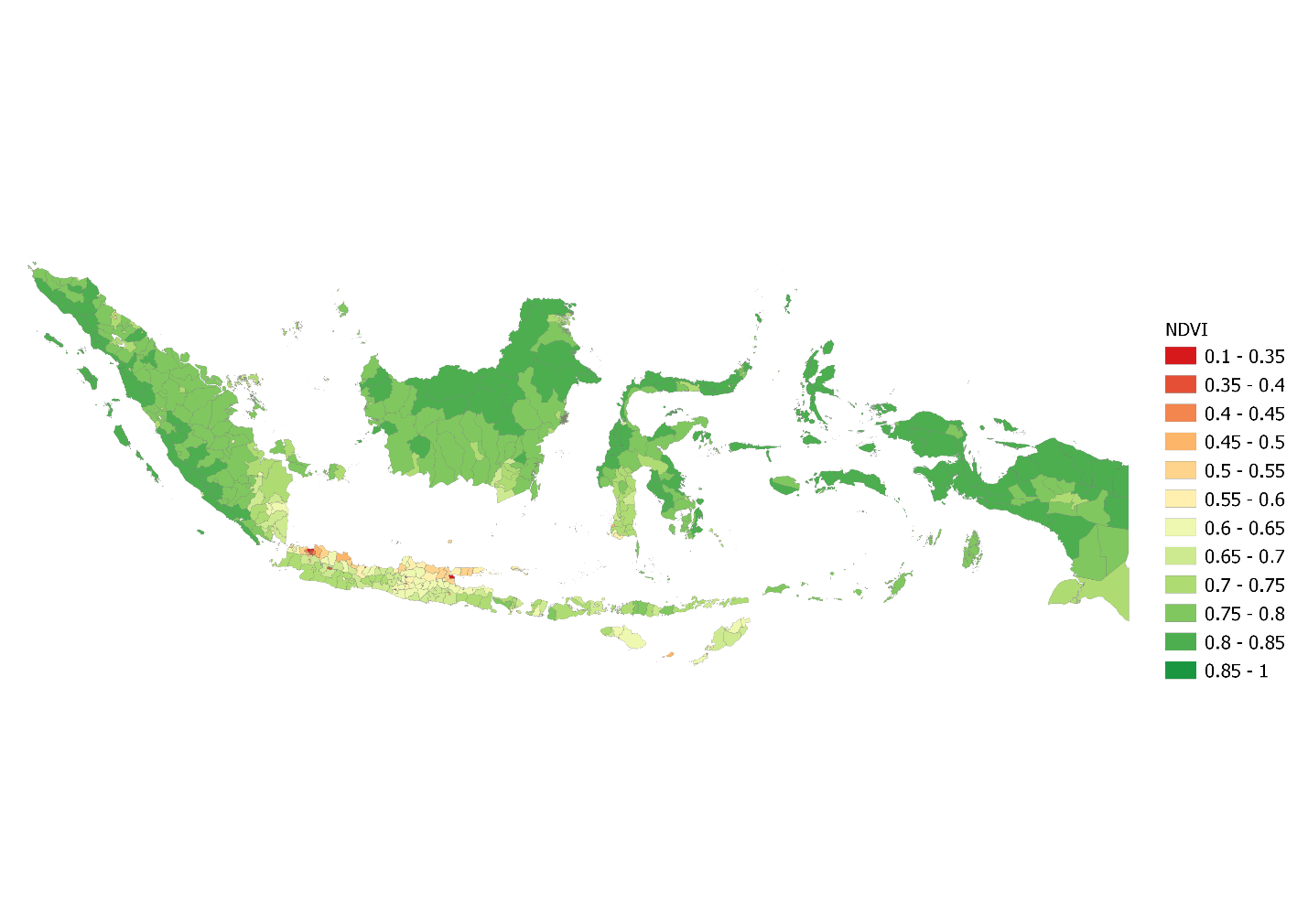


2004
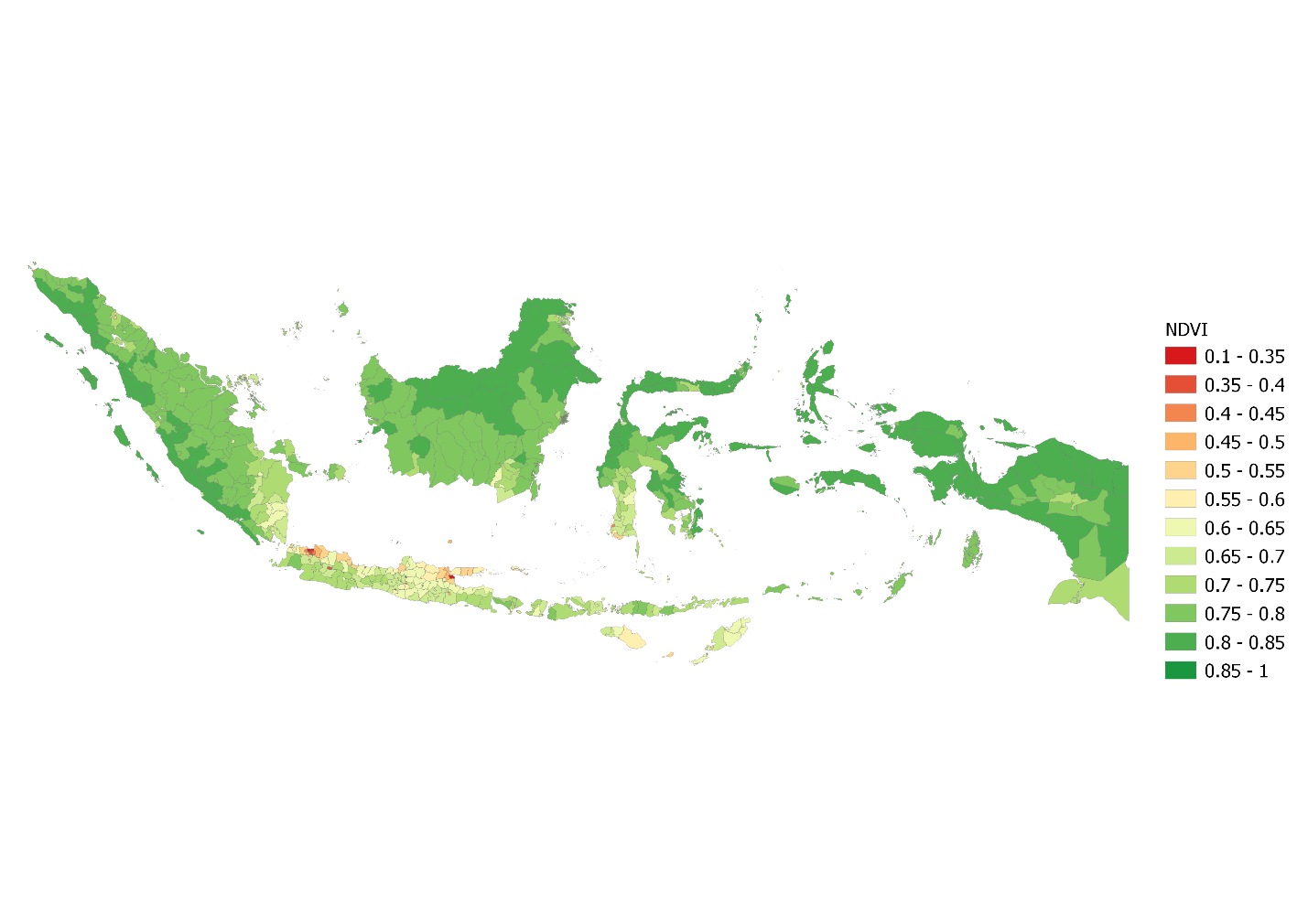


2005
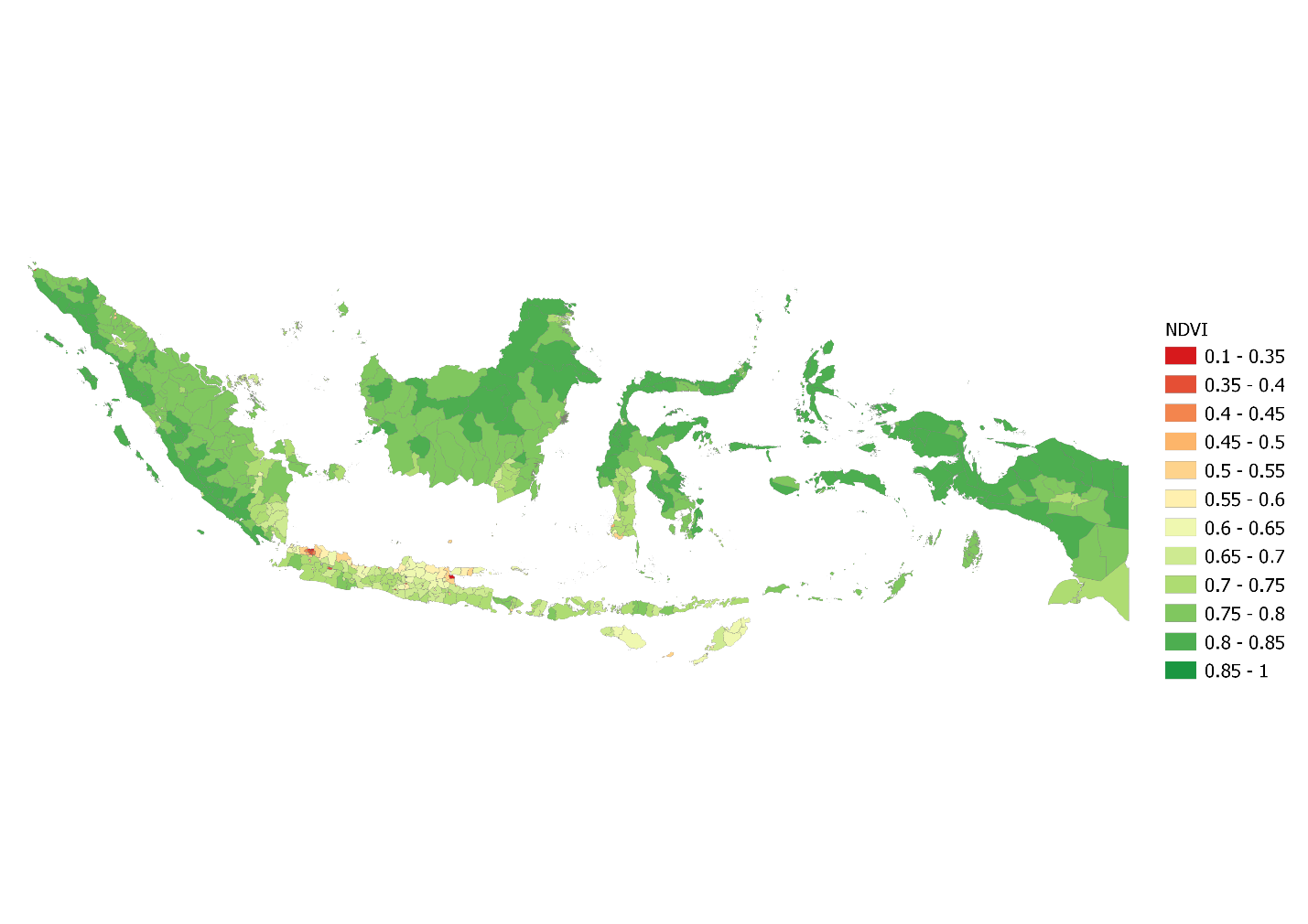
2006
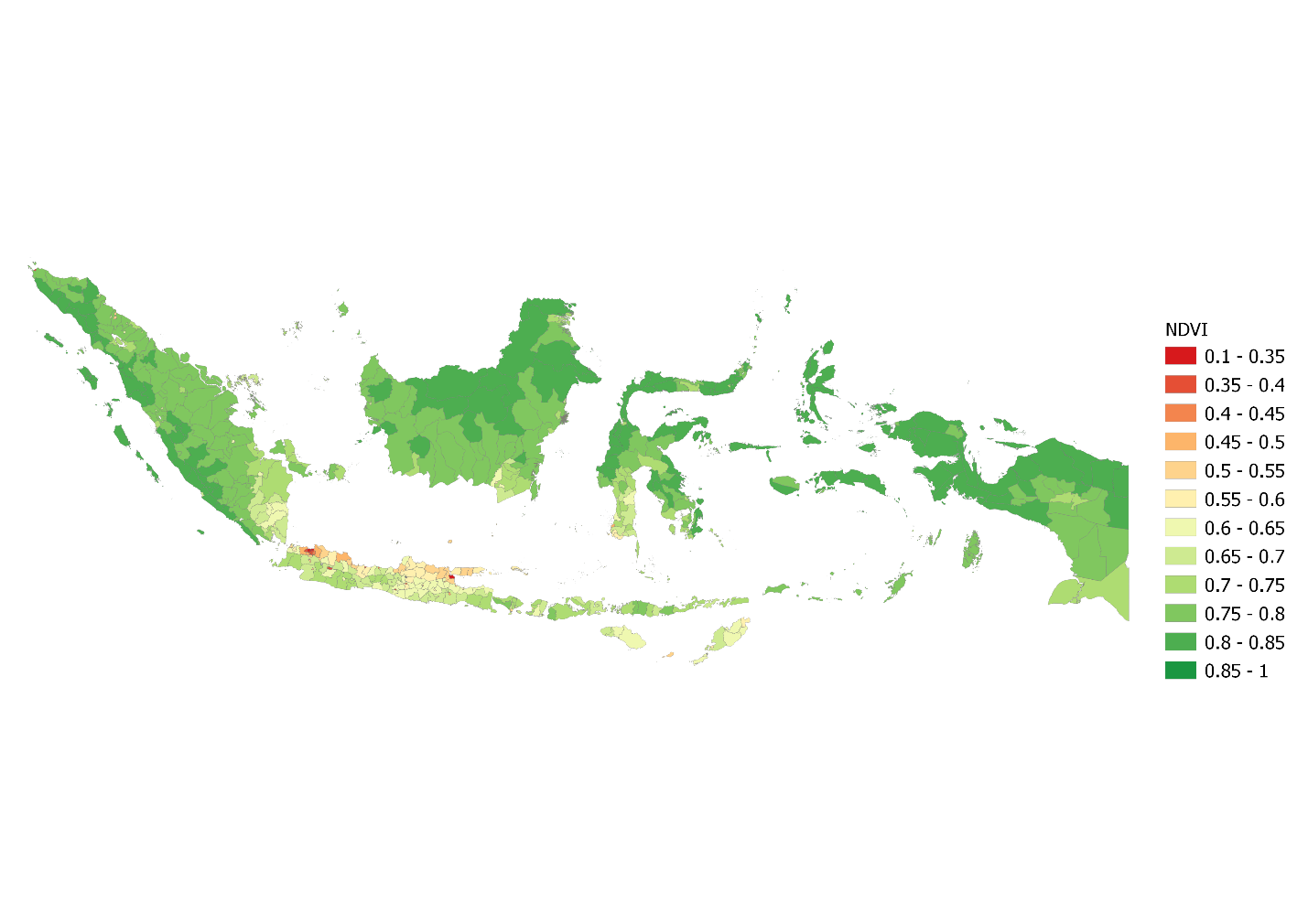


2007
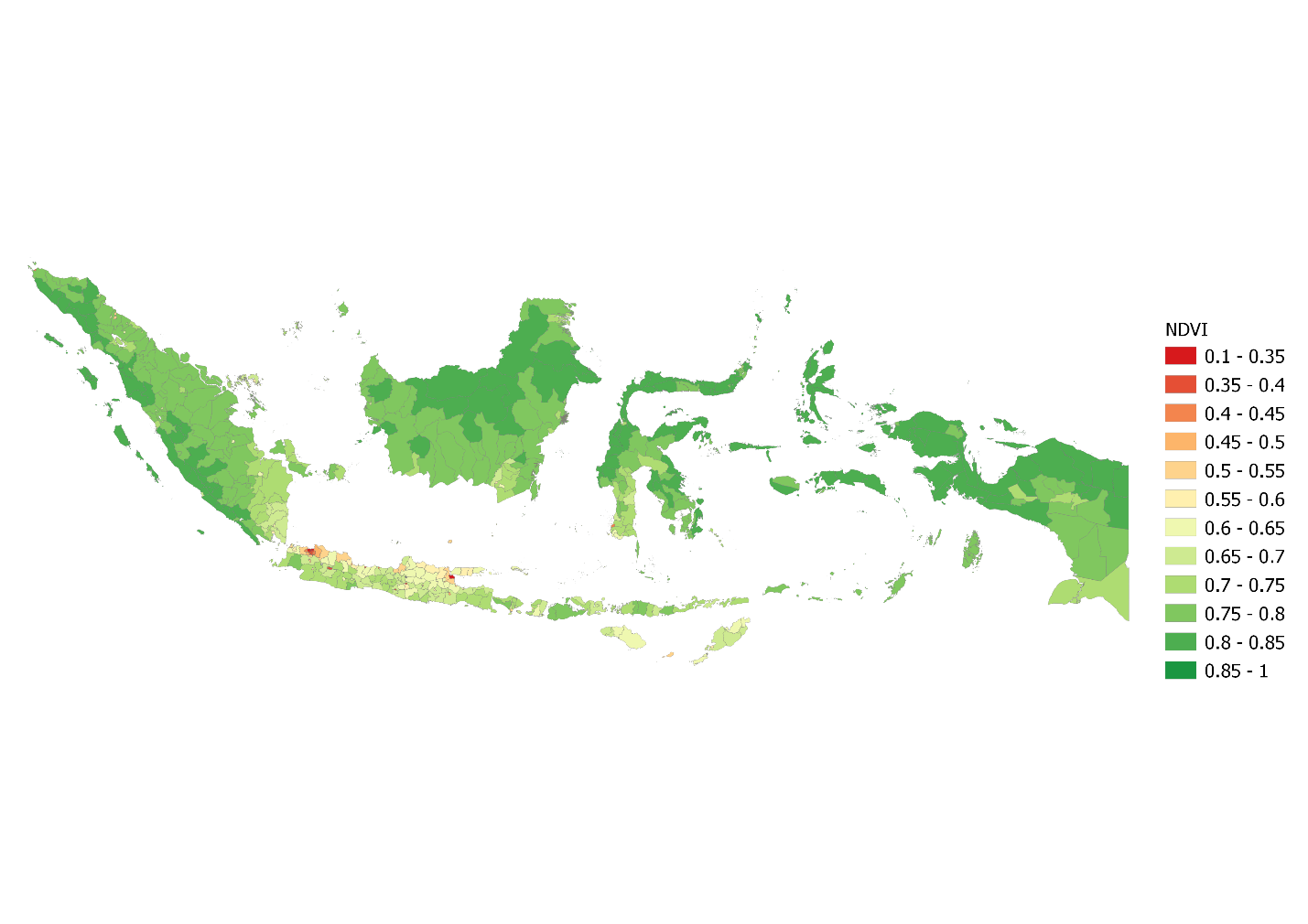
2008
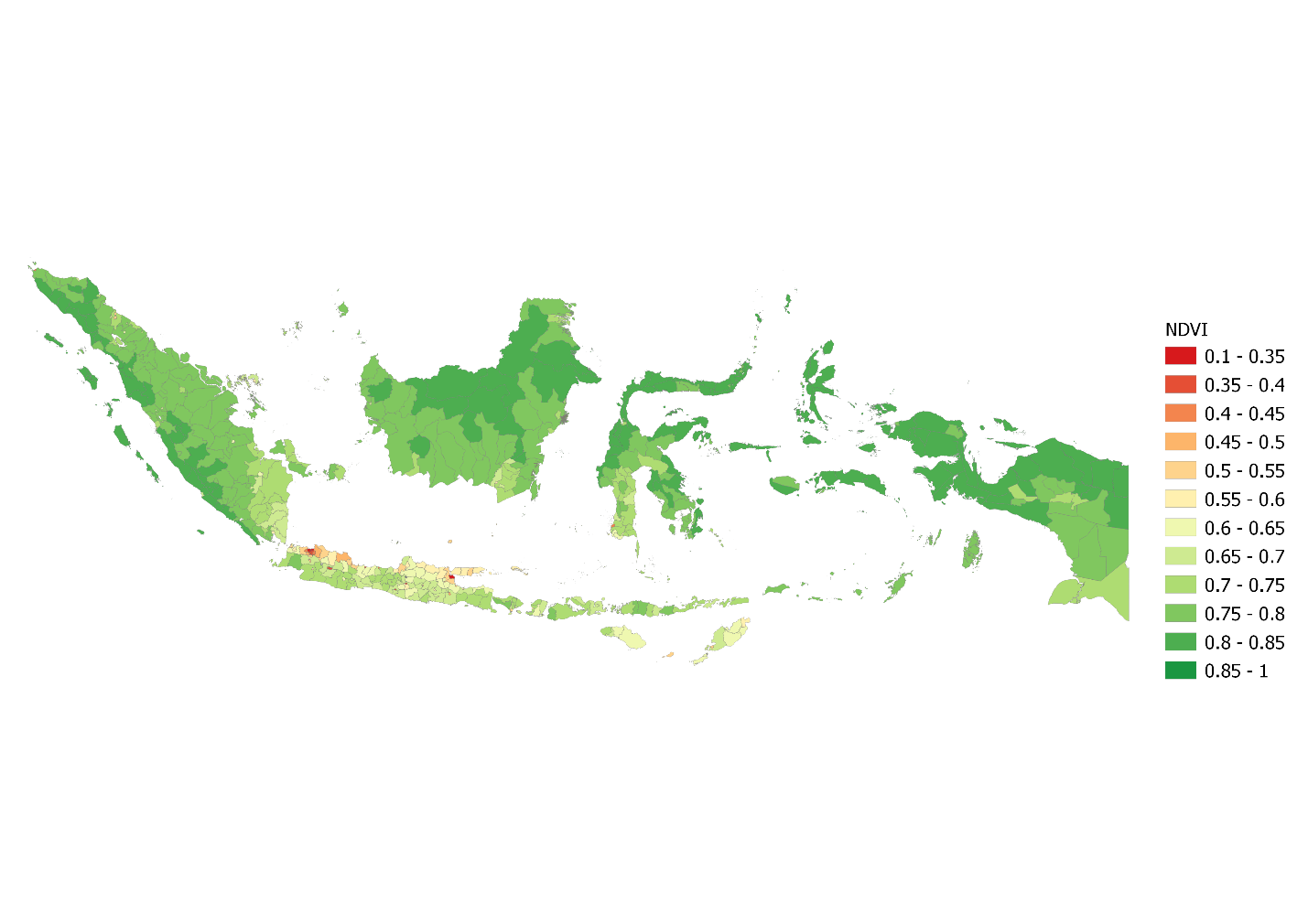


2009
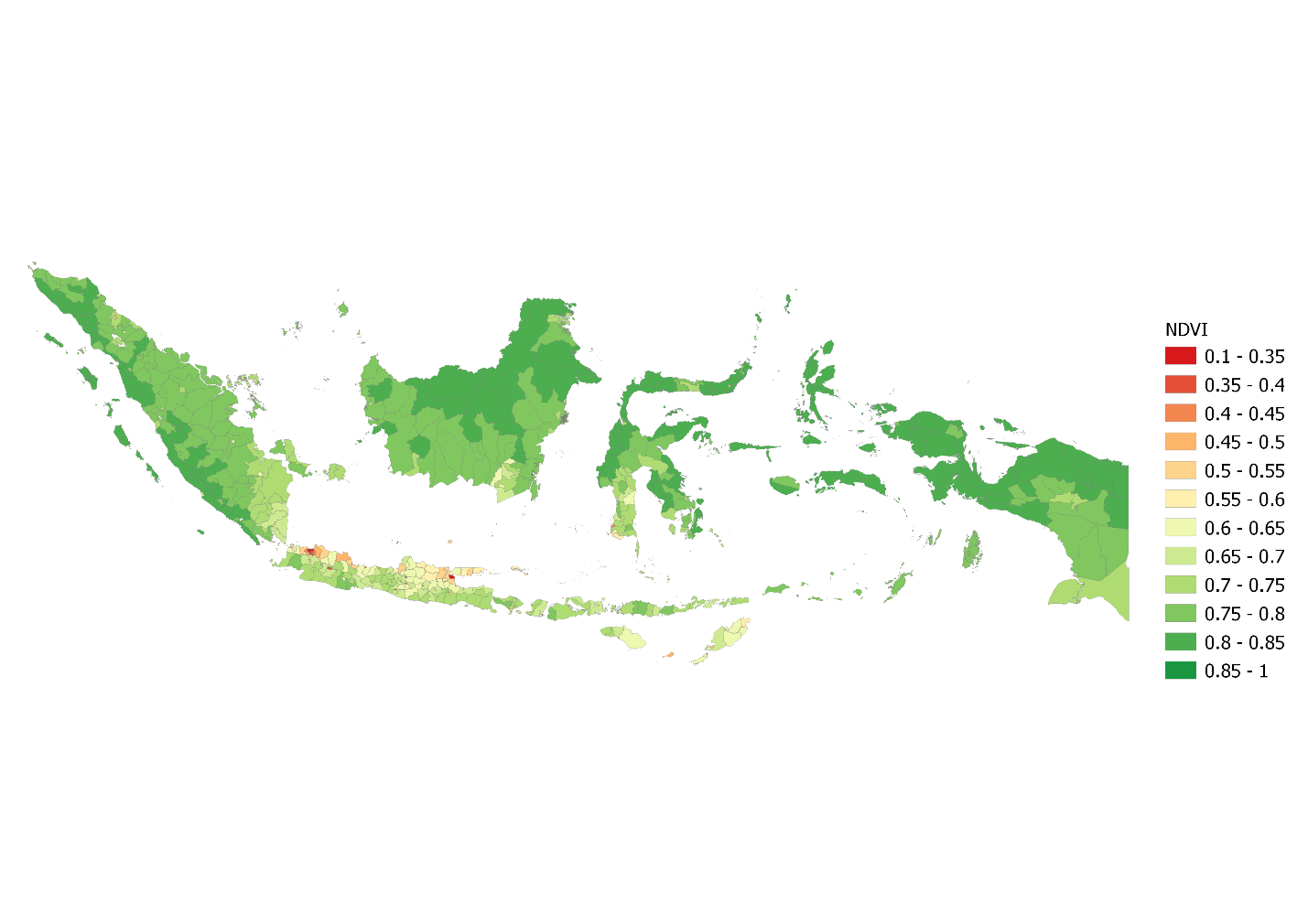
2010
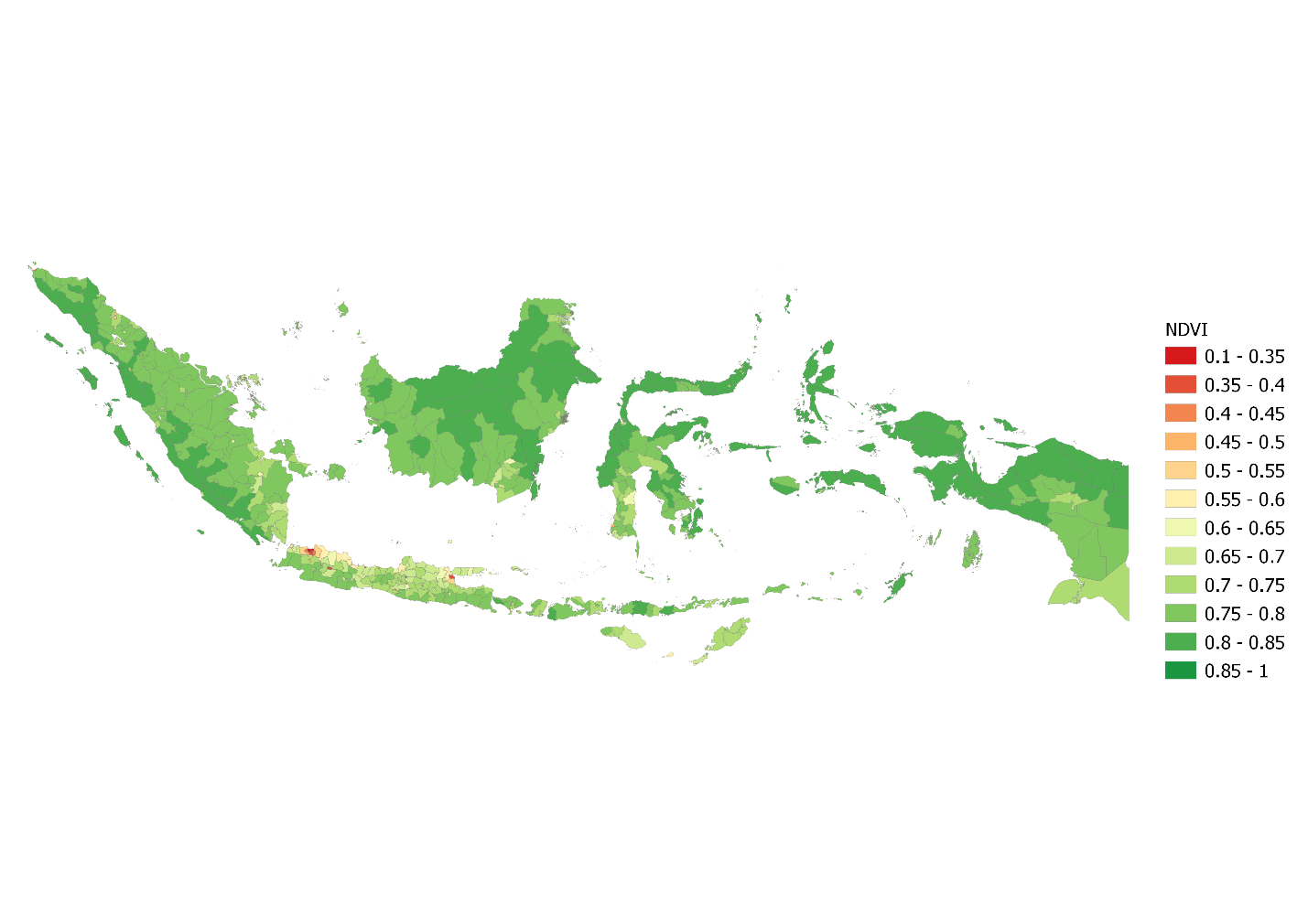


2011
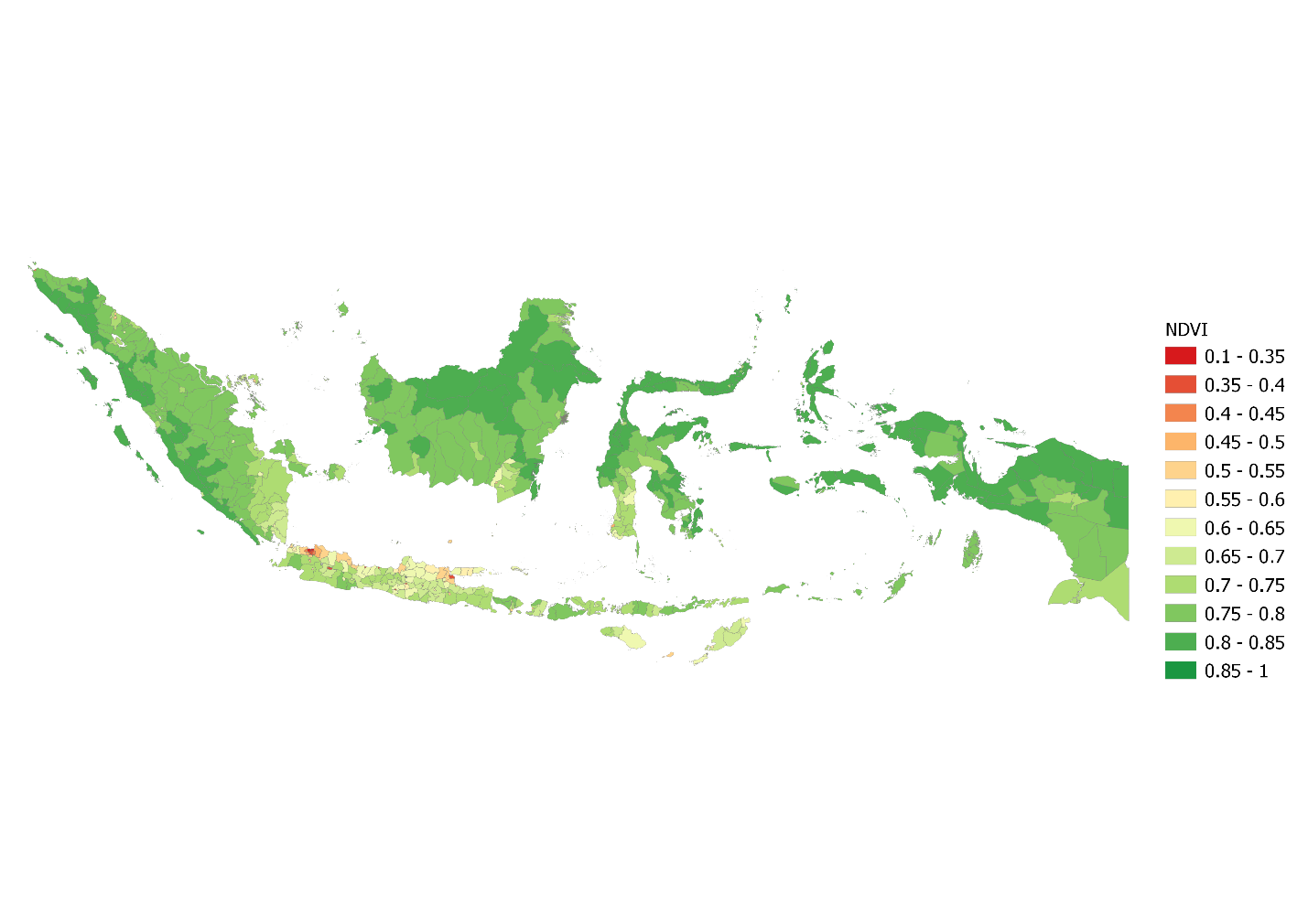
2012
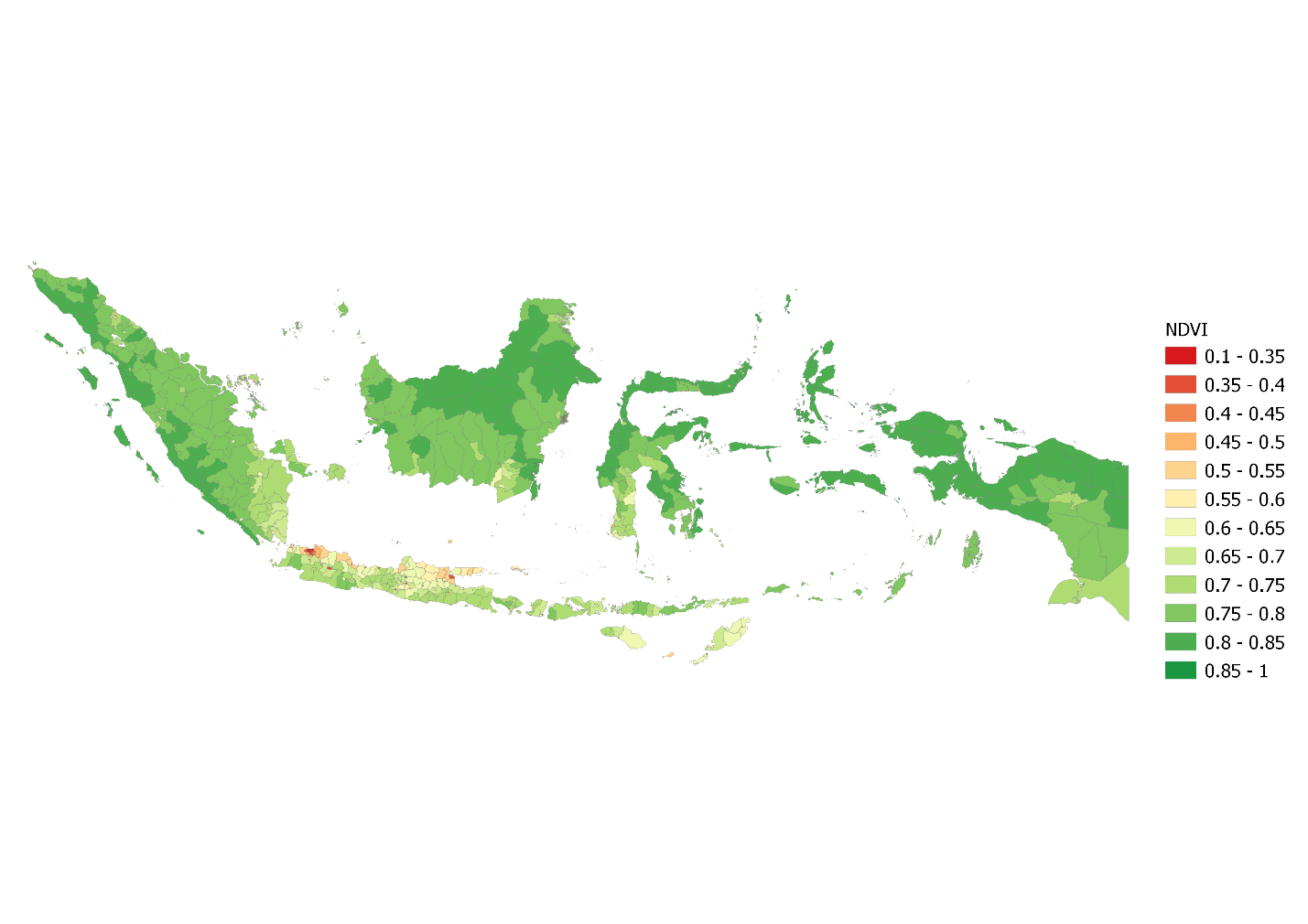


2013
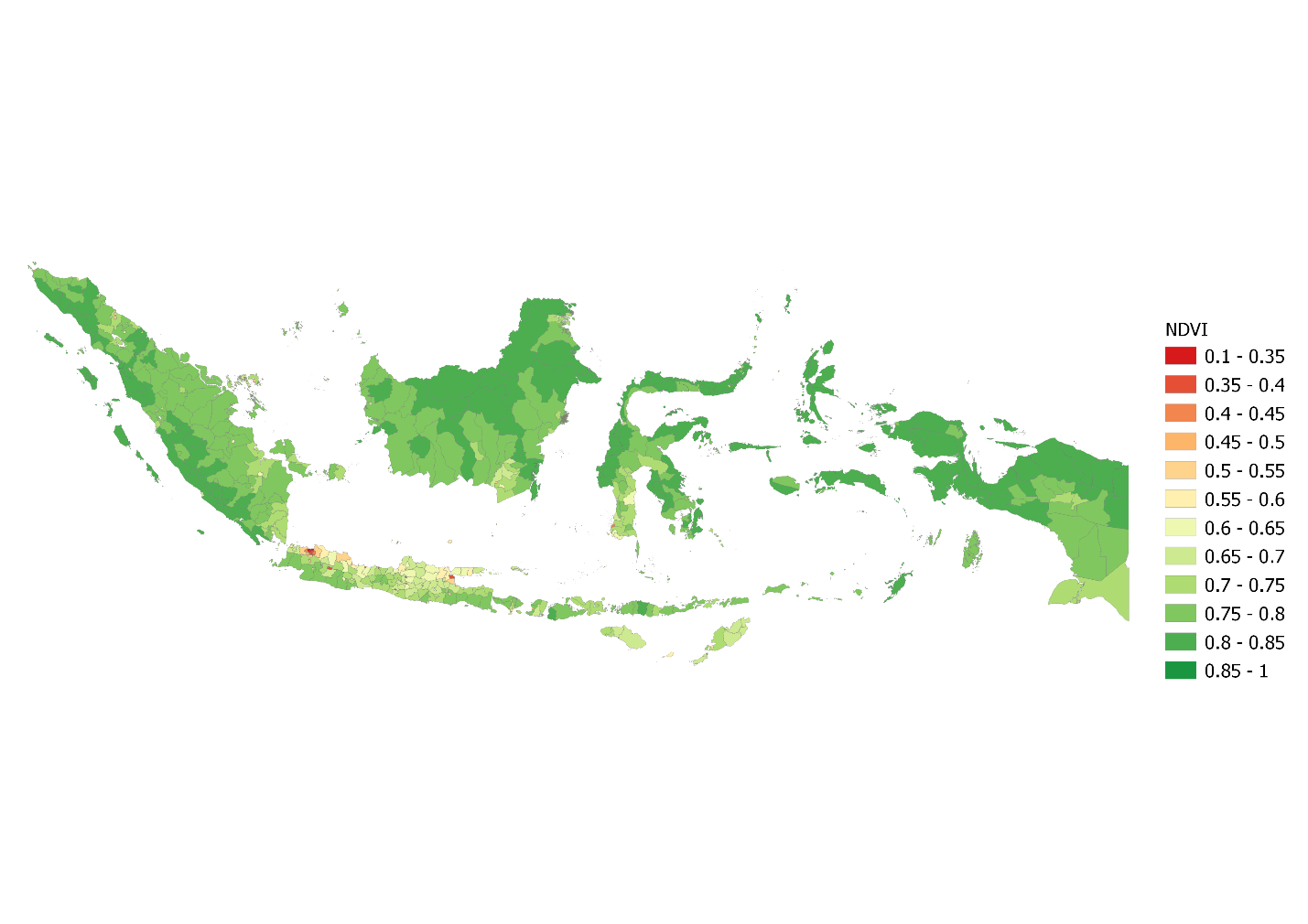
2014
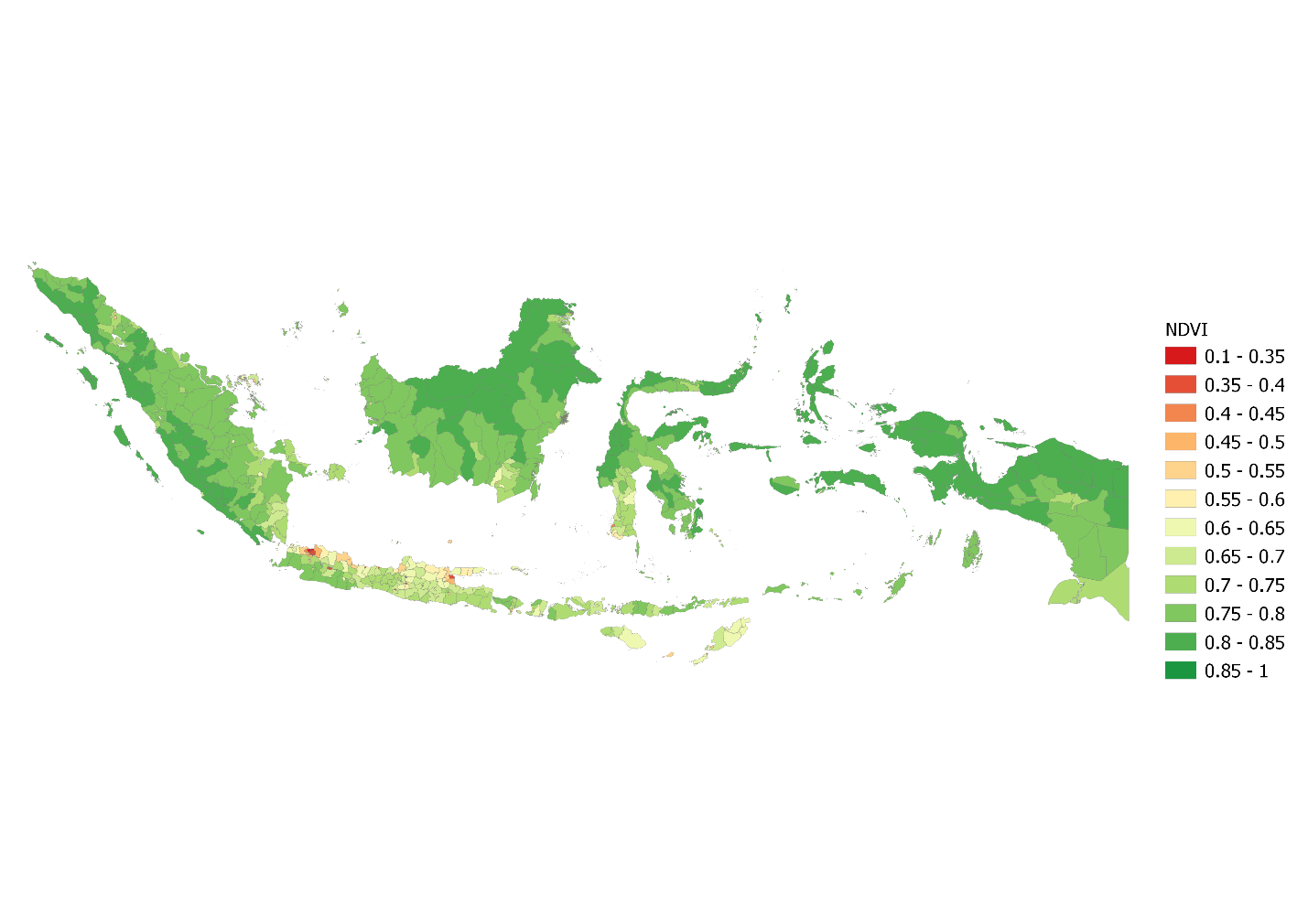


2015
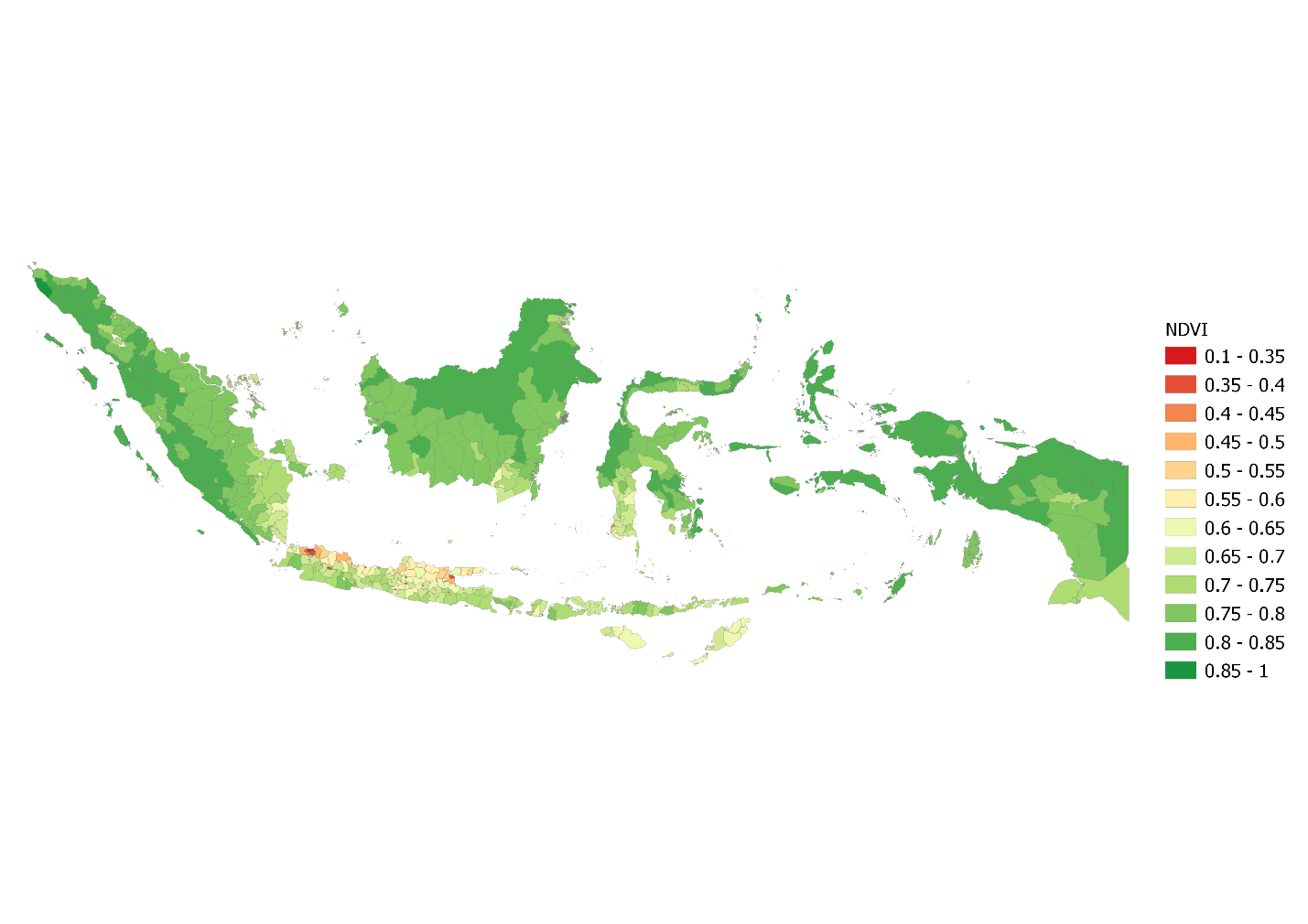
2016
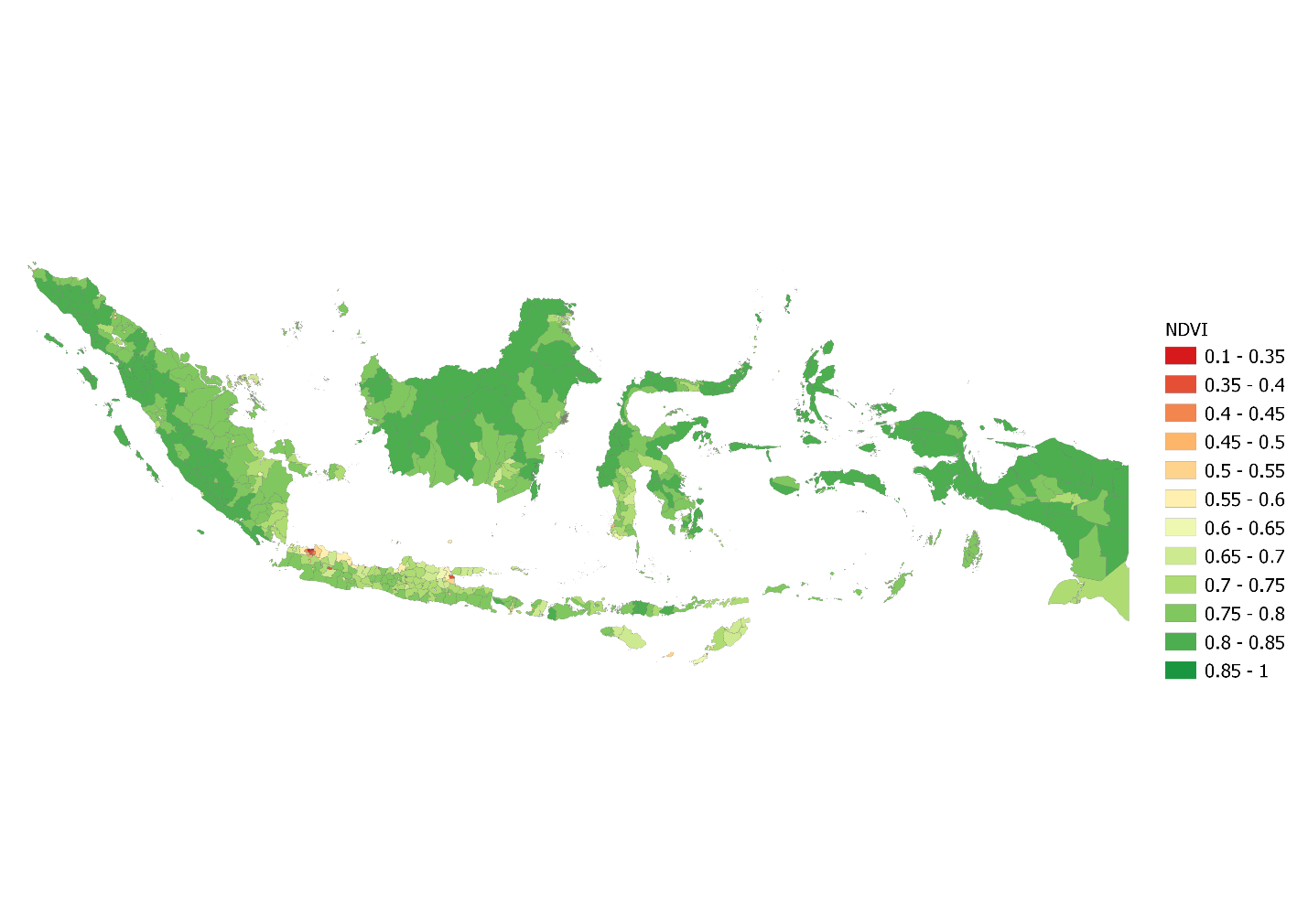


2017
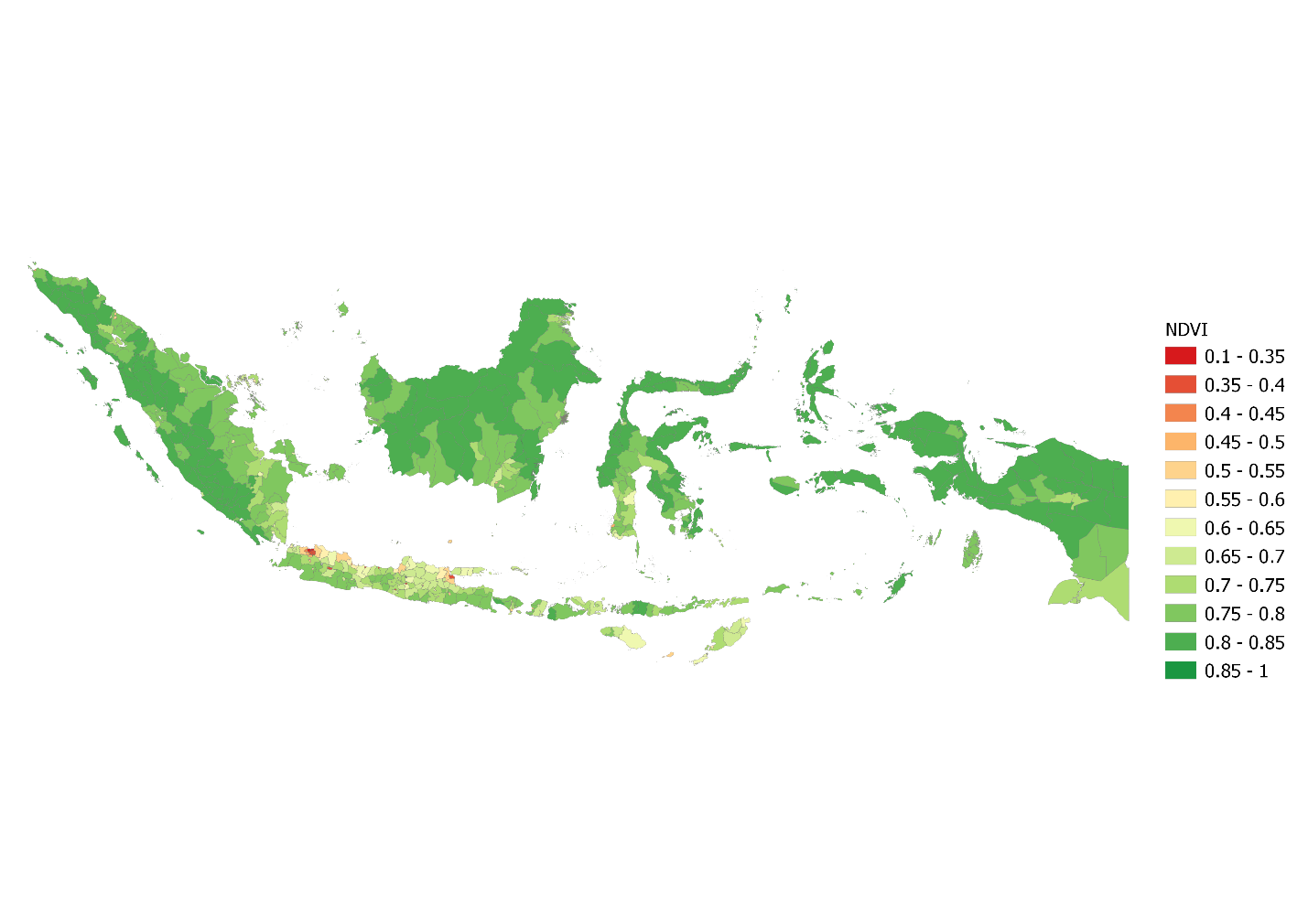
2018
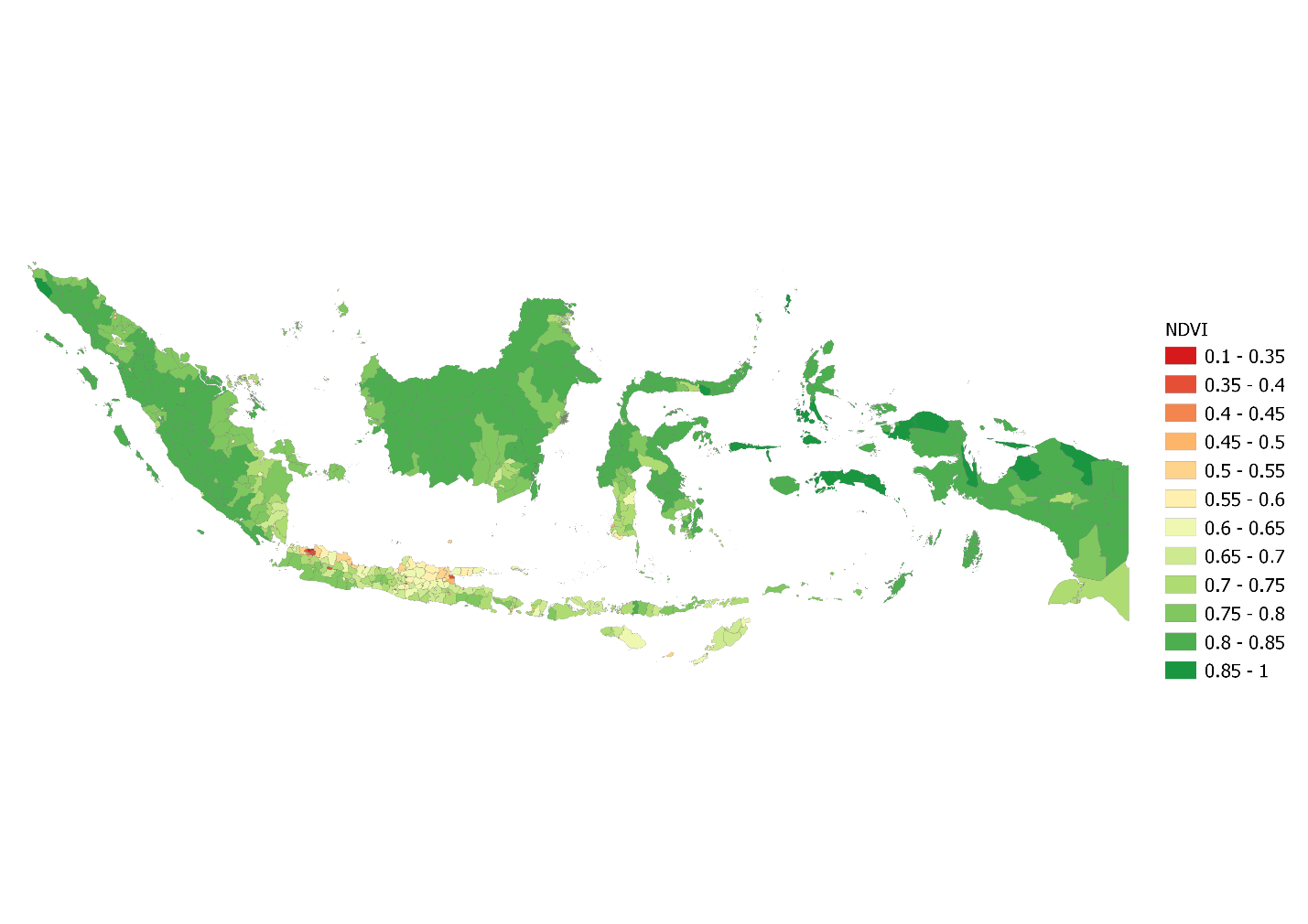


2019
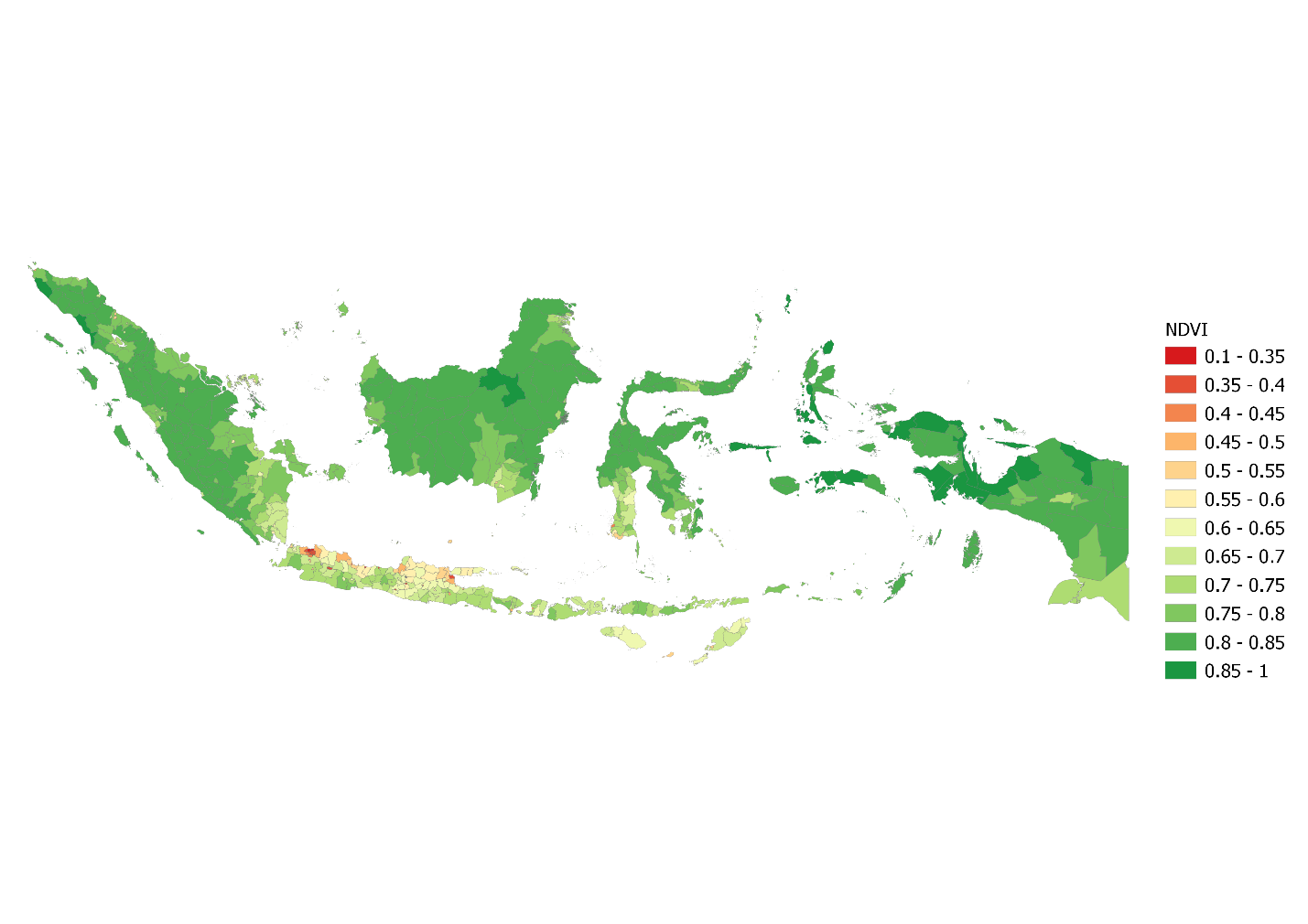
2020
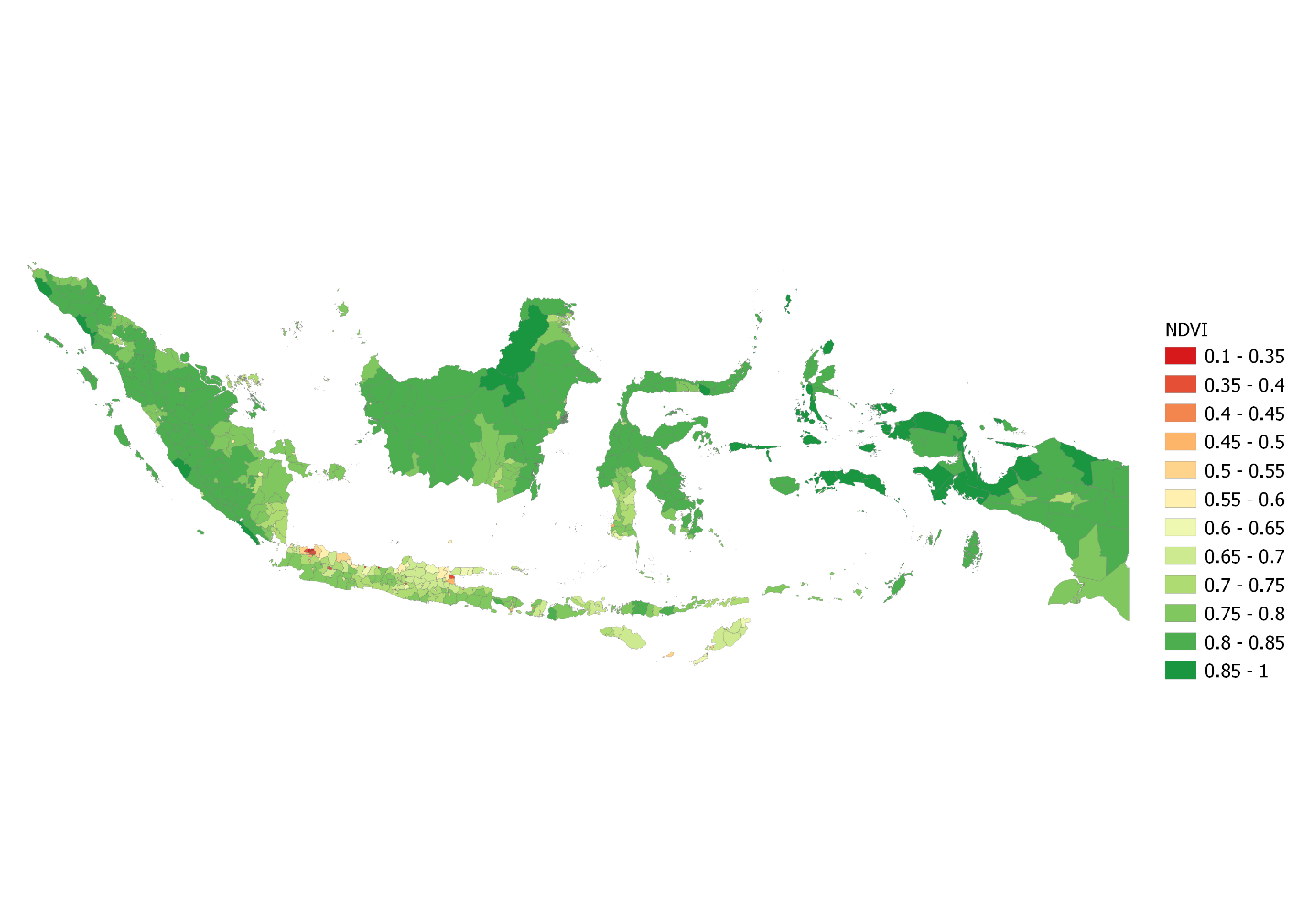

Supplement: Supplementary file 5 — Supplementary Figure S4. [file 41598_2023_35330_MOESM5_ESM.docx]
