# Supplementary material for: Time-series analysis of satellite imagery for detecting vegetation cover changes in Indonesia
Source: Sci Rep. 2023 May 25;13:8437. doi: 10.1038/s41598-023-35330-1 (PMC10212945; doi:10.1038/s41598-023-35330-1)
Supplement: Supplementary file 7 — Supplementary Figure S5. [file 41598_2023_35330_MOESM7_ESM.docx]

Figure S5. Maps of the socioeconomic and demographic status of each regency/city in Indonesia. This map was created by TF using QGIS 3.22.4 Białowieża software and open administrative boundary data published by OCHA HDX.


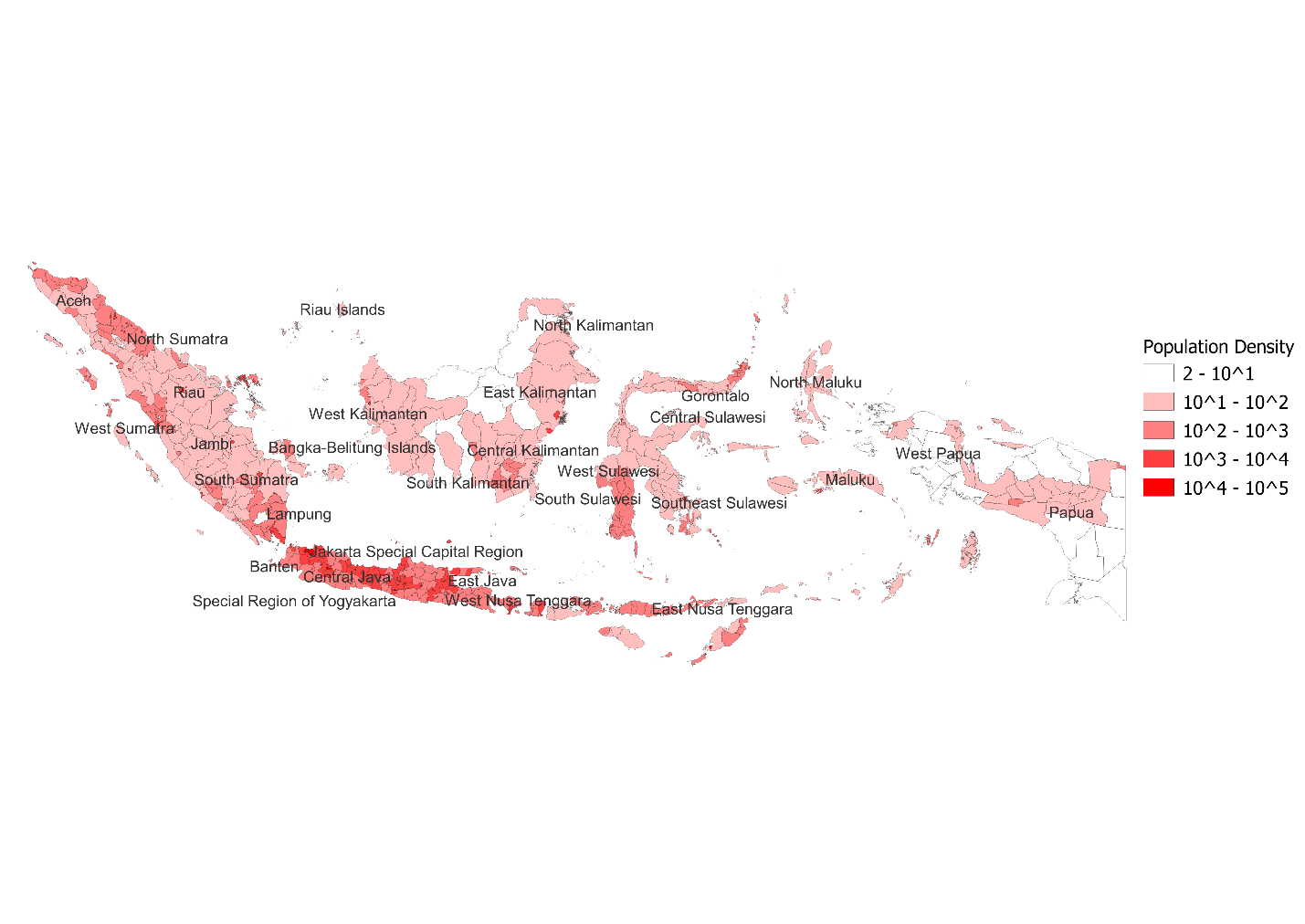


Map of population densities at regencies/cities in Indonesia


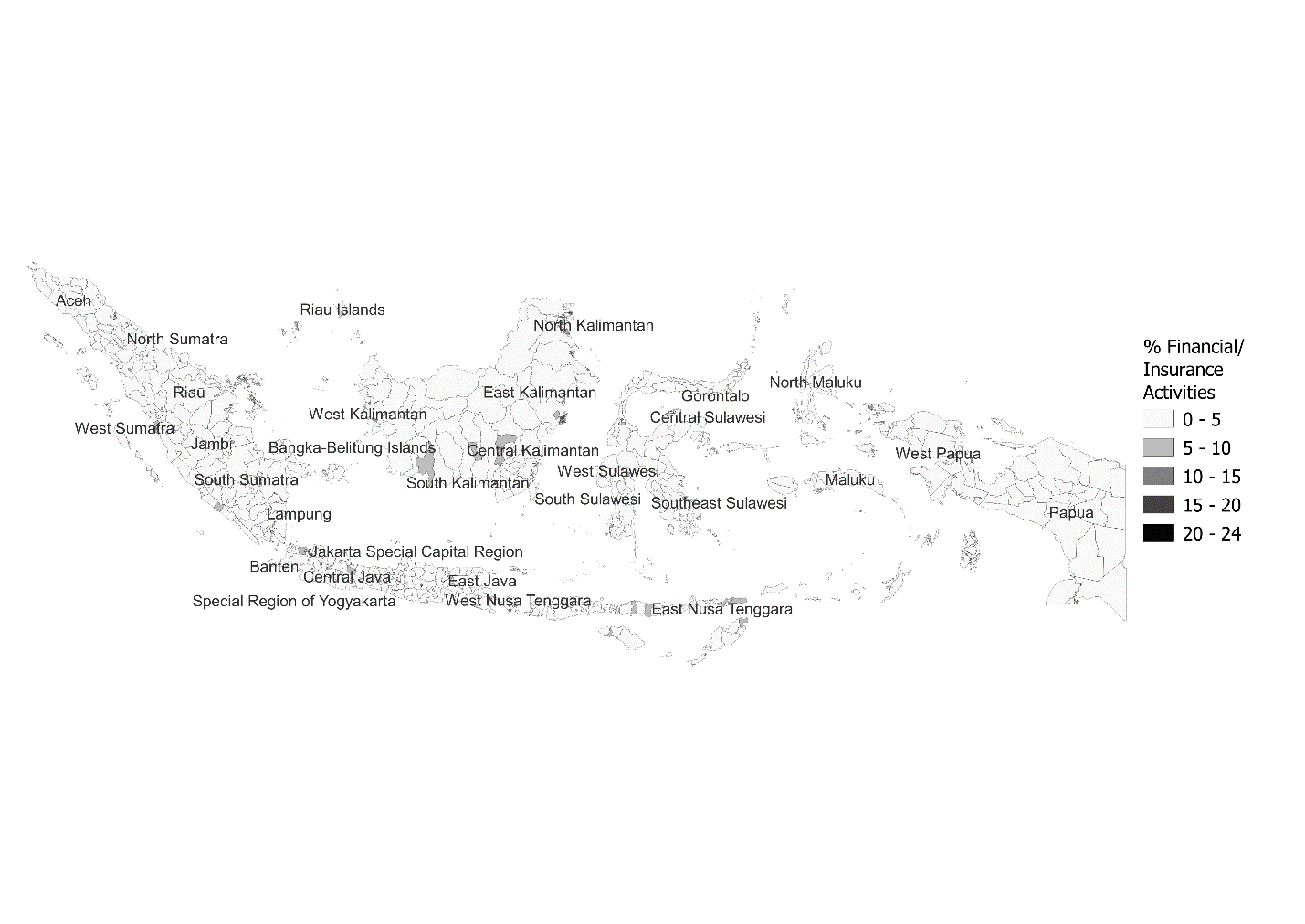


Map of the proportions of GDP from financial and insurance activities at regencies/cities in Indonesia


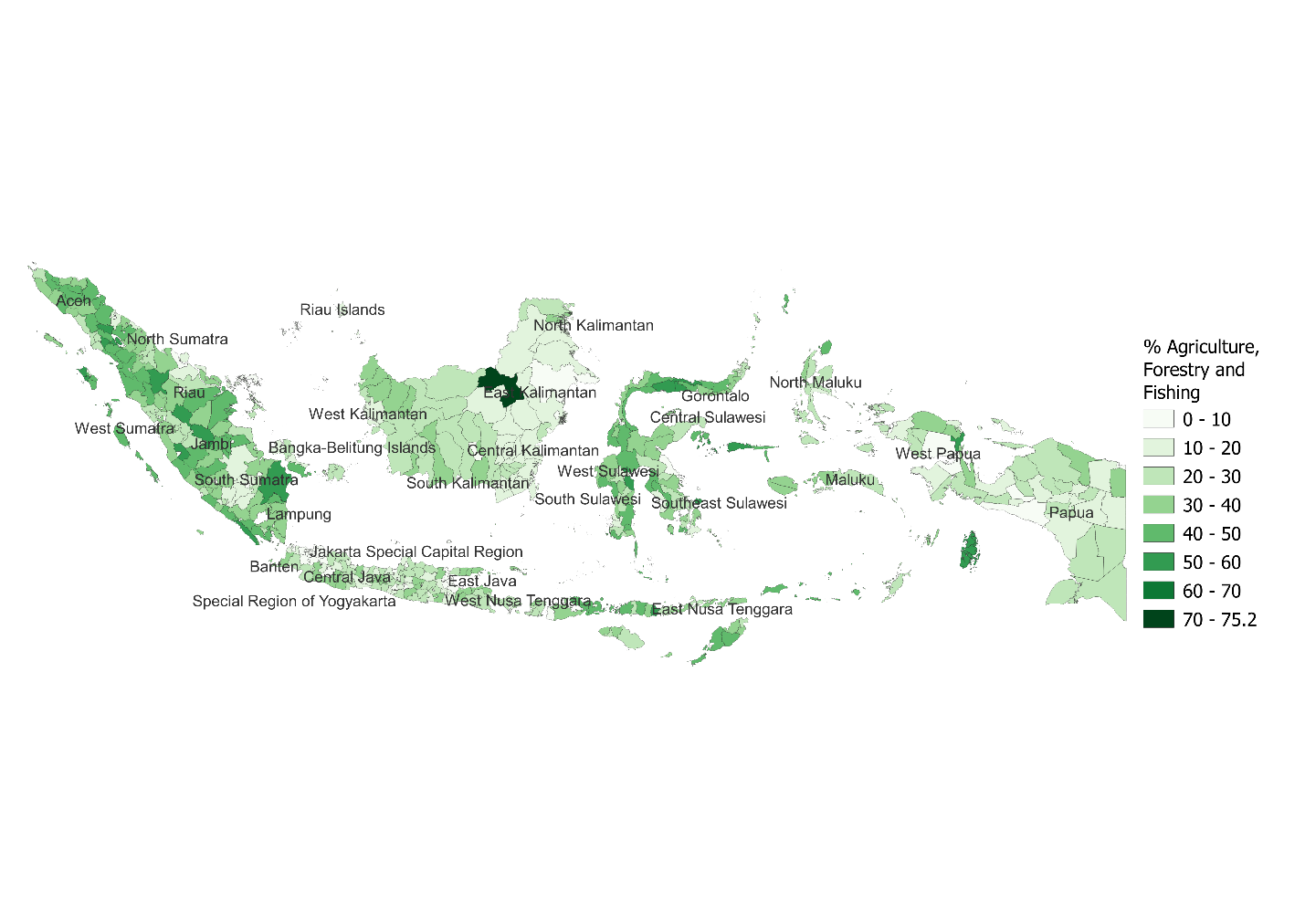


Map of the proportions of GDP from Agriculture, Forestry, and Fisheries at regencies/cities in Indonesia
