# Supplementary material for: Time-series analysis of satellite imagery for detecting vegetation cover changes in Indonesia
Source: Sci Rep. 2023 May 25;13:8437. doi: 10.1038/s41598-023-35330-1 (PMC10212945; doi:10.1038/s41598-023-35330-1)

Figure S6. Univariate correlations: (a) among the NDVI change trend, rainfall level (average rainfall per month), rainfall correlation (trend with time), population density, %GDP from financial and insurance activities, and %GDP from agriculture, forestry, and fisheries; and (b) among the NDVI value change, rainfall level (average rainfall per month), rainfall difference (difference in trend between 2001 and 2020), population density, %GDP from financial and insurance activities, and %GDP from agriculture, forestry, and fisheries.

(a)


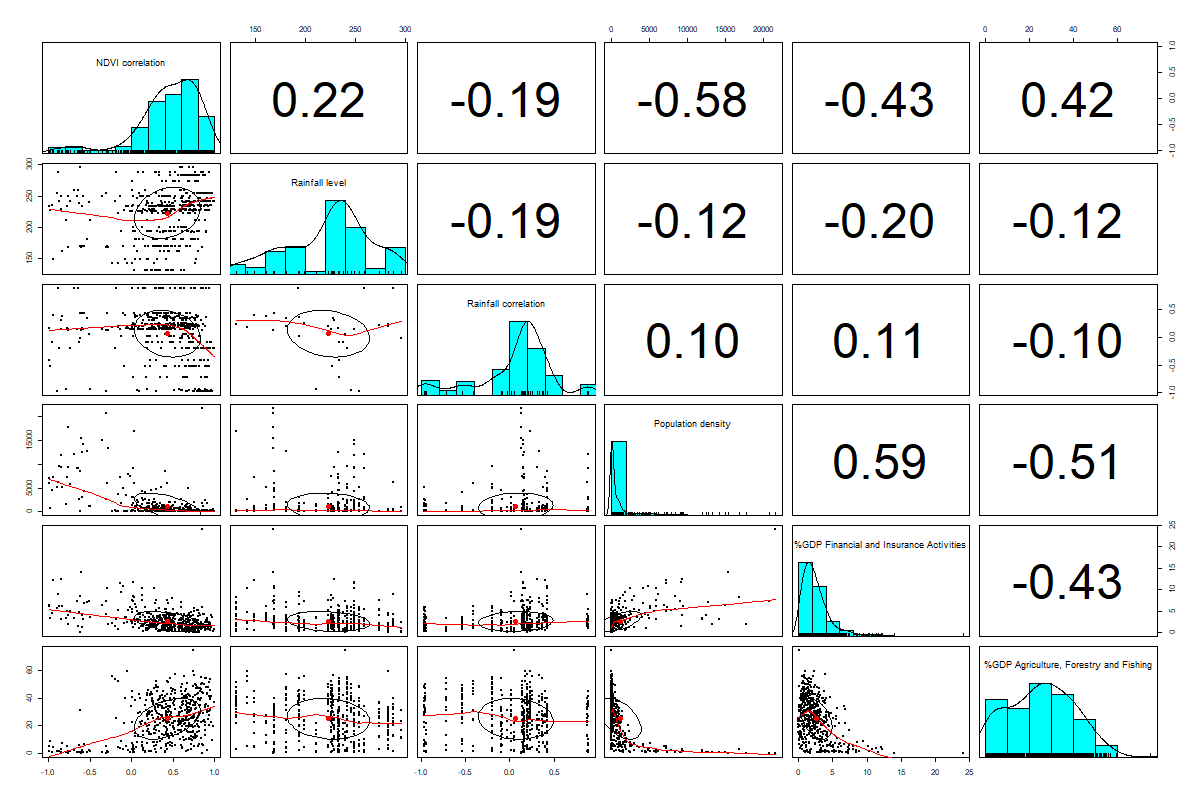


(b)


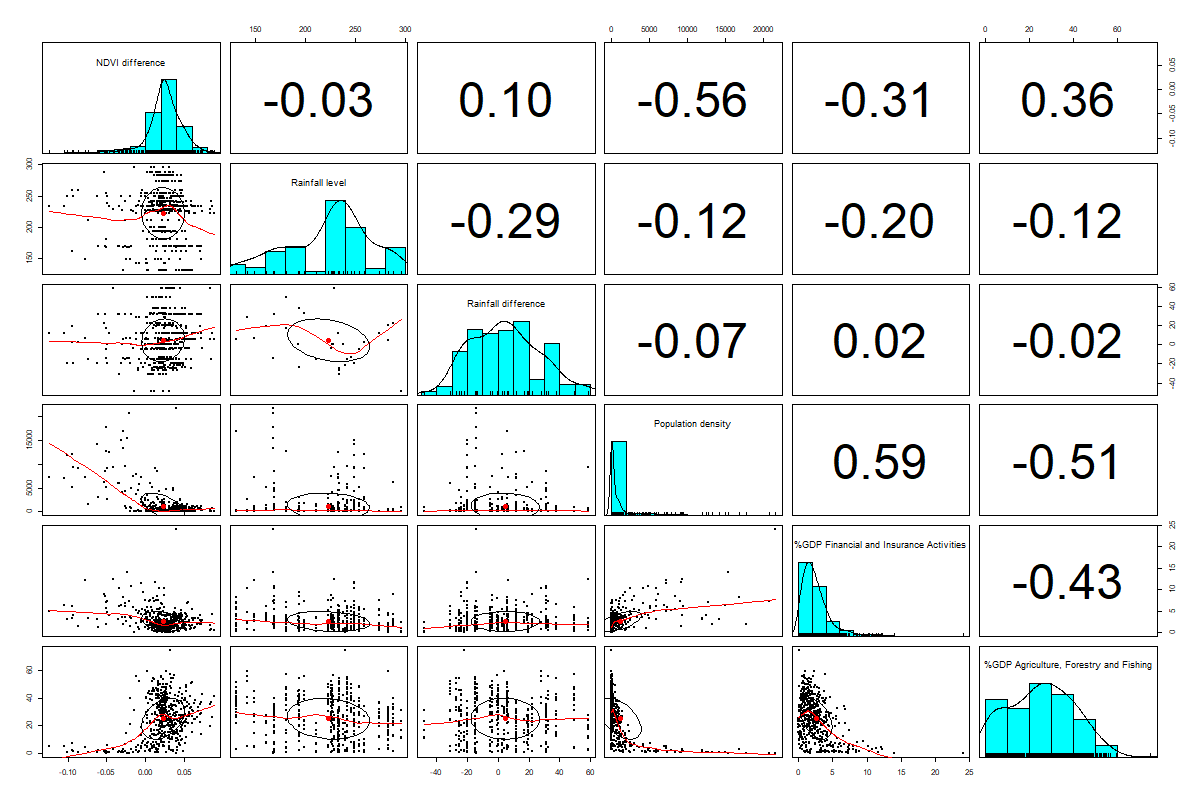

Supplement: Supplementary file 9 — Supplementary Figure S6. [file 41598_2023_35330_MOESM9_ESM.docx]
